# A Modular Rapid-Injection NMR for Structure Elucidation and Kinetic Analysis

**DOI:** 10.1021/acs.orglett.6c02870

**Published:** 2026-09-17

**Authors:** Andrew J. Ressler, Han-Hsiang Hsu, Andy A. Thomas

**Affiliations:** Department of Chemistry, 2655 Texas A&M University, College Station, Texas 77843-3255, United States

## Abstract

Rapid-injection nuclear magnetic resonance (RI-NMR) spectroscopy transforms a standard NMR tube into a reaction vessel within the NMR spectrometer, enabling kinetic and spectroscopic characterization of intermediates under synthetically relevant conditions. Despite its versatility as a tool for mechanistic analysis, the broad adoption of RI-NMR has been hindered by the lack of cost- and space-efficient instrument designs, which can be operated with negligible interference to the NMR facility. Herein, we describe an RI apparatus that is compatible with most NMR spectrometers, requires no modification of the NMR instrument or probe, and may be easily transported to and from the laboratory. Accurate reagent delivery is achieved across a range of solvents and temperatures, and samples can be mixed within seconds after injection. The utility of this apparatus is highlighted through four case studies chosen to illustrate different aspects of the instrument.

## Graphical abstract

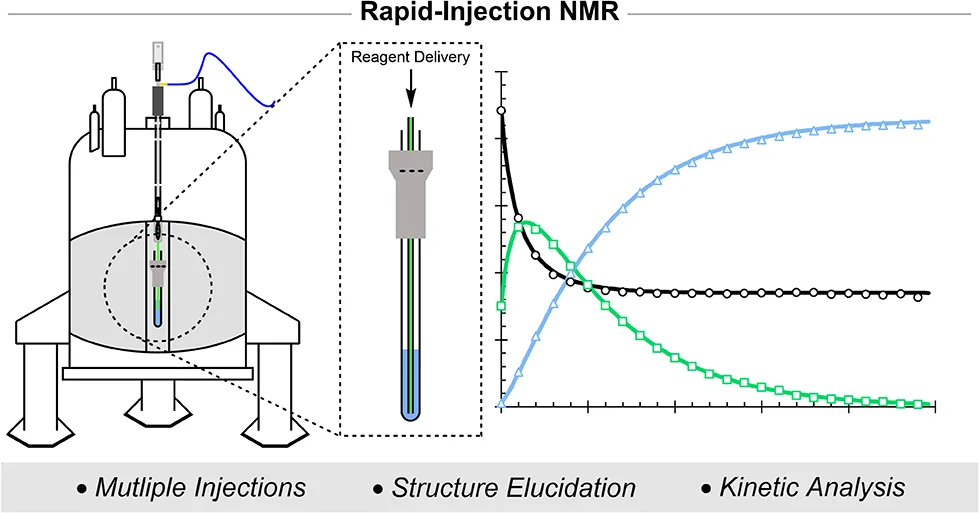

## Introduction

The introduction of Fourier-transform nuclear magnetic resonance (NMR) spectroscopy to organic chemistry in the 1960s revolutionized the way chemists characterize novel compounds and identify reactive intermediates in applications as far-ranging as polymer, biological, and synthetic chemistry.^1–3^ For synthetic chemists, in particular, reaction monitoring within the NMR spectrometer permits the collection of accurate kinetic data under conditions mimicking those possible on the bench, offering a unique opportunity to gain a detailed understanding of reaction mechanisms and enabling the identification and characterization of fleeting intermediates. Despite this potential, the acquisition of accurate kinetic data on fast reactions (*t*_1/2_ < 5 min) remains experimentally challenging. To address this need, numerous NMR systems have been developed to minimize the time between sample mixing and data acquisition. In this context, continuous-flow^4–6^ and stopped-flow^7–10^ NMR systems have been successfully employed to elucidate complex mechanisms. Our laboratory routinely employs rapid-injection NMR (RI-NMR) for the acquisition of kinetic data for fast reactions with no modifications to the spectrometer itself.^11^ Conceptually, the RI-NMR apparatus consists of a calibrated syringe from which a reagent is injected into an uncapped NMR tube. The simplicity of this design offers numerous practical advantages with respect to experimental flexibility, especially when coupled with the ability of modern NMR spectrometers to precisely regulate sample temperature and protect samples under an inert atmosphere. These advantages have led to the limited adoption of RI-NMR in polymer chemistry,^12,13^ biological chemistry,^14^ and especially synthetic chemistry.^15–20^

Since the inception of the rapid-injection technique by McGarrity in 1981, the development of RI-NMR instrumentation has often been driven by specific experimental needs (Figure 1A).^11^ The original McGarrity apparatus consists of syringe mounted to a movable rail above the spectrometer, which is manually lowered during injection. The syringe is connected to a fluoropolymer tube that feeds into the NMR tube, through which reagent is delivered. McGarrity’s second-generation apparatus placed the reagent syringe immediately above the NMR sample tube inside the transfer tube – this modification decreases the amount of material lost as dead volume within the reagent tubing.^21^ A major hindrance to the adoption of this design is the requirement to mount the rail guide to the spectrometer itself, which necessitates a dedicated instrument for installation of the RI-NMR platform. Despite this, these devices have been used by a number of groups for studies of organolithium compounds,^22^ aldol condensations,^23^ and chelation effects.^24^ More recently, Reich and Denmark independently disclosed RI-NMR platforms, each of which incorporated additional features to solve specific experimental problems. The Reich apparatus features two mounted syringes enabling sequential injections of two different reagents in a single experiment, in addition to a mechanical stirrer to improve sample mixing; this apparatus was specifically designed for the analysis of low-intensity heteronuclear spins in relatively large solution volumes.^25^ While this design provides more experimental flexibility with respect to reagent addition, it requires the use of 10 mm NMR tubes, and the authors note that each syringe can only deliver a single injection during the course of an experiment. Comparatively, the Denmark RI-NMR delivers precise quantities of any liquid reagent under rigorously inert conditions to 5 or 10 mm tubes but is limited operationally to the injection of one solution during an experiment.^26^

**Figure 1:**
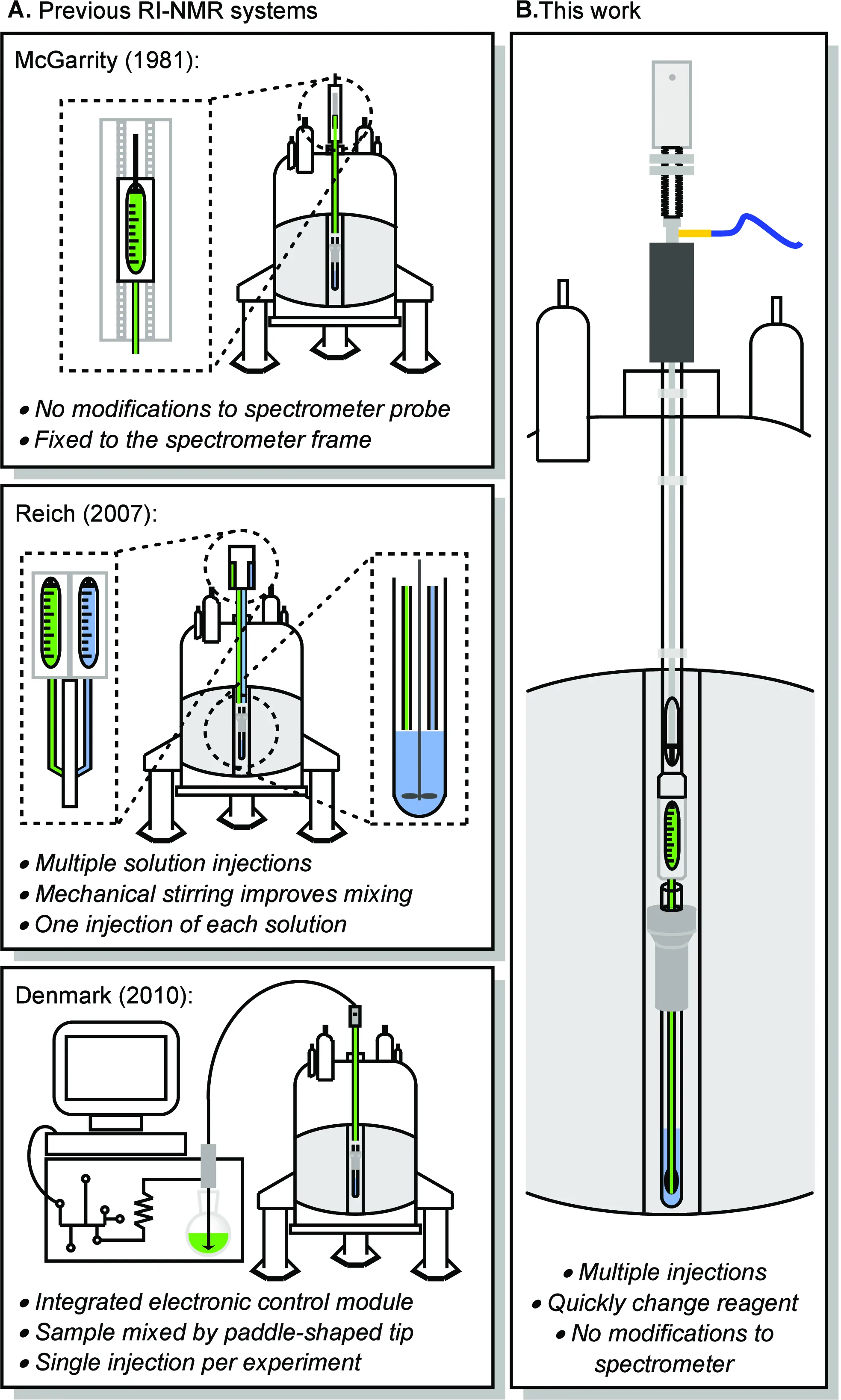
(A) Previous RI-NMR designs. (B) This work: modular and versatile RI-NMR.

Given our interest in mechanism-guided reaction discovery facilitated by RI-NMR^27,28^ and expertise in the design of rapid injection systems,^29,30^ we sought to design a standardized RI-NMR apparatus capable of meeting a wider range of experimental demands (Figure 1B). Specifically, we desired a device that would (1) deliver precise quantities of liquid reagents across various temperatures and solvent viscosities, (2) enable sequential injections of a single solution at any desired volume, (3) be rapidly reconfigured to introduce different reagents during the course of a single RI-NMR experiment, and (4) be user-friendly and easily replicated by other laboratories. Here, we disclose an instrument that fulfills these criteria and demonstrate its application toward structure elucidation and kinetic analysis.

## Design of the RI-NMR

Taking inspiration from the RI-NMR systems of McGarrity, Reich, Ogle,^31^ and Denmark, we developed the instrument shown in Figure 2. Full schematics and construction details are presented in Supporting Information section 2.

**Figure 2:**
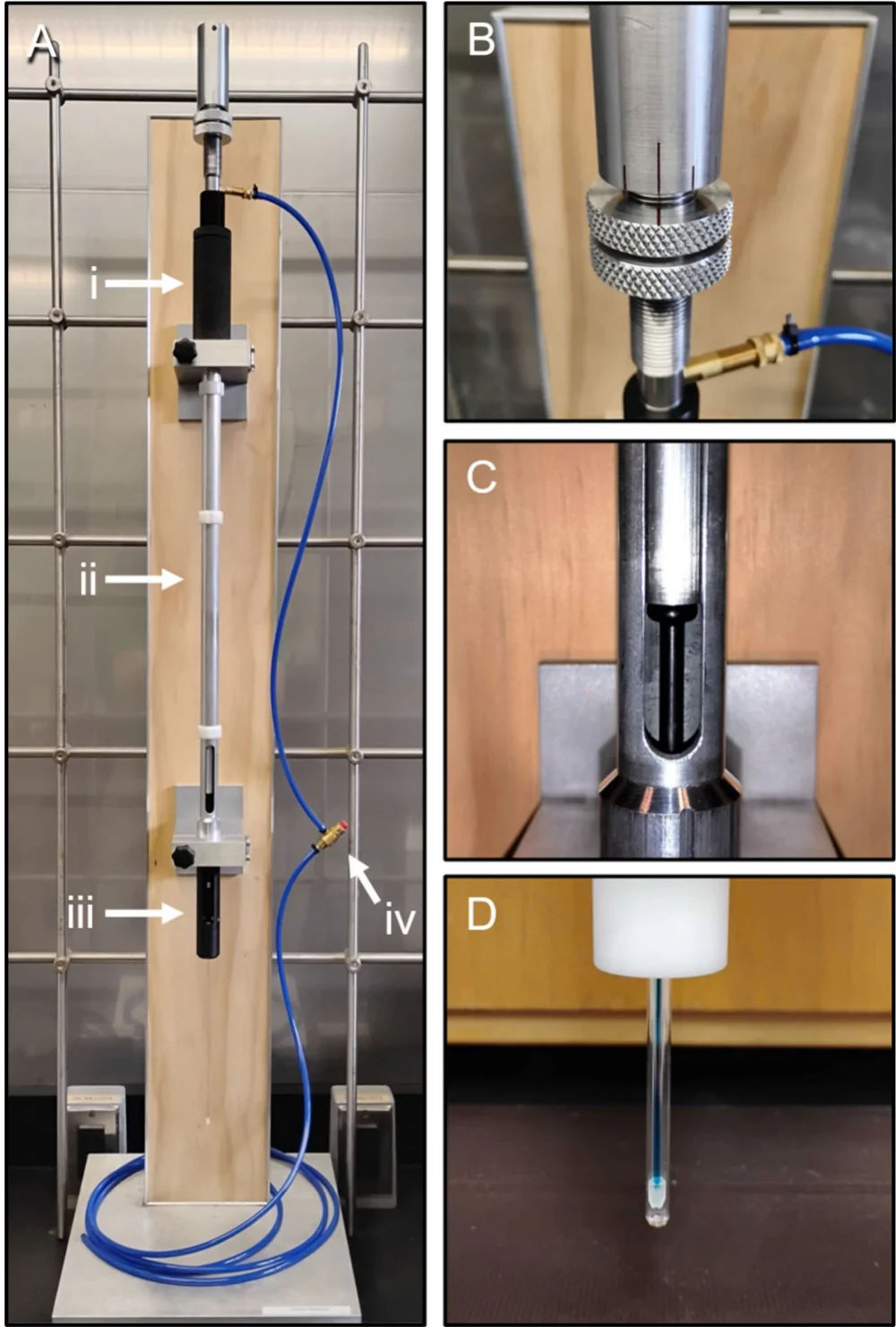
(A) Complete RI-NMR apparatus: (i) Depth-adjusting handle and piston. (ii) Aluminum NMR insert barrel and Teflon alignment disks. (iii) 1 mL syringe housing. (iv) Pneumatic trigger. (B) Volume set screw. (C) Syringe plunger attached to a piston rod. (D) Glass mixing capillary tip inside an NMR tube.

Our design consists of a piston-driven syringe positioned immediately above an uncapped NMR tube and connected to a 20.3 cm glass capillary that delivers the injected solution into the bottom of the NMR tube (Figure 2).

The body of the apparatus consists of a polyacetal depth-adjusting handle [Figure 2A­(i)], which houses a pneumatic piston for delivering the reagent. This is connected to an aluminum barrel ringed with Teflon alignment disks [Figure 2A­(ii)], which ensure proper alignment of the capillary into the NMR tube and allow the RI-NMR to be adapted for use in Bruker, Varian, or JEOL spectrometers. Finally, the bottom of the barrel connects to a chemical-resistant PEEK syringe housing [Figure 2A­(iii)], which holds the syringe in alignment above the NMR tube. The piston is connected to house nitrogen through a gas line operating at 276 kPa, and is pneumatically actuated by depressing a relief valve button [Figure 2A­(iv)]. When the button is pressed, the upper piston arm is lowered by a distance determined by a calibrated volume wheel (Figure 2B). The lower piston arm is connected to the syringe plunger by an aluminum rod inside the barrel (Figure 2C), allowing for precise reagent delivery. Finally, a glass capillary was designed to improve mixing efficiency (Figure 2D), which can be readily prepared in-house and resembles a device previously reported by Lloyd-Jones for heterogeneous sample mixing.^32^ The simple and lightweight (<1 kg) device can be easily transported and disassembled as needed, and is entirely manually operated, requiring no modifications to the spectrometer.

Two significant features of this design are (1) the ability to perform sequential injections of a single reagent solution by simply readjusting the volume wheel and (2) the ability to quickly exchange the injection syringe by detaching the syringe assembly and replacing it with a second assembly loaded with a different reagent. This operation can be performed in less than 1 min and allows sequential injection of multiple reagents over the course of a single experiment (see Supplemental Video S1). Additional details regarding the instrument, including standard procedures, capillary construction, calibration curves, sample premagnetization studies, and temperature changes upon injection, can be found in Supporting Information sections 3–7.

## Applications

### Multiple Injection RI-NMR for Synthetic Sequences

To demonstrate the multiple-injection capabilities of our RI-NMR system, we revisited the sodiation selectivity of pyridine—a transformation recently developed in our laboratory (Figure 3).^27^ Specifically, we sought to perform three sequential injections at cryogenic temperatures in order to investigate: (Injection 1) the formation of kinetic deprotonation products; (Injection 2) the generation of thermodynamic deprotonation products; (Injection 3) the electrophilic capture of the thermodynamic carbanion mixture with an alkyl halide.

**Figure 3:**
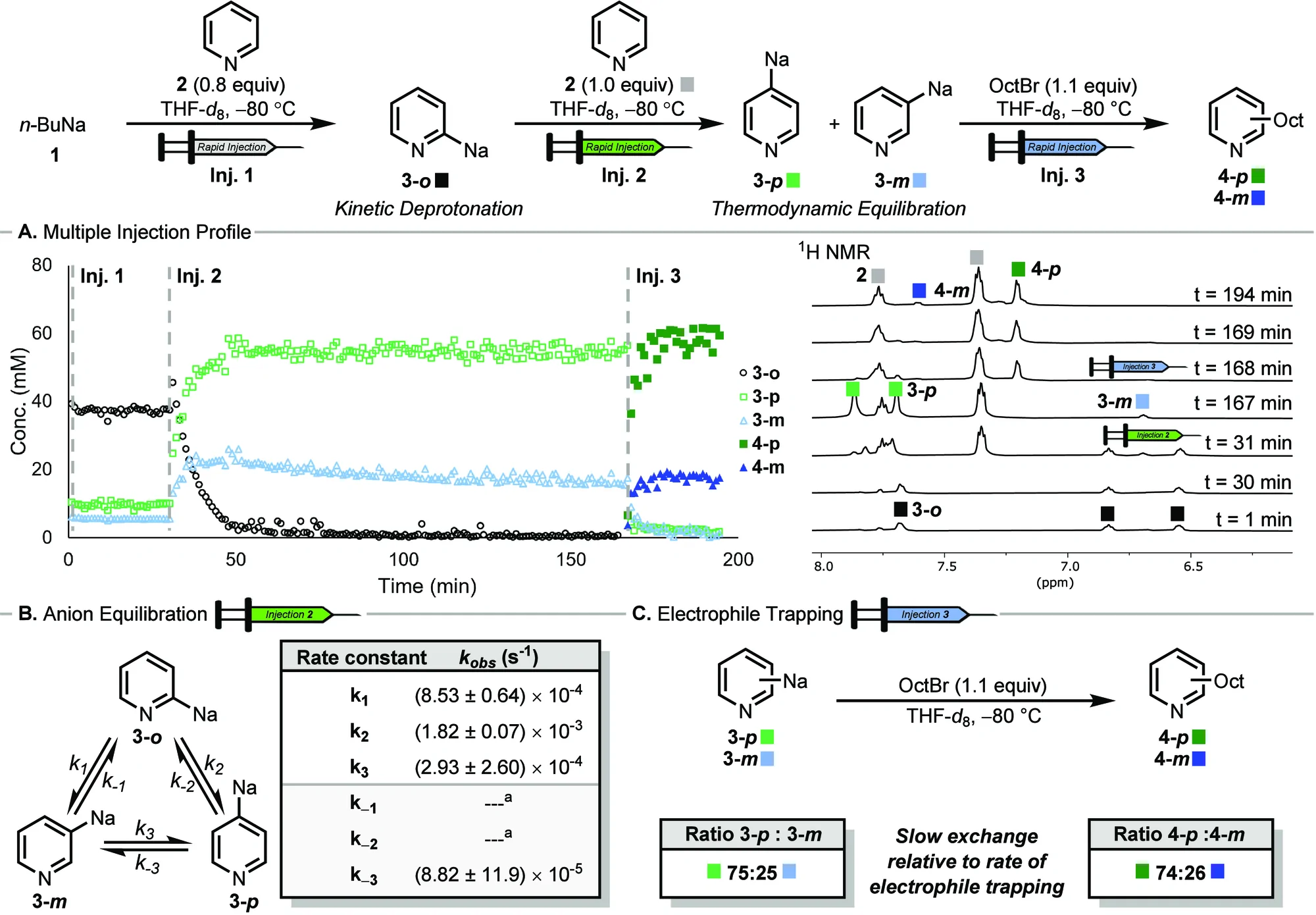
Exploratory RI-NMR study of *n*-BuNa-mediated deprotonation/trapping of pyridine in a single NMR tube. ^a^Kinetic constant constrained to zero.

First, pyridine **2** (0.80 equiv) was injected into a precooled solution of *n*-BuNa **1** in THF-*d*_8_ at −80 °C. In contrast to the expected statistical distribution of pyridyl anion isomers, **3**-*o* was formed as the primary deprotonation product (71%), indicating that the deprotonation of **2** at the ortho position is favored kinetically. Furthermore, no change in the isomeric distribution was observed over 30 min, suggesting that a proton source (e.g., excess pyridine) was essential to achieve a thermodynamic mixture of anions.

Injection of excess **2** (1.0 equiv) resulted in rapid equilibration leading to the preferential generation of **3**-*p* (73%) and **3**-*m* (27%) (Injection 2). Global fitting according to a first-order profile afforded observed rate constants in agreement with those previously reported. Based on these kinetic constants, the thermodynamic ratio of **3**-*p*:**3**-*m* was determined to be 78:22. Isomers **3**-*p* and **3**-*m* were characterized on the basis of correlation spectra (COSY) obtained after the equilibration period in a separate experiment.

The ability to quantify both **3**-*p* and **3**-*m* in a single solution afforded a valuable opportunity to investigate the reactivity of each isomer toward alkylation. During the same NMR experiment, injection of 1-bromooctane (1.1 equiv) led to rapid consumption of both isomers within 1 scan. While we are unable to determine precise rate constants for each step,^33^ we confirmed that the ratio of anion isomers is retained during the trapping step, and rapid re-equilibration of anions is not operative. This scouting study highlights the power of RI-NMR to investigate multistep synthetic sequences with exquisite detail in a single NMR experiment and allows for rapid generation and refinement of mechanistic hypotheses. Focused investigations of each individual step are ongoing.

### Multiple-Injection RI-NMR for Polymerization Kinetics

The ability to perform sequential injections of different reagents provides a valuable opportunity to investigate polymer propagation kinetics in an evolving polymer architecture. As an example, we investigated the rate of polymerization of two norbornene-derived monomers **6** and **8** during the Ru-catalyzed ring-opening metathesis polymerization (ROMP) reported by Guironnet et al. (Figure 4).^34^ Complete consumption of monomer **6** was observed within 5 min after injection (*k*_1_ = 2.04 × 10^–2^ s^–1^, Supporting Information section 8.2.6) comparable to the reported value obtained by manual sampling (*k* = 2.00 × 10^–2^ s^–1^). Subsequent injection of monomer **8** into the same tube afforded diblock copolymer **9** (*k*_2_ = 1.53 × 10^–2^ s^–1^).

**Figure 4:**
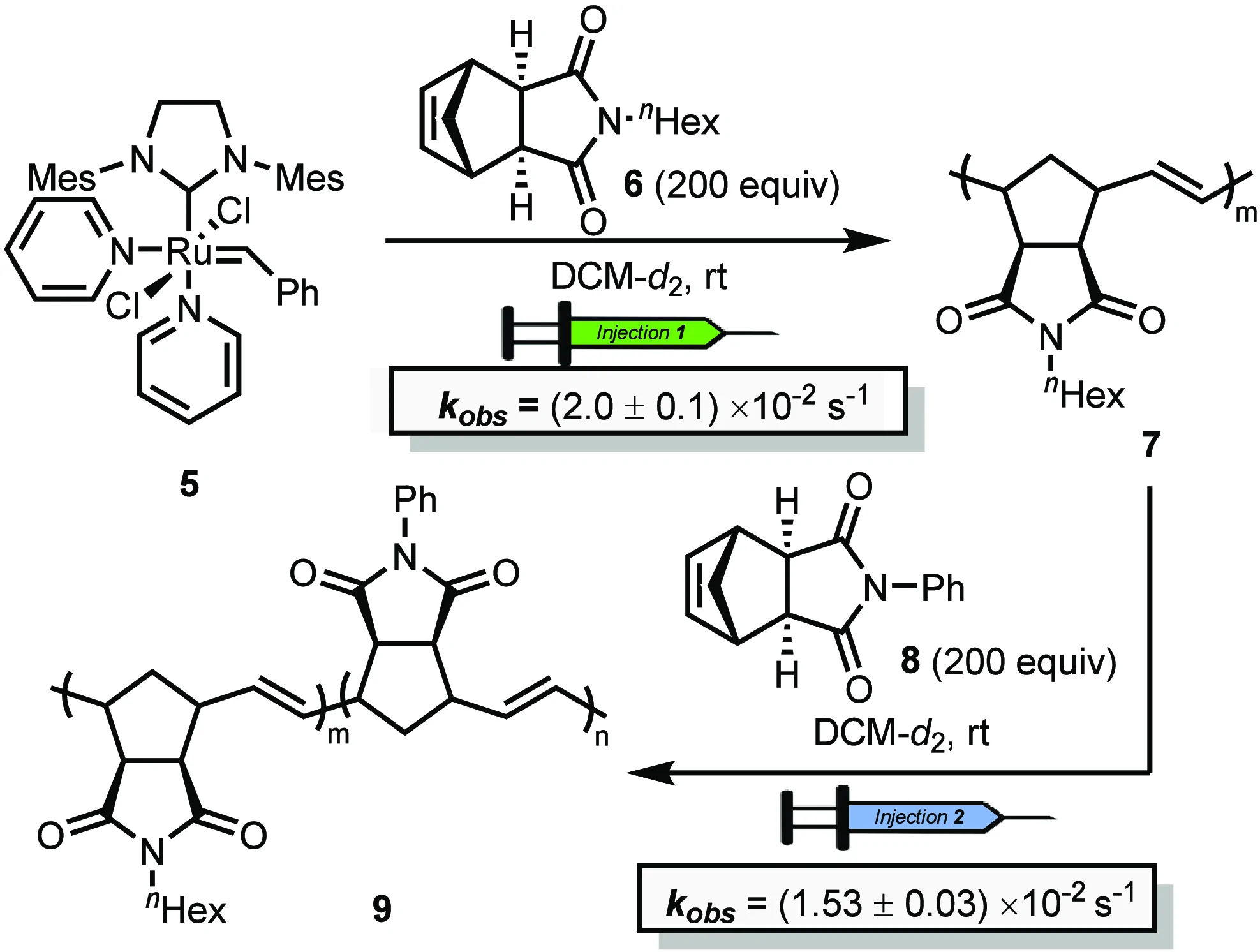
Ru-catalyzed ROMPs. Kinetic data are an average of three experiments.

### High-Temperature Injections

While most RI-NMR studies reported to date employ low temperatures, RI-NMR is equally useful for high temperature kinetic studies. To demonstrate the injection accuracy of the RI apparatus at high temperature, we studied the base-catalyzed isomerization of glucose recently reported by Tessonier et al. (Figure 5).^35,36^ Injection of Et_3_N into a thermally equilibrated solution of glucose **10** (0.52 M in D_2_O, 80 °C) resulted in the formation of fructose **11**, with a glucose consumption rate constant *k*_obs_ = (1.91 ± 0.09) × 10^–3^ s^–1^.^37^ Experiments were conducted in triplicate with an injected catalyst loading of 12 ± 1%.

**Figure 5:**
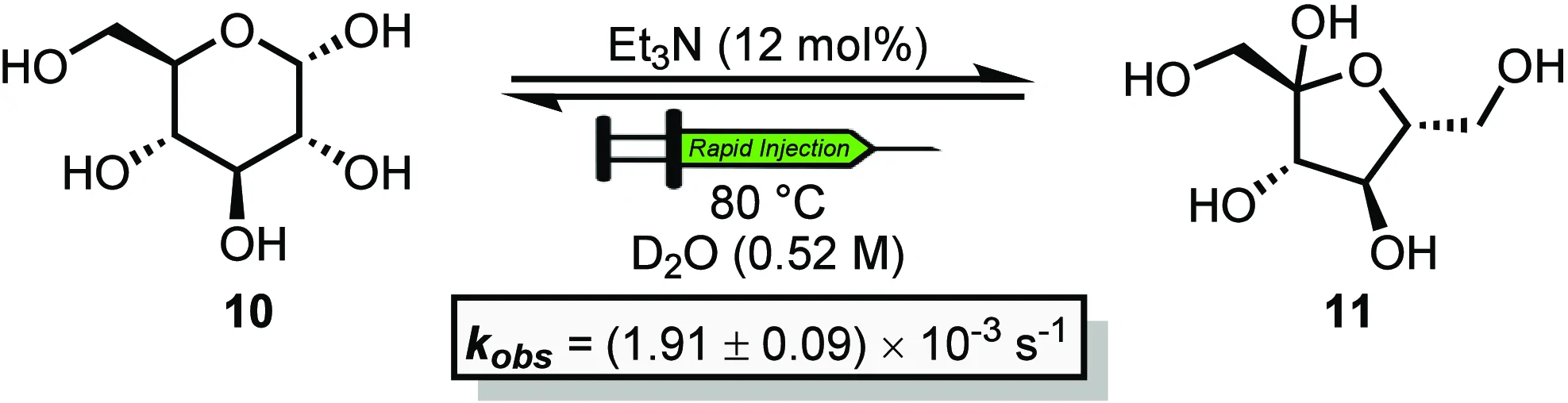
Base-catalyzed glucose isomerization. Kinetic data are an average of three experiments.

### Simplifying Spectral Complexity

While ^1^H NMR delivers valuable information for structural characterization, kinetic analysis by ^1^H NMR can be challenging due to peak overlap between structurally related species. Reaction monitoring by heteronuclei such as ^19^F can resolve intermediate species and ensure the collection of accurate kinetic data.^38,39^ To demonstrate this strategy, we briefly investigated the Mumm rearrangement with the goal of observing the imidate ester intermediate, as well as other potential intermediate species or side products.^40^ Upon injection of a Et_3_N-buffered solution of **13** into imidoyl chloride (**12**; 90 mM in CDCl_3_) at 25 °C, rapid formation of imidate ester (**14**) was observed,^41^ which decayed over several minutes to afford the rearranged product **15** (Figure 6). Initial profiling using ^1^H NMR enabled the observation of **14** (Figure S64). Subsequent reaction monitoring by ^19^F NMR enabled the collection of accurate kinetic data and permitted the detection of low-abundance species. Global fitting of the kinetic profile revealed that the formation of **14** proceeded through a second-order process (*k* = 1.30 ± 0.02 × 10^–2^ M^–1^ s^–1^), while rearrangement of **14** to **15** followed a first-order kinetic profile (*k* = 1.95 ± 0.01 × 10^–3^ s^–1^). No other intermediates besides **14** were detected by ^19^F NMR. Based upon these results, the mechanism of the Mumm rearrangement can be viewed as an initial bimolecular reaction between **12** and **13**, followed by a intramolecular migration.

**Figure 6:**
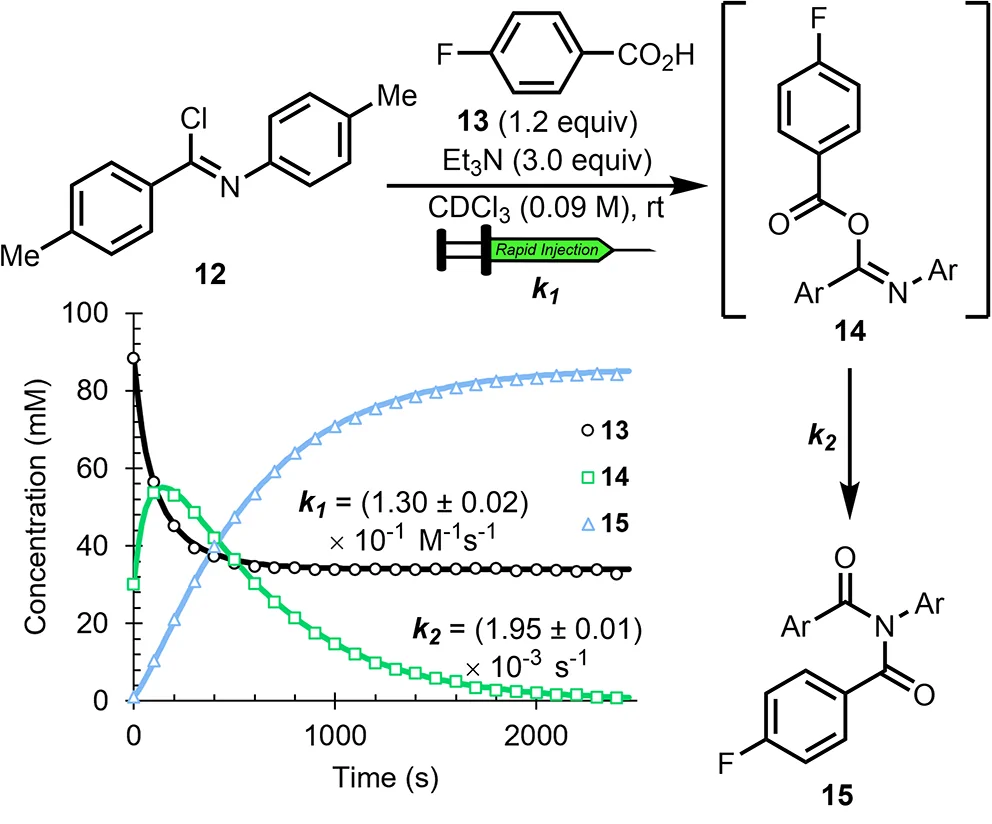
Mumm rearrangement. Kinetic data are an average of three experiments. Ar = *p*-tolyl.

## Conclusion

In summary, we present a modular RI-NMR apparatus that enables sequential injections of single or multiple reagent solutions with high accuracy. Due to its simple design and readily available components, we anticipate that this device will lower the barrier to adoption of RI-NMR as a tool for reaction discovery and mechanistic investigation.

## Supplementary Material



## Data Availability

The data underlying this study are available in the published article and its Supporting Information

## Notes

All experimental procedures, schematics for the RI-NMR apparatus, data analysis, compound characterization data, and captions for videos (PDF)

## Abbreviations

RI-NMR: rapid-injection nuclear magnetic resonance
GPC: gel-permeation chromatography
