# Supplementary material for: A Modular Rapid-Injection NMR for Structure Elucidation and Kinetic Analysis
Source: Org Lett. 2026 Sep 17;28(39):12159–64. doi: 10.1021/acs.orglett.6c02870 (PMC13640424; doi:10.1021/acs.orglett.6c02870)
Supplement: Supplementary file 1 [file ol6c02870_si_001.pdf]

A Modular Rapid-Injection NMR for Structure Elucidation and Kinetic Analysis

Andrew J. Ressler, Han-Hsiang Hsu, and Andy A. Thomas*

Department of Chemistry Texas A&M University, College Station, TX 77842-0001; United States.
*Correspondence to: andythomas@tamu.edu

1. Materials and Methods 4

1.1. Materials 4

1.2. NMR Spectroscopy 4

1.3. Gas Chromatography (GC) 4

2. Design of the RI-NMR System 5

2.1. Complete RI-NMR Assembly 5

2.2. List of RI-NMR Components 6

2.3 Schematics of RI-NMR Components 10

3. Using the RI-NMR 14

3.1. Description of the RI-NMR Apparatus 14

3.2. Preparing the RI-NMR for Use 15

3.2.1. Capillary Alignment 15

3.2.2. Setting and Monitoring the Capillary Depth 16

3.2.3. Loading the Syringe Assembly 17

3.2.4. Setting the Injection Volume 19

3.3. Performing an RI-NMR kinetics experiment 19

4. Capillary Tip Design and Calibration 20

4.1. Calibration Procedure 20

4.2. Simple Capillary (A) 20

4.3. Ringed Capillary (B) 21

4.4. Bulbed Capillary (C) 22

4.5. Arrowhead Capillary (D) 23

4.6. Collared Capillary (E) 24

4.7. Discussion of Capillary Performance 25

4.8. Fabrication of Capillaries 25

5. Additional Injection Calibrations 27

5.1. Room-Temperature Calibration Procedure 27

5.2. Solvent = *n*-Hexane 27
5.3. Solvent = Toluene 28
5.4. Solvent = DMF 29
5.5. Solvent = DMA 30
5.6. Solvent = THF, T = –80 °C 31
6. Sample Magnetization 32
6.1. Data Acquisition for Premagnetization Measurements 32
6.2. Monitoring a Species Present Initially (Reagent Injection) 32
6.3. Monitoring a Species Injected by RI-NMR (Substrate Injection) 33
6.4. Discussion of Sample Premagnetization 35
7. Temperature Variation During Injection 40
7.1. Calibration of NMR Temperature Control 40
7.2. Data Acquisition for Temperature Measurements 40
7.3. Effect of Injection on Sample Temperature 41
8. Experimental Procedures 42
8.1. Pyridine Deprotonation with *n*-BuNa 42
8.1.1. Experimental Procedure 42
8.1.2. Peak Identification 43
8.1.3. Data Acquisition 43
8.1.4. Data Analysis 44
8.1.5. Experiment Results 47
8.1.6. Original Data 48
8.2. Kinetic Study of Ru-Catalyzed Ring-Opening Metathesis Polymerization 53
8.2.1. Experimental Procedure 53
8.2.2. Peak Identification 54
8.2.3. Data Acquisition 55
8.2.4. Data Analysis 55
8.2.5. Experiment Results 56
8.2.6. Original Data 59
8.2.7. Synthesis of Monomers. 62
8.2.8. T_1 Measurements of Monomers. 63
8.3. Kinetic Study of Brønsted Base-Catalyzed Isomerization of Glucose 65
8.3.1. Experimental Procedure 65
8.3.2. Peak Identification 65

8.3.3. Data Acquisition......66
8.3.4. Data Analysis......66
8.3.5. Experiment Results......67
8.3.6. Original Data......70
8.4. Kinetic Study of the Mumm Rearrangement......76
8.4.1. Experimental Procedure......76
8.4.2. Peak Identification......77
8.4.3. Data Acquisition......78
8.4.4. Data Analysis......78
8.4.5. Experiment Results......80
8.4.6. Original Data......83
8.4.7. Synthesis of Starting Materials......92
8.4.8. T_1 Measurements......95
9. Video Captions......98
10. NMR Spectra......100
11. References......114

1. Materials and Methods

1.1. Materials

Tetrahydrofuran (Sigma-Aldrich, HPLC grade) was distilled over sodium/benzophenone. Pentane (Fisher Scientific, ACS grade) and toluene (Fischer Scientific, HPLC grade) was distilled over sodium. *n*-Hexane (VWR, HPLC grade), diethyl ether (VWR, inhibitor-free, for spectroscopy), and dichloromethane ((Fischer Scientific, stabilizer free) was purified by passage through alumina. D-Glucose was purchased from Amresco and used without purification. Triethylamine (HPLC grade) was purchased from Acros and used without purification. *n*-BuNa was synthesized following a previously reported procedure.[1] All deuterated solvents for NMR samples were purchased from Cambridge Isotope Labs. THF-d_8 was distilled over NaK (sodium/potassium alloy). Chloroform-*d* was used as received.

1.2. NMR Spectroscopy

^{1}H, ^{13}C, and ^{19}F NMR spectra for synthesized compounds were recorded on a Bruker Avance Neo 400 MHz spectrometer (^{1}H, 400 MHz; ^{13}C, 99 MHz; ^{19}F, 376 MHz). NMR spectra for rapid injection experiments were recorded on a Bruker Avance Neo 500 MHz NMR spectrometer (^{1}H, 500 MHz, ^{19}F, 471 MHz). Spectra for kinetics were referenced to 1,2-difluorobenzene (^{19}F NMR, –140.71 ppm) or tetramethylsilane (^{1}H NMR, 0.00 ppm) as internal standard. Chemical shifts are reported in ppm, multiplicities are indicated by s (singlet), d (doublet), t (triplet), q (quartet), p (pentet), h (hextet), hept (heptet), m (multiplet), and br (broad). Coupling constants, *J*, are reported in Hertz.

1.3. Gas Chromatography (GC)

Gas Chromatography (GC) spectra were recorded on an Agilent 8860 GC System using mass spectrometer (MS) (EI, single quadrupole mass filter). The standard method for reaction monitoring holds a temperature of 50 °C for 3 min and ramps up (10 °C/min) to a final temperature of 275 °C which is held for 5 min.

2. Design of the RI-NMR System

2.1. Complete RI-NMR Assembly

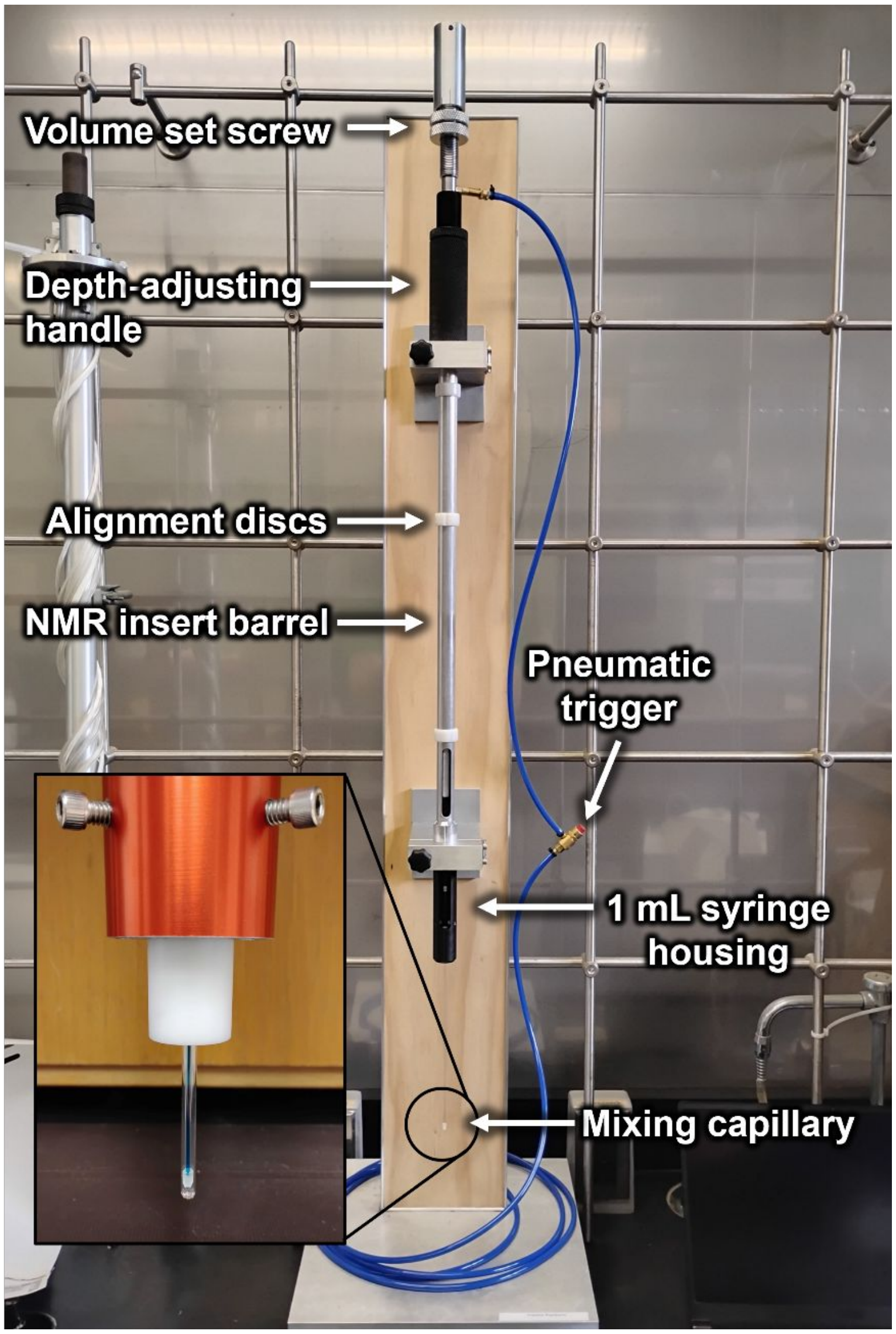

Figure S1. Complete RI-NMR apparatus.

2.2. List of RI-NMR Components

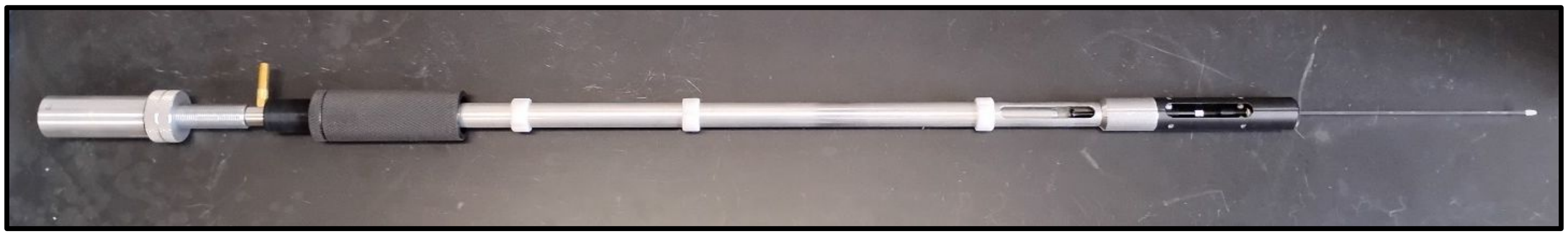

Figure S2. Complete RI-NMR assembly.

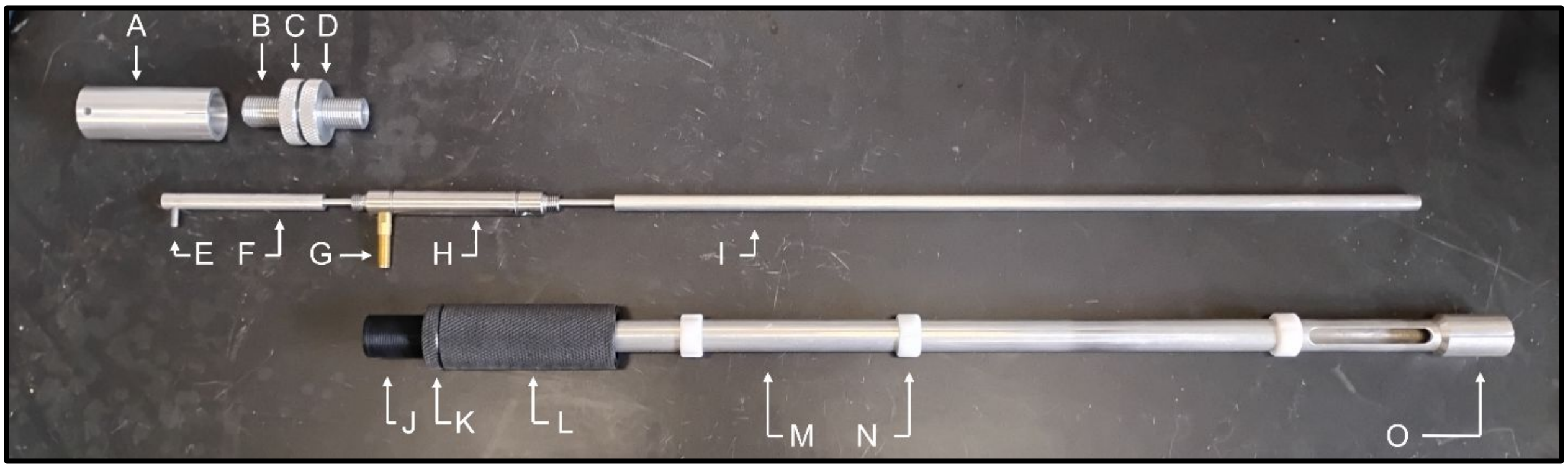

Figure S3. Overview of RI-NMR body.

Table S1. List of components and sources for RI-NMR body.

Label	Component Name	Description	Supplier	Part Number
**A**	Cap	Aluminum rod, 1 1/4" dia., 1 ft length	McMaster-Carr	8974K16
**B**	Volume gauge	Aluminum tube, 5/8 OD, 3/8 ID, 1 ft length	McMaster-Carr	9390N16
**C**	Volume wheel	Aluminum Rod 1-1/2" dia., 6” length	McMaster-Carr	8974K18
**D**	Volume locknut	Same as **C**	Same as **C**	Same as **C**
**E**	Cap screw	Cup-Point Set Screw 316 Stainless Steel, 10-32 Thread, 3/4"	McMaster-Carr	92313A832
**F**	Cap rod	Aluminum rod, 3/8" dia., 1 ft length	McMaster-Carr	8974K24
**G**	Valve	Quick Connect Valve, Body, #10-32M	Clippard	MQC-V2
**H**	Piston	9/16” Bore Double Acting Air Cylinder, 2” stroke	American Cylinder	562DESS-5
**I**	Rod	Aluminum rod, 3/8", 26" length	McMaster-Carr	4012N14

**J**	Piston housing	Acetal Copolymer Rods, 1 1/2” dia., 1 ft length, black	Calvana	B08MVVYHZV
**K**	Depth locknut	Same as **J**	Same as **J**	Same as **J**
**L**	Depth-adjusting handle	Same as **J**	Same as **J**	Same as **J**
**M**	Barrel	Aluminum tube, 3/4" OD,1/2" ID, 3ft length	McMaster-Carr	89965K377
**N**	Alignment disc	Delrin Acetal Resin Tube, 1” OD, ¾” ID, 1 ft length	McMaster-Carr	8627K239
**O**	Adapter	Aluminum rod, 1" dia., 1 ft length	McMaster-Carr	8974K13

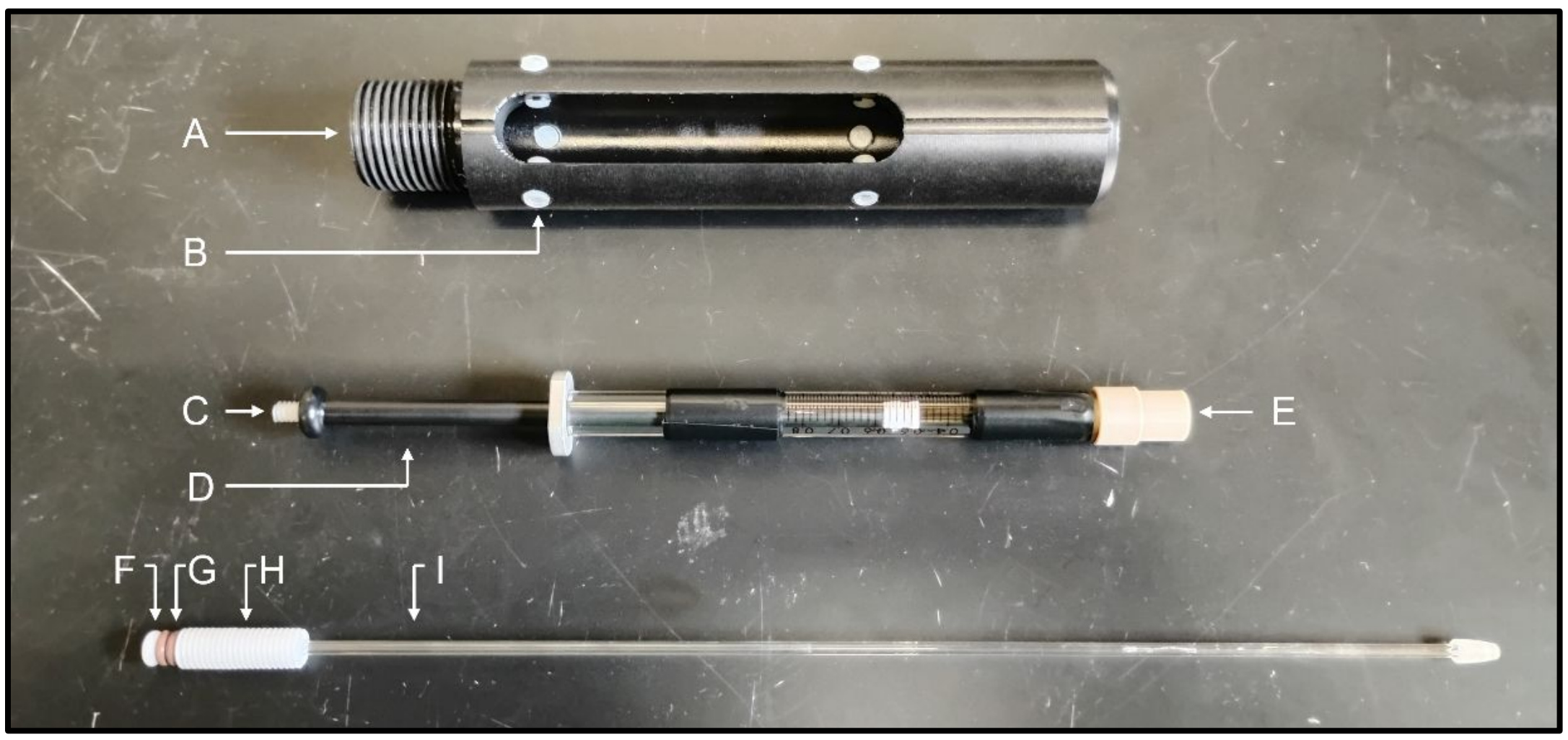

Figure S4. Overview of syringe housing and capillary adapter.

Table S2. List of components and sources for syringe housing and capillary adapter.

Label	Description	Supplier	Part Number
**A**	PEEK rod, 1" dia., 2 ft length	McMaster-Carr	8974K24
**B**	Nylon Plastic Flat-Tip Set Screws, 10-32 Thread, 5/16" Long (6x)	McMaster-Carr	94564A156
**C**	Nylon Plastic Flat-Tip Set Screws, 6-32 Thread, 1/2" Long	McMaster-Carr	94564A027
**D**	1 mL Plunger Assembly, PEEK	Hamilton	6612275-01-TMU (Custom ordered)
**E**	Gastight 1/4-28 UNF Glass Syringes	Hamilton	HM-10103662
**F**	Ultra-Chemical-Resistant Rigid PTFE O-Ring, #004	McMaster-Carr	9559K111
**G**	Chemical-Resistant Viton Fluoroelastomer O-Ring, Brown, #004 (2x)	McMaster-Carr	5267T13
**H**	Teflon rod, 3/8" dia., 1 ft length	McMaster-Carr	8546K12
**I**	Glass capillary, 1/16" OD	Chemglass	N/A

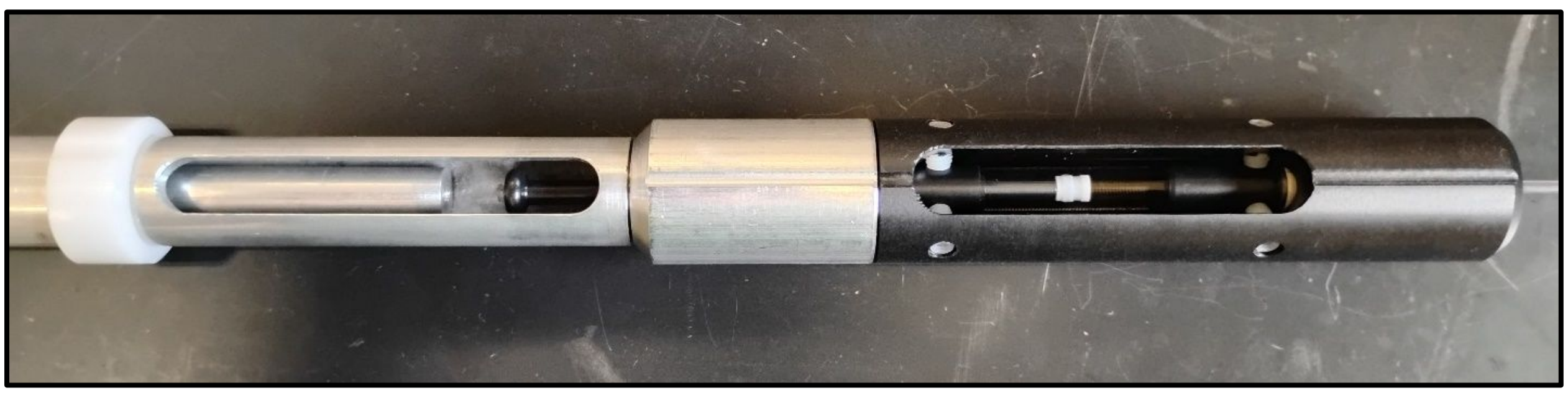

Figure S5. Close-up view of assembled syringe housing.

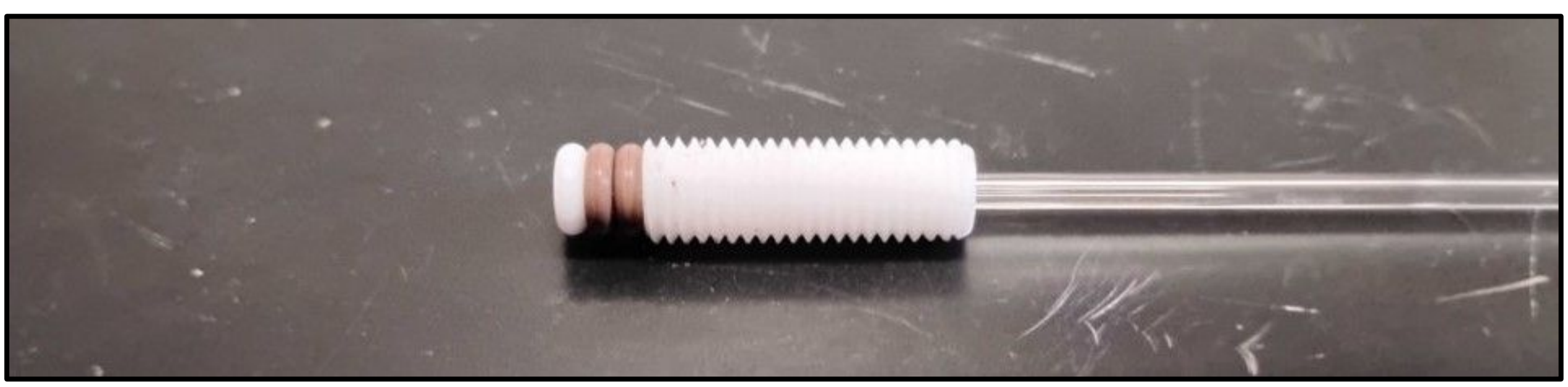

Figure S6. Close-up view of glass capillary adapter.

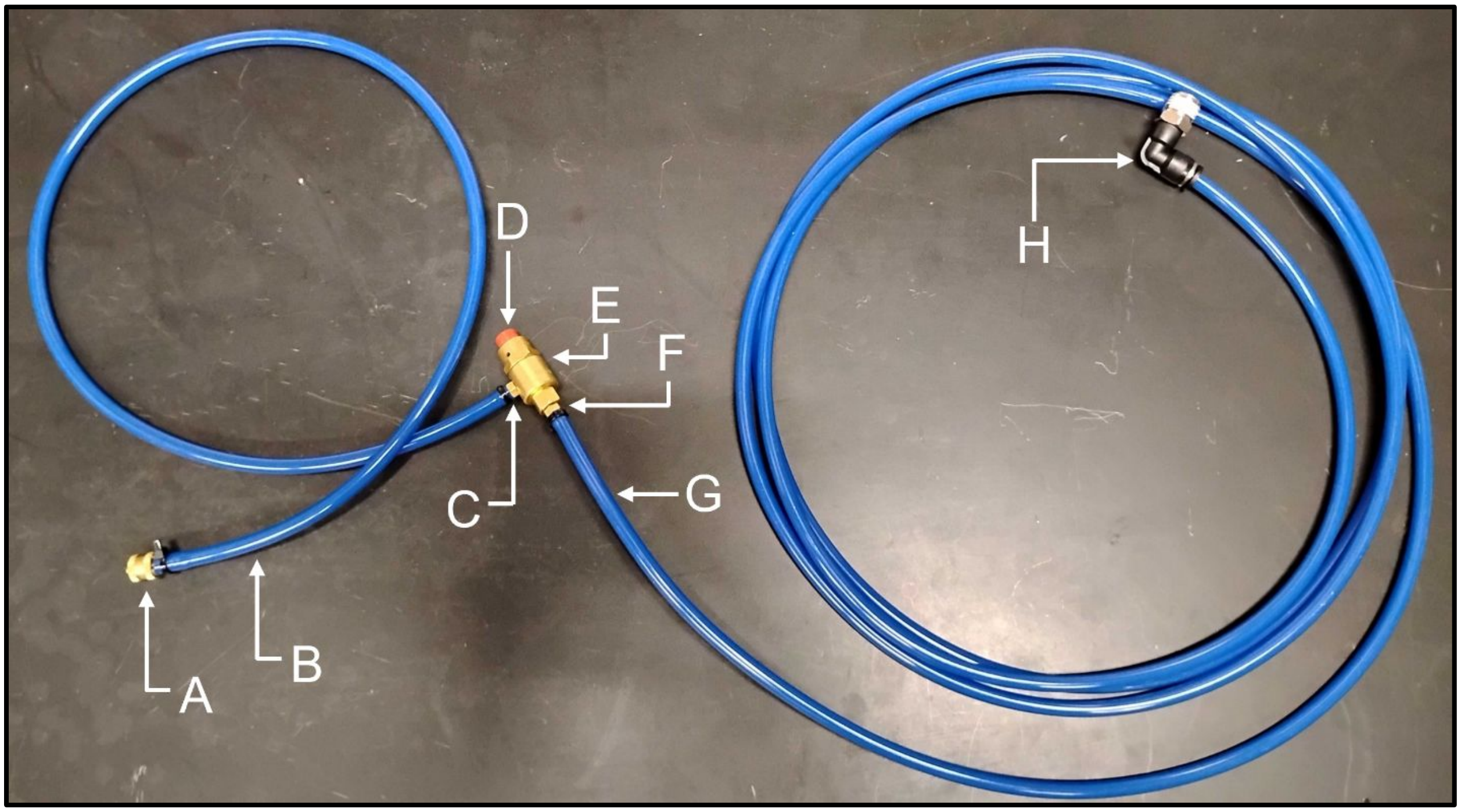

Figure S7. Overview of trigger mechanism.

Table S3. List of components and sources for trigger mechanism.

Label	Description	Supplier	Part Number
**A**	Quick Connect Cap, 1/8" Hose	Clippard	MQC-F
**B**	Firm Polyurethane Tubing, 1/8" ID, 1/4" OD, 18" length	McMaster-Carr	5648K31
**C**	#10-32 to 1/8" ID Single Barb Hose Fitting	Clippard	11750-2
**D**	3/16" captivated push button	Clippard	M-PC-2R
**E**	2-Way Stem Valve, Normally-Closed, #10-32	Clippard	MAV-2
**F**	Same as **C**	Same as **C**	Same as **C**
**G**	Firm Polyurethane tubing, 1/8" ID, 1/4" OD, 11.5 ft length	McMaster-Carr	5648K31
**H**	Push to Connect Fitting, Male Elbow 1/4" Tube OD x 1/4" NPT	MECCANIXITY	mea250506ee000202

2.3 Schematics of RI-NMR Components

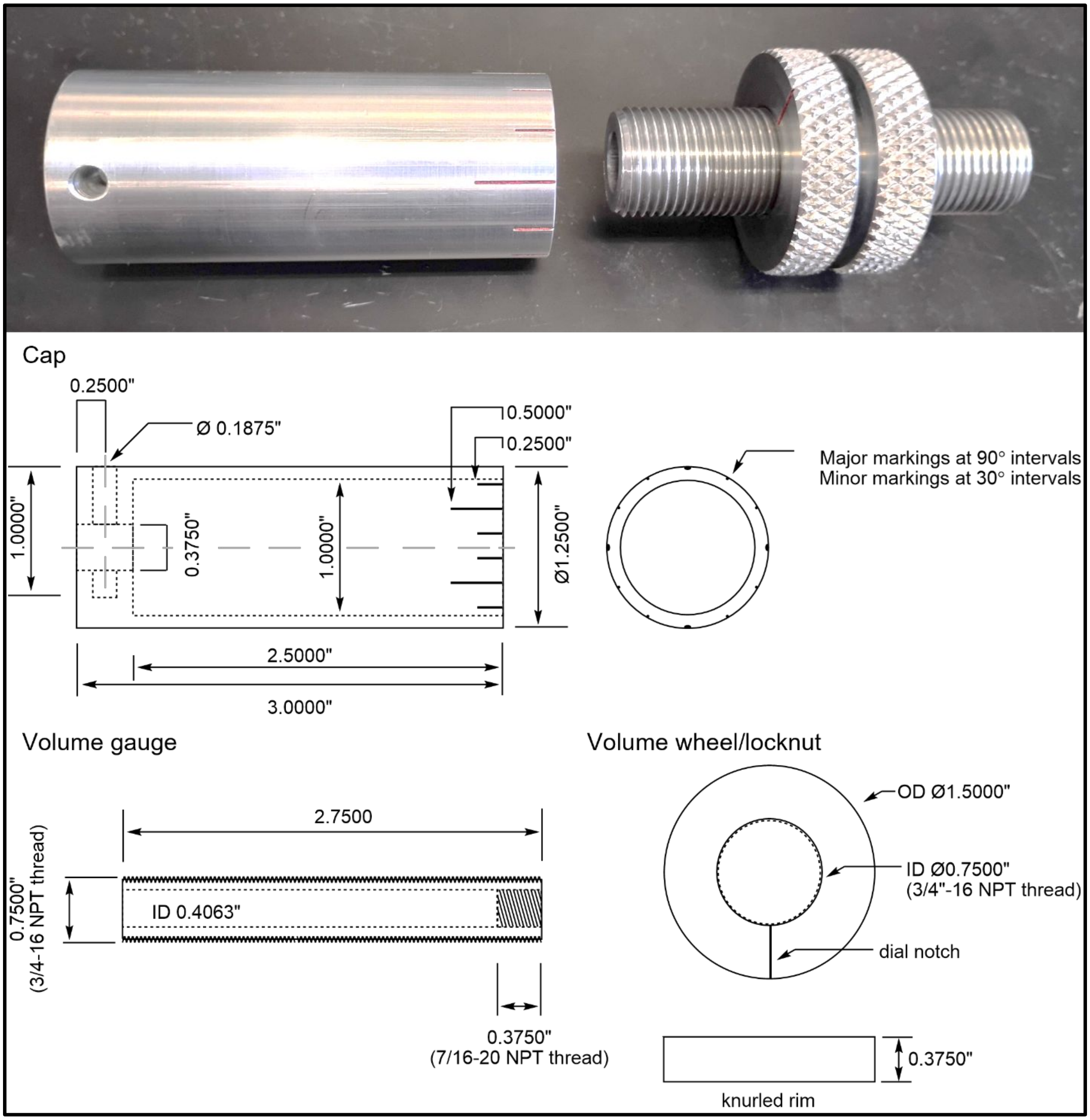

Figure S8. Schematics of cap, volume gauge, volume wheel and locknut.

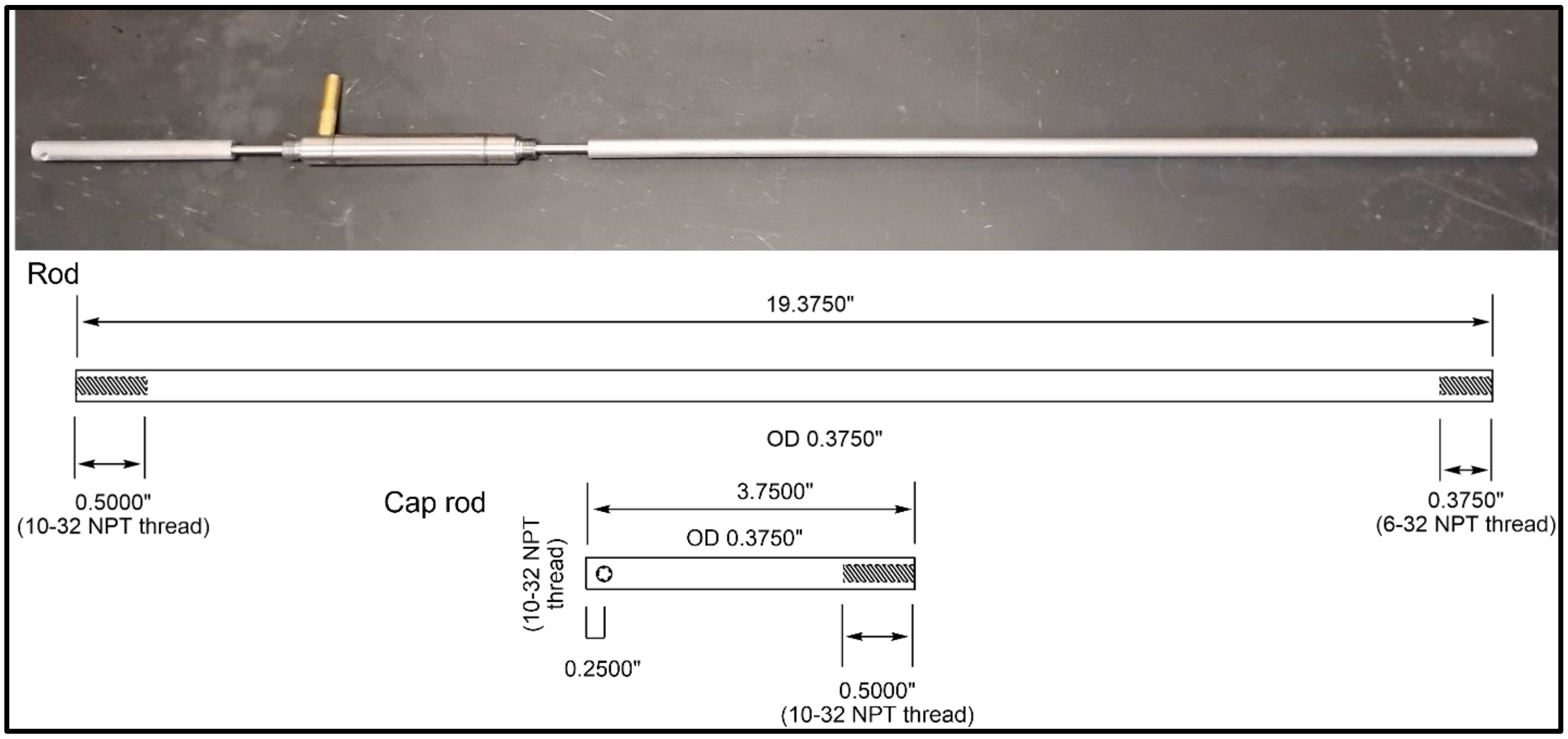

Figure S9. Schematics of rod and cap rod, connected to the piston.

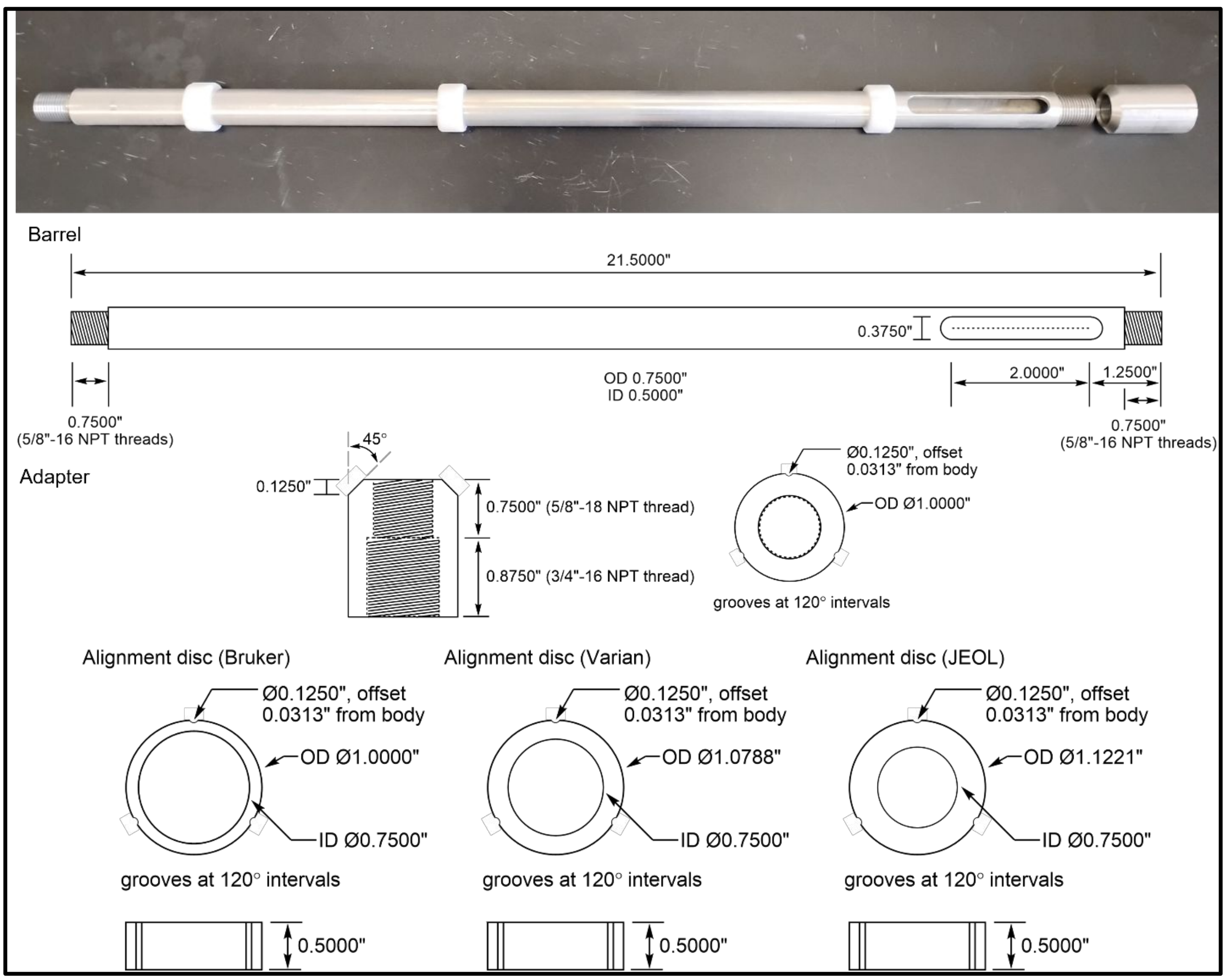

Figure S10. Schematics of barrel, adapter and alignment discs for Bruker, Varian, and JEOL spectrometers.

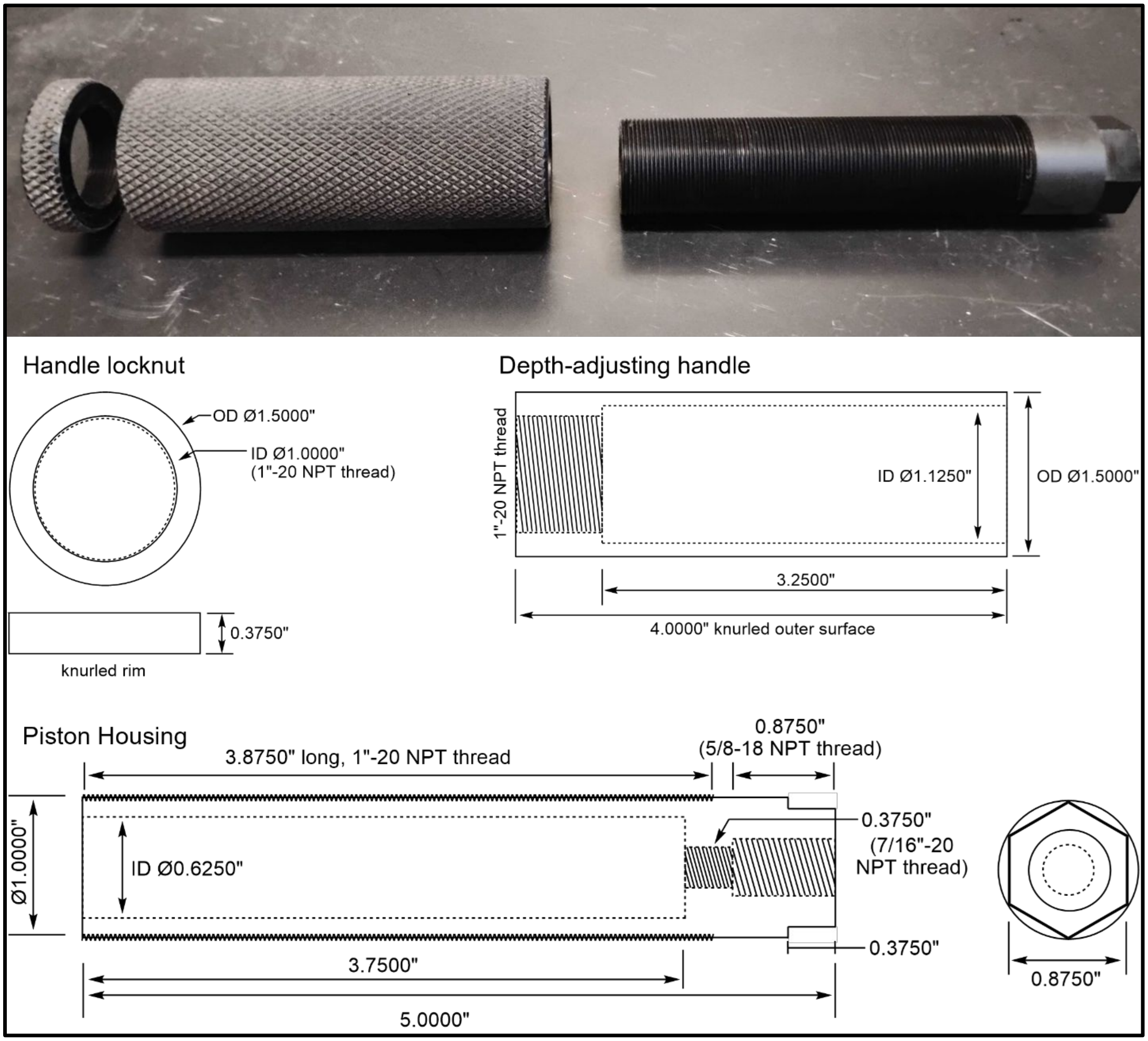

Figure S11. Schematics of handle locknut, handle, and piston housing.

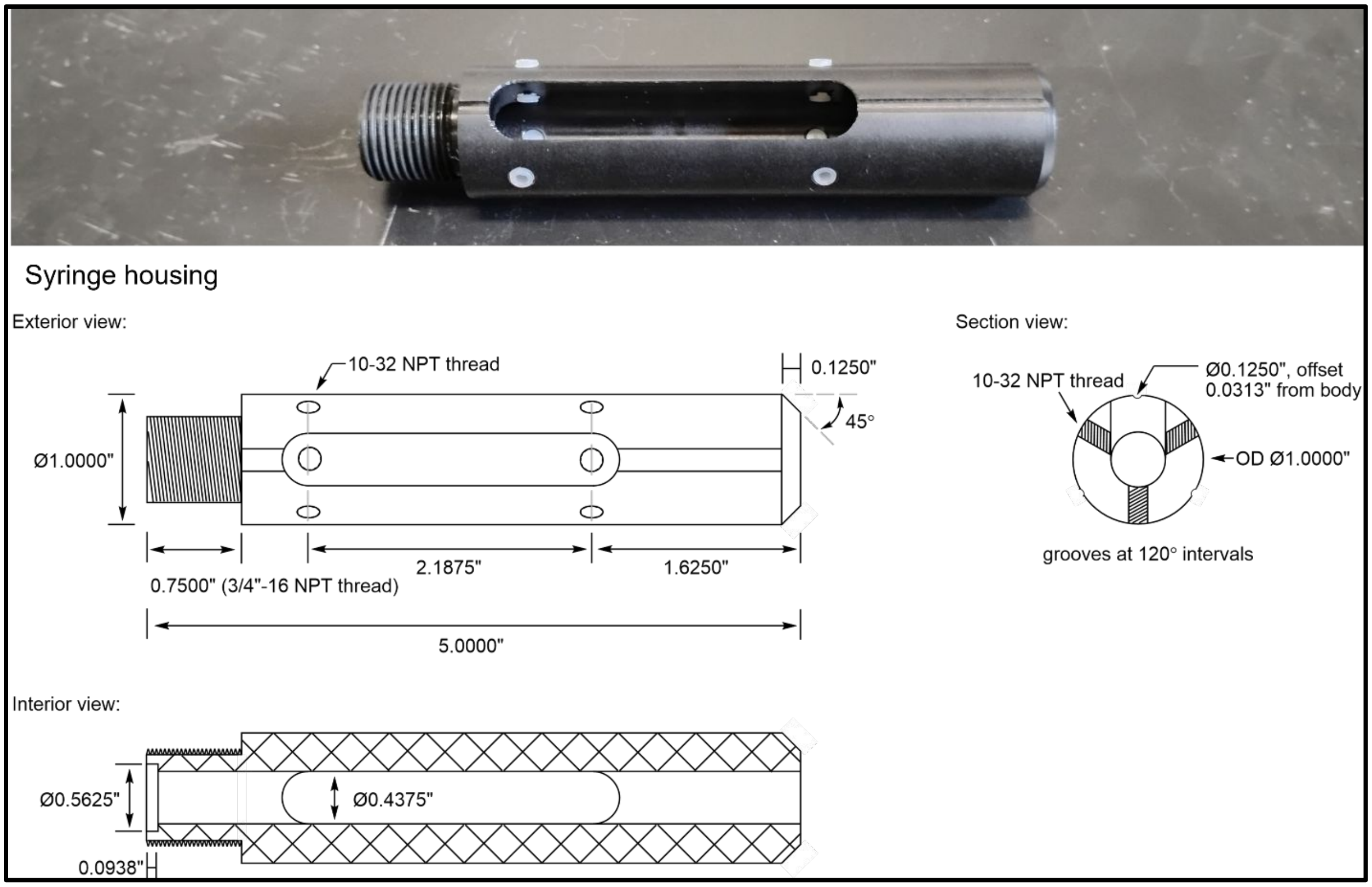

Figure S12. Schematics of syringe housing.

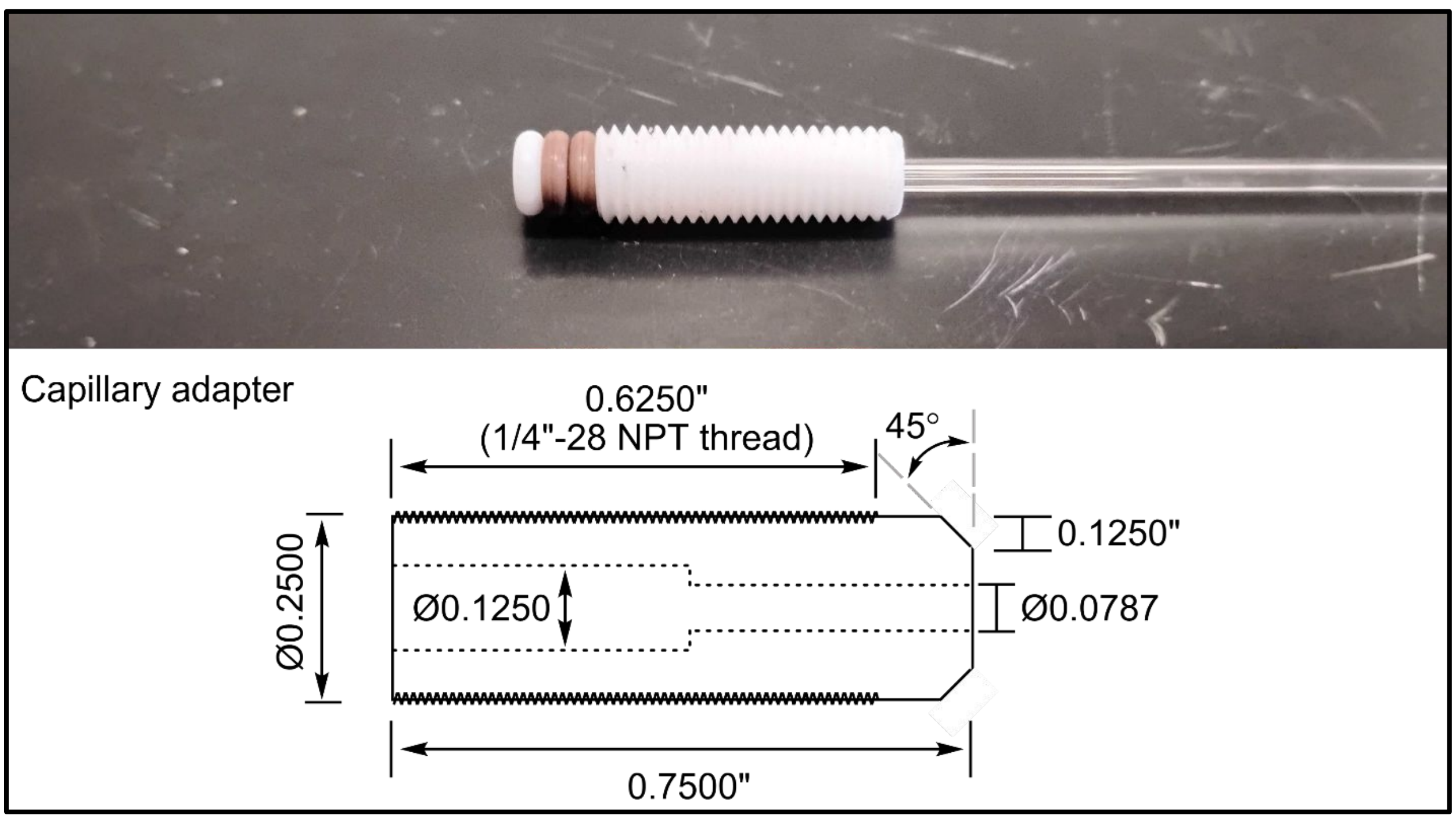

Figure S13. Schematics of capillary adapter.

3. Using the RI-NMR

3.1. Description of the RI-NMR Apparatus

The RI-NMR consists of a hand-held aluminum insert lowered into the NMR upper barrel so that the handle rests on the top of the spectrometer, which positions a non-magnetic 1 mL Hamilton syringe just above a standard 5 mm NMR tube within the spectrometer probe, from which a glass capillary extends to the bottom of the NMR tube. Accurate injection volumes between 20 – 300 μL can be set by turning the volume set screw to the appropriate distance from the piston cap, and the injection is performed by firing a pneumatic trigger connected to house air or nitrogen, which lowers the syringe piston and forces the solution inside the syringe into the NMR tube. The capillary dead volume is 200 μL, meaning that only small amounts of precious substrates or deuterated materials are lost per experiment. After injection, efficient sample mixing is achieved by manually agitating the solution with the capillary, then the apparatus is raised out of the spectrometer upper barrel. The syringe assembly can be quickly exchanged by simply detaching the syringe housing from the RI barrel and attaching a second housing, containing a syringe loaded with a second solution, enabling sequential injections of different reagent solutions.

The position of the RI-NMR inside the spectrometer is shown in **Figure S14**. Here, it is suspended from the ceiling by a pulley hanging directly above the spectrometer upper barrel for visual clarity, although the pulley is not necessary for normal operation. Instead, it is held by the experimenter who lowers, injects, and raises the RI-NMR by hand.

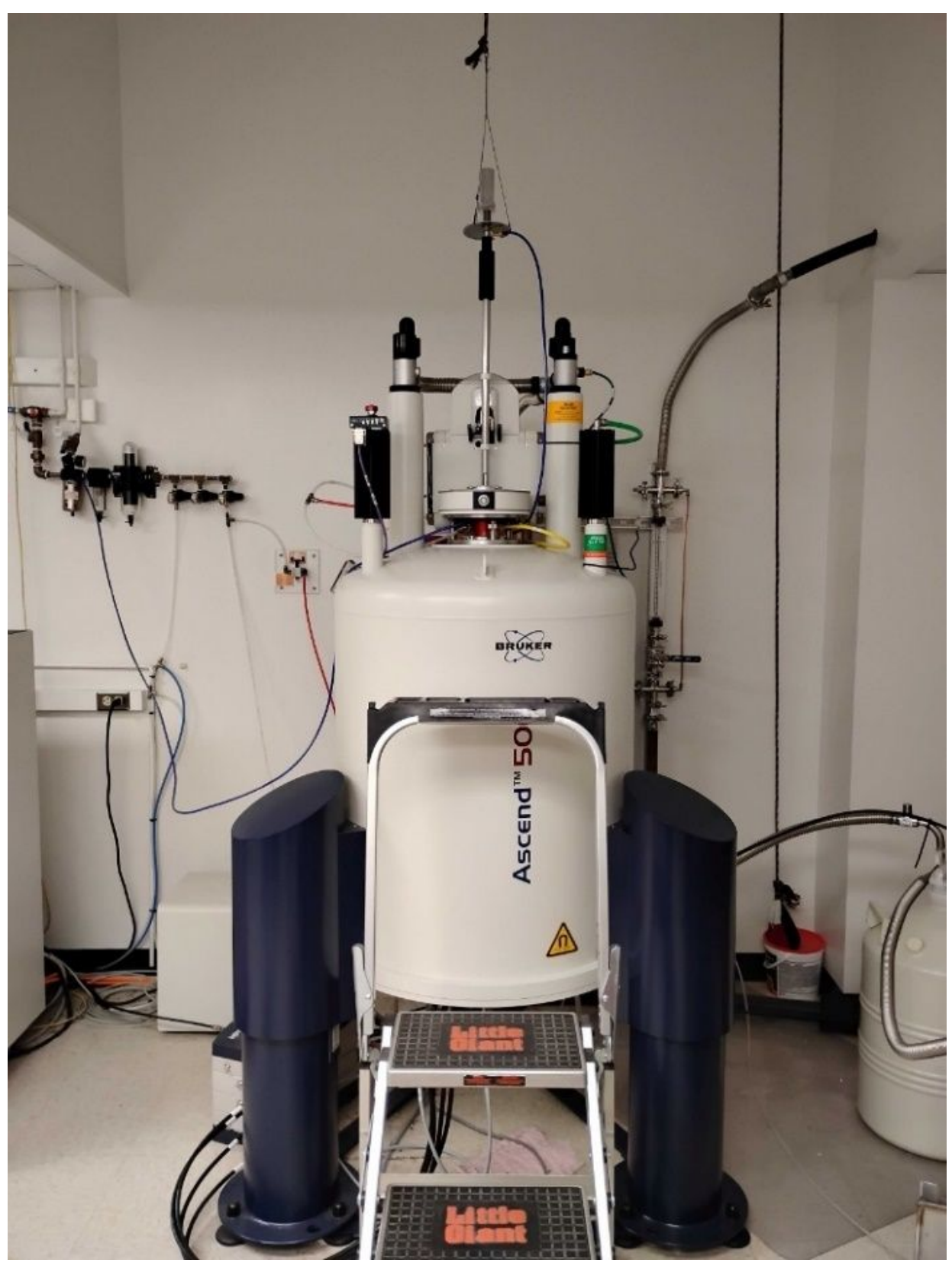

Figure S14. The RI-NMR apparatus positioned within the spectrometer upper barrel.

3.2. Preparing the RI-NMR for Use

To prevent damage to the NMR probe, it is essential that the RI-NMR be calibrated to sit properly inside the spectrometer before use. This involves two steps: (1) alignment of the capillary to prevent contact with the walls of the NMR tube; and (2) calibration of the depth-adjusting handle to seat the capillary tip at the appropriate depth.

3.2.1. Capillary Alignment

To align the capillary, the assembled RI-NMR is placed on a stand, and an alignment block is placed at so that the two prongs sit around the capillary tip (**Figure S15**). The RI apparatus is then rotated while resting in the stand. Inspect the position of the capillary tip while rotating: If the position of the tip moves closer to one side of the notch, then the capillary is not aligned properly. Adjust the six screws which set the position of the syringe. Continue to rotate the RI apparatus to check the alignment. ***When the capillary tip does not move during rotation, the capillary is aligned.***

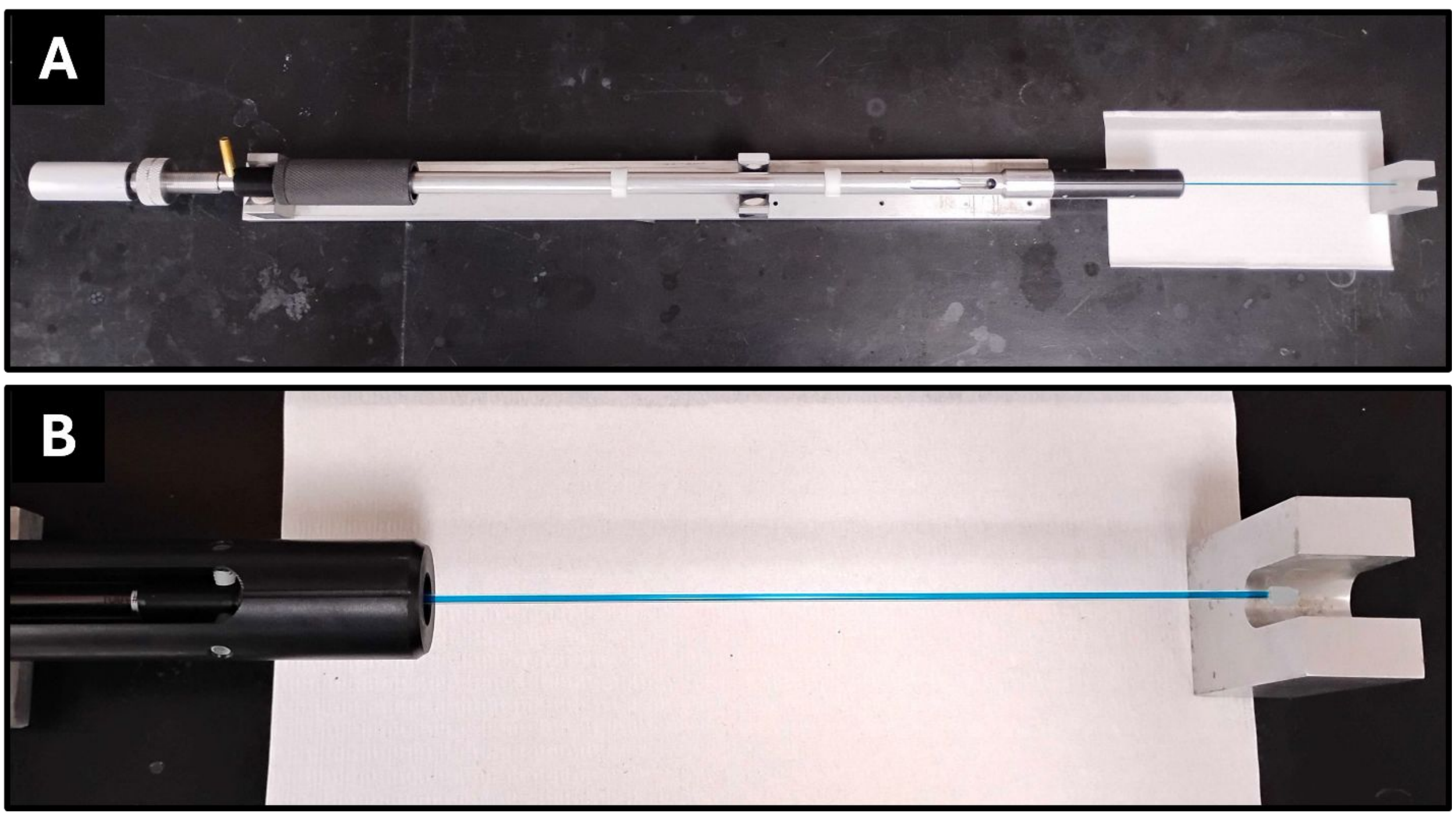

Figure S15. Capillary alignment. **A**. The RI apparatus is rotated on the aluminum stand. **B**. Check the tip of the capillary – when it is does not move during rotation, the capillary is aligned.

3.2.2. Setting and Monitoring the Capillary Depth

To set the depth of the capillary tip, a spectrometer upper barrel can be used to visually inspect the depth of the capillary tip before inserting the apparatus into the NMR itself (**Figure S16A**). The depth of the capillary can be set by rotating the depth-adjusting handle so that the tip of the capillary sits just above the bottom of the NMR tube (**Figure S16B**). This ensures that the entire sample volume can be mixed after injection. This procedure can also be conducted by carefully lowering the apparatus into the spectrometer itself and until the capillary tip gently touches the bottom of the NMR tube, then setting the depth-adjusting handle so the capillary tip sits just above this position. Once the depth is set, it does not need to be adjusted if capillaries of identical length are used.

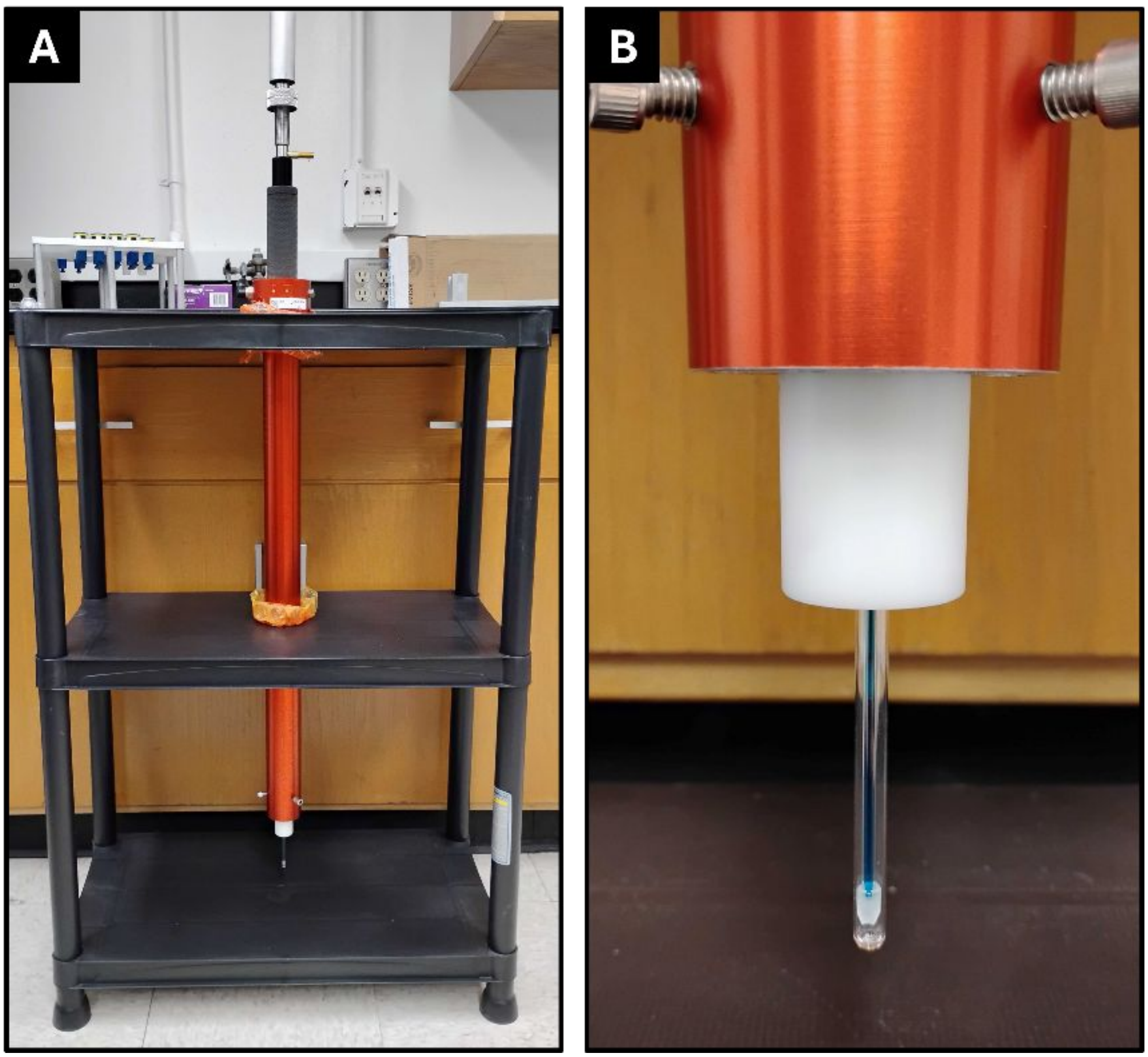

Figure S16. Depth adjustment using a spectrometer upper barrel. The tip of the capillary should be positioned just above the bottom of the NMR tube.

The Teflon alignment discs can be used as depth gauges to track the position of the capillary tip relative to the NMR tube. Before an experiment, align an NMR tube parallel to the barrel and flush with the bottom of the handle (**Figure S17A**). Position the upper disc so that it is seated roughly to the height of the solution inside the NMR tube, and the lower disc just above the end of the

NMR tube. This makes it easy to judge when the capillary is inside the NMR tube and when it is inserted into the reaction solution (**Figure S17B-D**).

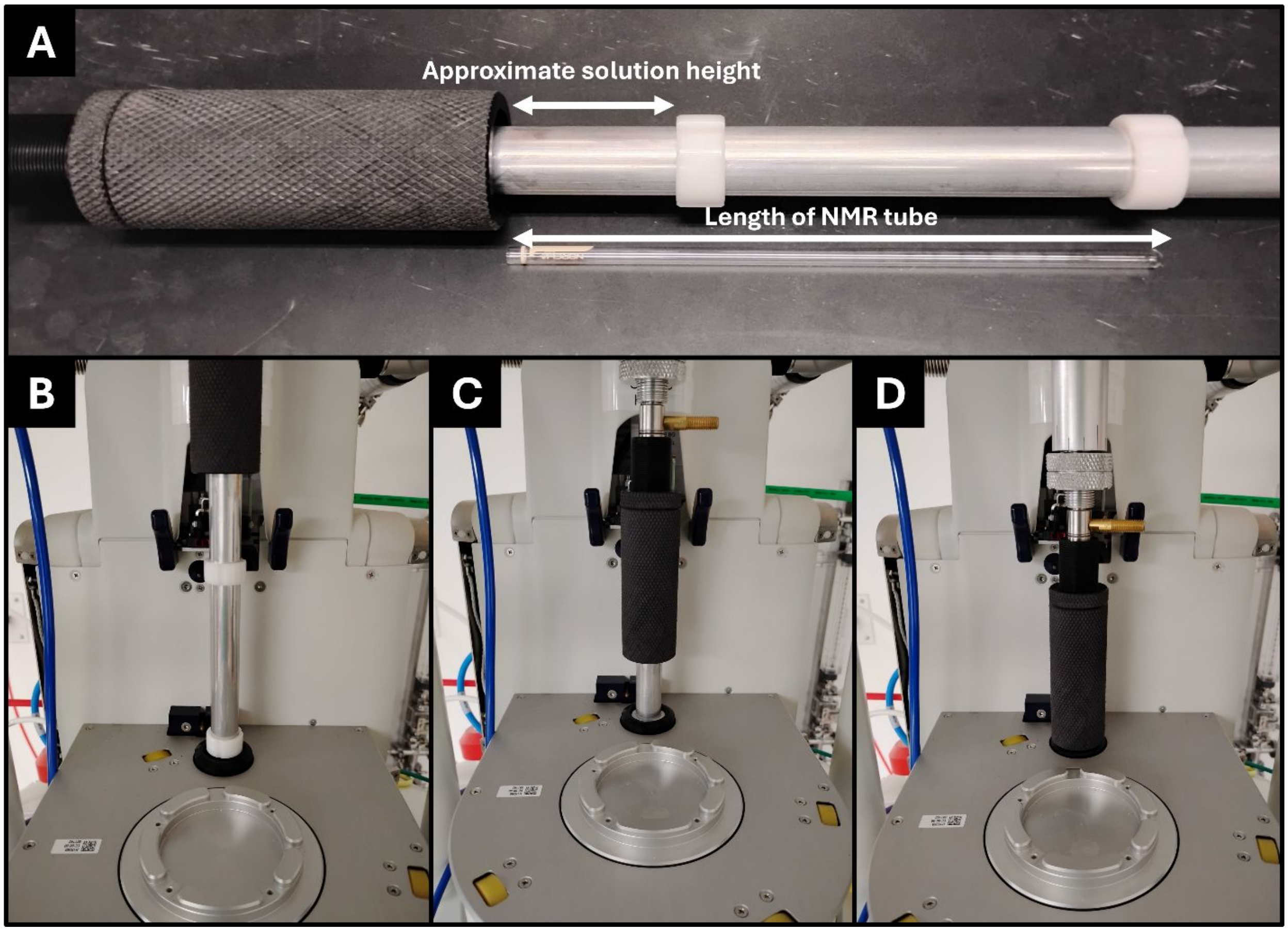

Figure S17. Alignment discs as capillary depth gauges. **A.** Set the discs as shown. **B.** When the lower disc is just above the upper barrel hole, the capillary tip is sitting just above the NMR tube. **C.** When the upper disc is flush with the hole, the capillary tip is sitting just above the solution. Hold in this position for 30 sec to ensure premagnetization of the reagent solution. **D.** When the RI apparatus is fully lowered, the capillary tip is sitting just above the bottom of the NMR tube. Inject from this position or that shown in **C**. Move between **C** and **D** to mix the solution.

3.2.3. Loading the Syringe Assembly

If the experiment will involve sequential injections of multiple solutions, the capillary of each syringe assembly (syringe barrel + syringe housing + capillary) should be aligned before use, and capillaries of uniform length should be used so that the depth-adjusting handle does not need to be readjusted during the course of an experiment. The syringe assembly ***without the syringe plunger*** is cleaned with acetone and water and dried in a low-temperature oven (70 °C) for at least 30 mins before use if the reaction is moisture-sensitive or if it will be brought into the glovebox (**Figure S18A**). ***The syringe plunger will be destroyed if it is placed in the oven.***

Solutions for NMR studies are be prepared using low-temperature oven-dried volumetric flasks. After loading the solution into the syringe assembly, hold the syringe with the capillary tip upright and draw any solution from the capillary into the syringe barrel, then push out any air until the

solution sits at the capillary tip. Attach the assembly to the body of the RI apparatus and screw the plunger into the piston rod (**Figure S18B**). If the solution inside the syringe is air- or moisture-sensitive, the capillary can be capped with a septum (Sigma SKU Z554014).

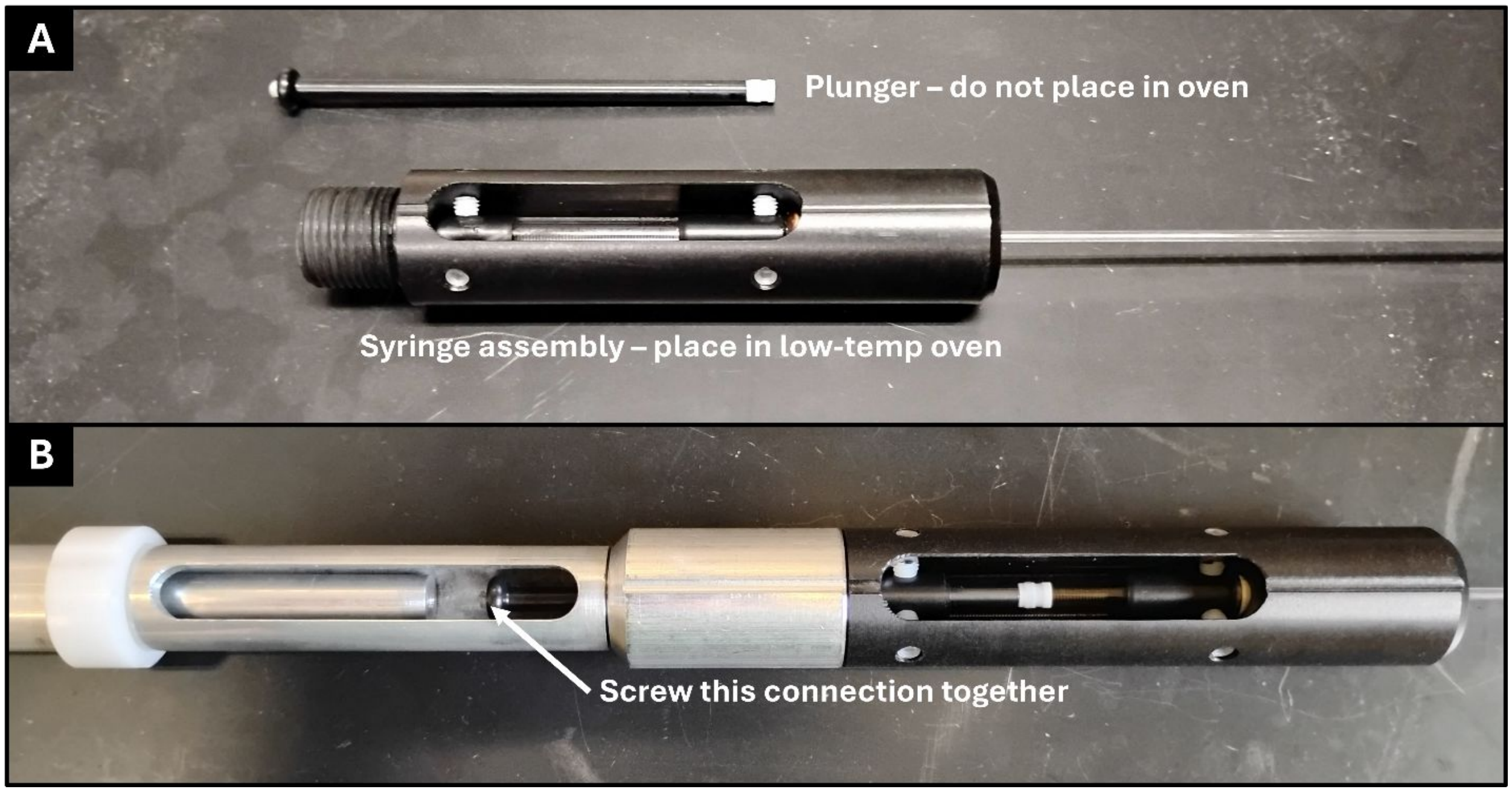

Figure S18. Syringe assembly and plunger. **A.** Syringe assembly. **B.** Connections between syringe assembly, RI barrel and rod.

3.2.4. Setting the Injection Volume

Just before beginning the data acquisition sequence, push any remaining air out of the capillary tip. Then, set the desired injection volume using the volume dial at the top of the RI apparatus (**Figure S19**). The piston cap is marked with twelve marks like a clock face, and the volume set screw has a single marking. Each revolution and partial revolution of the screw can be easily tracked, and each mark corresponds to about 3 μL.

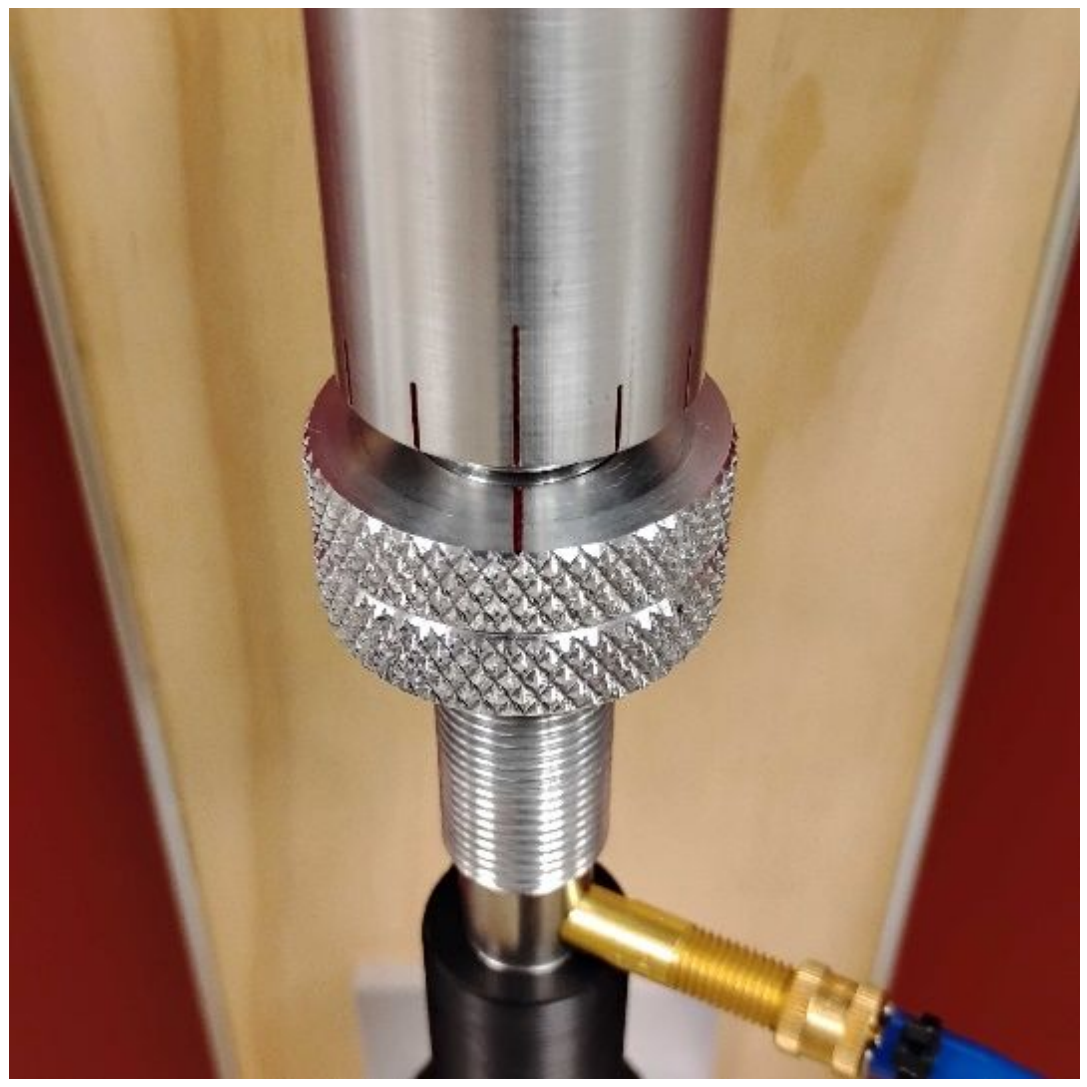

Figure S19. The volume set screw and piston cap are used together to determine injection volume.

3.3. Performing an RI-NMR kinetics experiment

Lower an uncapped NMR tube containing the reaction sample into the spectrometer probe. To protect air-sensitive samples, the spectrometer bore and spinning can be switched from air to nitrogen like a typical low-temperature VT-experiment. This makes a N_2 atmosphere above the tube and prevents exposure to air. As an additional precaution against exposure to air, the septum cap is removed and the top of the NMR tube is protected with a gloved finger until the sample is lowered into the bore.

With the RI apparatus in hand, begin the data acquisition sequence. Insert the RI apparatus into the NMR upper barrel and down into the NMR tube. Hold the capillary above the solution for several seconds to ensure premagnetization of the reagent solution (see Supporting Information Section 6), then simultaneously press the pneumatic trigger button to perform the injection and lower the apparatus fully to mix the sample, then remove the RI from the spectrometer. To prevent cross-contamination between samples, eject ~50 μL of solution into a waste container and wipe the exterior of the capillary before the next experiment. The RI-NMR experiment is now running.

4. Capillary Tip Design and Calibration

4.1. Calibration Procedure

All calibrations were performed by injection into an NMR tube inside the spectrometer at 25 °C using the indicated capillary. To determine the injection volume, various amounts of a 0.1 M solution of 1,3,5-trimethoxybenznene in THF was injected into NMR tubes containing 500 µL of a 0.1 M solution of 1,2,4,5-tetramethylbenzene in $CDCl_3$ inside the spectrometer, manually mixed, and analyzed by quantitative ^{1}H NMR to determine the integral ratio of injection solution versus standard. Injection volumes were selected as revolutions of the volume wheel.

4.2. Simple Capillary (A)

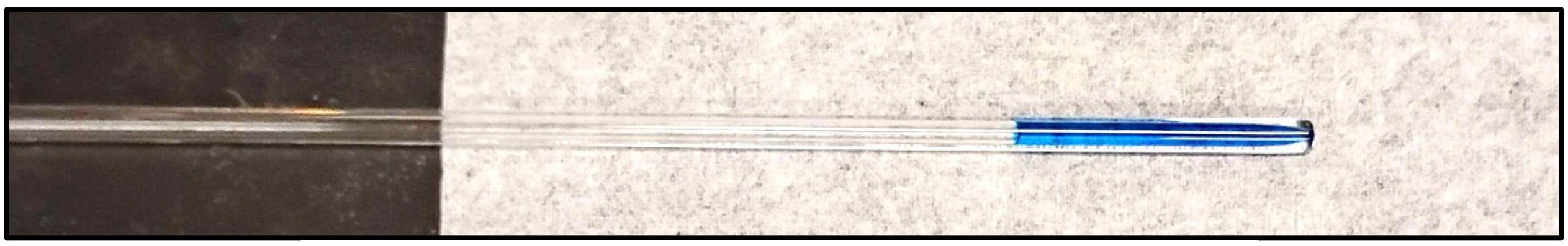

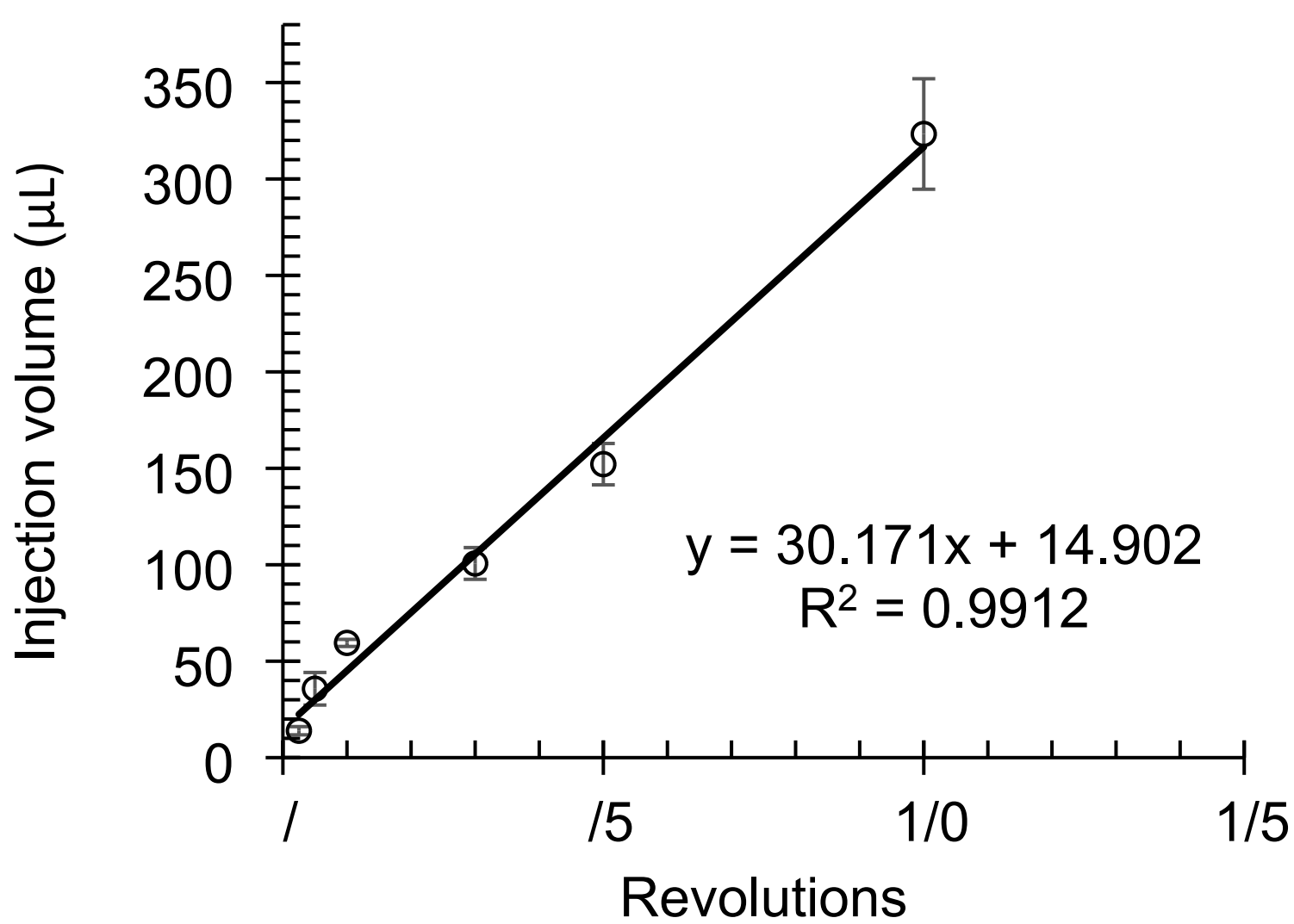

Figure S20. Design and calibration curve of the simple capillary.

Table S4. Calibration data for injections with the simple capillary.

Revolutions	Injected volume (µL)
3/12	14.0 ± 2.1
6/12	35.7 ± 8.4
1	59.5 ± 1.8
3	100.7 ± 8.2
5	152.2 ± 10.7
10	323.3 ± 28.6

4.3. Ringed Capillary (B)

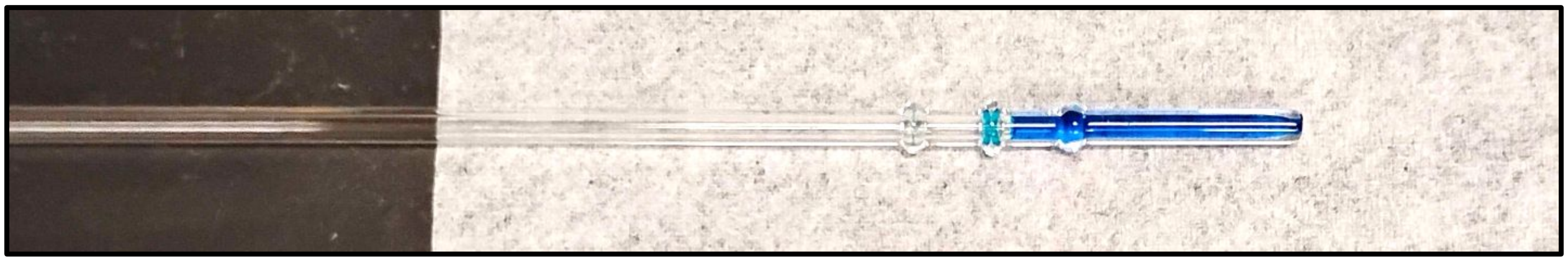

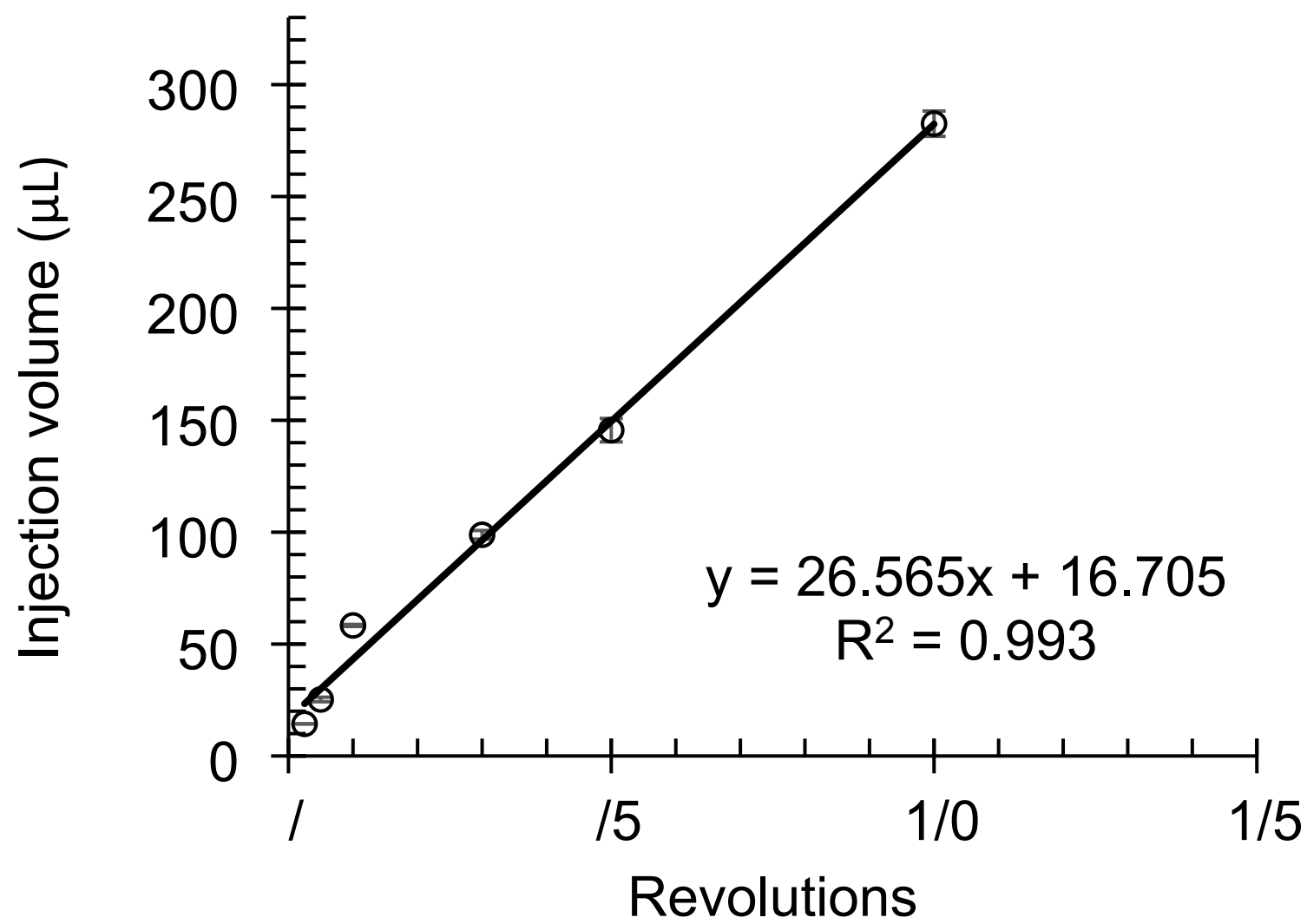

Figure S21. Design and calibration curve of the ringed capillary.

Table S5. Calibration data for injections with the ringed capillary.

**Revolutions**	**Injected volume (µL)**
3/12	14.3 ± 2.6
6/12	25.2 ± 3.9
1	58.3 ± 4.1
3	98.8 ± 2.9
5	145.6 ±1.1
10	282.5 ± 3.3

4.4. Bulbed Capillary (C)

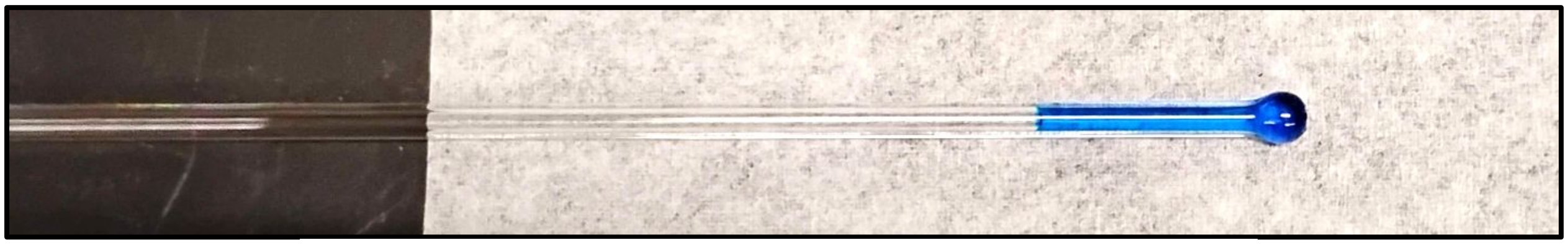

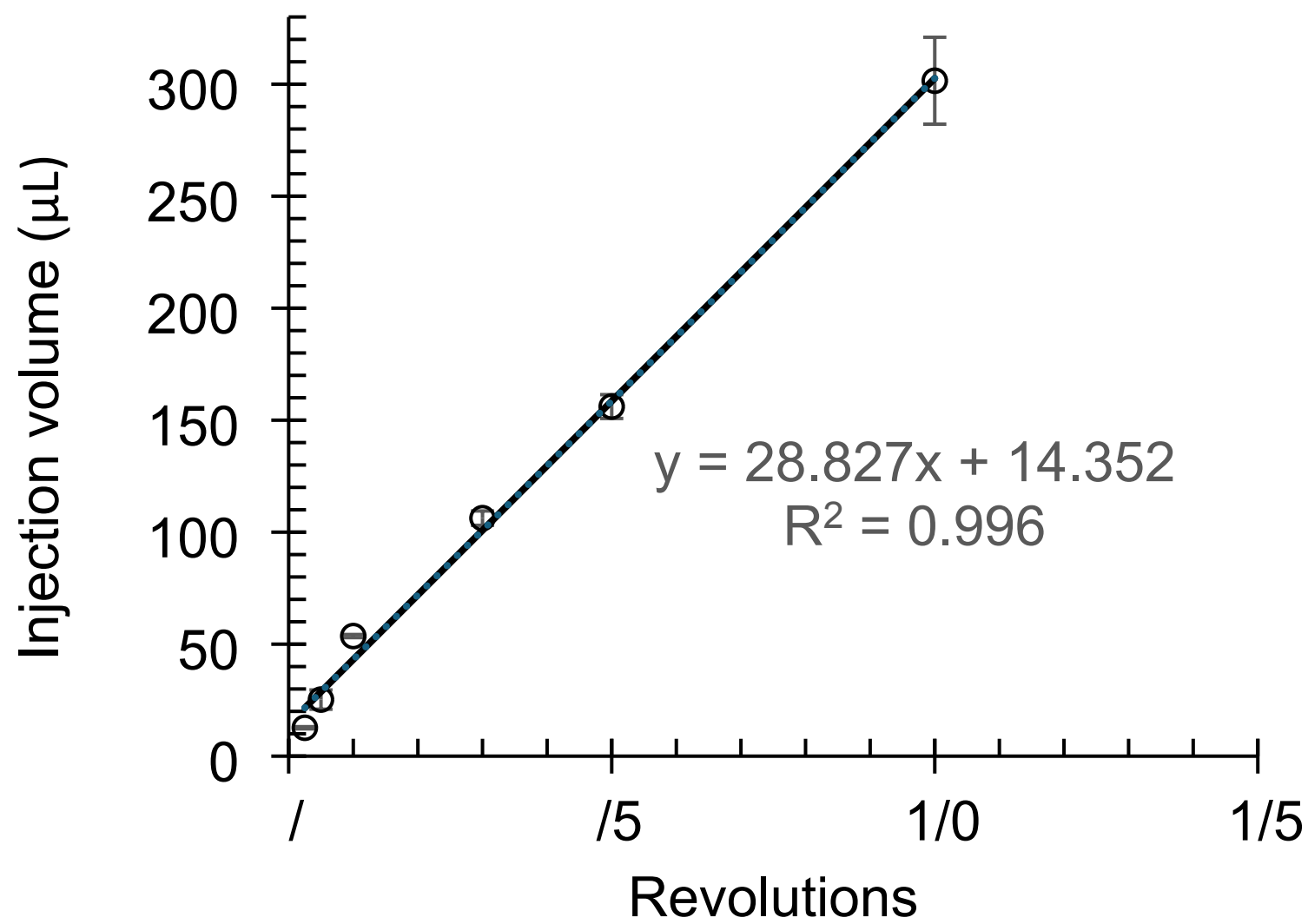

Figure S22. Design and calibration curve of the bulbed capillary.

Table S6. Calibration data for injections with the bulbed capillary.

Revolutions	Injected volume (µL)
3/12	12.7 ± 0.5
6/12	25.3 ± 4.3
1	53.6 ± 0.7
3	106.3 ± 3.2
5	156.1 ± 5.3
10	301.5 ± 19.4

4.5. Arrowhead Capillary (D)

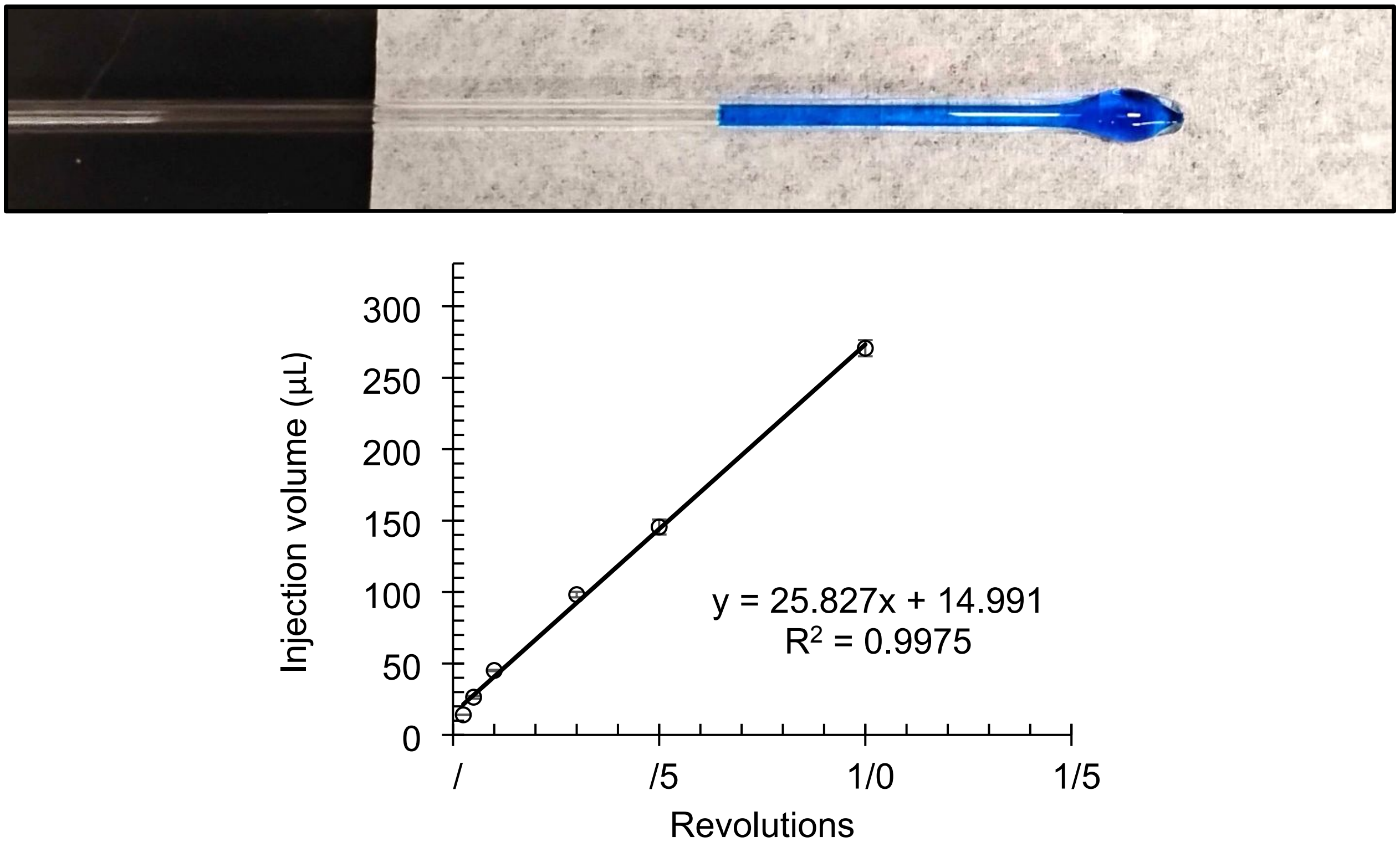

Figure S23. Design and calibration curve of arrowhead capillary.

Table S7. Calibration data for injections with the arrowhead capillary.

Revolutions	Injected volume (μL)
3/12	14.1 ± 0.03
6/12	26.4 ± 1.0
1	45.1 ± 0.5
3	98.2 ± 1.9
5	145.5 ± 5.2
10	270.7 ± 5.7

4.6. Collared Capillary (E)

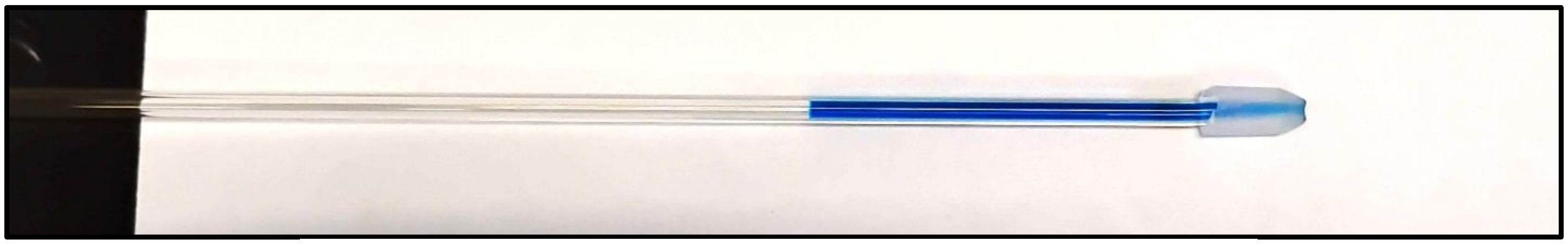

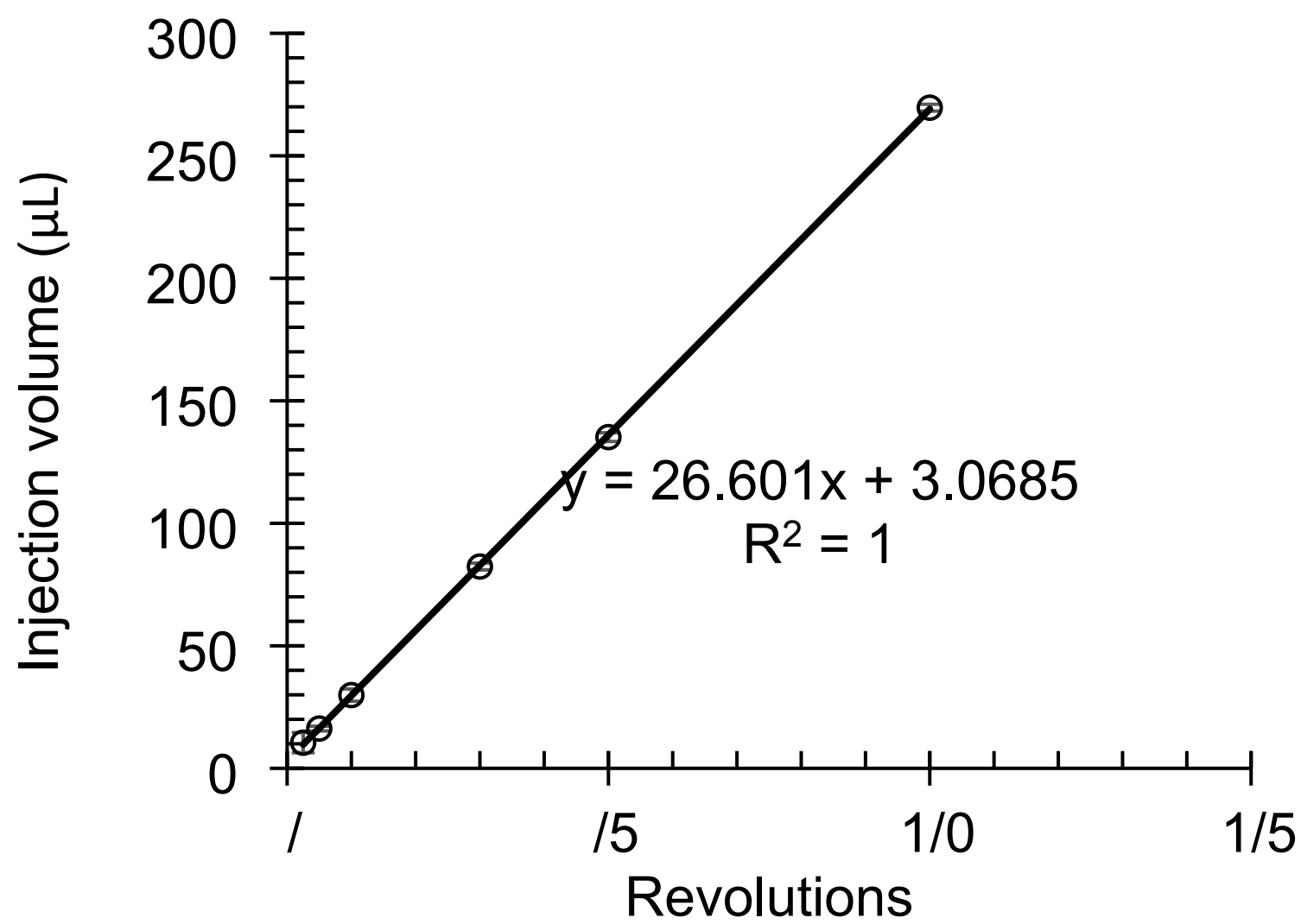

Figure S24. Design and calibration curve of collared capillary.

Table S8. Calibration data for injections with the collared capillary.

Revolutions	Injected volume (µL)
3/12	10.5 ± 4.1
6/12	16.2 ± 1.0
1	29.9 ± 2.5
3	82.4 ± 1.4
5	135.2 ± 1.8
10	269.6 ± 1.4

4.7. Discussion of Capillary Performance

In order to ensure the collection of reproducible kinetic data, the sample must efficiently mix immediately following injection of the reagent. While all capillary designs evaluated delivered accurate injection volumes, the ability to quickly mix the sample differed. We evaluated the mixing efficiency of each capillary by filming injections of Blue #2 dye in MeOH (45 μL) into a 5mm tube containing MeOH (500 μL) and manually agitating the solution (see Supplementary Videos **S2–S5**). We found that the simple 2 mm OD glass capillary (A) provided poor mixing. To address this, we affixed a series of glass rings near the bottom of the capillary (B). While this improved mixing slightly, we reasoned that the placement of mass at the tip of the capillary would provide the most efficient mixing. Both bulbed (C) and arrowhead-shaped (D) blown-glass capillary tips provided efficient mixing, but often introduced bubbles into the sample due to the large internal cavity. Collared capillary (E) was superior in terms of mixing efficiency and volume delivery and was used for all further experiments.

4.8. Fabrication of Capillaries

Simple capillary (A) was prepared by melting the tip of a 9”, 2 mm OD capillary tube with a blowtorch until the tip was nearly, but not fully, sealed. The capillary was tempered in an oven (120 °C) for several hours, then brought to room temperature, and the tip was opened with a 0.5 or 0.7 mm drill bit on a lathe.

Ringed capillary (B) was prepared by heating small sections of capillary (A) with a glassblowing torch while spinning on a double-chuck lathe, then pushing the tube together to extrude a ring of glass at three positions roughly 1/8” apart.

Bulbed capillary (C) was prepared by melting the tip of a 9”, 2mm OD capillary tube with a blowtorch until the tip was nearly, but not fully, sealed, then blowing through the tube to extrude a bulb with a diameter of approximately 3 mm.

Arrowhead capillary (D) was prepared from capillary (A) by heating the glass approximately 1/4” from the tip, and blowing through the tube to extrude a bulb of the desired shape. Because the tip is not malleable, the size of the size of the opening remains constant.

Collared capillary (E) was prepared in a three-step process (**Figure S25**). A short bead of 1/8” OD glass is fused to an 8.5”-long, 2 mm OD capillary with a blowtorch (A). The fused capillary is tempered in an oven (120 °C) for several hours (B). Finally, the capillary was opened with a 0.5 mm drill bit on a lathe and injector tip is ground on a glass grinding wheel to create a self-guiding tip. Videos showing the production of (B) and (C) are provided (Supplemental videos **S6** and **S7**) .

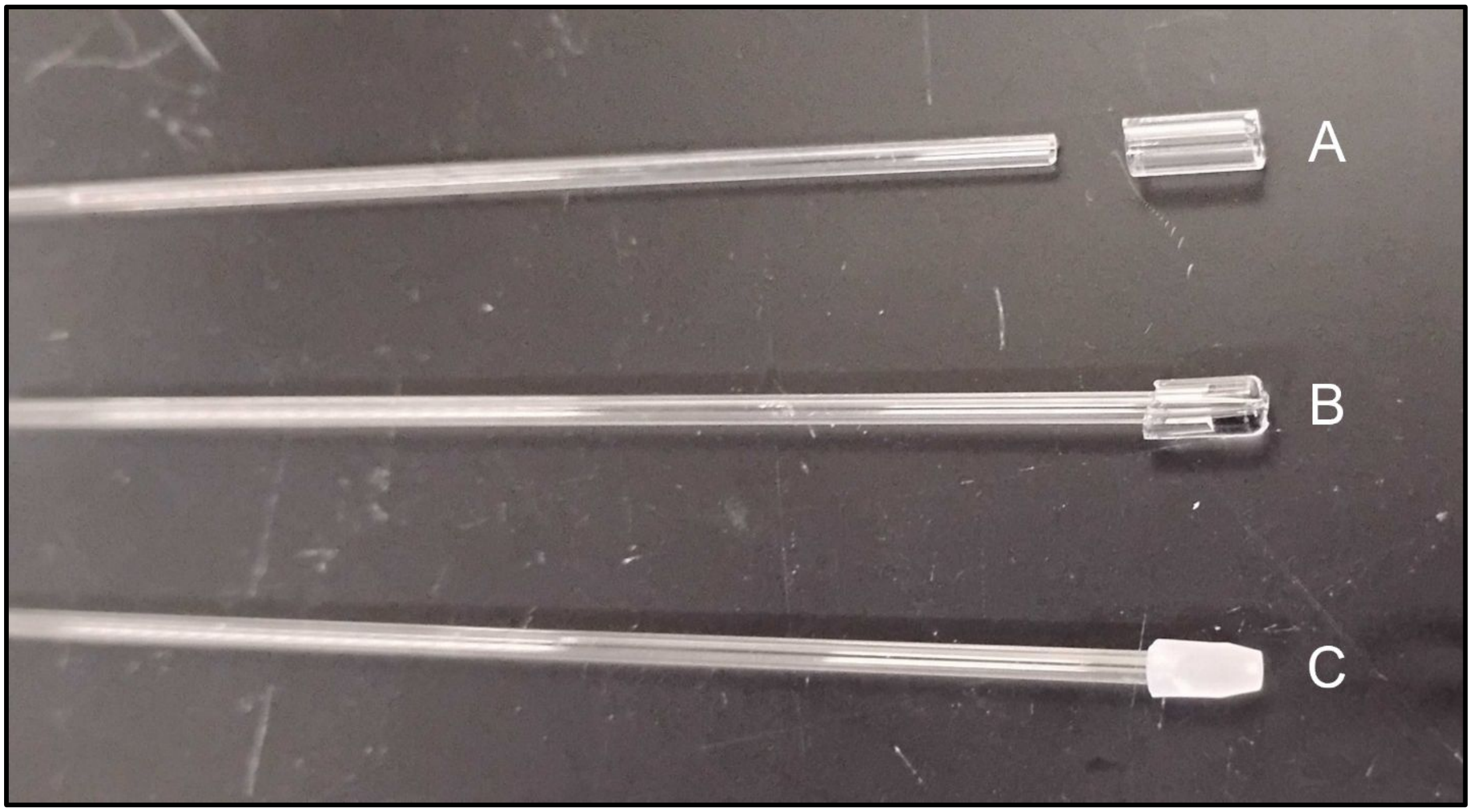

Figure S25. Fabrication of collared capillary.

5. Additional Injection Calibrations

5.1. Room-Temperature Calibration Procedure

All calibrations were performed by injecting various amounts of a 0.1 M solution of 1,4-bromofluorobenzene in the indicated solvent into NMR tubes containing 500 µL of a 0.1 M solution of 4-fluorotoluene inside the spectrometer at 25 °C, manually mixed, and analyzed by quantitative ^{19}F NMR to determine the integral ratio of injection solution versus standard. Injection volumes were selected as revolutions of the volume wheel.

5.2. Solvent = *n*-Hexane

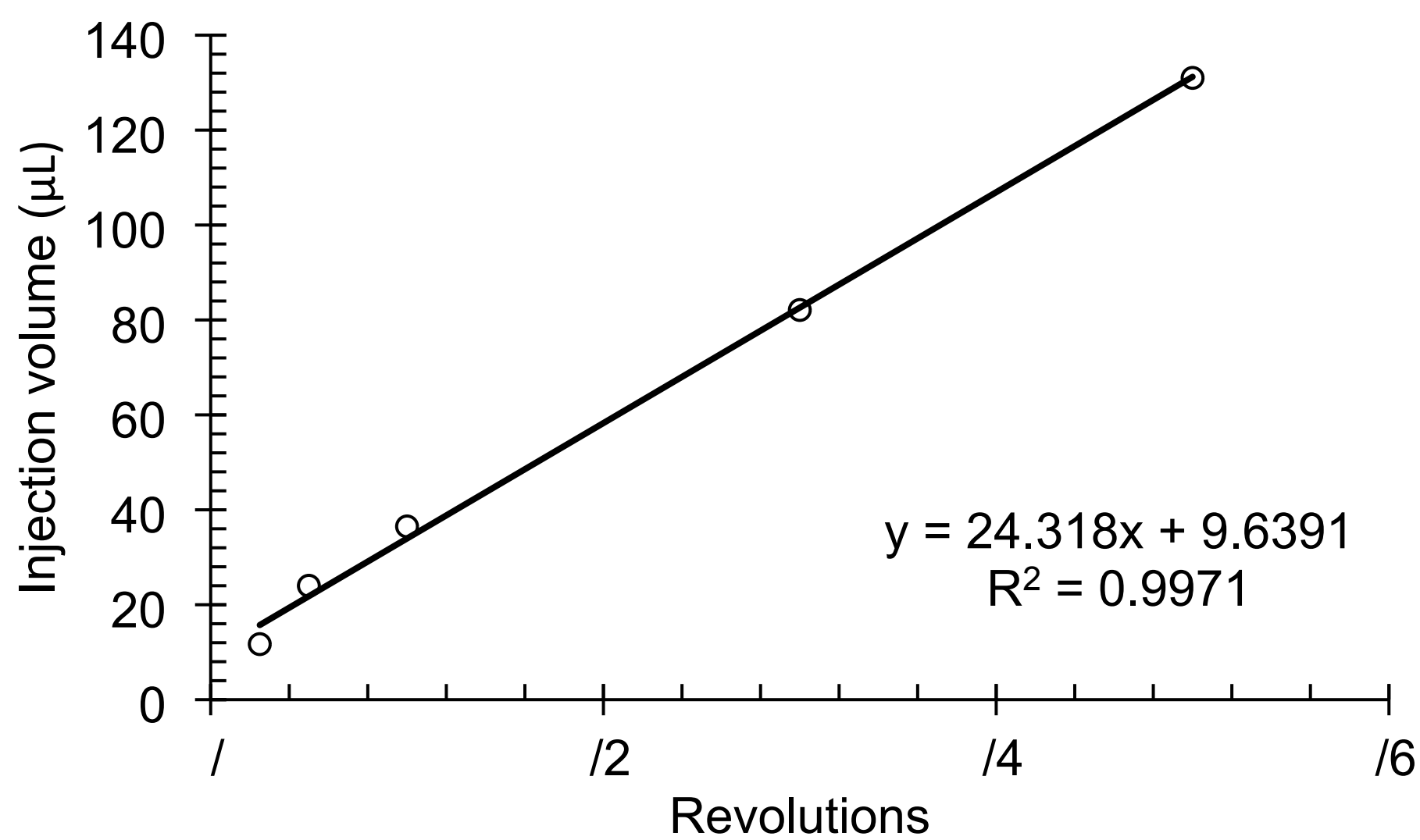

Figure S26. Calibration curve for *n*-hexane at 25 °C (viscosity: 0.31 cp).

Table S9. Calibration data for injections in *n*-hexane.

Revolutions	Injected volume (µL)
3/12	11.7
6/12	24
1	36.5
3	82.1
5	131

5.3. Solvent = Toluene

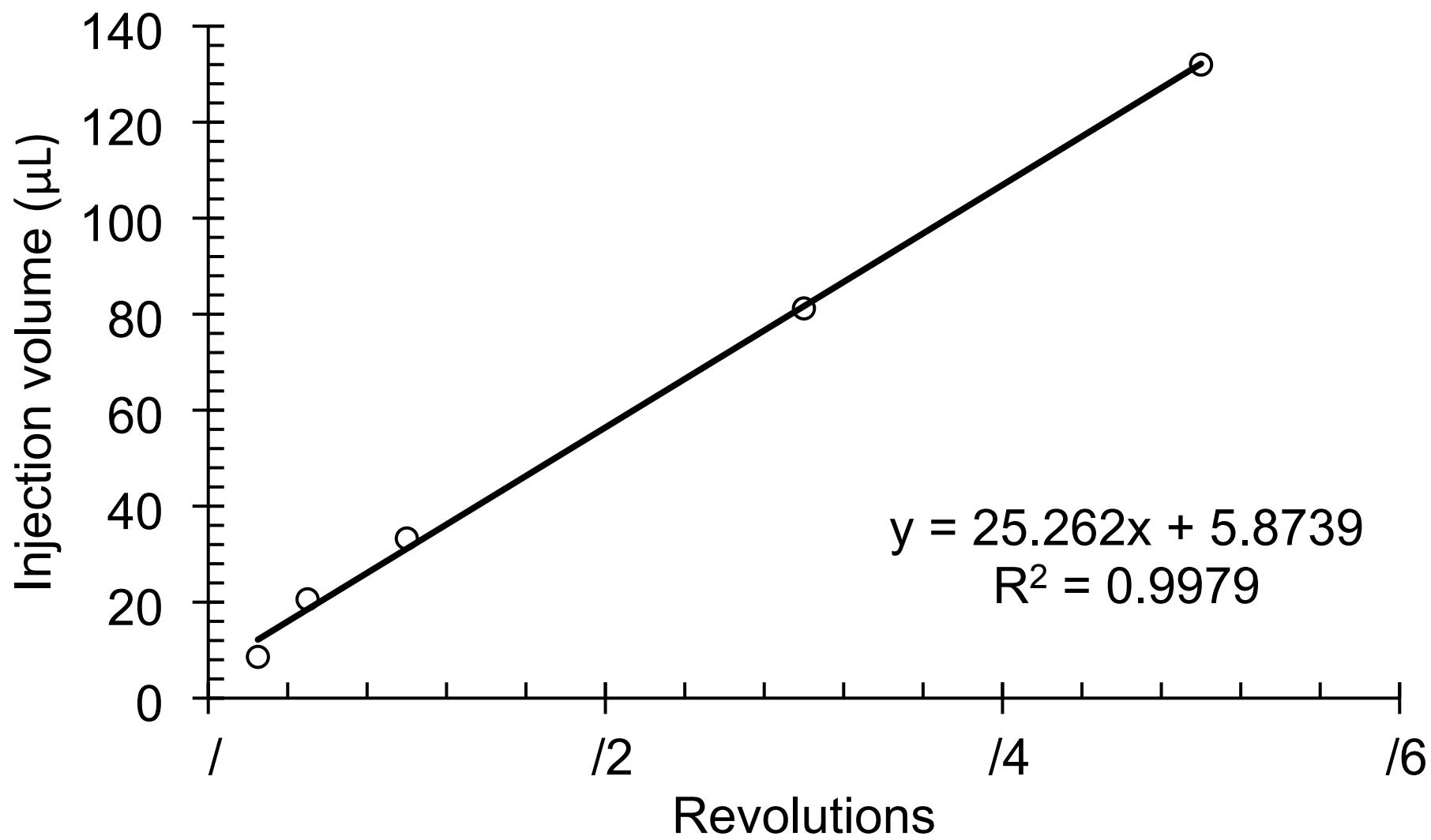

Figure S27. Calibration curve for toluene at 25 °C (viscosity: 0.59 cp).

Table S10. Calibration data for injections in toluene.

**Revolutions**	**Injected volume (µL)**
3/12	8.6
6/12	20.6
1	33.27
3	81.2
5	132.0

5.4. Solvent = DMF

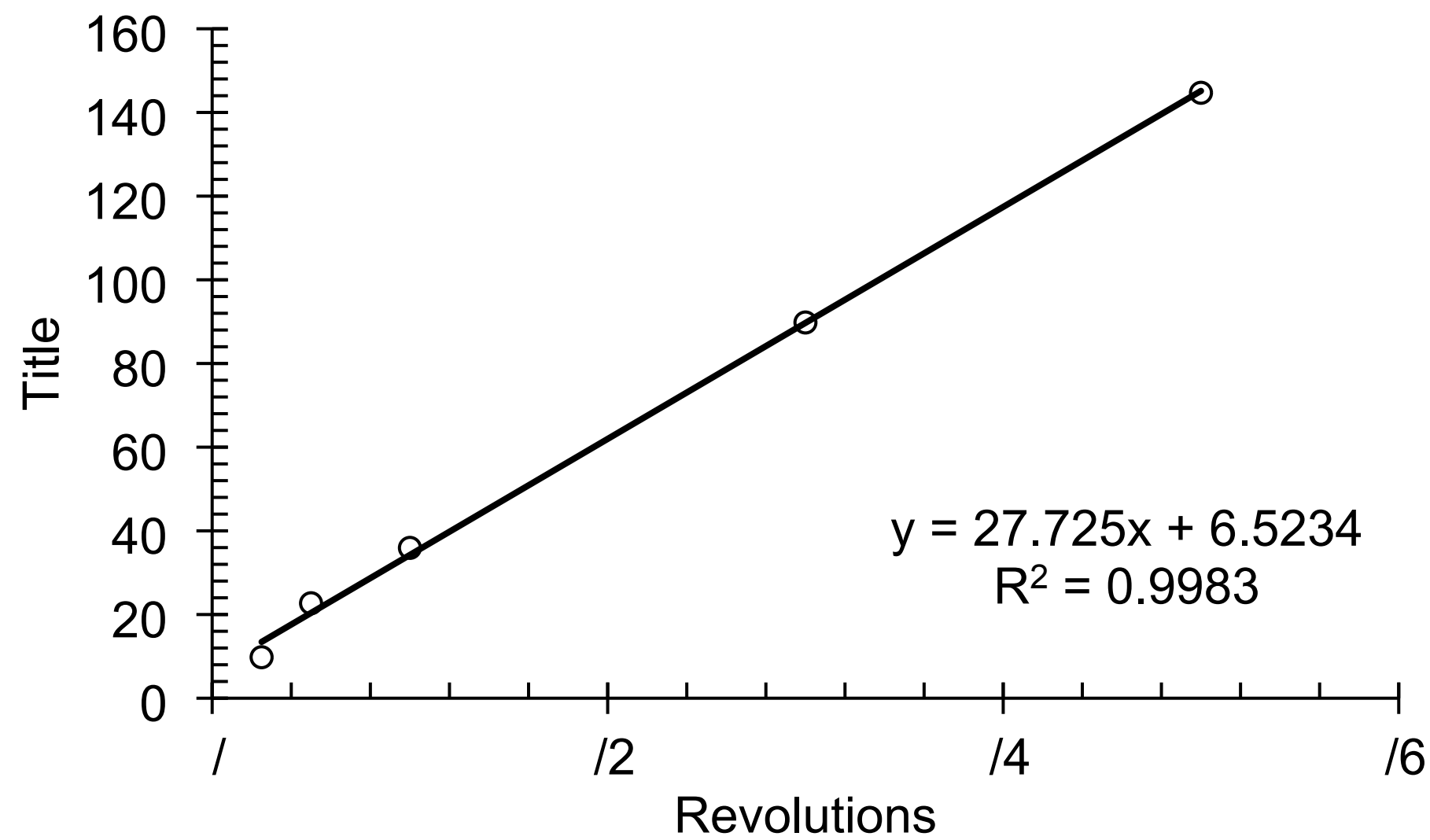

Figure S28. Calibration curve for DMF at 25 °C (viscosity: 0.92 cp).

Table S11. Calibration data for injections in DMF.

Revolutions	Injected volume (µL)
3/12	8.6
6/12	20.6
1	33.27
3	81.2
5	132.0

5.5. Solvent = DMA

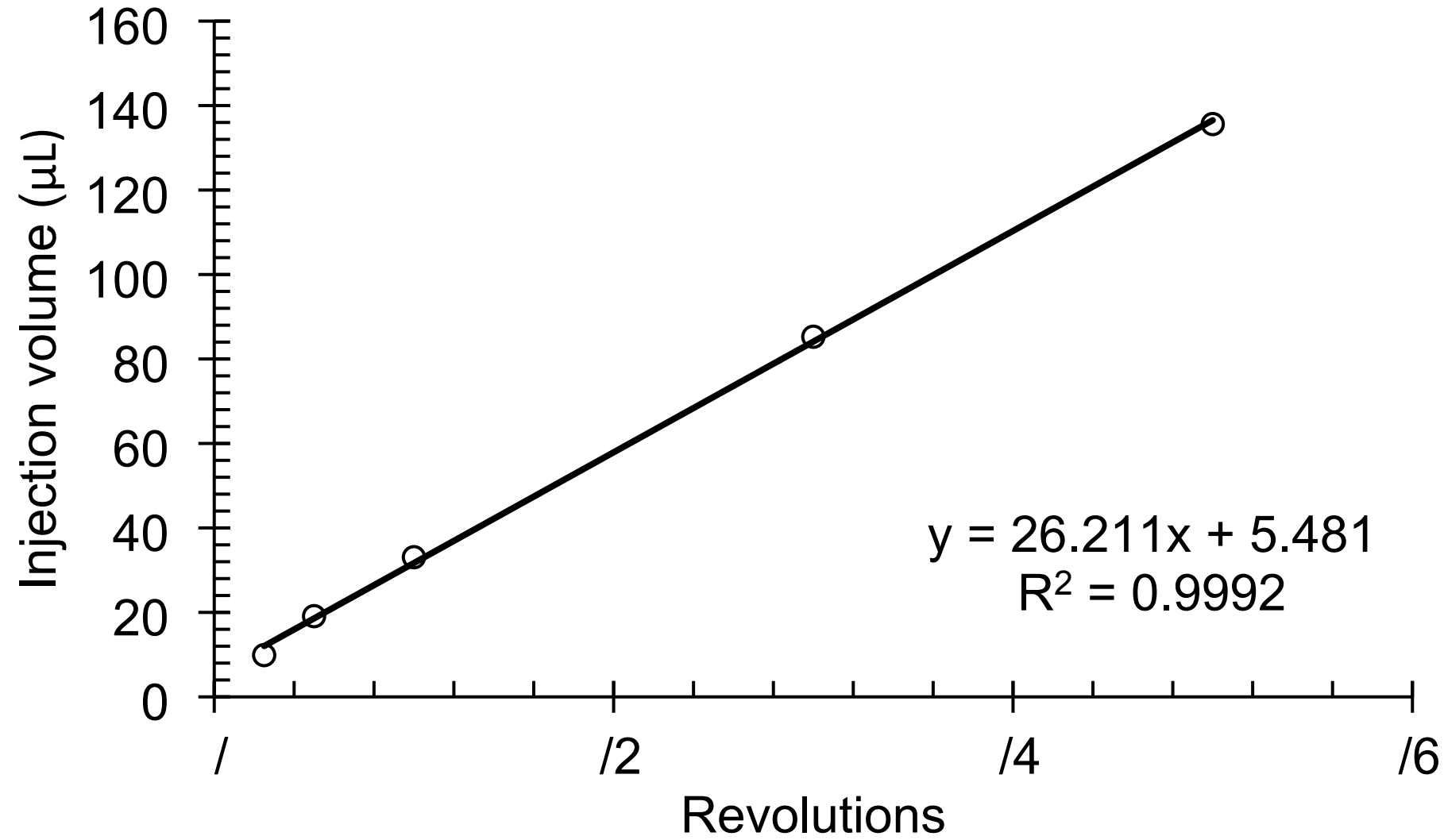

Figure S29. Calibration curve for DMA at 25 °C (viscosity: 2.14 cp).

Table S12. Calibration data for injections in DMA.

Revolutions	Injected volume (µL)
3/12	9.34
6/12	20.55
1	33.07
3	85.27
5	135.62

5.6. Solvent = THF, T = –80 °C

Calibrations were performed by injecting various amounts of a 0.1 M solution of 1,4-bromofluorobenzene in THF into NMR tubes containing 500 µL of a 0.1 M solution of 4-fluorotoluene inside the spectrometer at –80 °C using a collared capillary. The RI syringe was positioned just above the solution inside the NMR tube for 60 s prior to injection to allow the solution inside the capillary to cool, the solution was injected, the sample was manually mixed and analyzed by quantitative ^{19}F NMR to determine the integral ratio of injection solution versus standard. Injection volumes were selected as revolutions of the volume wheel.

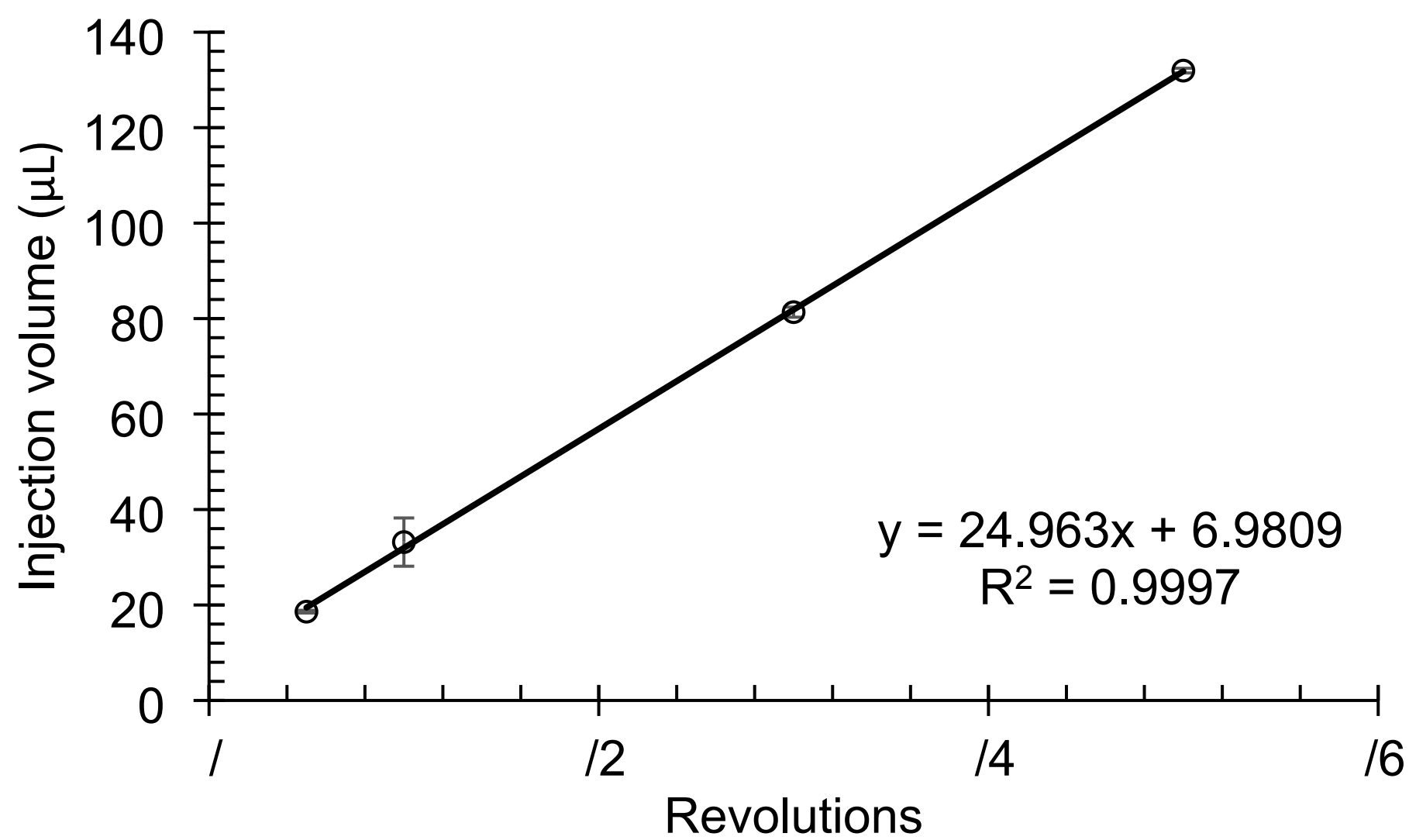

Figure S30. Calibration curve for THF at –80 °C.

Table S13. Calibration data for injections in THF at –80 °C. Injections were performed in duplicate.

Revolutions	Injected volume (µL)
6/12	18.6 ± 0.4
1	33.2 ± 5.1
3	81.4 ± 1.1
5	132.0 ± 0.5

6. Sample Magnetization

6.1. Data Acquisition for Premagnetization Measurements

During the injection, the sample was monitored (^{19}F) with Bruker pseudo-2D program with the following parameters.
Pulse angle: 30 degrees
Number of scans (NS) = 1
Spectral center (o1p) = –100.01 ppm
Spectral width (sw) = 241.50 ppm
Acquisition time = 0.5767 sec
Variable delay (vd): 30 ms (for all scans)
Relaxation delay (d1) = 0.400 sec (1 sec between each scan)

6.2. Monitoring a Species Present Initially (Reagent Injection)

Stock solutions were prepared as followed:
Solution A.
A stock solution of 4-fluorotoluene (**S1**, 63.8 mg, 0.5 mmol, 0.1 M) and 4-fluorobromobenzene (**S2**, 72.6 mg, 0.4 mmol, 40 mM) in toluene was prepared in a 5 mL volumetric flask, and 500 µL of this solution was added to an NMR tube for each experiment.
Solution B.
A stock solution containing 3-fluoronitrobenzene (**S3**, 42.8 mg, 0.2 mmol, 50 mM) in toluene was prepared in a 5 mL volumetric flask. This solution was loaded into the RI syringe for injection.

Procedure: An uncapped NMR tube inside the spectrometer containing **solution A** (500 µL) was injected (RI-NMR) with 83 µL of **solution B** and monitored by ^{19}F NMR (180 scans, 1 s between scans). Before injection, the injection capillary was held immediately above the level of the solution in the NMR tube for 30 s to allow premagnetization of **solution B**.

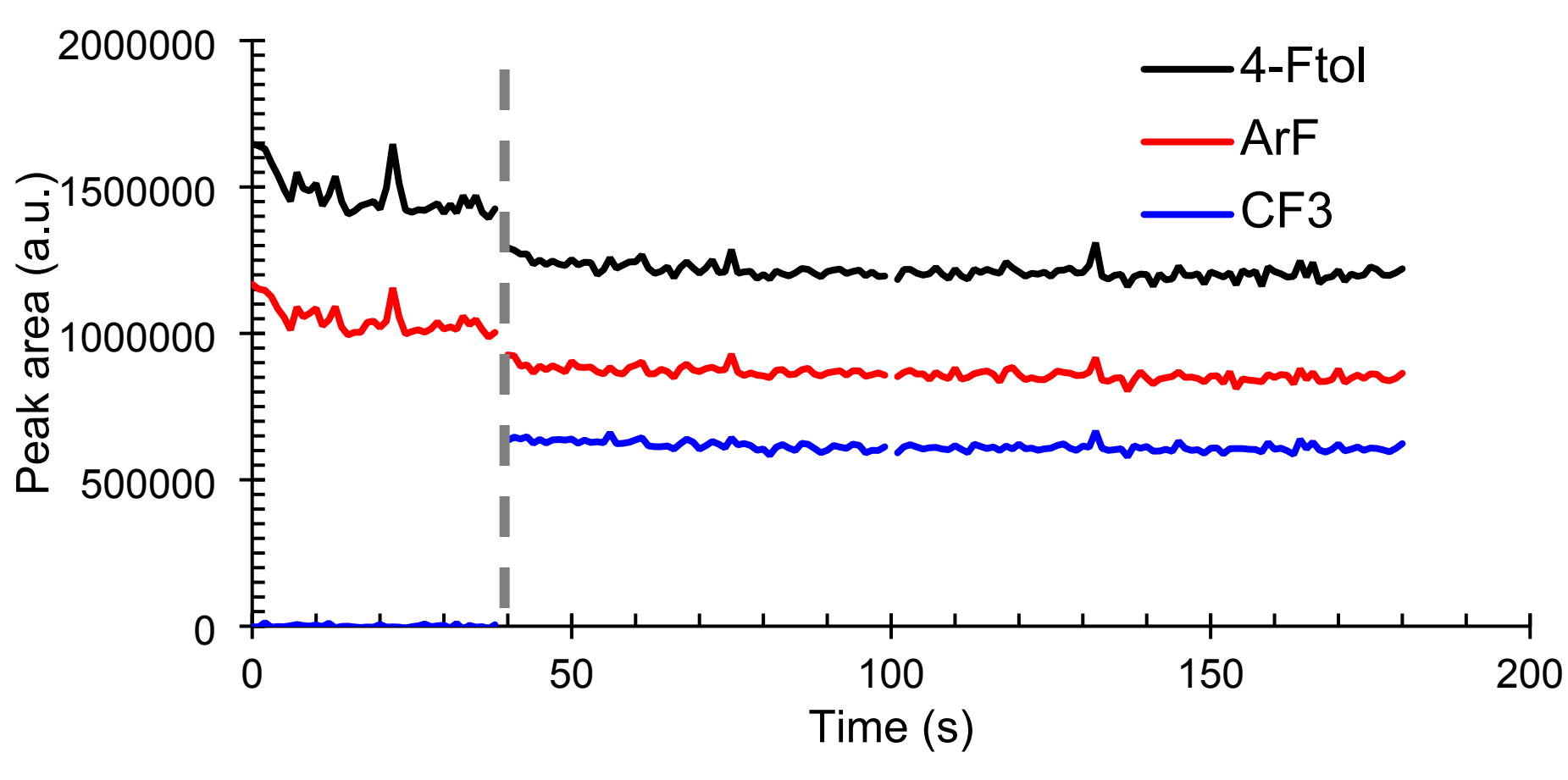

Figure S31. Solution B injected into an NMR tube containing **solution A** after a 30 s premagnetization period.

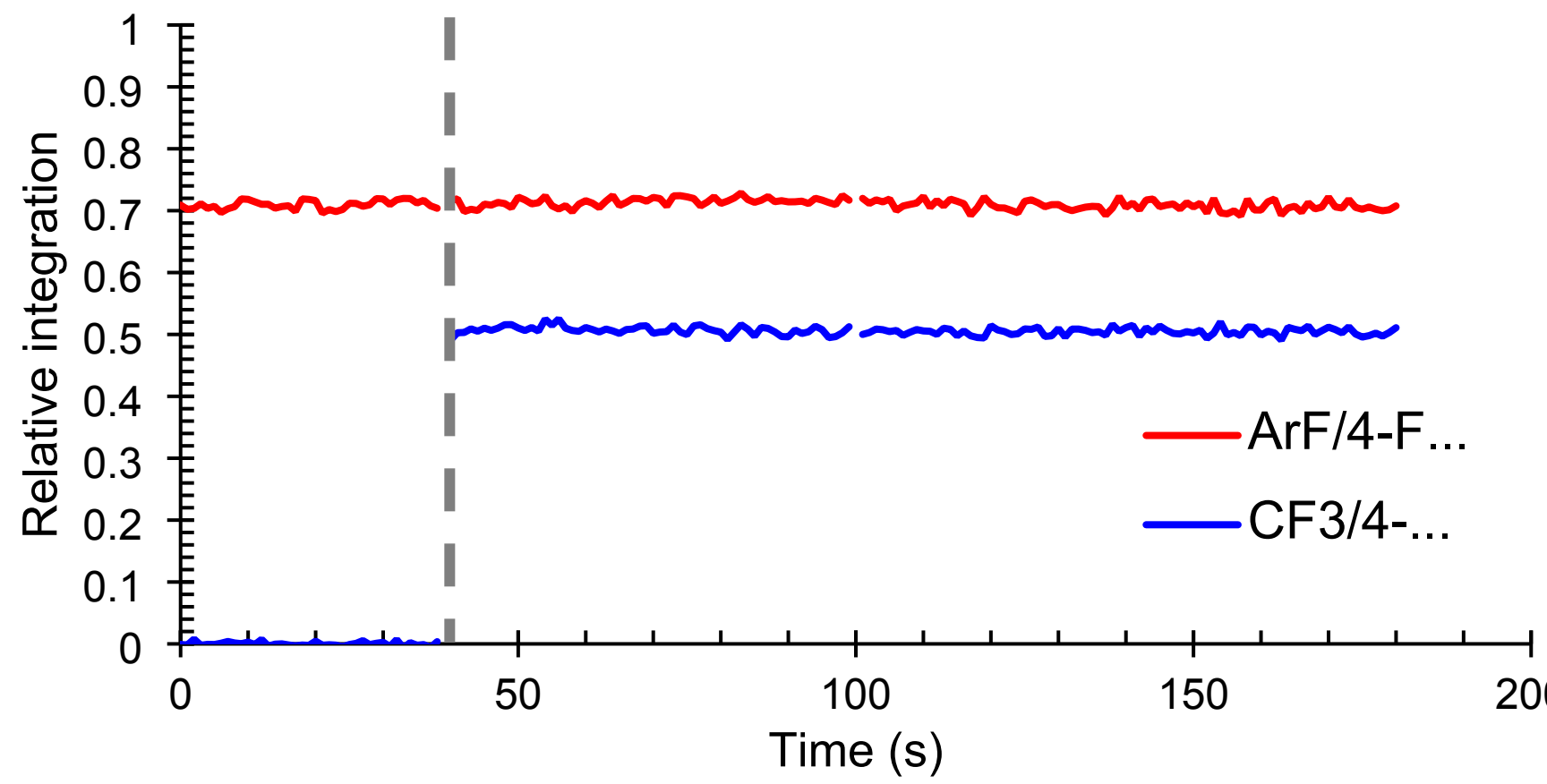

Figure S32. Apparent ratios of species versus **S1** for the experiment described above.

6.3. Monitoring a Species Injected by RI-NMR (Substrate Injection)

Stock solutions were prepared as followed:

Solution C.

A stock solution of 4-fluorotoluene (**S1**, 274.4 mg, 1.0 mmol, 0.1 M) in toluene was prepared in a 25 mL volumetric flask, and 500 μL of this solution was added to an NMR tube for each experiment.

Solution D.

A stock solution containing 4-fluorobromobenzene (**S2**, 175.0 mg, 1.0 mmol, 0.1 M) and 3-fluoronitrobenzene (**S3**, 90.8 mg, 0.47 mmol, 47 mM) in toluene was prepared in a 10 mL volumetric flask, and loaded into the RI syringe for injection.

Procedure: An uncapped NMR tube inside the spectrometer containing **solution C** (500 μL) was injected (RI-NMR) with 83 μL of **solution D** and monitored by ^{19}F NMR (180 scans, 1 s between scans). Before injection, the injection capillary was held immediately above the level of the solution in the NMR tube for the 30 s to ensure premagnetization of **solution D**.

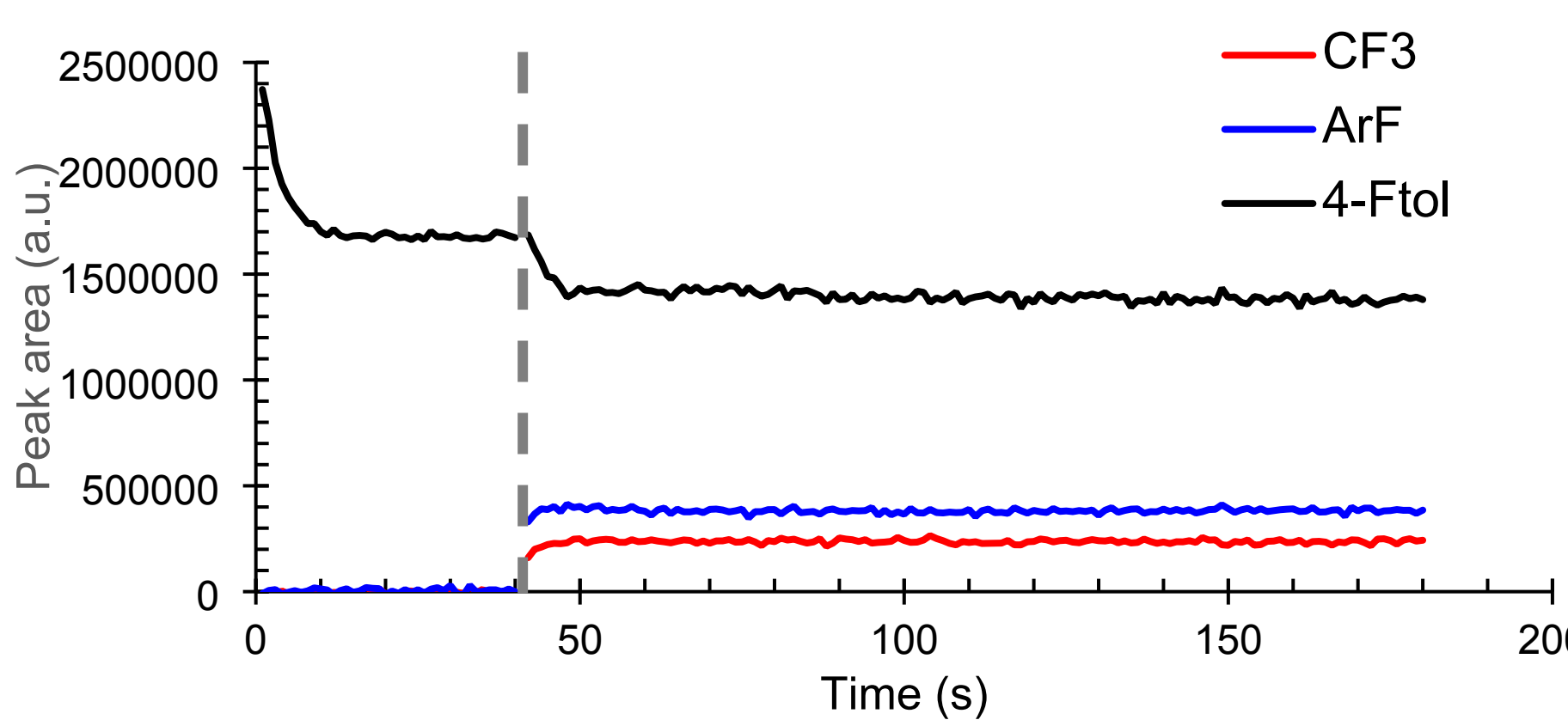

Figure S33. **Solution D** injected into an NMR tube containing **solution C** immediately after lowering the RI-NMR apparatus into the tube.

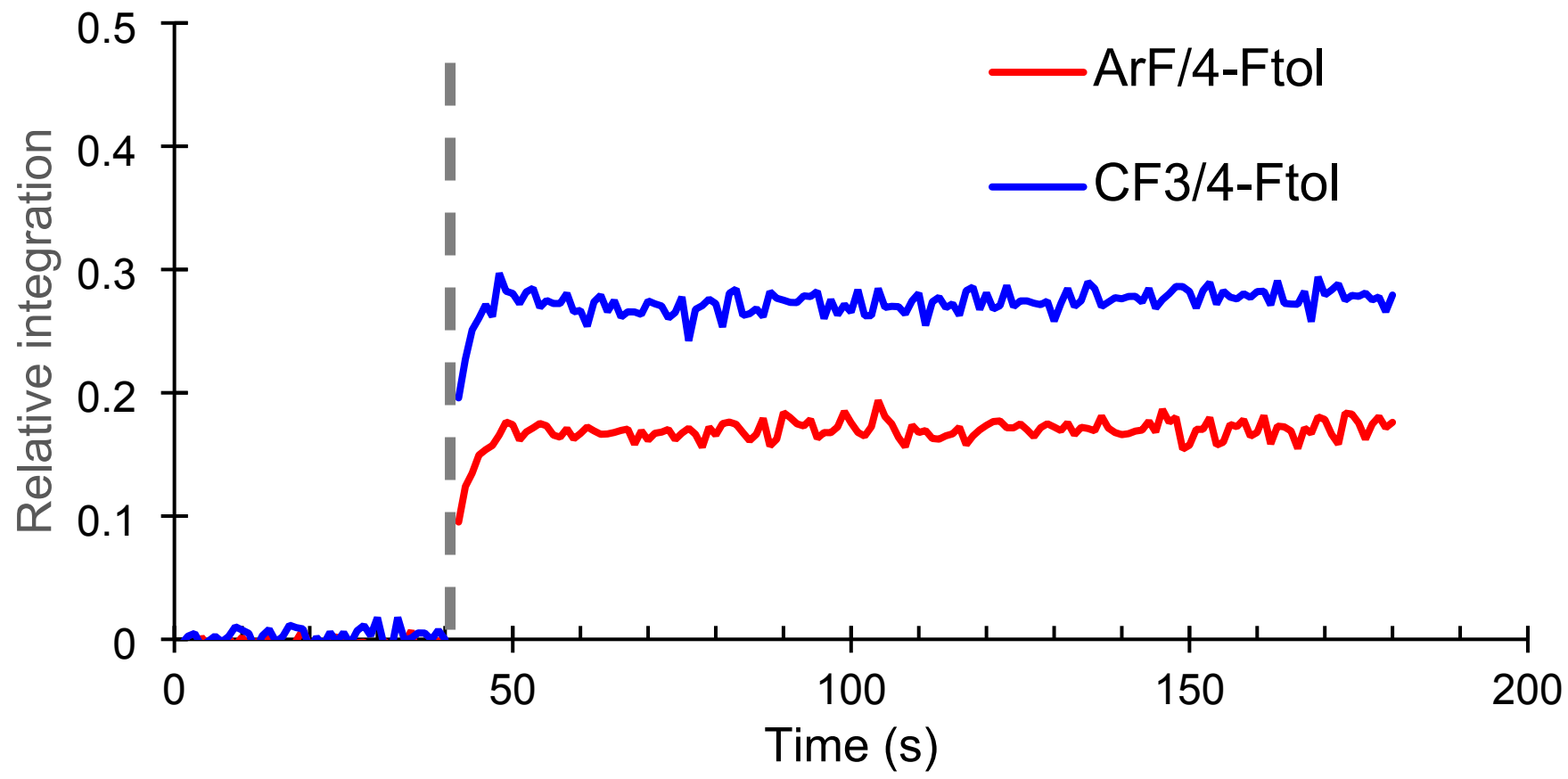

Figure S34. Apparent ratios of species versus 4-fluorotoluene for the experiment described above.

Procedure: An uncapped NMR tube inside the spectrometer containing **solution C** (500 μL) was injected (RI-NMR) with 83 μL of **solution D** and monitored by ^{19}F NMR (180 scans, 1 s between scans) immediately after lowering the RI-NMR apparatus into the NMR tube.

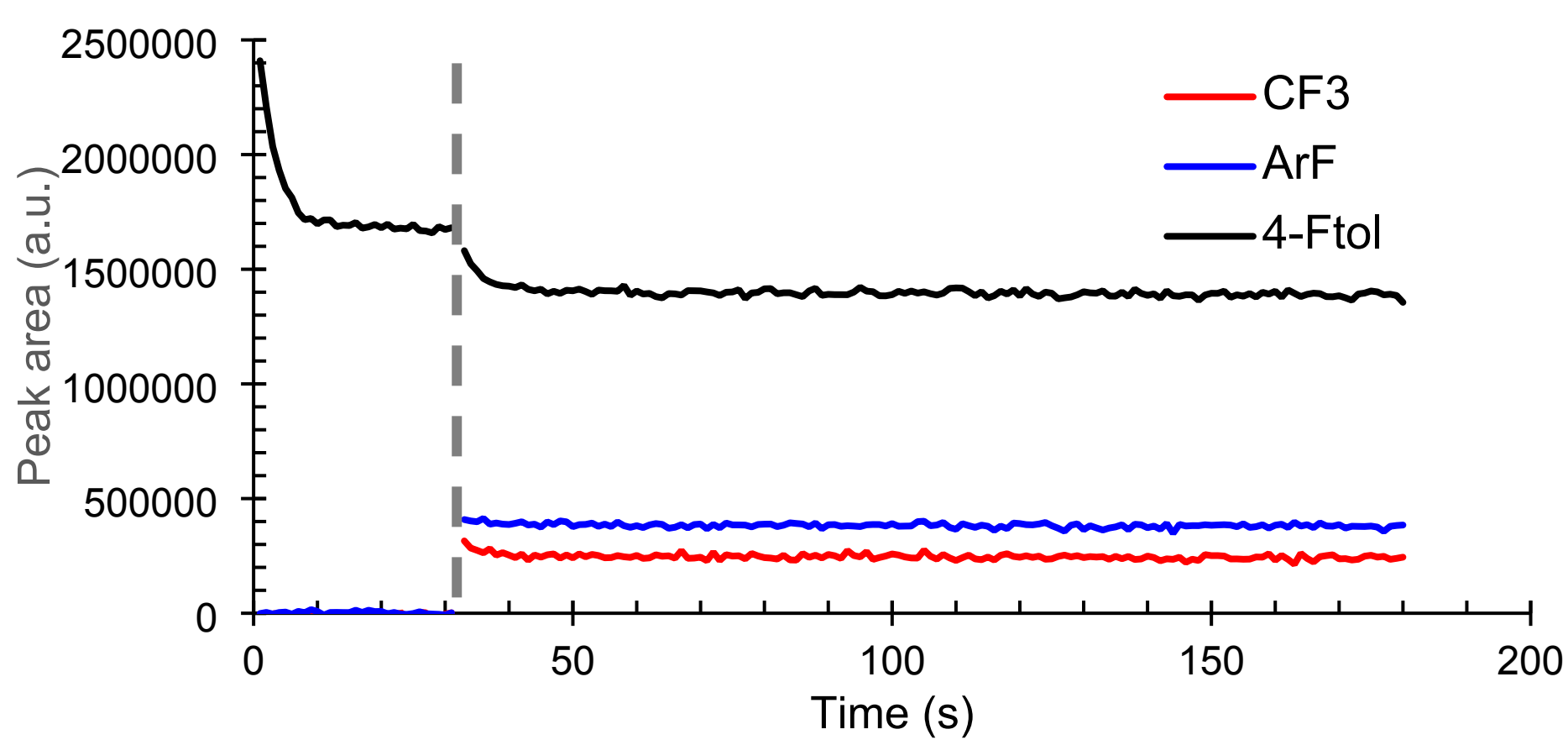

Figure S35. **Solution D** injected into an NMR tube containing **solution C** after 30 s premagnetization period.

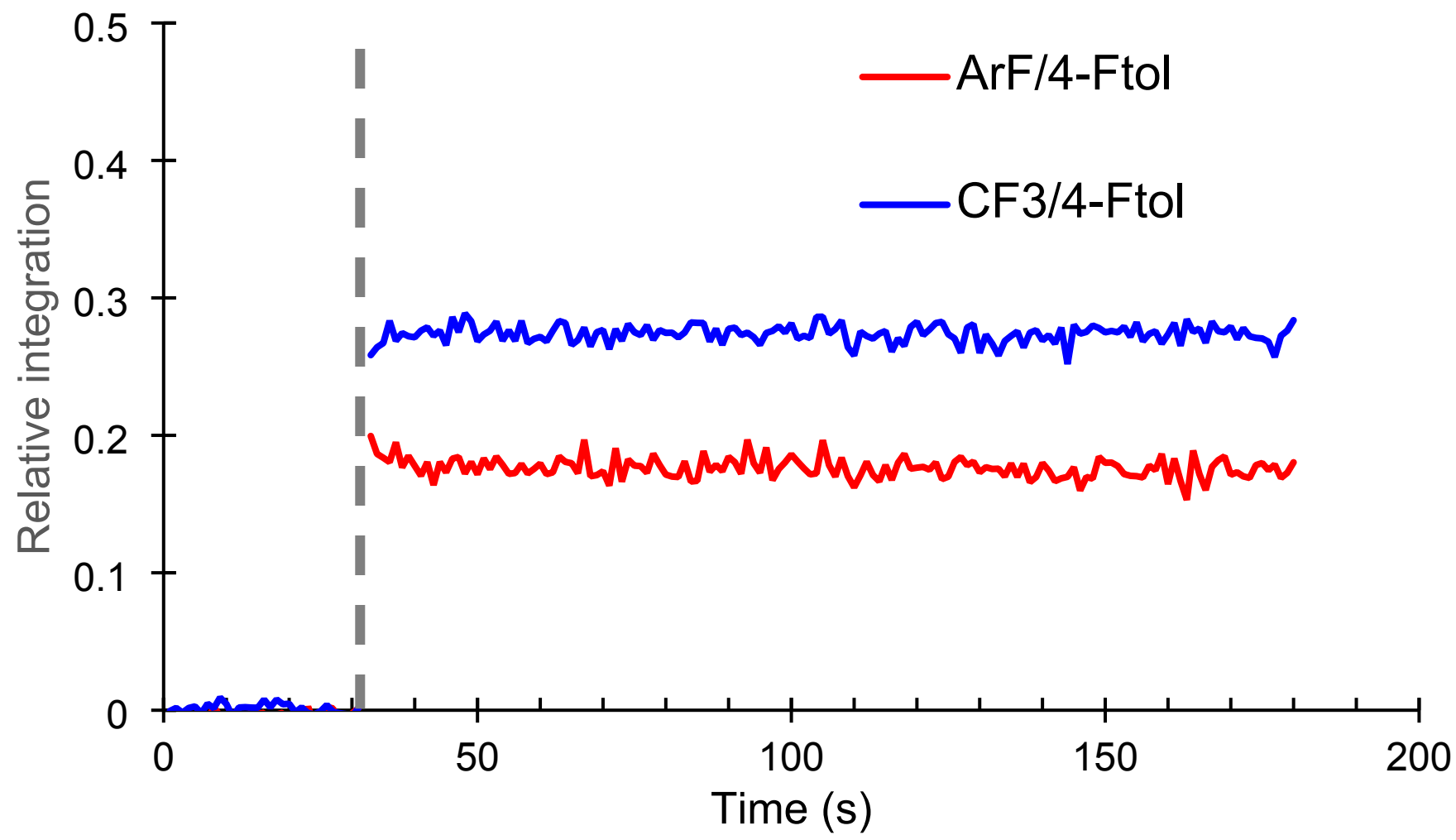

Figure S36. Apparent ratios of species versus 4-fluorotoluene for the experiment described above.

6.4. Discussion of Sample Premagnetization

The time required to achieve sample magnetization is related to T_1 – after 5 × T_1, the sample will have achieved over 99% magnetization. To ensure adequate magnetization of the injected solution, the injector capillary tip is positioned ~1 cm above the solution for at least 5 × T_1 before injection. The experiments described above monitor the apparent molar ratios of 4-fluorobromobenzene (**S2**, T_1 = 3.90 ± 0.13 s) and 3-trifluoronitrobenzene (**S3**, T_1 = 1.70 ± 0.01 s) versus **S1**. The premagnetization period was defined as 30 s, during which the RI apparatus was suspended just above the NMR tube solution, or experiments were conducted with no premagnetization period, and injection was performed as quickly as possible after lowering the apparatus into the sample tube.

Reagent Injection: When monitoring a species present in the NMR tube prior to injection, the injection event leads to a decrease in absolute signal intensity of all species present in the initial solution due to dilution. For example, when **solution B** is injected into **solution A**, the absolute signal intensity of **S1** and **S2** decreased upon injection (**Figure S31**, black and red traces). Because both **S1** and **S2** are fully magnetized, and diluted by the same amount, no decrease in the apparent molar ratio of **S2** is observed (Figure **S32**, red trace). The apparent molar ratio of the injected **S3** is essentially constant immediately after injection (**Figure S32**, blue trace). Thus, reagent injection, where the substrate of interest is present in the tube prior to injection, is preferred where possible, as magnetization errors are much less significant.

Substrate Injection: In contrast, measuring the concentration of a species injected by RI-NMR (substrate injection) requires premagnetization of the injected solution prior to injection. Without the premagnetization period, the absolute signal intensity of the injected species increases over time as the sample achieves magnetization. For example, when **solution D** was injected into **solution C**, the absolute signal intensity of **S2** and **S3** increases over the first several seconds, while the absolute intensity of **S1** decreases (**Figure S33**). During this time, the apparent molar ratio of **S2** and **S3** increases relative to **S1** (**Figure S34**). This is due

to both incomplete reagent magnetization (leading to an increase in signal intensity of substrate over time) and dilution of the total sample (leading to a decrease in signal intensity of the internal standard).

To address this issue, the injection solution is held above the solution for a premagnetization period of at least $5 \times T_1$ before injection. For example, when **solution D** is injected into **solution C** after a 30 s premagnetization period, a steady apparent molar ratio of **S2** and **S3** versus **S1** is achieved within 5 s (**Figures S35** and **S36**). During most experiments where the delay between scans is larger than $5 \times T_1$, this error will be resolved within one scan. This procedure enables accurate integral values to be obtained during substrate injection experiments.

6.5. T_1 Measurements of Substrates

A standard ^{1}F NMR inversion-recovery sequence (180° - τ - 90°) was employed to measure the spin-lattice relaxation time constant (T_1). The spectral center was moved to the signal peak for data collection, and the peak area was plotted against time and fitted to the exponential decay model:

$$M = M_0(1 - 2e^{\tau/T_1})$$

4-Fluorotoluene (S1)

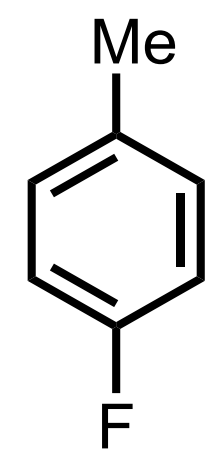

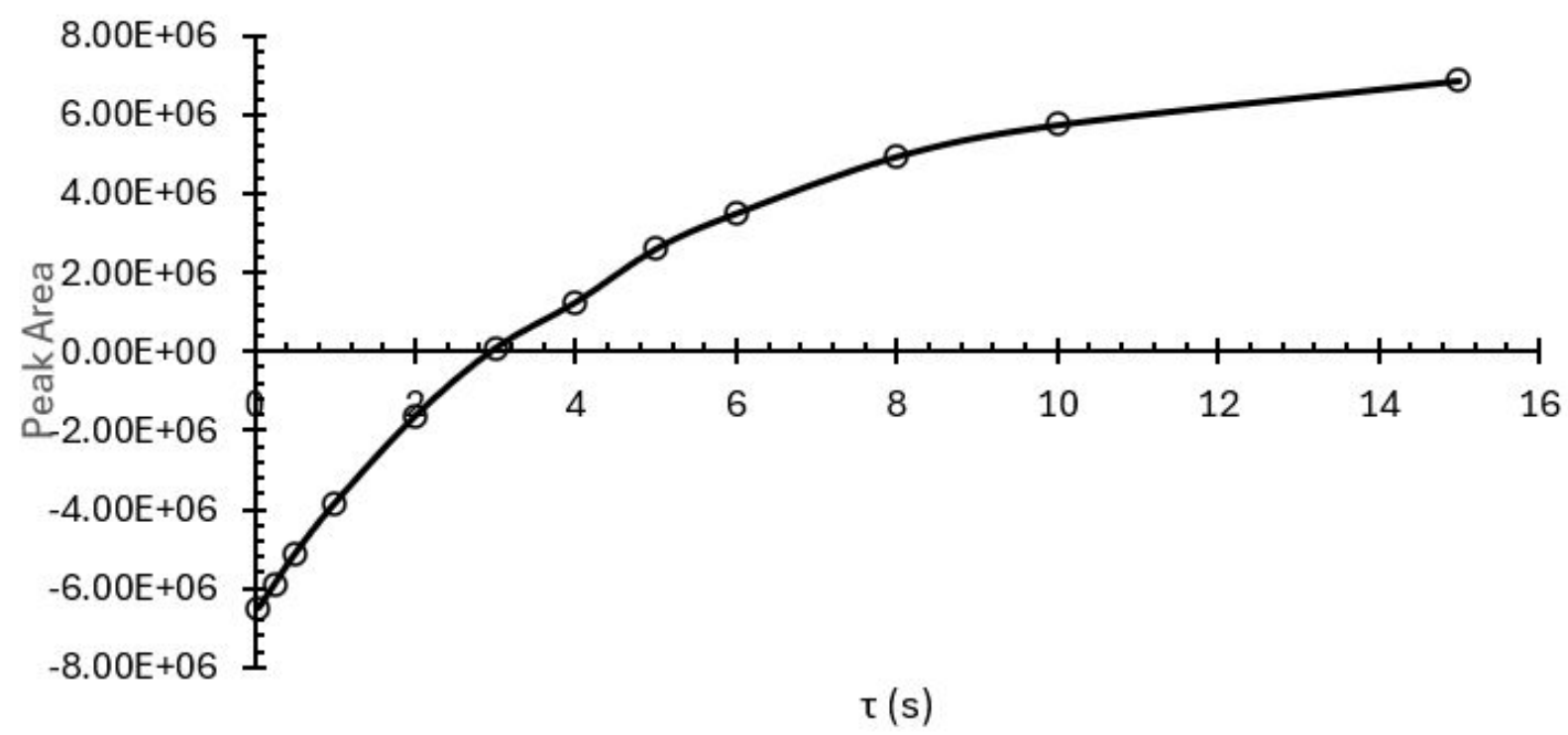

Figure S37. Inversion-recovery plot for **S1**.

Fitting Results

Acquisition parameters

Number of scans (NS) = 2

Spectral center o1p = –118.16 ppm

Spectral width sw = 241.50 ppm

Acquisition time = 0.5767 sec

Relaxation delay (d1) = 45 sec

Fitting parameters

Function f(t) = M0*(1-2*exp(-t/T1))

Fitting results in 95% confidence bonds (R^2 = 0.999)

T_1 = 4.20 ± 0.03 (sec)

M_0 = (6.84 ± 0.11) × 10^6

4-Bromofluorobenzene (S2)

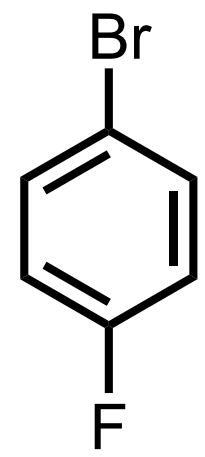

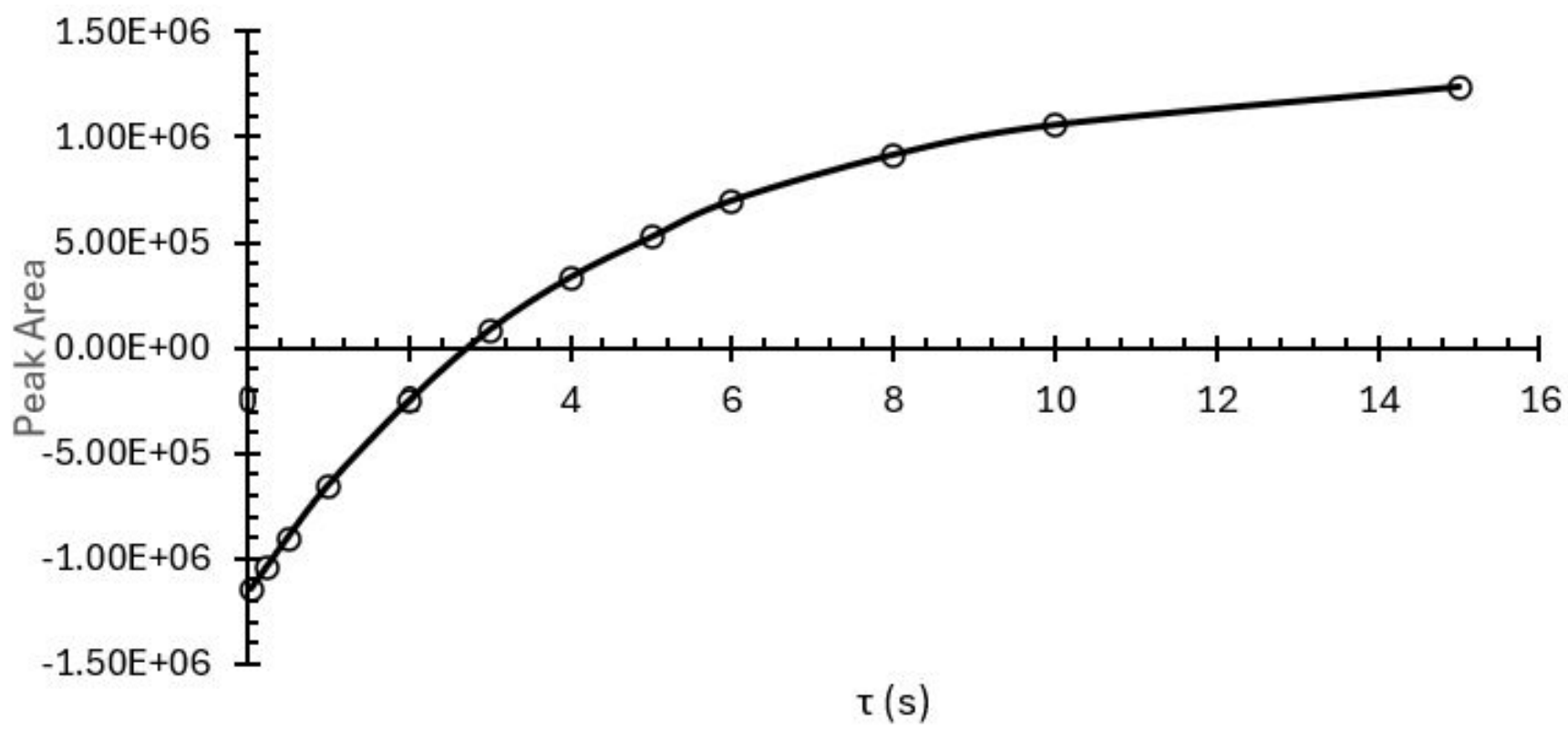

Figure S38. Inversion-recovery plot for **S2**.

Fitting Results

Acquisition parameters

Number of scans (NS) = 2

Spectral center o1p = –115.35 ppm

Spectral width sw = 241.50 ppm

Acquisition time = 0.5767 sec

Relaxation delay (d1) = 45 sec

Fitting parameters

Function f(t) = M0*(1-2*exp(-t/T1))

Fitting results in 95% confidence bonds (R^2 = 0.999)

T_1 = 3.90 ± 0.13 (sec)

M_0 = (1.22 ± 0.03) × 10^6

3-Nitrotrifluorotoluene (S3)

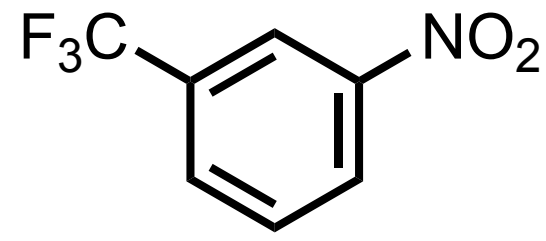

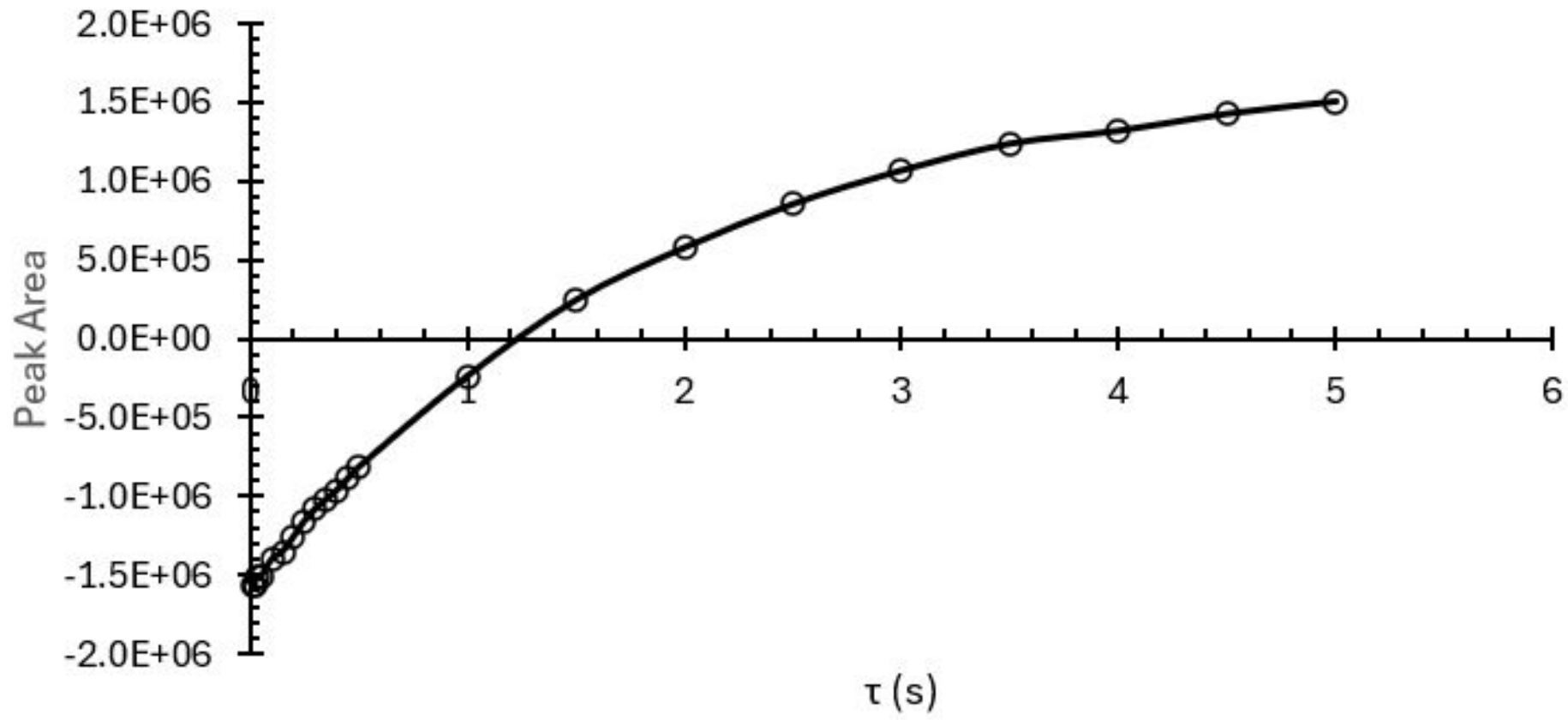

Figure S39. Inversion-recovery plot for **S3**.

Fitting Results

Acquisition parameters

Number of scans (NS) = 2

Spectral center o1p = –62.94 ppm

Spectral width sw = 241.50 ppm

Acquisition time = 0.5767 sec

Relaxation delay (d1) = 45 sec

Fitting parameters

Function f(t) = M0*(1-2*exp(-t/T1))

Fitting results in 95% confidence bonds ($R^2 = 0.999$)

$T_1 = 1.70 \pm 0.01$ (sec)

$M_0 = (1.62 \pm 0.02) \times 10^6$

7. Temperature Variation During Injection

7.1. Calibration of NMR Temperature Control

The temperature profile was probed using the chemical shift difference of two signals in ethylene glycol at 80 °C, and methanol at −80 °C. The accuracy of the spectrometer temperature control was first verified by allowing the sample to reach thermal equilibrium at different temperatures in 10 °C intervals. The internal temperature and the instrument setting were found to be in good correlation (**Figure S40**).

Calibration equations

Ethylene glycol ($\Delta\delta = \delta O\underline{\mathbf{H}} - \delta C\underline{\mathbf{H}}_2$)

$$T(°C) = 193.35 - \Delta\delta \times 102$$

Methanol ($\Delta\delta = \delta O\underline{\mathbf{H}} - \delta C\underline{\mathbf{H}}_3$)

$$T(°C) = 135.85 - \Delta\delta \times 36.54 - \Delta\delta^2 \times 21.85$$

(a)
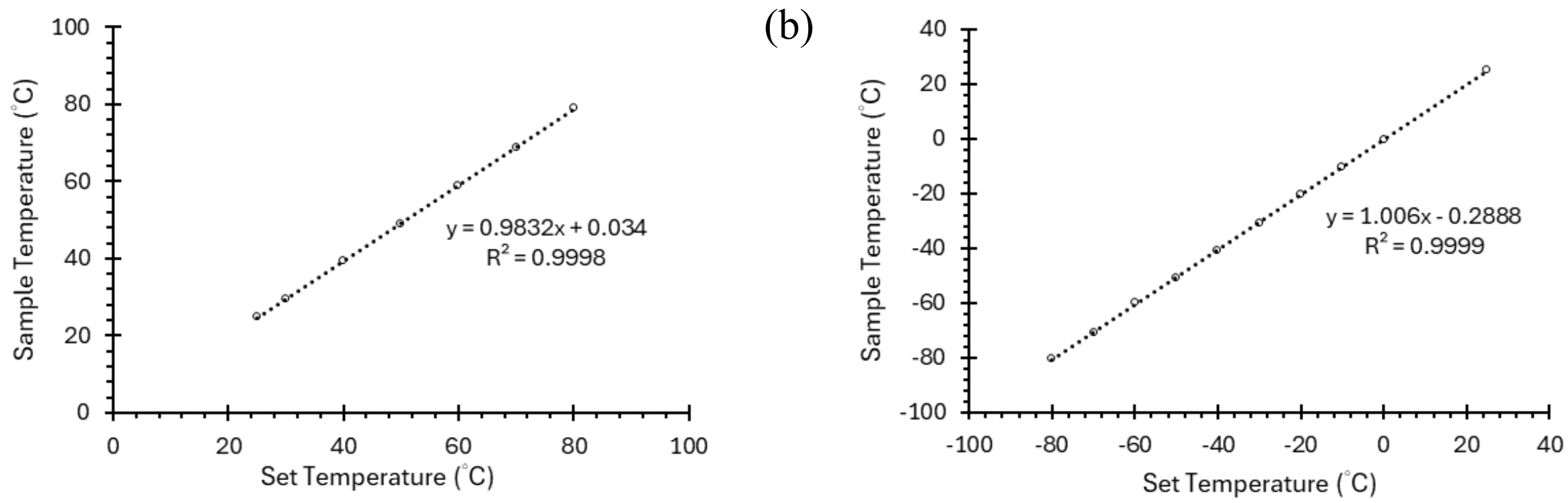

Figure S40. Validation of spectrometer temperature control. (a) Ethylene glycol as reporter. (b) Methanol as reporter.

7.2. Data Acquisition for Temperature Measurements

During the injection, the sample was monitored with Bruker pseudo-2D program with the following parameters.
Pulse angle: 30 degrees
Number of scans (NS) = 1
Spectral center (o1p) = 6.18 ppm
Spectral width (sw) = 19.22 ppm
Acquisition time = 3.41 sec
Variable delay (vd): 600 ms (for all scans)
Relaxation delay (d1) = 1 sec (5 sec between each scan)

7.3. Effect of Injection on Sample Temperature

We examined the impact of injection on sample temperature as a function of injection volume at two temperatures. To allow the solution inside the injection capillary to achieve thermal equilibrium, the capillary was held above the solution for 30 s before injection. Injection of various volumes of ethylene glycol (16, 30, 83, 136 µL, or 0.5, 1, 3, and 5 revolutions, respectively) into 500 µL of ethylene glycol at 80 °C resulted in relatively minor (< 5 °C) to major (> 15 °C) fluctuation depending on the injection volume, which recovered within 100 s. (**Figure S41**).

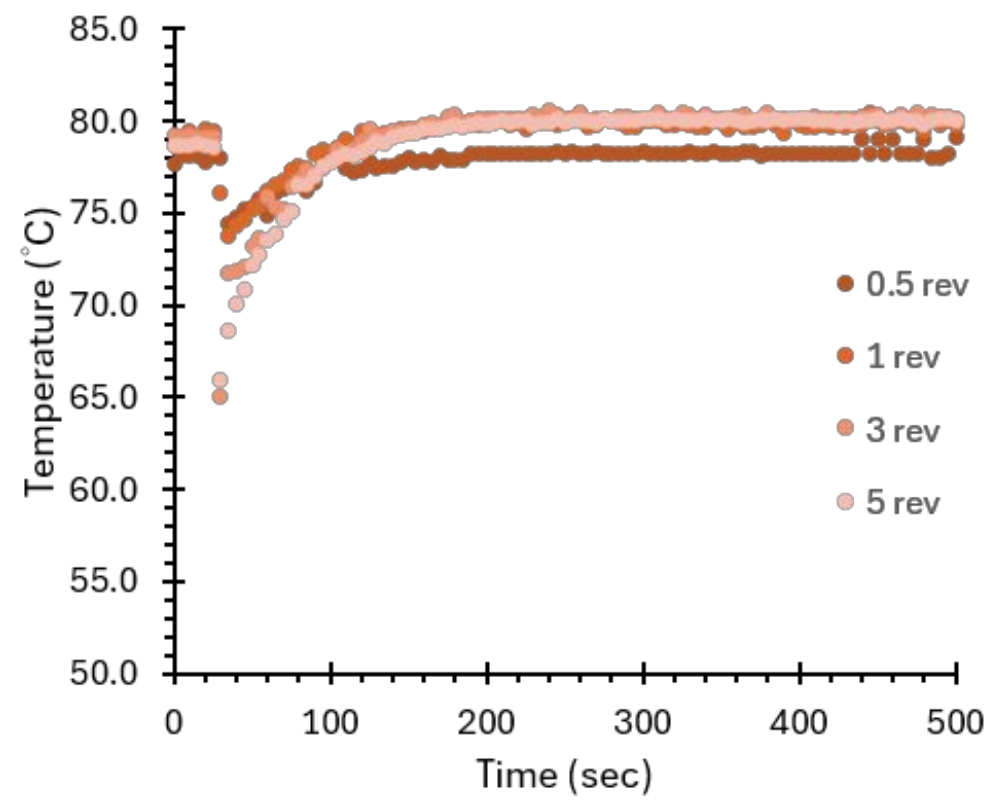

Figure S41. Temperature evolution curve different injection volumes to 500-µL samples equilibrated at 80 °C.

Similarly, we investigated the effect of injection on sample temperature at −80 °C using 500-µL methanol samples. (**Figure S42**). Temperature recovery was slower at −80 °C (150 s).

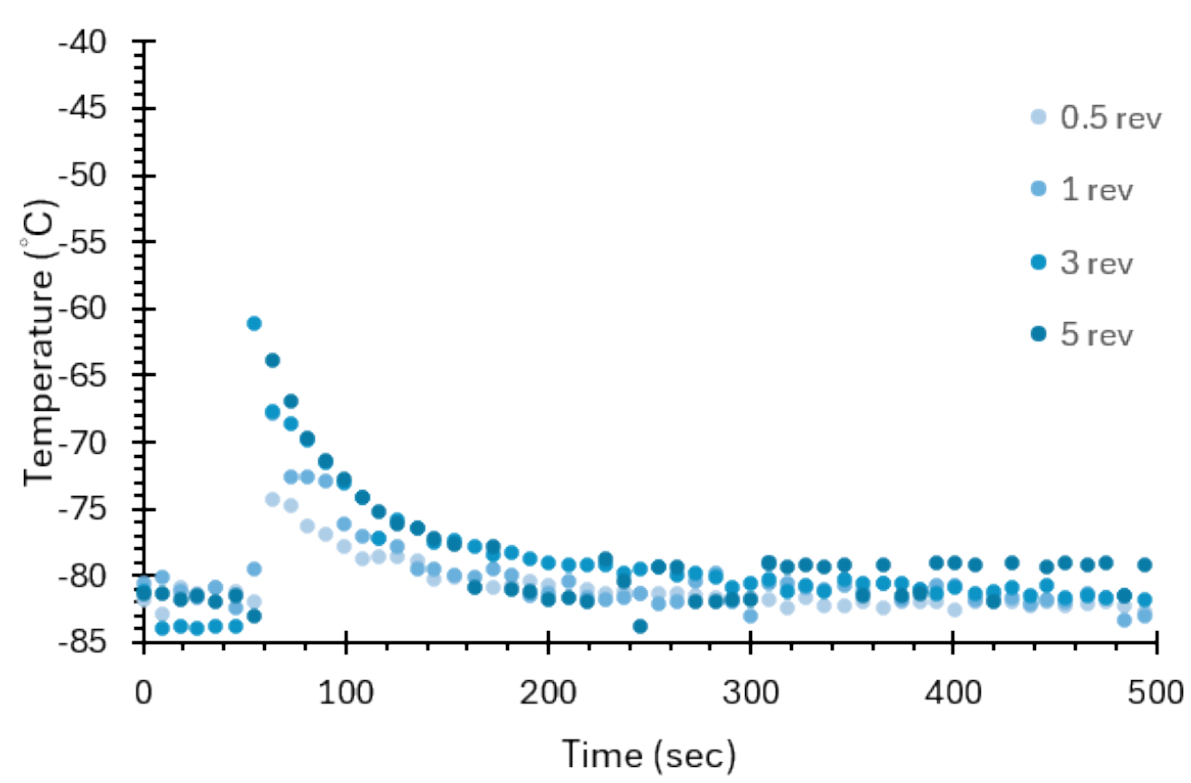

Figure S42. Temperature evolution curve different injection volumes to 500-µL samples equilibrated at −80 °C.

8. Experimental Procedures

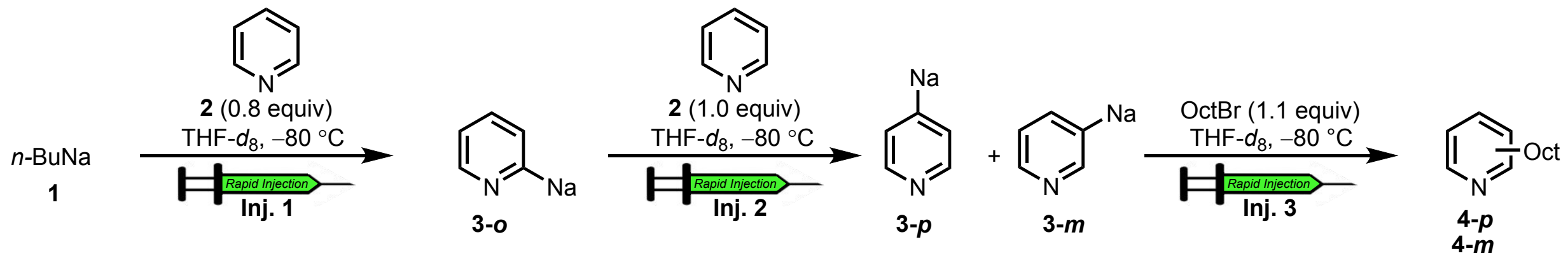

8.1. Pyridine Deprotonation with *n*-BuNa

8.1.1. Experimental Procedure

The following stock solutions were freshly prepared for the reaction:

- A stock solution of **2** (2.0 M) was prepared by dissolving **2** (160 mg, 2.0 mmol) in freshly distilled (NaK) tetrahydrofuran-d_8 in a 1.0 mL volumetric flask in a glove box.
- A stock solution of tetramethylsilane (73 mM) was prepared by diluting tetramethylsilane (10 µL, 73.4 µmol) in freshly distilled (NaK) tetrahydrofuran-d_8 in a 1.0 mL volumetric flask in a glove box. The solution was loaded into syringe A.
- A stock solution of 1-bromooctane (1.1 M) was prepared by diluting 1-bromooctane (193 mg, 1.00 mmol) in freshly distilled (NaK) tetrahydrofuran-d_8 in a 1.0 mL volumetric flask in a glove box. The solution was loaded into syringe B.

In a glovebox, *n*-BuNa (4.0 mg, 50 µmol, 1.0 equiv) was weighed into an oven-dried NMR tube. The tube was fitted with a rubber septum, removed from the glovebox and submerged into a dry ice-acetone bath (−78 °C) followed by the slow addition of tetramethylsilane stock solution (100 µL, 73 mM, 7.34 µmol, 0.15 equiv) and freshly distilled (NaK) tetrahydrofuran-d_8 (450 µL), followed by mixing on a vortexer in 1-2 s bursts. The tube was maintained in the –78 °C bath until it was placed into the NMR spectrometer that was precooled to –80 °C. After 5 min for temperature equilibration, data collection was started and the pyridine solution (18.5 µL, 0.74 equiv) was injected with syringe A. During the same NMR array experiment, additional pyridine solution was injected (25.1 µL, 1.0 equiv) with syringe A at 1873 s (ca 30 min). At 10016 s (ca 2 h after the 2nd injection), 1-bromooctane solution (49.5 µL, 1.10 equiv) was injected with syringe B.

8.1.2. Peak Identification

The peaks of three sodiopyridine isomers and two octylpyridine isomers are assigned based on relative integration and peak patterns and ^{1}H-^{1}H COSY spectrum of a separate injection experiment.[2]

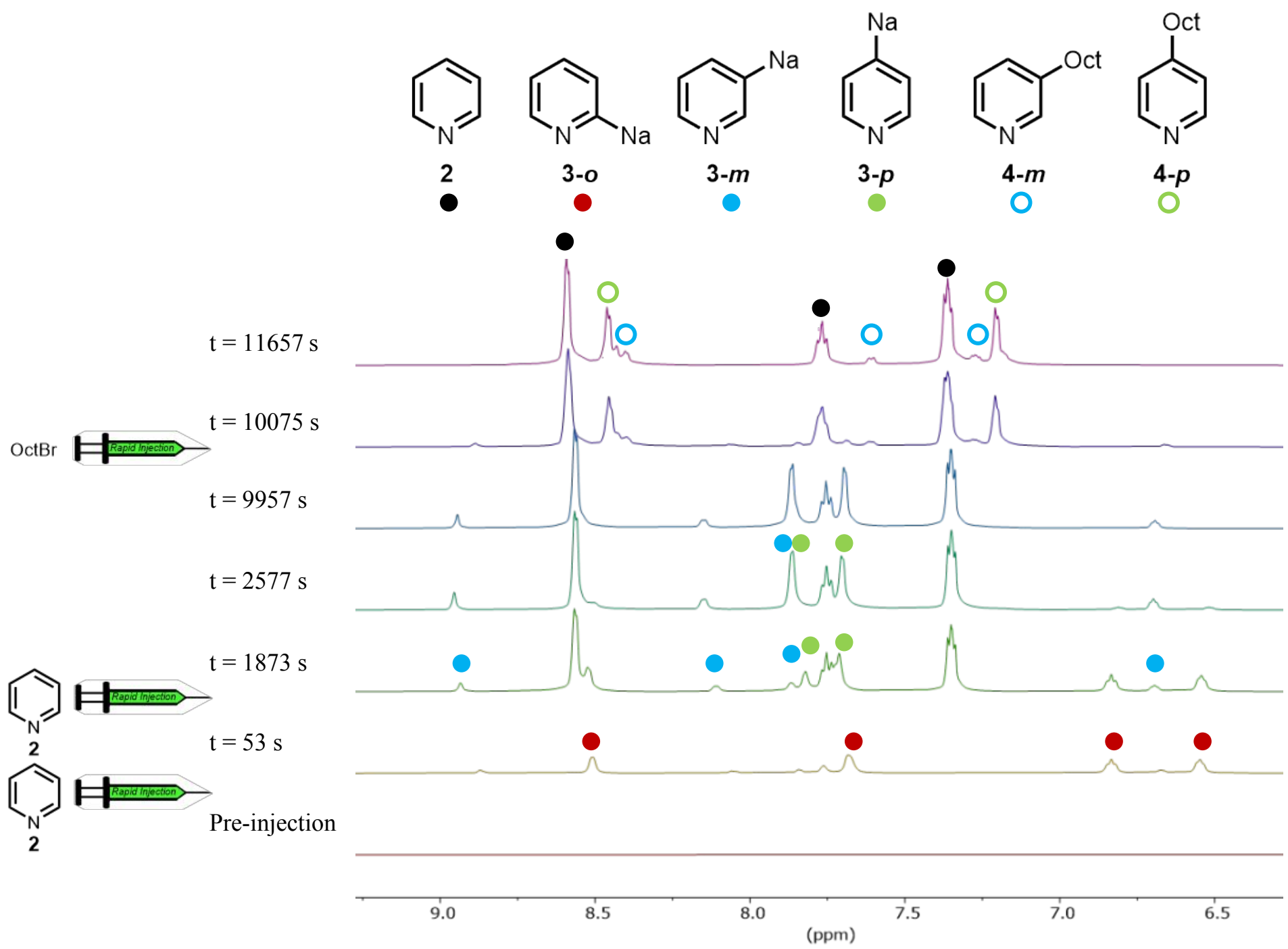

Figure S43. Reaction profile of pyridine with *n*-butyl sodium.

8.1.3. Data Acquisition

During the injection, the sample was monitored with Bruker multizg program with the following parameters.

Pulse angle: 30 degrees
Number of scans (NS) = 1
Spectral center (o1p) = 6.18 ppm
Spectral width (sw) = 19.22 ppm
Acquisition time = 3.41 sec
Relaxation delay (d1) = 50 sec (ca 58 sec between each scan)

8.1.4. Data Analysis

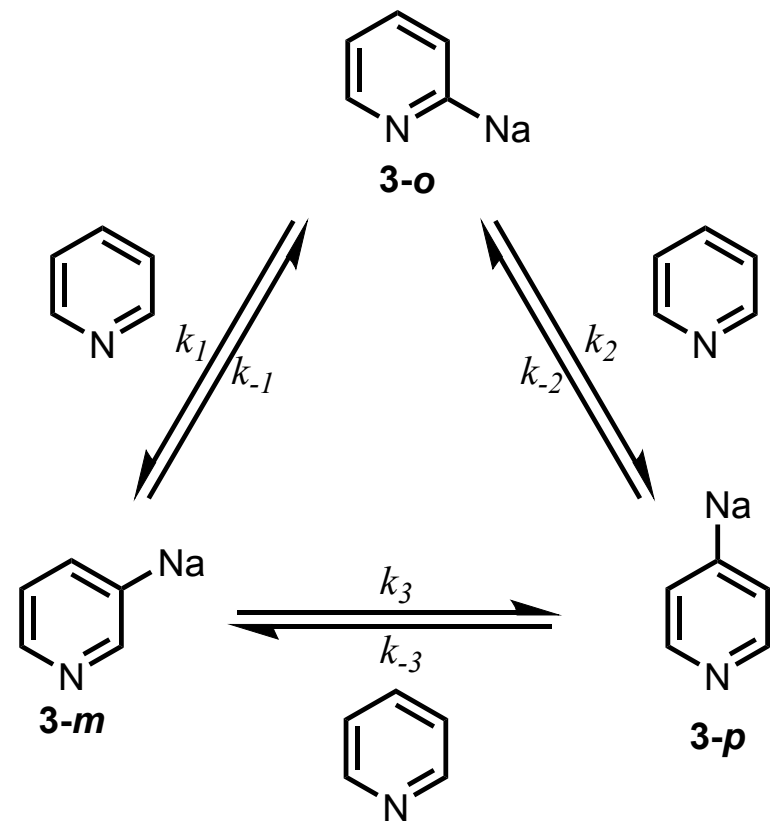

The rate equations between **3-*o***, **3-*m***, **3-*p*** can be written as:

$$\frac{d[\mathbf{3\text{-}}\boldsymbol{o}]}{dt} = -(k_1 + k_2)[\mathbf{2}][\mathbf{3\text{-}}\boldsymbol{o}] + k_{-1}[\mathbf{2}][\mathbf{3\text{-}}\boldsymbol{m}] + k_{-2}[\mathbf{2}][\mathbf{3\text{-}}\boldsymbol{p}]$$

$$\frac{d[\mathbf{3\text{-}}\boldsymbol{m}]}{dt} = k_1[\mathbf{2}][\mathbf{3\text{-}}\boldsymbol{o}] - (k_{-1} + k_3)[\mathbf{2}][\mathbf{3\text{-}}\boldsymbol{m}] + k_{-3}[\mathbf{2}][\mathbf{3\text{-}}\boldsymbol{p}]$$

$$\frac{d[\mathbf{3\text{-}}\boldsymbol{p}]}{dt} = k_2[\mathbf{2}][\mathbf{3\text{-}}\boldsymbol{o}] + k_3[\mathbf{2}][\mathbf{3\text{-}}\boldsymbol{m}] - (k_{-3} + k_{-2})[\mathbf{2}][\mathbf{3\text{-}}\boldsymbol{p}]$$

This can be expressed in a matrix form:

$$\begin{bmatrix} \frac{d[\mathbf{3\text{-}}\boldsymbol{o}]}{dt} \\ \frac{d[\mathbf{3\text{-}}\boldsymbol{m}]}{dt} \\ \frac{d[\mathbf{3\text{-}}\boldsymbol{p}]}{dt} \end{bmatrix} = [\mathbf{2}] \begin{bmatrix} -(k_1 + k_2) & k_{-1} & k_{-2} \\ k_1 & -(k_{-1} + k_3) & k_{-3} \\ k_2 & k_3 & -(k_{-3} + k_{-2}) \end{bmatrix} \begin{bmatrix} [\mathbf{3\text{-}}\boldsymbol{o}] \\ [\mathbf{3\text{-}}\boldsymbol{m}] \\ [\mathbf{3\text{-}}\boldsymbol{p}] \end{bmatrix}$$

Since the isomerization does not consume **2**, the apparent rate constants can be written as:

$$k_{i,\,obs} = [\mathbf{2}]k_i$$

And **3-*o***, **3-*m***, **3-*p*** can be expressed as a ratio of isomers for fitting:

$$[A] = [\mathbf{3\text{-}}\boldsymbol{o}] + [\mathbf{3\text{-}}\boldsymbol{m}] + [\mathbf{3\text{-}}\boldsymbol{p}]$$

$$r_o = \frac{[\mathbf{3\text{-}}\boldsymbol{o}]}{[A]};\ r_m = \frac{[\mathbf{3\text{-}}\boldsymbol{m}]}{[A]};\ r_p = \frac{[\mathbf{3\text{-}}\boldsymbol{p}]}{[A]}$$

Therefore, the rate equation for fitting is as follows:

$$\frac{d}{dt}\begin{bmatrix} r_o \\ r_m \\ r_p \end{bmatrix} = \begin{bmatrix} -(k_1 + k_2) & k_{-1} & k_{-2} \\ k_1 & -(k_{-1} + k_3) & k_{-3} \\ k_2 & k_3 & -(k_{-3} + k_{-2}) \end{bmatrix}_{obs} \begin{bmatrix} r_o \\ r_m \\ r_p \end{bmatrix}$$

With this setup, the differential equation was evaluated with ode45 solver, the rate constants were optimized using nonlinear least-square curve fitter tool (lsqcurvefit) in Matlab®, and the 95% confidence intervals were evaluated with nonlinear regression parameter confidence

intervals tool (nlparci). In the fitting, k_{-1}, k_{-2} were manually set to 0 due to the negligible concentration of ***3-o*** towards equilibrium.

Full Matlab® Script for optimization

```
%Data import
A0 = A(1); B0 = B(1); C0 = C(1);
Conc_input = [A; B; C];
y0 = [A(1); B(1); C(1)];

%Initial guess
%p = [k1 k2 k3 j1 j2 j3];
startpt = [0.00001, 0.00001, 0.00001, 0.00001, 0.00001, 0.00001];

%Boundaries
% [k1 k2 k3 j1 j2 j3];
lb = [0, 0, 0, 0, 0, 0];
ub = [1, 1, 1, 0, 0, 1];

%Fitting method
options = optimoptions('lsqcurvefit', 'Display', 'iter', 'ScaleProblem', 'obj-and-constr',
'StepTolerance', 1e-15, 'FunctionTolerance', 1e-15);
[k_final, resnorm, residual, exitflag, output, lambda, jacobian] =
lsqcurvefit(@(p,t)fittingmodel(p,t,y0), startpt, t, Conc_input, lb, ub, options);

%Calculate CI
ci = nlparci(k_final, residual, 'jacobian', jacobian, 'alpha', 0.05);
errbar = (ci(:,2) - ci(:,1))/2;

%generate fitted curves
    k1 = k_final(1); k2 = k_final(2); k3 = k_final(3);
    j1 = k_final(4); j2 = k_final(5); j3 = k_final(6);
    [~, y_fitted] = ode45(@(t,y) [
    -(k1+k2) j1 j2;
    k1 -(j1+k3) j3;
    k2 k3 -(j2+j3)] * y, t, y0);

%Extrapolation for eqm ratio
    t_eqm = [0,1e8];
    [~, y_eqm] = ode45(@(t,y) [
    -(k1+k2) j1 j2;
    k1 -(j1+k3) j3;
    k2 k3 -(j2+j3)] * y, t_eqm, y0);

    y_inf = y_eqm(end, :);

%Outputs
disp("k1 k2 k3 j1 j2 j3 =")
disp(vpa(k_final, 10));

disp("95% Confidence Intervals (Lower, Upper):")
disp(vpa(ci, 10));
disp("error bars:")
disp(vpa(errbar, 10));

disp("predicted equilibium concentrations")
disp(vpa(y_inf, 4));

%-------------functions---------------
%set Fitting models
function GuessStack = fittingmodel(p, t, y0)
    %Assign variables
    k1 = p(1);
    k2 = p(2);
    k3 = p(3);
    j1 = p(4);
    j2 = p(5);
    j3 = p(6);
```

```
    %--------RATE LAW--------
    rate_eqn = @(t,y) [
    -(k1+k2) j1 j2;
    k1 -(j1+k3) j3;
    k2 k3 -(j2+j3)] * y;

    [~, y_out] = ode45(rate_eqn, t, y0);

    %Stacked guess functions
    GuessStack = [
        y_out(:, 1);
        y_out(:, 2);
        y_out(:, 3);
        ];
end
```

8.1.5. Experiment Results

Global Fitting of Equilibration of 3

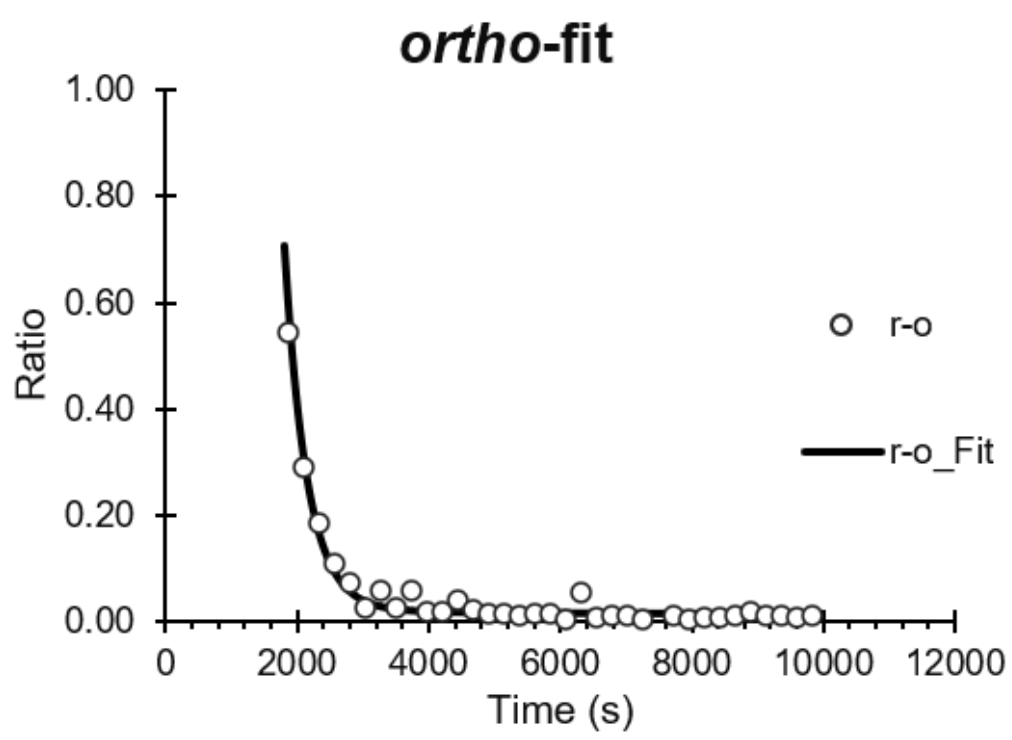

$R^2 = 0.978$

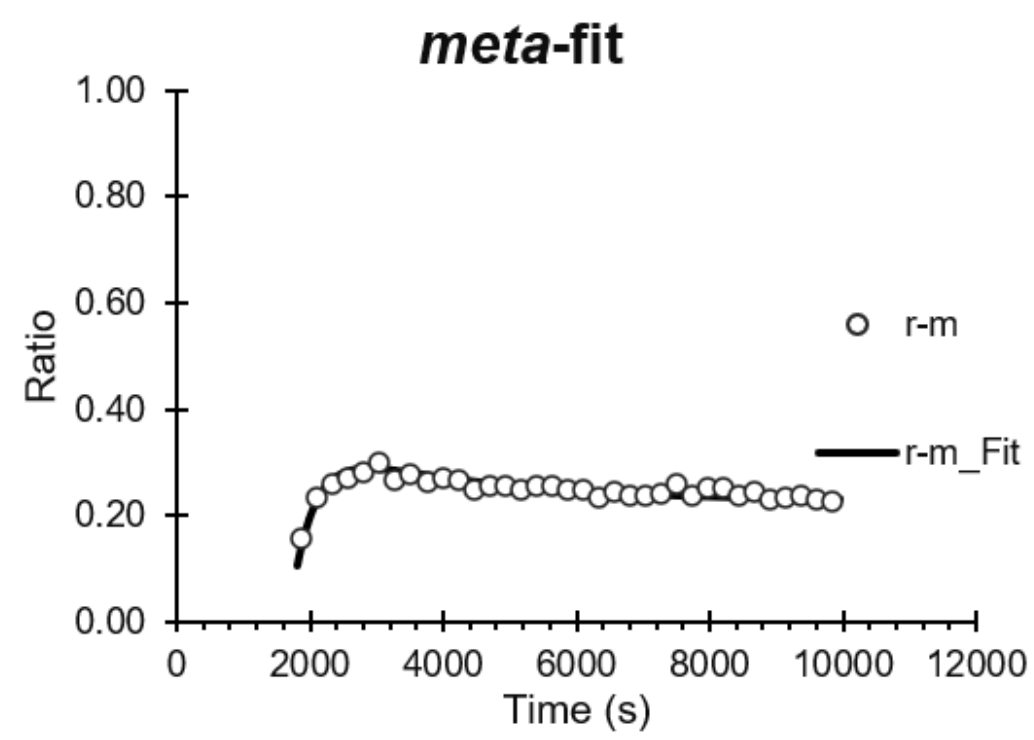

$R^2 = 0.857$

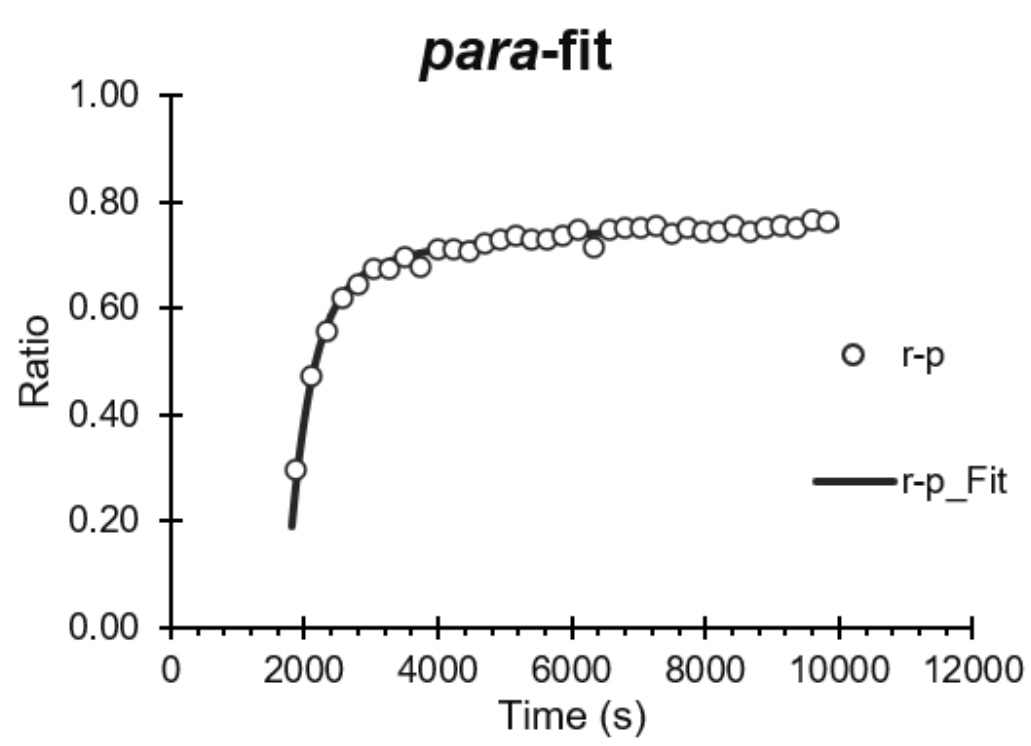

$R^2 = 0.986$

Fitting Results
Sample range: t = 1814 – 9957 s (n = 140)
Observed rate constants:
$k_1 = (8.53 \pm 0.64) \times 10^{-4}\ s^{-1}$
$k_2 = (1.82 \pm 0.07) \times 10^{-3}\ s^{-1}$
$k_3 = (2.93 \pm 2.60) \times 10^{-4}\ s^{-1}$
$k_{-1} = 0$ (assigned)
$k_{-2} = 0$ (assigned)
$k_{-3} = (8.82 \pm 11.88) \times 10^{-5}\ s^{-1}$

8.1.6. Original Data

Table S14. Original Data for the Thermodynamic Deprotonation of Pyridine with n-Butyl Sodium

**Time (sec)**	**3-*o*** (mM)	**3-*m*** (mM)	**3-*p*** (mM)	**4-*m*** (mM)	**4-*p*** (mM)
0	0	0	0	-	-
58	39	6	10	-	-
117	38	6	10	-	-
175	37	6	10	-	-
233	37	6	9	-	-
292	37	6	9	-	-
350	38	6	9	-	-
409	38	6	9	-	-
467	37	6	10	-	-
526	38	6	10	-	-
585	36	6	11	-	-
644	38	6	9	-	-
702	34	5	8	-	-
761	37	6	11	-	-
819	37	6	10	-	-
878	37	6	9	-	-
936	38	6	10	-	-
995	36	6	10	-	-
1053	37	6	10	-	-
1112	38	6	10	-	-
1171	39	6	8	-	-
1229	38	6	9	-	-
1288	38	6	10	-	-
1346	38	6	9	-	-
1405	38	6	9	-	-
1463	37	6	10	-	-
1522	36	6	10	-	-
1580	37	6	10	-	-
1639	36	6	10	-	-
1697	38	6	10	-	-
1756	38	6	10	-	-
1814	38	6	10	-	-
1873	46	13	25	-	-
1931	39	16	29	-	-
1990	35	17	35	-	-
2048	30	17	35	-	-
2107	26	21	43	-	-
2165	23	23	46	-	-
2224	20	21	44	-	-
2282	18	24	50	-	-
2341	16	22	48	-	-
2399	13	22	49	-	-

2459	12	22	49	-	-
2518	10	22	50	-	-
2577	9	22	51	-	-
2635	8	23	50	-	-
2694	7	23	52	-	-
2752	5	23	53	-	-
2811	5	21	49	-	-
2869	3	26	59	-	-
2928	4	25	57	-	-
2986	4	23	54	-	-
3045	2	26	59	-	-
3104	3	23	55	-	-
3163	3	22	57	-	-
3221	3	22	54	-	-
3280	5	21	54	-	-
3338	2	22	54	-	-
3397	3	22	55	-	-
3457	5	22	55	-	-
3515	2	22	54	-	-
3574	2	21	55	-	-
3633	2	21	56	-	-
3691	1	20	55	-	-
3750	5	21	55	-	-
3808	4	21	55	-	-
3867	2	20	54	-	-
3926	1	22	57	-	-
3984	1	21	55	-	-
4042	2	19	52	-	-
4101	1	21	55	-	-
4159	5	20	52	-	-
4218	1	21	55	-	-
4276	1	20	54	-	-
4336	1	22	57	-	-
4394	5	20	55	-	-
4453	3	19	54	-	-
4511	4	19	53	-	-
4570	1	19	54	-	-
4628	1	20	55	-	-
4687	2	19	53	-	-
4745	1	19	56	-	-
4804	1	21	57	-	-
4862	1	19	52	-	-
4921	1	19	53	-	-
4979	1	19	55	-	-
5038	1	19	55	-	-

5097	1	19	55	-	-
5156	1	18	55	-	-
5214	0	18	53	-	-
5272	1	20	56	-	-
5331	1	19	54	-	-
5390	1	18	52	-	-
5449	1	18	54	-	-
5507	1	18	55	-	-
5565	0	21	58	-	-
5624	1	19	56	-	-
5682	1	18	54	-	-
5741	1	18	56	-	-
5799	0	18	57	-	-
5858	1	19	57	-	-
5917	0	18	54	-	-
5975	0	18	57	-	-
6033	1	19	56	-	-
6092	0	18	55	-	-
6150	0	18	54	-	-
6209	1	19	56	-	-
6267	1	18	55	-	-
6326	4	17	54	-	-
6384	1	20	58	-	-
6444	0	17	54	-	-
6502	0	17	53	-	-
6561	1	18	54	-	-
6619	1	17	56	-	-
6678	0	18	55	-	-
6737	0	17	55	-	-
6795	1	17	53	-	-
6853	0	19	56	-	-
6912	2	20	58	-	-
6970	1	17	54	-	-
7028	1	17	53	-	-
7087	1	17	54	-	-
7145	1	16	51	-	-
7204	0	19	57	-	-
7263	0	17	54	-	-
7321	0	17	54	-	-
7380	3	16	53	-	-
7438	1	18	56	-	-
7496	0	20	58	-	-
7555	0	19	56	-	-
7613	1	18	55	-	-
7672	1	16	53	-	-

7730	1	16	52	-	-
7789	0	18	56	-	-
7848	1	17	53	-	-
7907	0	17	55	-	-
7965	0	19	57	-	-
8023	1	17	53	-	-
8083	0	18	56	-	-
8141	0	16	53	-	-
8200	0	19	56	-	-
8258	0	18	55	-	-
8317	1	17	53	-	-
8375	0	19	57	-	-
8433	0	17	53	-	-
8492	0	19	56	-	-
8550	1	16	53	-	-
8609	0	16	54	-	-
8667	1	19	57	-	-
8726	0	19	57	-	-
8785	1	17	54	-	-
8843	0	16	57	-	-
8902	1	16	52	-	-
8960	0	18	56	-	-
9018	0	17	52	-	-
9077	1	16	54	-	-
9135	1	17	55	-	-
9194	1	16	53	-	-
9252	1	16	53	-	-
9312	0	17	56	-	-
9370	1	18	56	-	-
9429	0	16	54	-	-
9488	0	16	54	-	-
9547	1	17	56	-	-
9605	0	16	54	-	-
9664	1	16	53	-	-
9722	0	19	58	-	-
9782	0	16	54	-	-
9840	1	16	54	-	-
9898	4	16	55	-	-
9957	5	16	55	-	-
10016	0	18	56	-	-
10075	3	9	5	13	37
10135	1	6	2	13	46
10193	2	6	4	15	45
10251	4	4	3	17	56
10310	1	3	3	16	54

10368	1	4	2	14	47
10427	1	3	3	17	59
10485	0	3	3	18	58
10544	1	2	2	15	52
10602	2	2	3	19	60
10661	1	4	3	18	56
10719	3	2	3	19	60
10778	2	1	3	19	61
10837	2	0	2	18	61
10895	0	2	2	17	58
10954	0	2	2	19	60
11013	0	3	2	18	57
11071	0	2	2	16	54
11129	0	3	2	18	56
11188	0	1	2	19	61
11247	0	3	3	18	57
11305	0	2	2	18	58
11364	0	1	2	19	62
11422	0	2	2	18	57
11481	0	0	1	17	61
11540	0	1	1	15	54
11598	0	1	2	17	61
11657	0	1	2	18	59

8.2. Kinetic Study of Ru-Catalyzed Ring-Opening Metathesis Polymerization

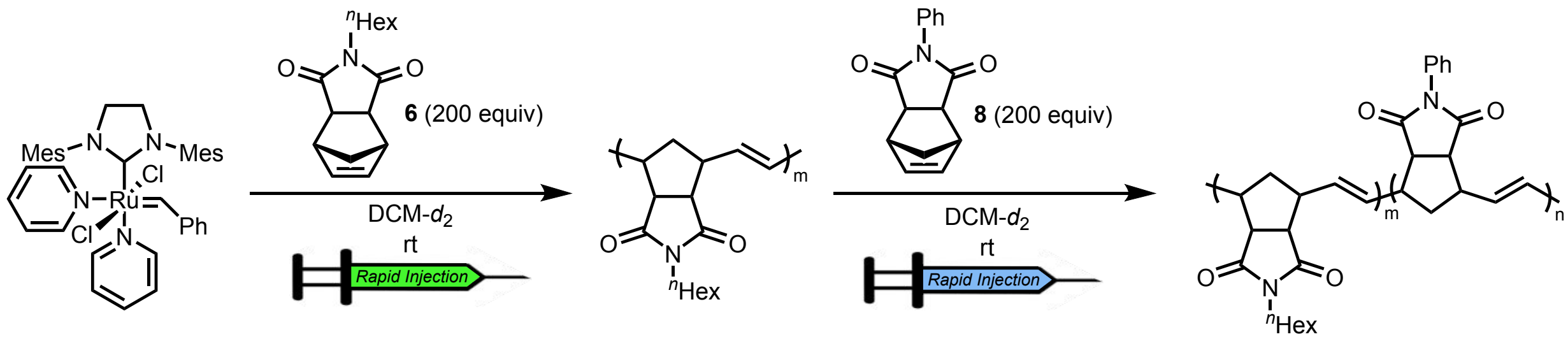

5 7 9

8.2.1. Experimental Procedure

The following stock solutions were freshly prepared for the reaction:

- A stock solution of **5** (0.25 mM) with 1,2,4,5-tetramethylbenzene was prepared by dissolving **5** (0.4 mg, 0.5 μmol) and 1,2,4,5-tetramethylbenzene (28.7 mg, 0.214 mmol) in freshly distilled (CaH_2) dichloromethane-d_2 in an argon-purged 2.0 mL volumetric flask.
- A stock solution of **6** (0.50 M) was prepared by diluting **6** (123.7 mg, 0.50 mmol) in freshly distilled (CaH_2) dichloromethane-d_2 in a 1.0 mL volumetric flask. The solution was loaded into injector A.
- A stock solution of **8** (0.50 M) was prepared by diluting **8** (119.6 mg, 0.50 mmol) in freshly distilled (CaH_2) dichloromethane-d_2 in a 1.0 mL volumetric flask. The solution was loaded into injector B.

The freshly prepared stock solution of **5** (500 μL, 0.125 μmol [Ru], 1.0 equiv) was transferred to an argon-purged NMR tube fitted with a rubber septum, and the tube was placed into a N_2-purged NMR spectrometer maintained at 25 °C. **6** was first injected with injector A (50 μL, 25.0 μmol, 200 equiv) while the sample was being monitored by ^{1}H NMR. After the full consumption of **6** is confirmed by ^{1}H NMR, the sample was briefly reshimmed and **8** was injected with injector B (50 μL, 25.0 μmol, 200 equiv) following the same data acquisition method. After the full consumption of **8** is confirmed by ^{1}H NMR, the tube was removed from the instrument and excess ethyl vinyl ether (50 μL, 526 μmol, 4210 equiv) was added to the sample. The mixture was poured into hexanes (10 mL), and the solid precipitate was collected with a centrifuge (9000 rpm, 5 min), washed with hexanes (2 × 10 mL) and analyzed by SEC (DMF/LiBr, calibrated to polystyrene standards).

8.2.2. Peak Identification

The signals were compared to ^{1}H spectra of purified monomers **6** and **8**. The olefin peaks (**6**: δ6.22 ppm; **8**: δ6.38 ppm) were monitored with internal standard (1,2,4,5-tetramethylbenzene, δ6.90 ppm, 2H) to track the consumption of both monomers. Degree of polymerization (DP) was estimated by integration of the olefin peaks of the polymer (δ5.92-5.45 ppm) of the last ^{1}H spectrum after injection of **8**.

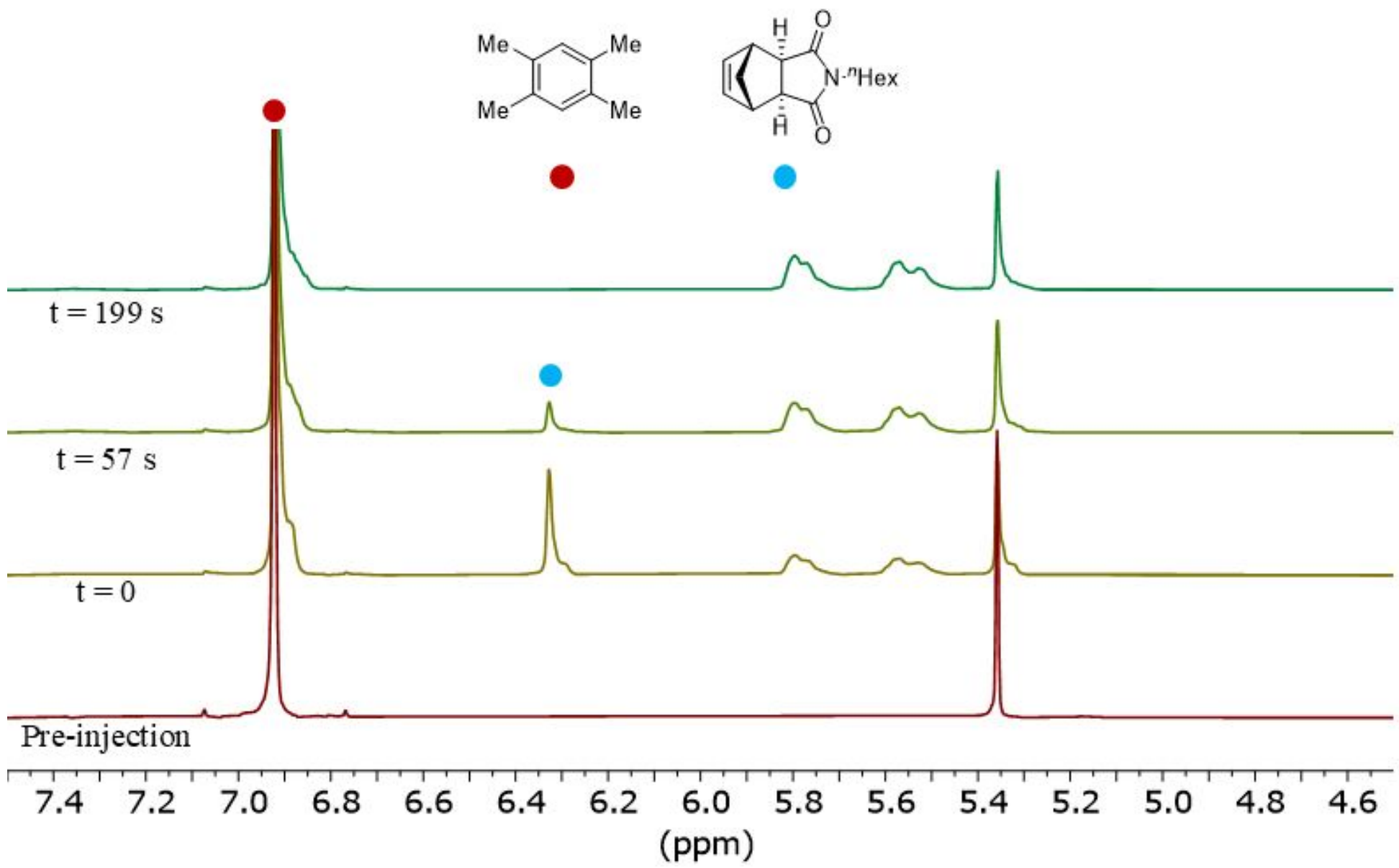

Figure S44. Reaction profile of **6**.

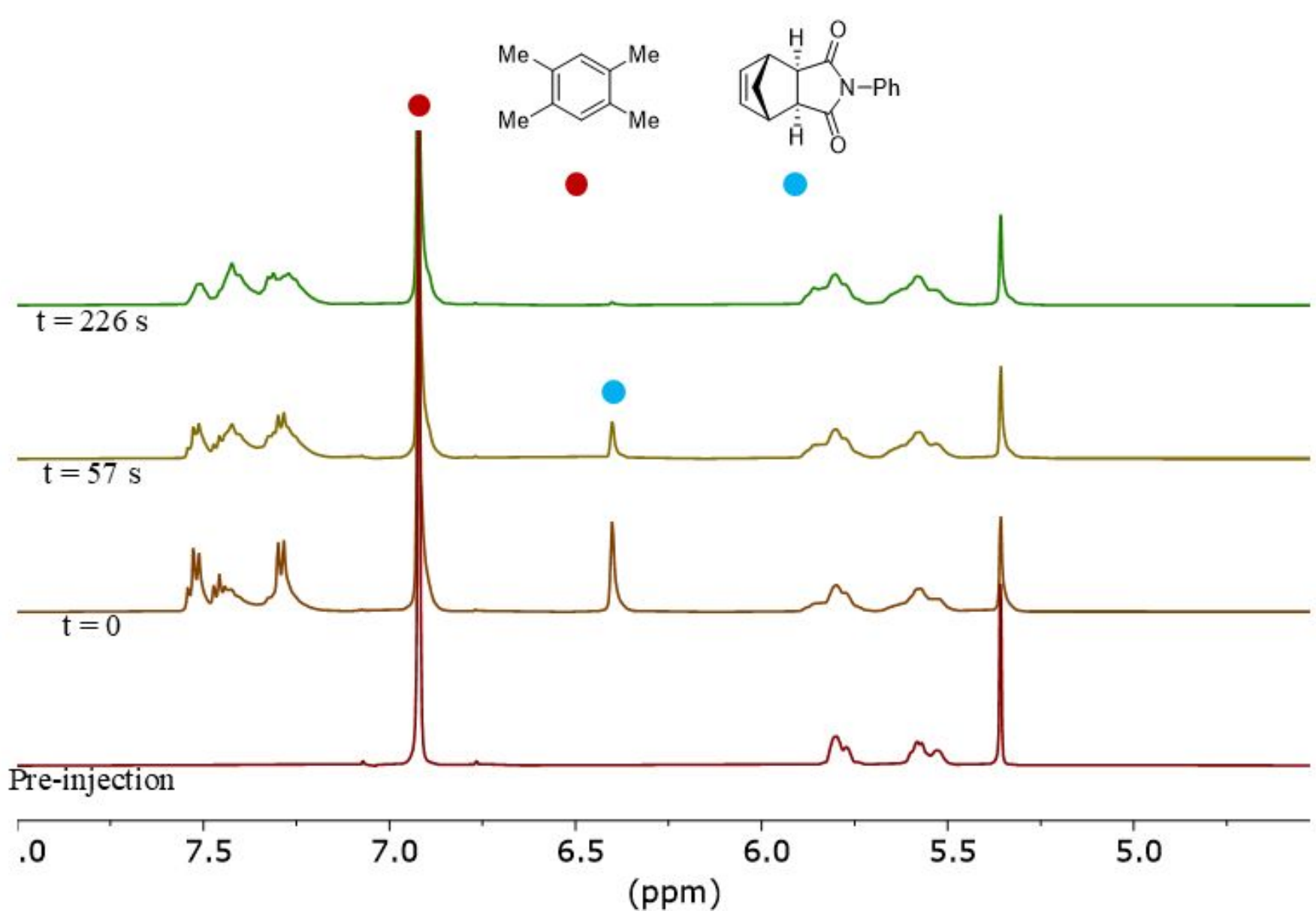

Figure S45. Reaction profile of **8**.

8.2.3. Data Acquisition

Prior to injection, a spectrum was acquired with the following parameters to determine the actual catalyst concentration.

Pulse angle: 30 degrees
Number of scans (NS) = 4
Spectral center (o1p) = 6.18 ppm
Spectral width (sw) = 29.40 ppm
Acquisition time = 2.23 sec
Relaxation delay (d1) = 30 sec

During the injection, the sample was monitored with Bruker multizg program with the following parameters.

Pulse angle: 30 degrees
Number of scans (NS) = 1
Spectral center (o1p) = 6.18 ppm
Spectral width (sw) = 19.22 ppm
Acquisition time = 3.41 sec
Relaxation delay (d1) = 20 sec (ca 29 sec between each scan)

8.2.4. Data Analysis

Concentration was calculated by integration of the olefin peaks relative to the internal standard and the total volume after injection (549 μL and 598 μL after **6** and **8** injection, respectively). The logarithm plot of the relative monomer concentration ($\ln([M]/[M]_0) - t$) was generated with the beginning of the linear region defined as $t = 0$ and $[M] = [M]_0$. The rate constants were determined by the absolute values of the slopes of linear region lines. The average and 95% confidence interval bounds of three runs were calculated with inverse-variance weighting method.

8.2.5. Experiment Results

Run 1

(a)

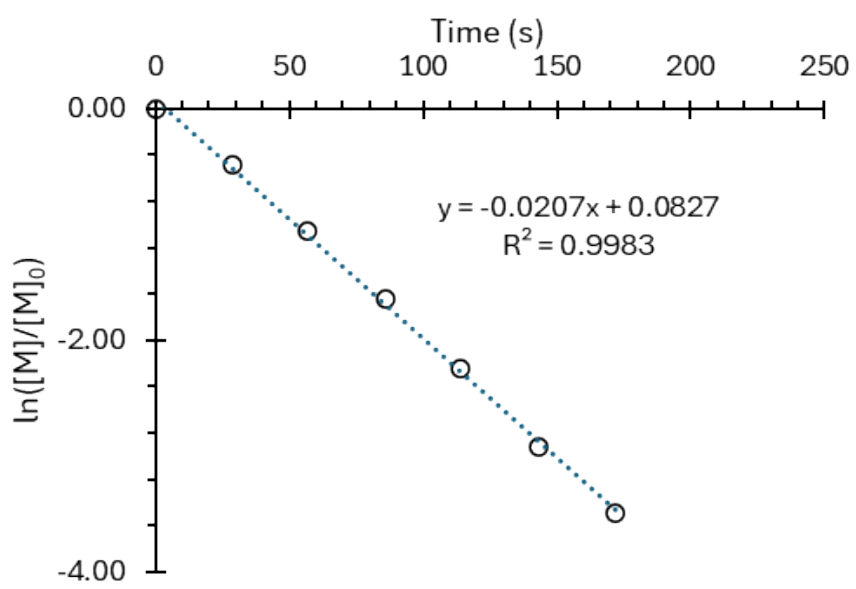

(b)

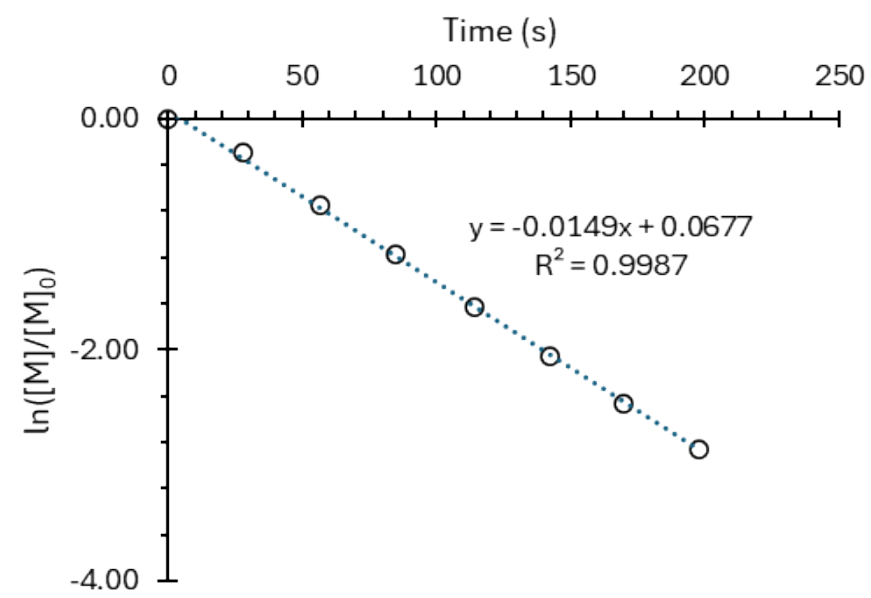

Figure S46. (a) logarithm plot of **6** consumption. (b) logarithm plot of **8** consumption

Catalyst loading of **5** was determined to be 0.124 μmol by integration of ^{1}H NMR prior to injection.

Expected properties

DP: 403

M_n^{theo} : 98.1 kDa

(calculated with catalyst loading, assuming 25.0 μmol for each monomer injection)

Characterization of polymer product

DP_{NMR}: 444

DP_{SEC}: 334 (from average of monomer weights)

M_n: 80.6 kDa (SEC)

M_w: 104 kDa (SEC)

PDI: 1.23 (Mw / Mn)

Propagation rate constants with 95% confidence intervals

$k_1 = (2.07 \pm 0.097) \times 10^{-2}\ s^{-1}$ (n = 7)

$k_2 = (1.49 \pm 0.053) \times 10^{-2}\ s^{-1}$ (n = 8)

SEC graph

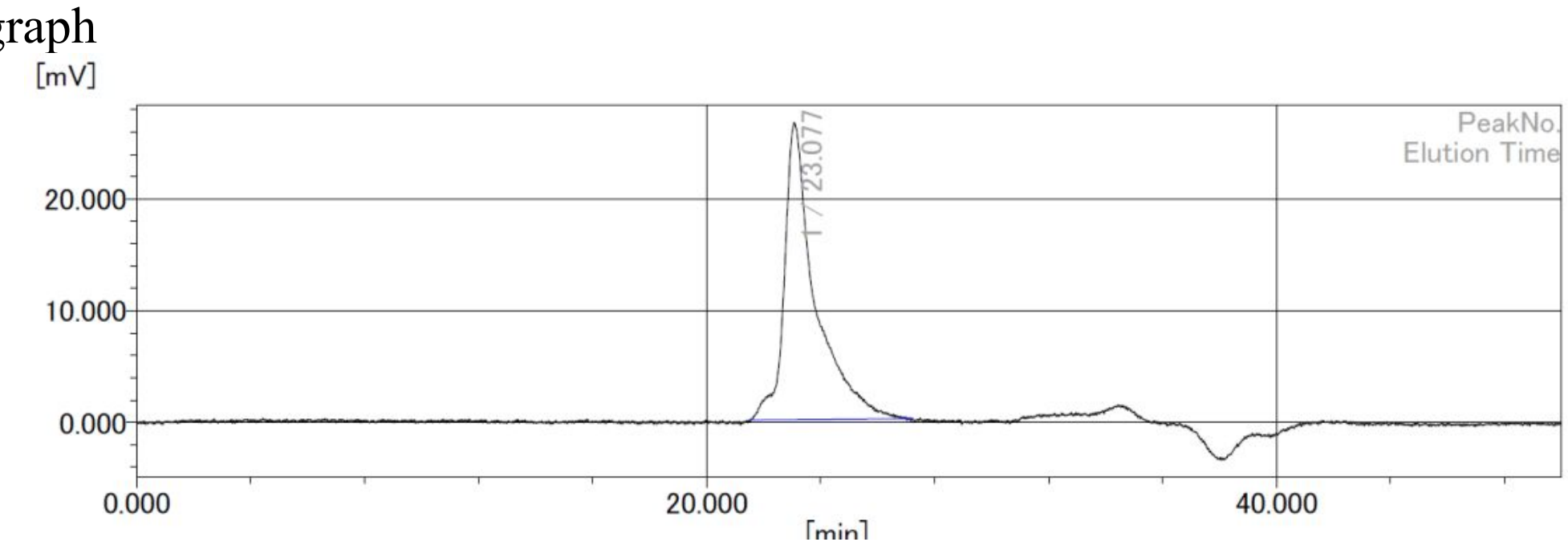

Run 2

(a) (b)

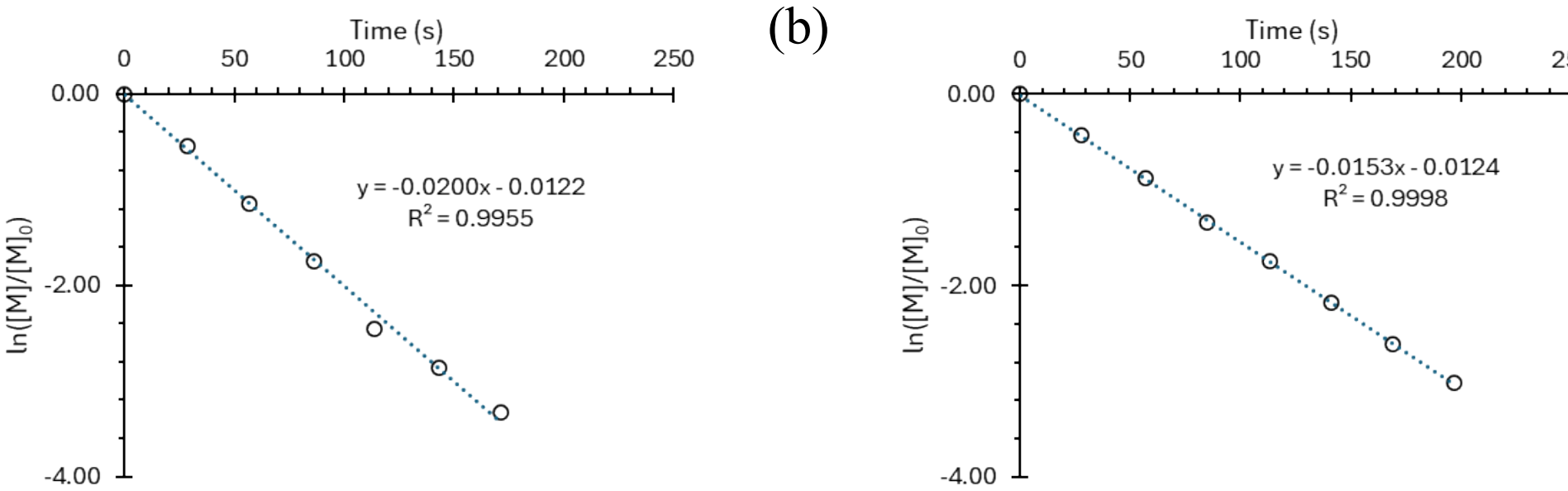

Figure S47. (a) logarithm plot of **6** consumption. (b) logarithm plot of **8** consumption

Catalyst loading of **5** was determined to be 0.0951 μmol by integration of ^{1}H NMR prior to injection.

Expected properties

DP: 525

M_n^{theo} :128 kDa

(calculated with catalyst loading, assuming 25.0 μmol for each monomer injection)

Characterization of polymer product

DP_{NMR}: 461

DP_{SEC}: 298 (from average of monomer weights)

M_n: 72.6 kDa (SEC)

M_w: 88.5 kDa (SEC)

PDI: 1.22 (M_w / M_n)

Propagation rate constants with 95% confidence intervals

$k_1 = (2.00 \pm 0.016) \times 10^{-2}$ s^{-1} (n = 7)

$k_2 = (1.53 \pm 0.020) \times 10^{-2}$ s^{-1} (n = 8)

SEC graph

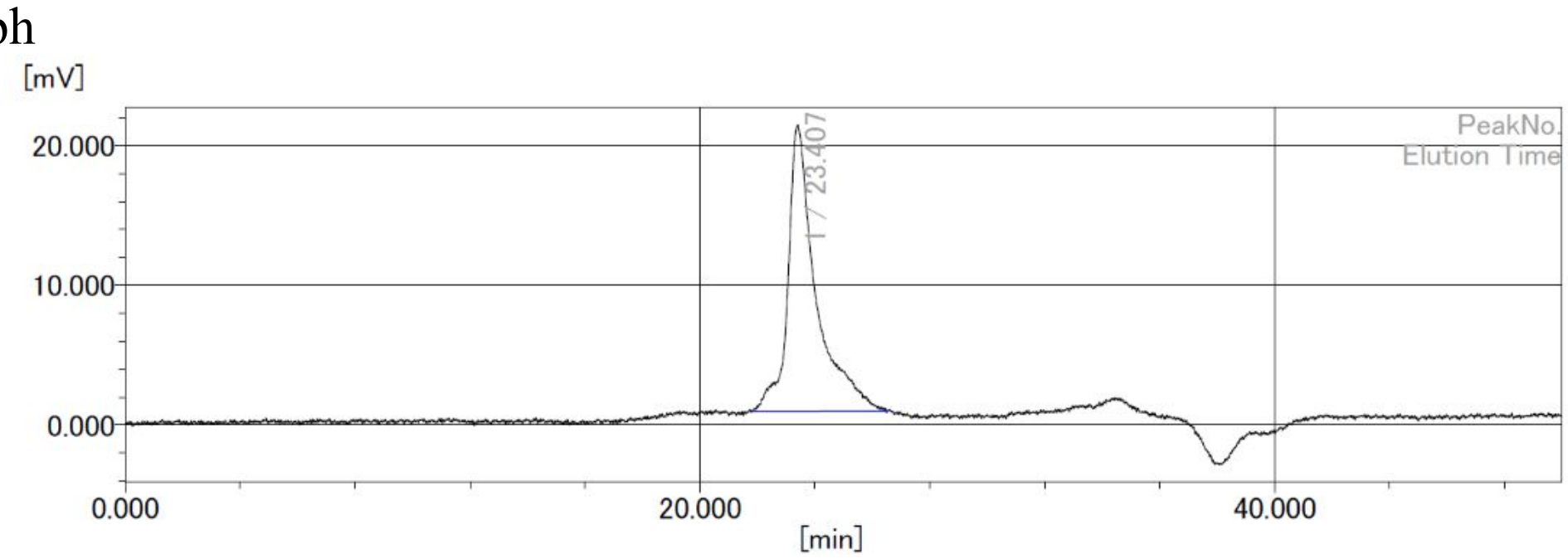

Run 3

(a)

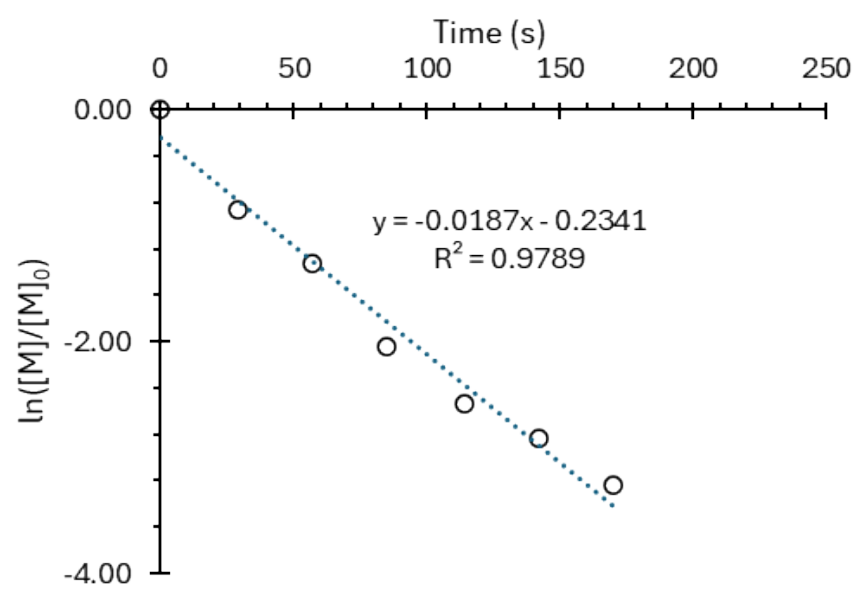

(b)

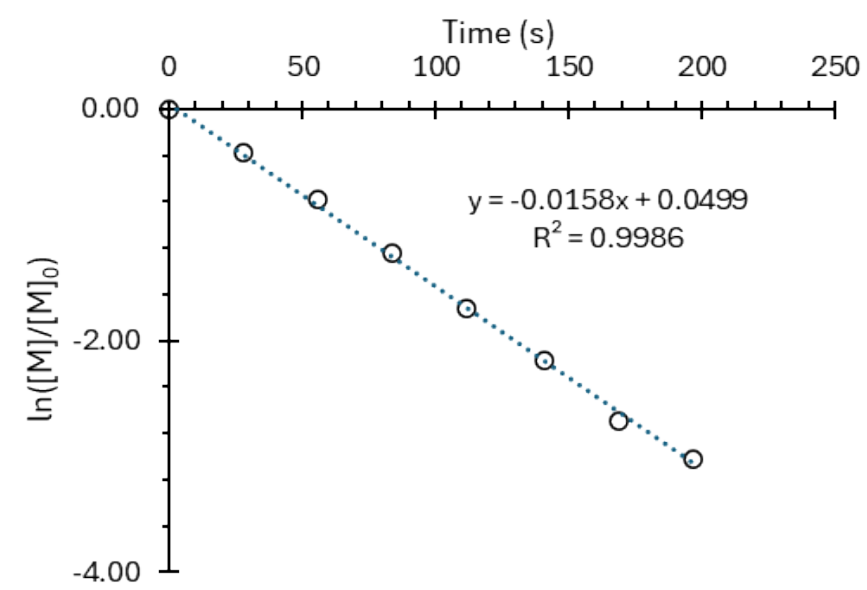

Figure S48. (a) logarithm plot of **6** consumption. (b) logarithm plot of **8** consumption

Catalyst loading of **5** was determined to be 0.119 μmol by integration of ^{1}H NMR prior to injection.

Expected properties

DP: 420

M_n^{theo} :102 kDa

(calculated with catalyst loading, assuming 25.0 μmol for each monomer injection)

Characterization of polymer product

DP_{NMR}: 558

DP_{SEC}: 356 (from average of monomer weights)

Mn: 86.6 kDa (SEC)

Mw: 111 kDa (SEC)

PDI: 1.28 (Mw / Mn)

Propagation rate constants with 95% confidence intervals

$k_1 = (1.87 \pm 0.320) \times 10^{-2}$ s^{-1} (n = 7)

$k_2 = (1.58 \pm 0.056) \times 10^{-2}$ s^{-1} (n = 8)

SEC graph

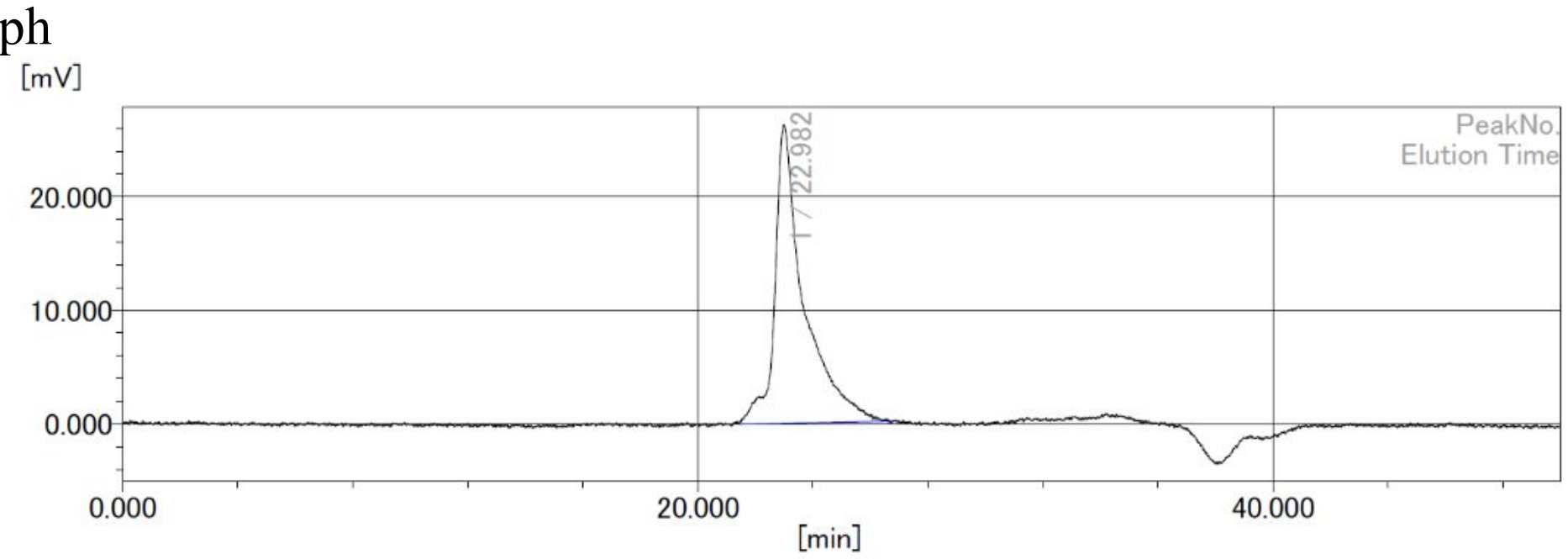

8.2.6. Original Data

Table S15. Original data for run 1 (linear regions for the logarithm plot was highlighted)

Time (s)	**6** (M)	ln([M]/[M]$_0$)
**0**	0.0366	**0.00**
**29**	0.0225	**-0.48**
**57**	0.0127	**-1.05**
**86**	0.0070	**-1.65**
**114**	0.0039	**-2.24**
**143**	0.0020	**-2.93**
**172**	0.0011	**-3.50**
200	0.0006	-4.04
228	0.0004	-4.43
256	0.0003	-4.98
285	0.0001	-6.11
313	0.0001	-6.31
342	0.0001	-6.14
370	0.0001	-6.57
398	0.0000	-7.96
426	0.0000	-6.93
454	0.0000	-
482	0.0000	-7.27
511	0.0000	-6.68
539	0.0000	-
567	-0.0001	-
595	0.0000	-7.96
624	0.0000	-
652	0.0000	-9.88

Time (s)	**8** (M)	ln([M]/[M]$_0$)
**0**	0.0309	**0.00**
**28**	0.0231	**-0.29**
**57**	0.0147	**-0.75**
**85**	0.0095	**-1.18**
**114**	0.0060	**-1.64**
**142**	0.0040	**-2.06**
**170**	0.0026	**-2.47**
**198**	0.0018	**-2.87**
226	0.0012	-3.26
254	0.0008	-3.71
282	0.0006	-3.98
310	0.0004	-4.24
338	0.0003	-4.51
367	0.0002	-4.95
395	0.0002	-5.28
423	0.0002	-5.32
451	0.0001	-5.38
479	0.0000	-6.68
507	0.0001	-6.02
535	0.0000	-6.55
563	0.0001	-6.12
591	0.0001	-6.23
619	0.0001	-6.09
647	0.0001	-5.95
675	0.0000	-7.32
703	0.0001	-6.30
732	0.0001	-5.76

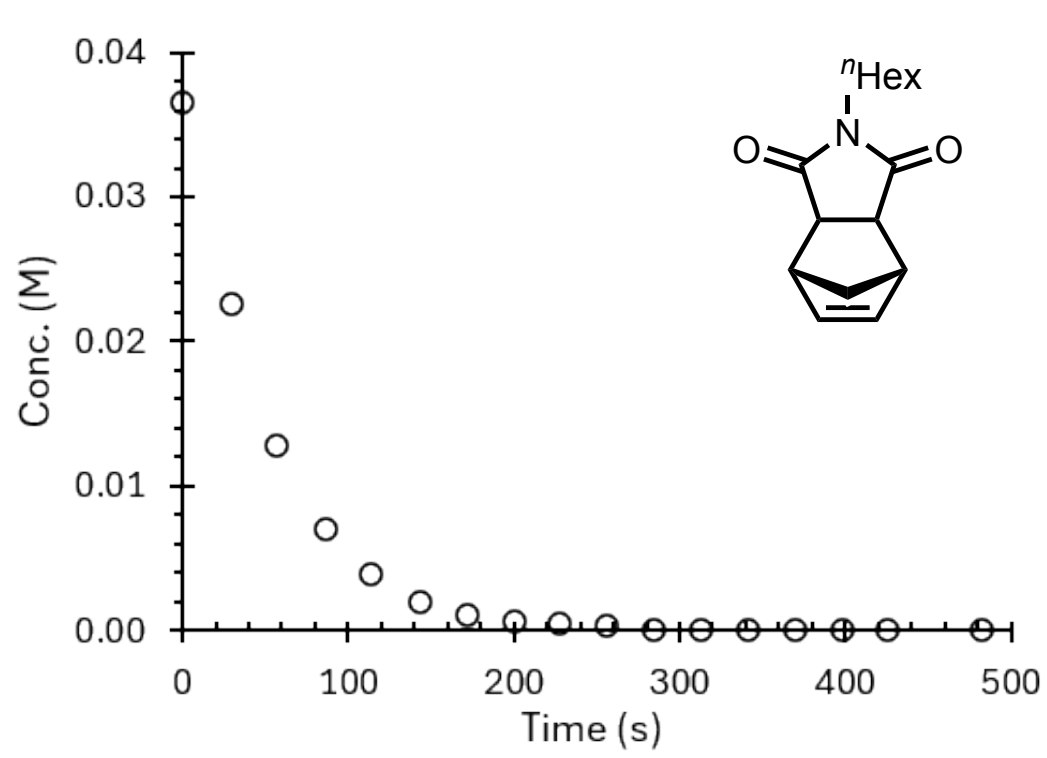

Figure S49. Ru-catalyzed ROMP of **6**, run 1.

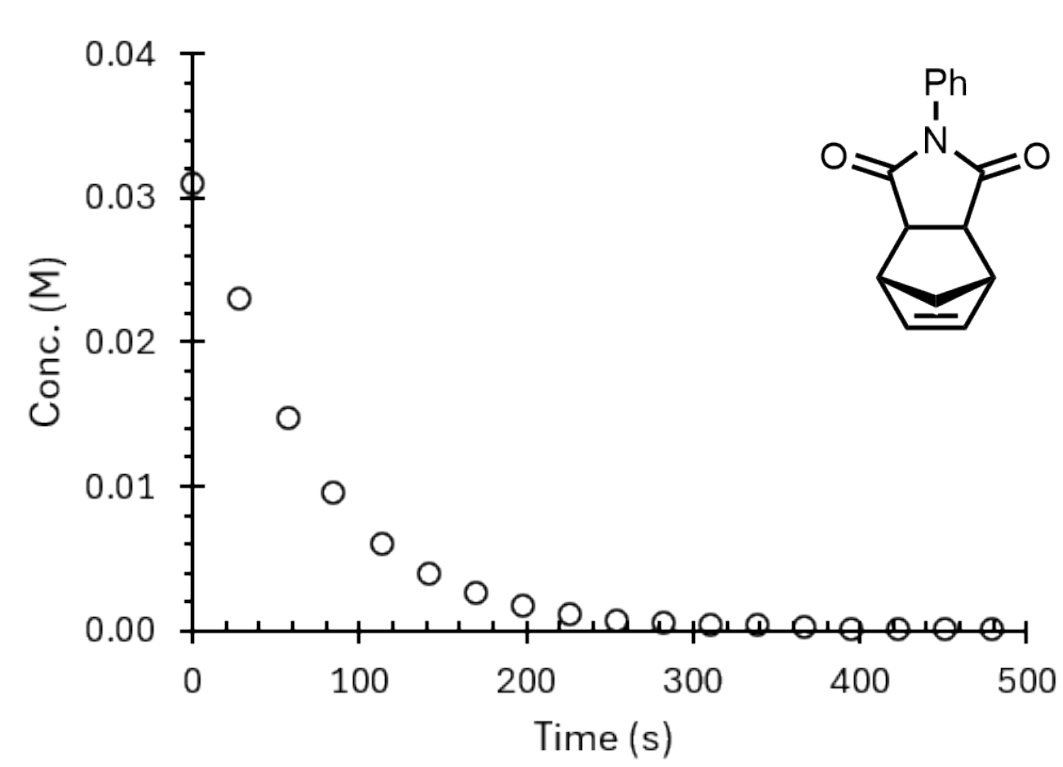

Figure S50. Ru-catalyzed ROMP of **8**, run 1.

Table S16. Original data for run 2 (linear regions for the logarithm plot was highlighted)

Time (s)	**6** (M)	ln([M]/[M]$_0$)	Time (s)	**8** (M)	ln([M]/[M]$_0$)
**0**	0.0202	**0.00**	**0**	0.0298	**0.00**
**29**	0.0118	**-0.54**	**28**	0.0192	**-0.44**
**57**	0.0064	**-1.14**	**57**	0.0123	**-0.88**
**86**	0.0035	**-1.75**	**85**	0.0078	**-1.34**
**114**	0.0017	**-2.46**	**113**	0.0052	**-1.75**
**143**	0.0012	**-2.86**	**141**	0.0034	**-2.18**
**171**	0.0007	**-3.33**	**169**	0.0022	**-2.61**
199	0.0002	-4.65	**197**	0.0015	**-3.01**
227	0.0002	-4.68	225	0.0009	-3.46
255	0.0002	-4.60	253	0.0007	-3.70
284	0.0000	-6.12	282	0.0005	-4.10
312	0.0001	-5.75	310	0.0004	-4.40
341	0.0000	-6.35	339	0.0003	-4.60
370	-0.0002	-	367	0.0003	-4.63
398	0.0001	-5.73	397	0.0002	-5.09
426	-0.0002	-	425	0.0002	-5.14
454	0.0000	-	453	0.0000	-6.83
482	-0.0001	-	482	0.0001	-5.44
510	0.0001	-5.88	511	0.0000	-6.49

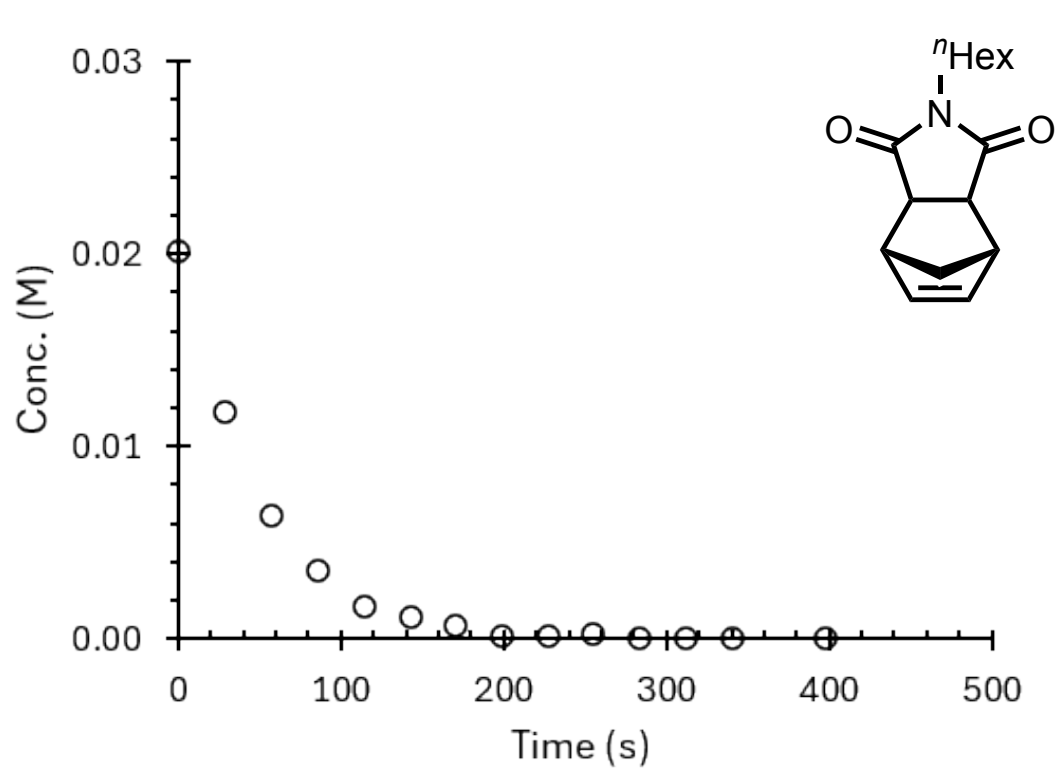

Figure S51. Ru-catalyzed ROMP of **6**, run 2.

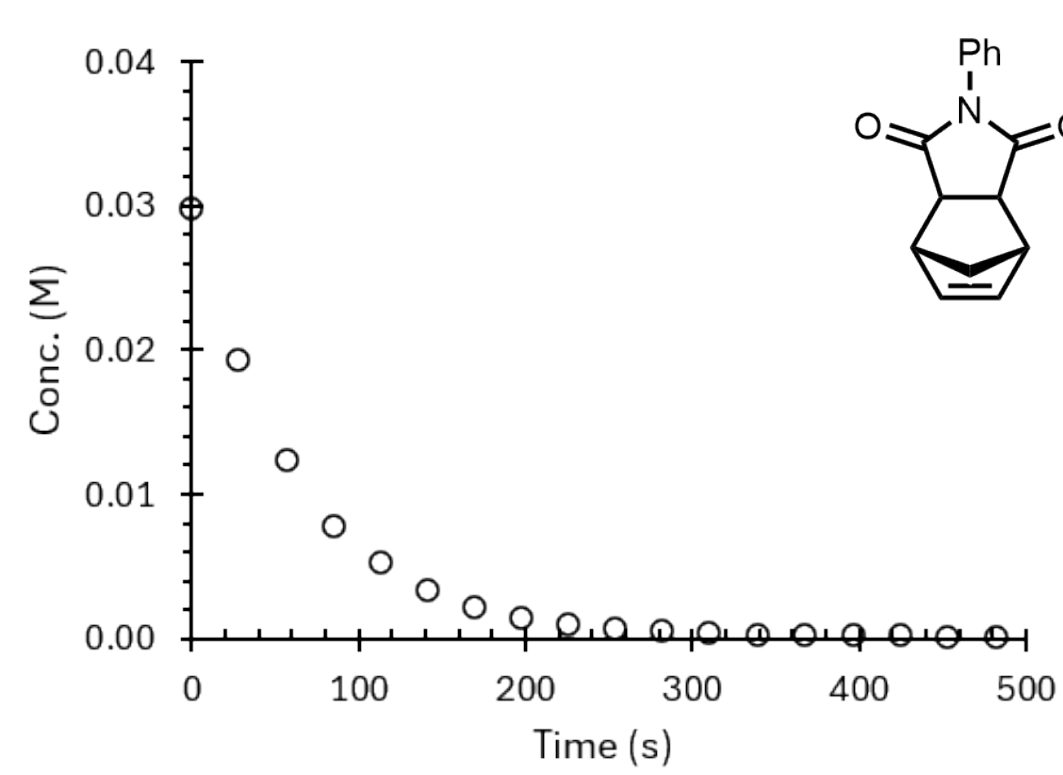

Figure S52. Ru-catalyzed ROMP of **8**, run 2.

Table S17. Original data for run 3 (linear regions for the logarithm plot was highlighted)

Time (s)	**6** (M)	ln([M]/[M]$_0$)
**0**	0.0640	**0.00**
**29**	0.0269	**-0.87**
**57**	0.0171	**-1.32**
**85**	0.0084	**-2.03**
**114**	0.0051	**-2.53**
**142**	0.0038	**-2.83**
**170**	0.0025	**-3.24**
198	0.0001	-6.31
226	0.0000	-
254	-0.0001	-
282	-0.0001	-
310	-0.0002	-
339	-0.0002	-
368	-0.0005	-
396	-0.0003	-

Time (s)	**8** (M)	ln([M]/[M]$_0$)
**0**	0.0352	**0.00**
**28**	0.0241	**-0.38**
**56**	0.0160	**-0.79**
**84**	0.0101	**-1.25**
**112**	0.0063	**-1.73**
**141**	0.0040	**-2.17**
**169**	0.0024	**-2.69**
**197**	0.0017	**-3.03**
225	0.0018	-2.99
253	0.0008	-3.85
281	0.0005	-4.17
309	-0.0003	-
337	-0.0006	-

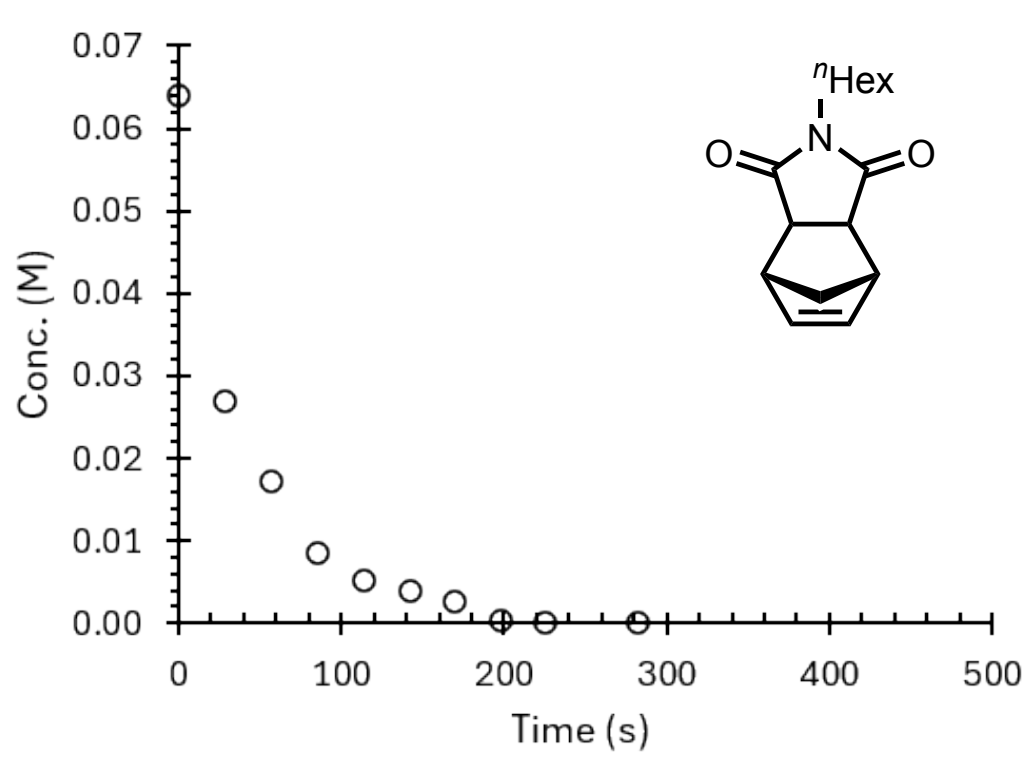

Figure S53. Ru-catalyzed ROMP of **6**, run 3.

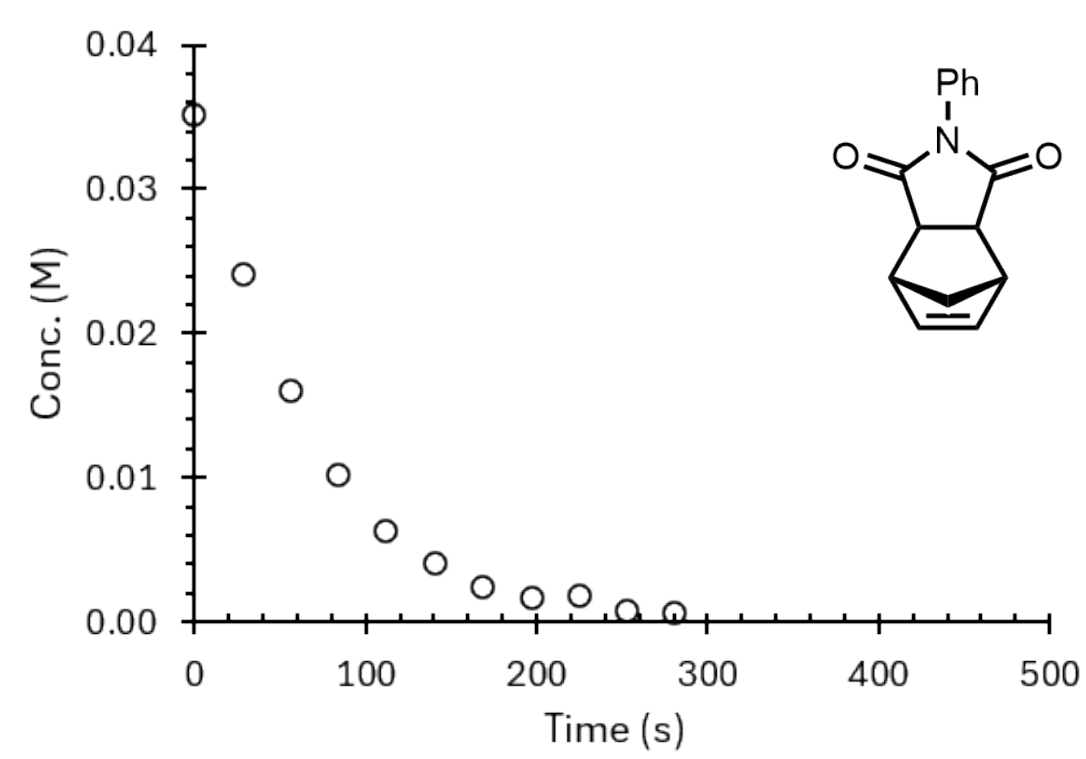

Figure S54. Ru-catalyzed ROMP of **8**, run 3.

8.2.7. Synthesis of Monomers.

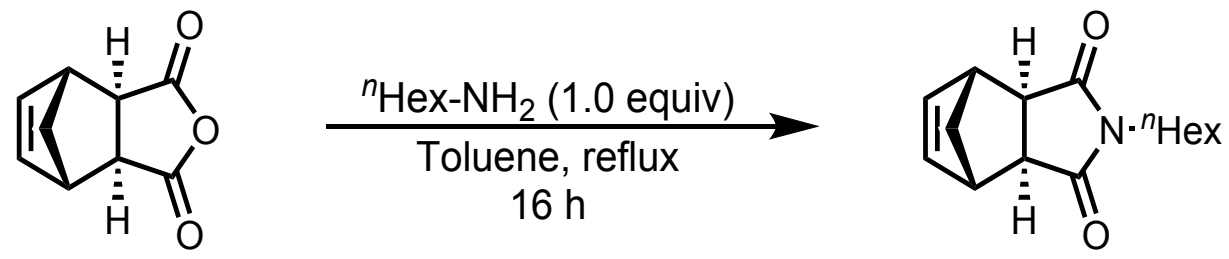

6

Cis-*N*-*n*-hexyl-norbornene-*exo*-2,3-dicarboximide **6** was synthesized according to a literature procedure. *cis*-5-norbornene-*exo*-2,3-dicarboxylic anhydride (3.28 g, 20.0 mmol, 1.0 equiv) and hexylamine (2.63 mL, 20.0 mmol, 1.0 equiv) was added to a 250-mL round bottom flask and purged with Ar. 90 mL of toluene was added and the reaction flask was fitted with a Dean-Stark trap and a condenser. The reaction was heated to reflux for 16 h. After the completion of the reaction verified by GC-MS, the crude mixture was concentrated *in vacuo* and purified by column chromatography on silica gel (10% to 30% EtOAc in hexanes), followed by two fractional distillation under vacuum (1.0 torr, 200 °C) discarding the initial (ca 2%) of the distillates to afford *cis*-*N*-*n*-hexyl-norbornene-*exo*-2,3-dicarboximide **6** (4.53 g, 91.5%). ^{1}H NMR is in correspondence to the reported literature.[3]

^{1}H NMR (400 MHz, $CDCl_3$) δ 6.28-6.24 (m, 2H), 3.43 (dd, *J* = 6.13, 7.36 Hz, 2H), 3.27-3.24 (m, 2H), 2.65 (d, *J* = 1.4 Hz, 2H), 1.58-1.45 (m, 3H), 1.35-1.17 (m, 7H), 0.85 (t, *J* = 6.5 Hz, 3H).
^{13}C NMR (101 MHz, $CDCl_3$) δ 178.2, 137.9, 47.9, 45.3, 42.8, 38.9, 31.4, 27.8, 26.72, 22.6, 14.1.

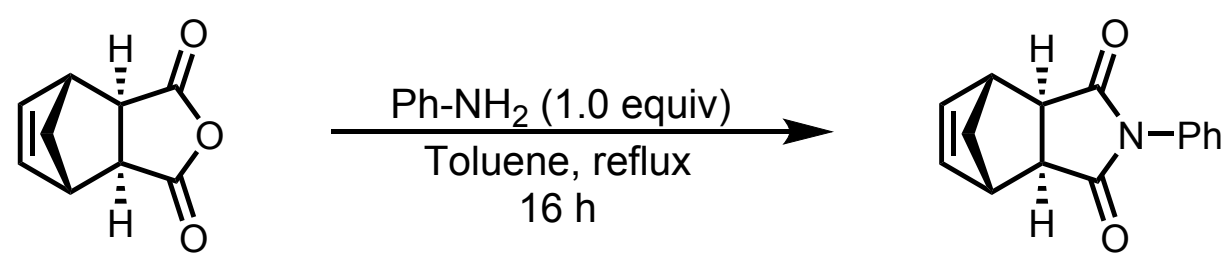

8

Cis-*N*-phenyl-norbornene-*exo*-2,3-dicarboximide **8** was synthesized according to a modified literature procedure.[3] *Cis*-5-norbornene-*exo*-2,3-dicarboxylic anhydride (3.28 g, 20.0 mmol, 1.0 equiv) and aniline (1.81 mL, 20.0 mmol, 1.0 equiv) was added to a 250-mL round bottom flask and purged with Ar. 90 mL of toluene was added and the reaction flask was fitted with a Dean-Stark trap and a condenser. The reaction was heated to reflux for 16 h. After the completion of the reaction verified by GC-MS, the crude mixture was concentrated *in vacuo* and recrystallized two times in benzene to afford *cis*-*N*-phenyl-norbornene-*exo*-2,3-dicarboximide **8** (1.88 g, 39.2%). ^{1}H NMR is in correspondence to the reported literature.[4]

^{1}H NMR (400 MHz, $CDCl_3$) δ 7.53-7.45 (m, 2H), 7.44-7.38 (m, 1H), 7.31-7.26 (m, 2H), 6.39-6.35 (m, 2H), 3.45-3.41 (m, 2H), 2.88 (d, *J* = 0.97 Hz, 2H), 1.68 (dt, *J* = 9.93, 1.36 Hz, 1H), 1.52 (d, *J* = 9.93, 1H).
^{13}C NMR (101 MHz, $CDCl_3$) δ 177.2, 138.2, 132.0, 129.3, 128.8, 126.5, 48.0, 46.0, 43.1.

8.2.8. T_1 Measurements of Monomers.

A standard ^{1}H NMR inversion-recovery sequence (180° - τ - 90°) was employed to measure the spin-lattice relaxation time constant (T_1). The spectral center was moved to the olefin signal for data collection, and the peak area was plotted against time and fitted to the exponential decay model:

$$\mathrm{M} = \mathrm{M}_0(1 - 2e^{\tau/T_1})$$

Exo-N-*n*-hexyl-norbornene-2,3-dicarboxamide (**6**)

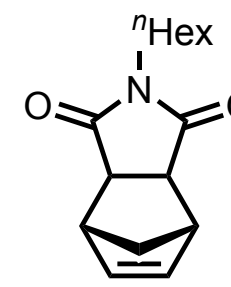

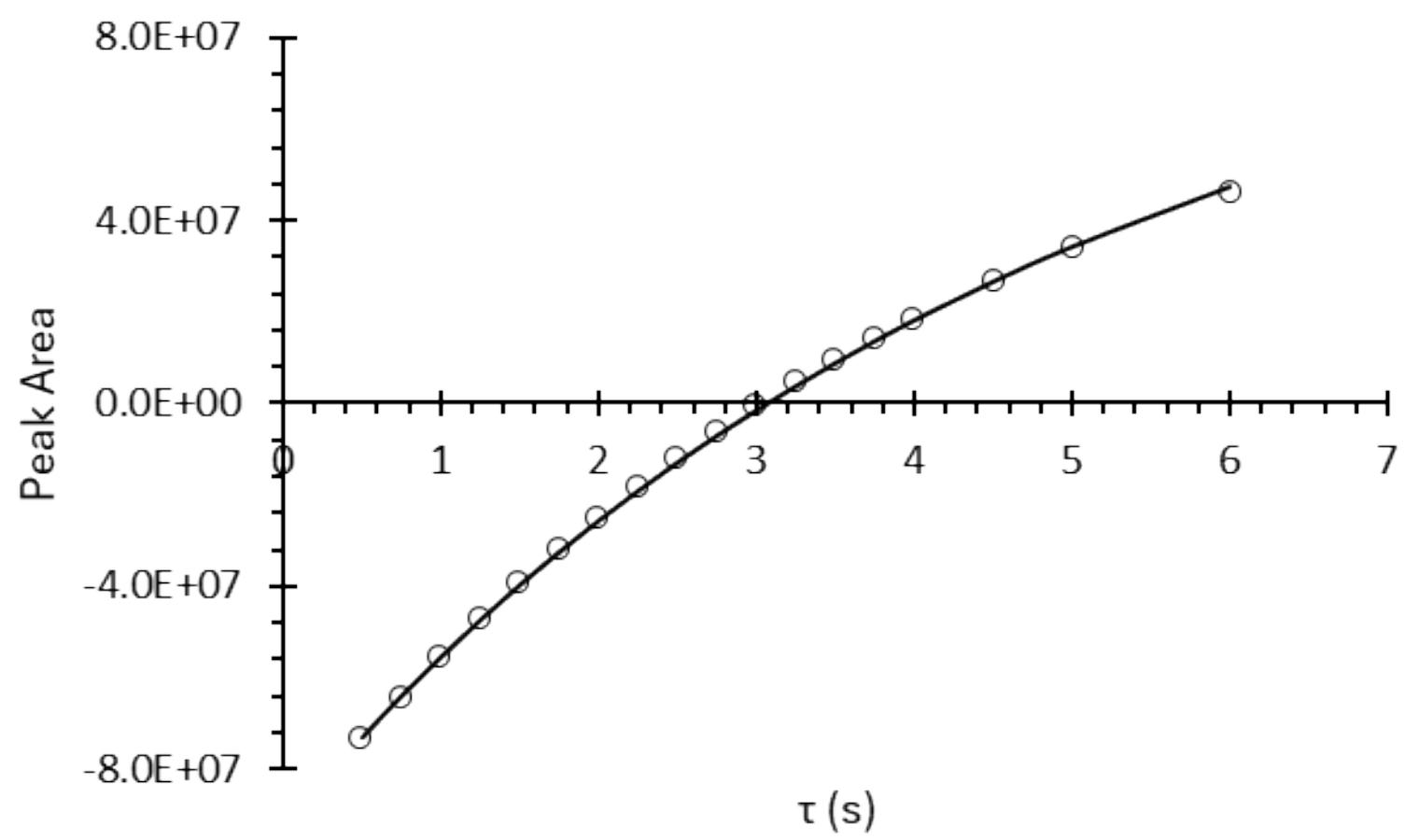

Figure S55. Inversion-recovery plot for monomer **6**.

Fitting Results

Sample Preparation

20.7 mg of **6** dissolved in 500 μL dichloromethane-d_2 (0.17 M).

Acquisition parameters

Number of scans (NS) = 4

Spectral center o1p = 6.32 ppm

Spectral width sw = 19.22 ppm

Acquisition time = 0.652 sec

Relaxation delay (d1) = 25 sec

Fitting parameters

Function f(t) = M0*(1-2*exp(-t/T1))

Fitting results in 95% confidence bonds (R^2 = 0.999)

T_1 = 4.38 ± 0.021 (sec)

M_0 = (9.38 ± 0.072) × 10^7

Exo-N-phenyl-norbornene-2,3-dicarboximide (**8**)

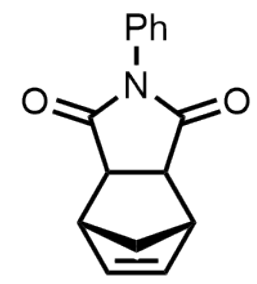

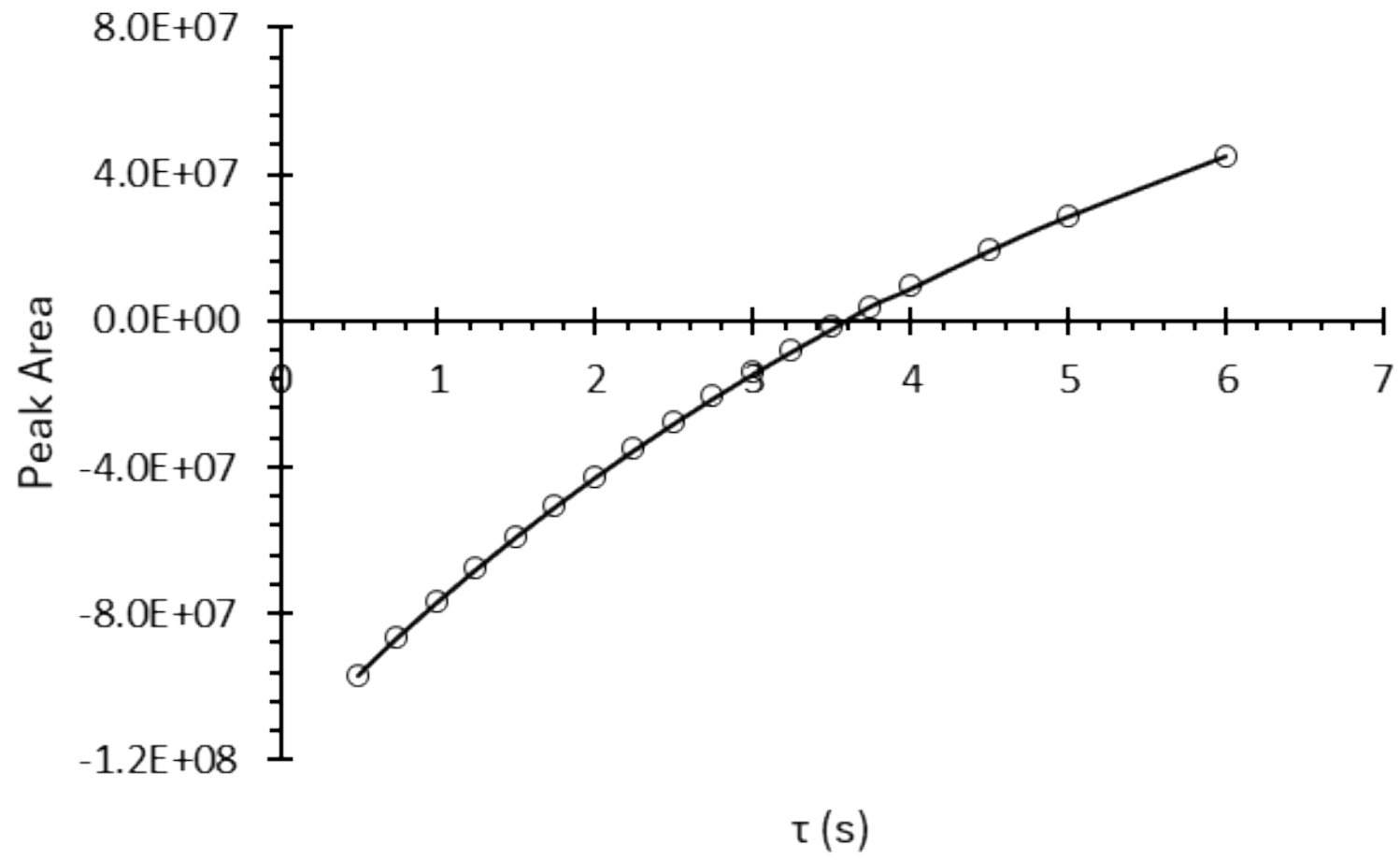

Figure S56. Inversion-recovery plot for monomer **8**.

Fitting Results

Sample Preparation

21.0 mg of **8** dissolved in 500 µL dichloromethane-d_2 (0.18 M).

Acquisition parameters

Number of scans (NS) = 4

Spectral center (o1p) = 6.40 ppm

Spectral width (sw) = 19.22 ppm

Acquisition time (AQ) = 0.652 sec

Relaxation delay (d1) = 25 sec

Fitting parameters

Function f(t) = M0*(1-2*exp(-t/T1))

Fitting results in 95% confidence bonds (R^2 = 0.999)

$T_1 = 5.18 \pm 0.012$ (sec)

$M_0 = (1.19 \pm 0.0041) \times 10^8$

8.3. Kinetic Study of Brønsted Base-Catalyzed Isomerization of Glucose

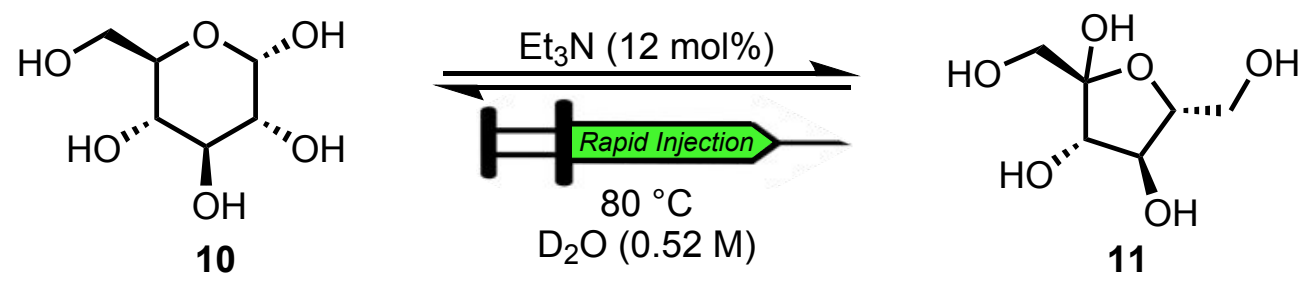

8.3.1. Experimental Procedure

Triethylamine stock solution (0.29 M) was prepared by dissolving triethylamine (80 µL, 0.58 mmol) in D_2O in a 2.0 mL volumetric flask. The solution was loaded into the injector syringe.

D-glucose **10** (46.8 mg, 0.26 mmol, 1.0 equiv) was weighed into a 5 mm NMR tube and dissolved in D_2O (400 µL). The sample tube was placed into the NMR spectrometer set at 80 °C and equilibrated for at least 10 minutes. Triethylamine solution (108 µL) was injected while the sample was monitored by ^1H NMR.

8.3.2. Peak Identification

The signals were compared to ^1H spectra of standard D-glucose and D-fructose in D_2O. The anomeric C-H of α-glucopyranose (δ5.66 ppm, ca 40% isomeric ratio of glucose[5]) was monitored for reaction progression. After injection, the catalyst loading was determined by comparing the relative integration of α-glucopyranose (δ5.66 ppm, 0.40 H) to the methyl peak ((C**H₃**CH₂)₃N, δ1.53 ppm, 9 H).

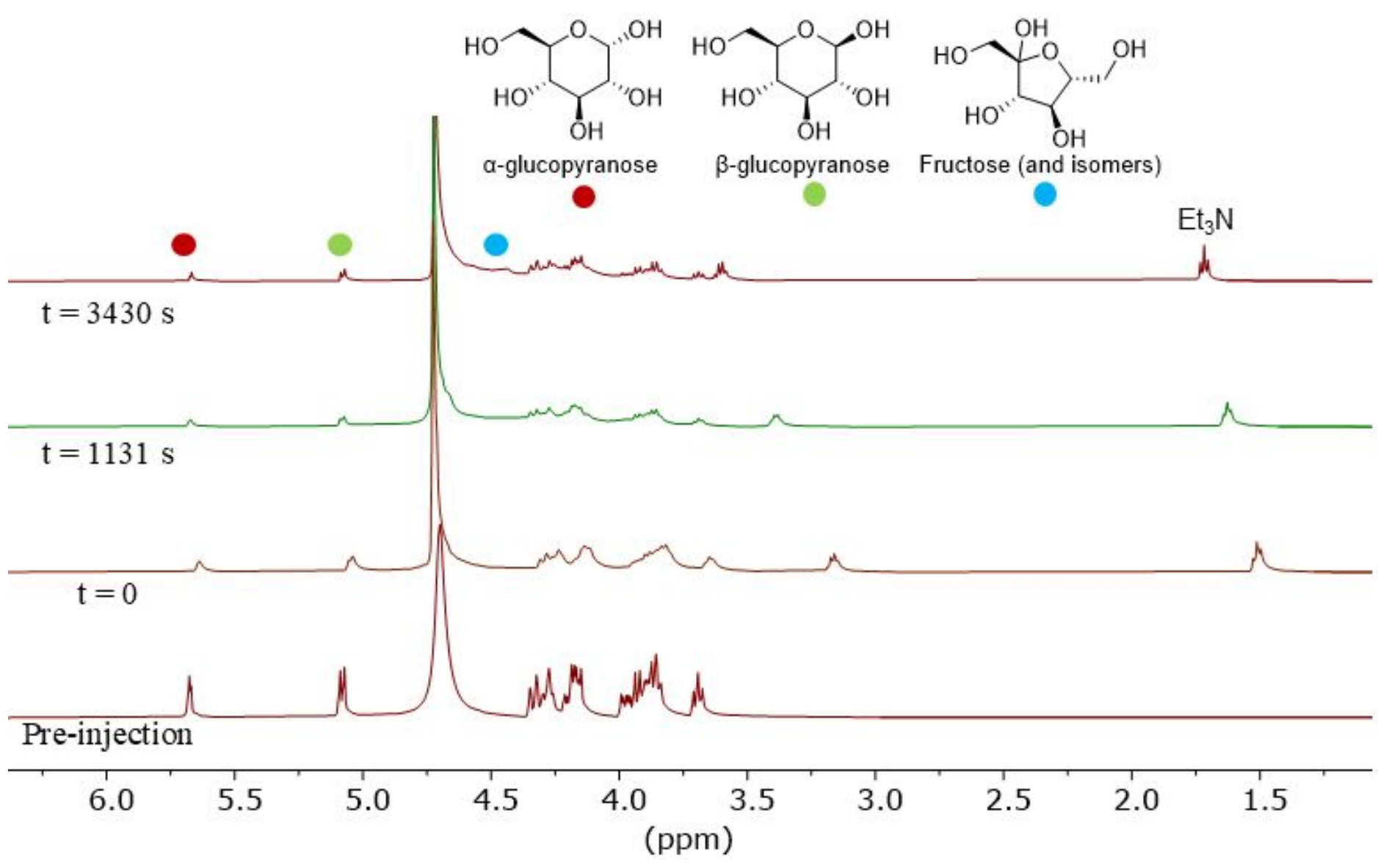

Figure S57. Reaction profile of Brønsted base-catalyzed isomerization of glucose.

8.3.3. Data Acquisition

During the injection, the sample was monitored with Bruker multizg program with the following parameters.

Pulse angle: 30 degrees
Number of scans (NS) = 1
Spectral center (o1p) = 6.18 ppm
Spectral width (sw) = 19.22 ppm
Acquisition time = 3.41 sec
Relaxation delay (d1) = 15 sec (ca 25 sec between each scan)

8.3.4. Data Analysis

After injection, the peak area of the first spectrum was defined as A_0, and the relative peak area of subsequent spectra A/A_0 was plotted against time. The resulting curve should follow a first-order reversible reaction model:

$$\frac{A}{A_0} = \left(\frac{A_{eq}}{A_0} + \frac{A_0 - A_{eq}}{A_0} e^{-(k_1+k_{-1})t}\right)$$

In the fitting, $(k_1 + k_{-1})$ was defined as k_{obs} and the data was fitted with Matlab curve fitter application to the following model to determine the fitted k_{obs} and its 95% confidence interval:

$$\frac{A}{A_0} = (C + A \times e^{-(k_1+k_{-1})t})$$

*Note: the α/β ratio of glucopyranose remains constant throughout the reaction (**Figure S56**), so monitoring of α-glucopyranose was used to determine the glucose concentration in the reaction.*

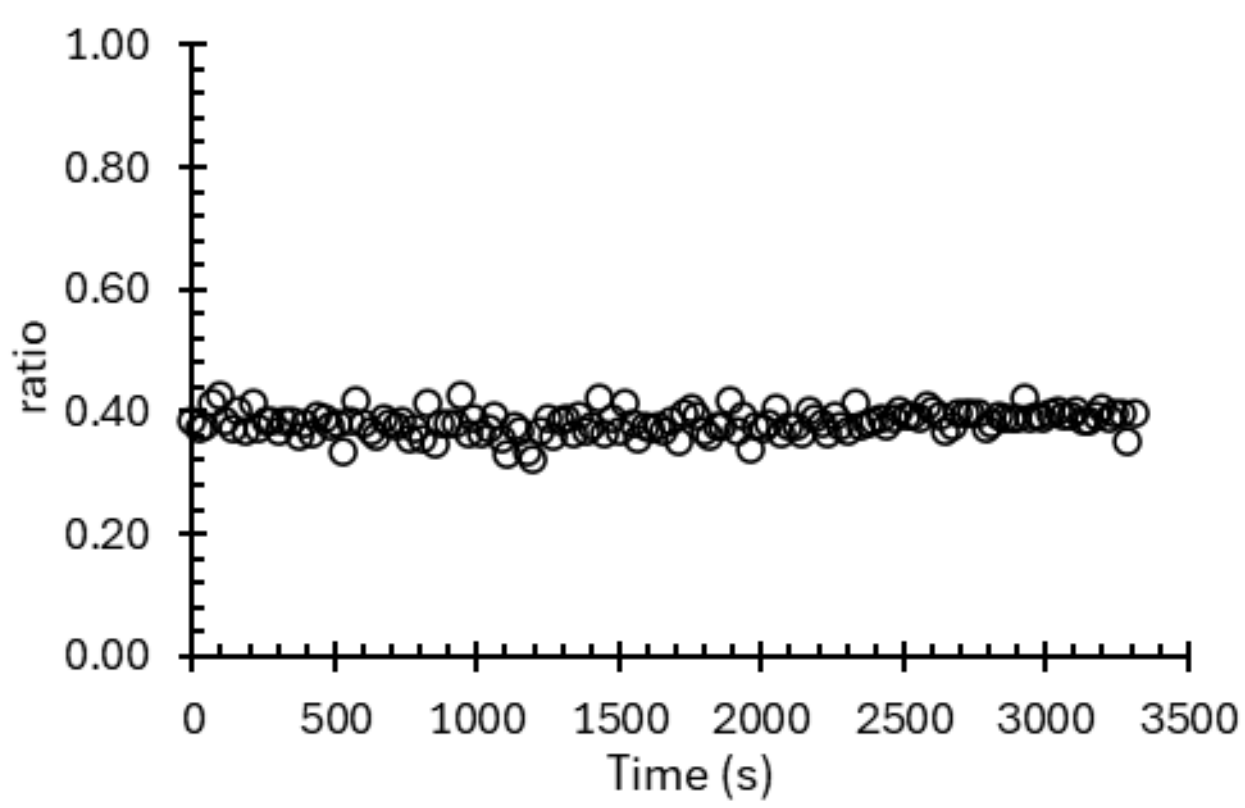

Figure S59. Isomeric ratio (α / (α + β)) of α-glucopyranose in run 1.

The experiment was repeated 3 times and the kinetic constants were averaged with inverse-variance weighting method.

8.3.5. Experiment Results

Run 1

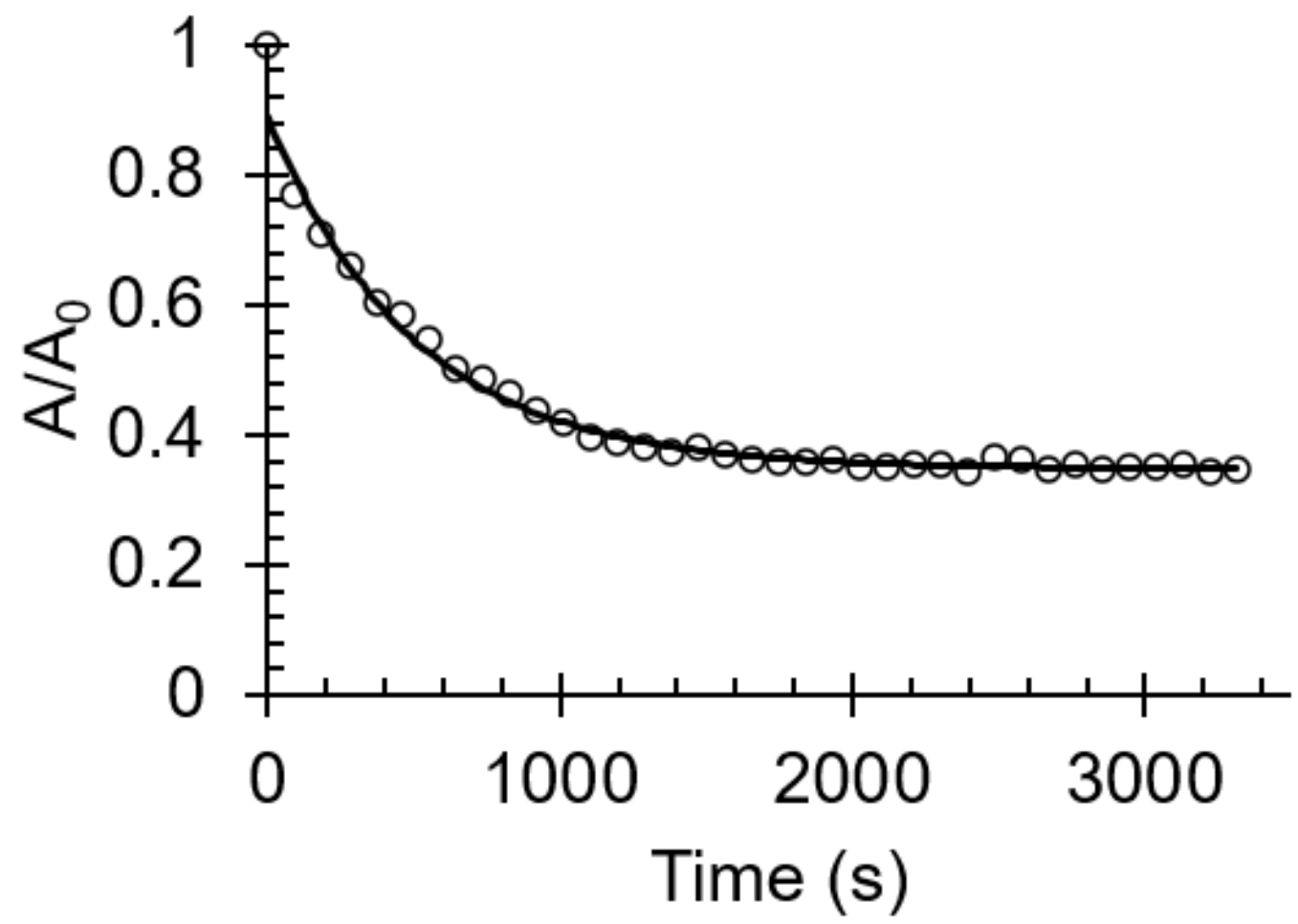

Figure S60. Curve fitting of base-catalyzed isomerization of glucose, run 1.

Fitting Results

Actual Et_3N loading: 11.1%
Fitting A/A_0 to t, using function f(t) = A*exp(-k*t)+C
Fitting results in 95% confidence bonds (n = 142; R^2 = 0.989)
A = 0.541 ± 0.011
C = 0.348 ± 0.003
k = (2.00 ± 0.077) × 10^{-3} (s^{-1})

Run 2

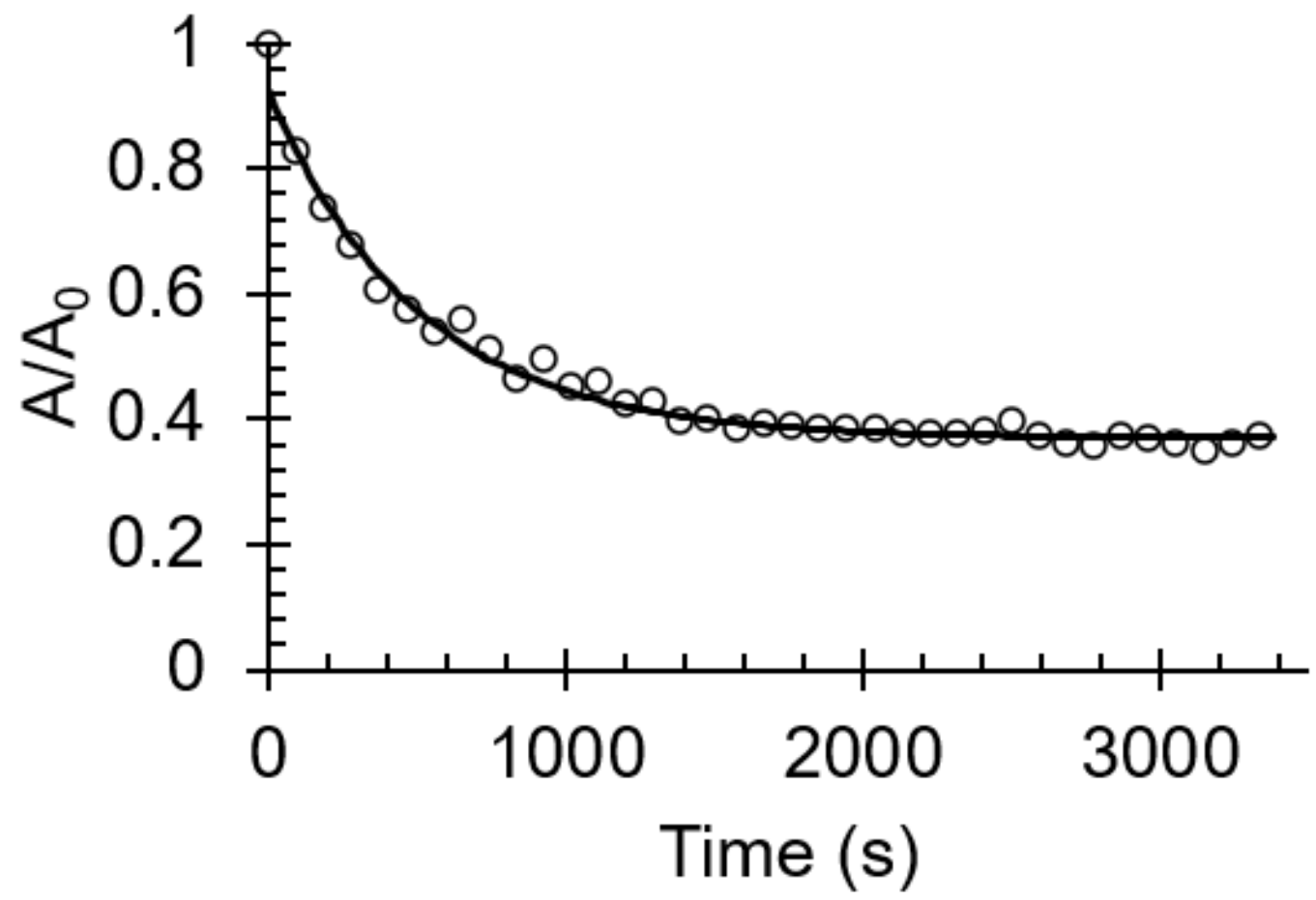

Figure S61. Curve fitting of base-catalyzed isomerization of glucose, run 2.

Fitting Results
Actual Et_3N loading: 11.1%
Fitting A/A_0 to t, using function f(t) = A*exp(-k*t)+C
Fitting results in 95% confidence bonds (n = 139; R^2 = 0.988)

A = 0.551 ± 0.012

C = 0.370 ± 0.004

k = (1.99 ± 0.081) × 10^{-3} (s^{-1})

Run 3

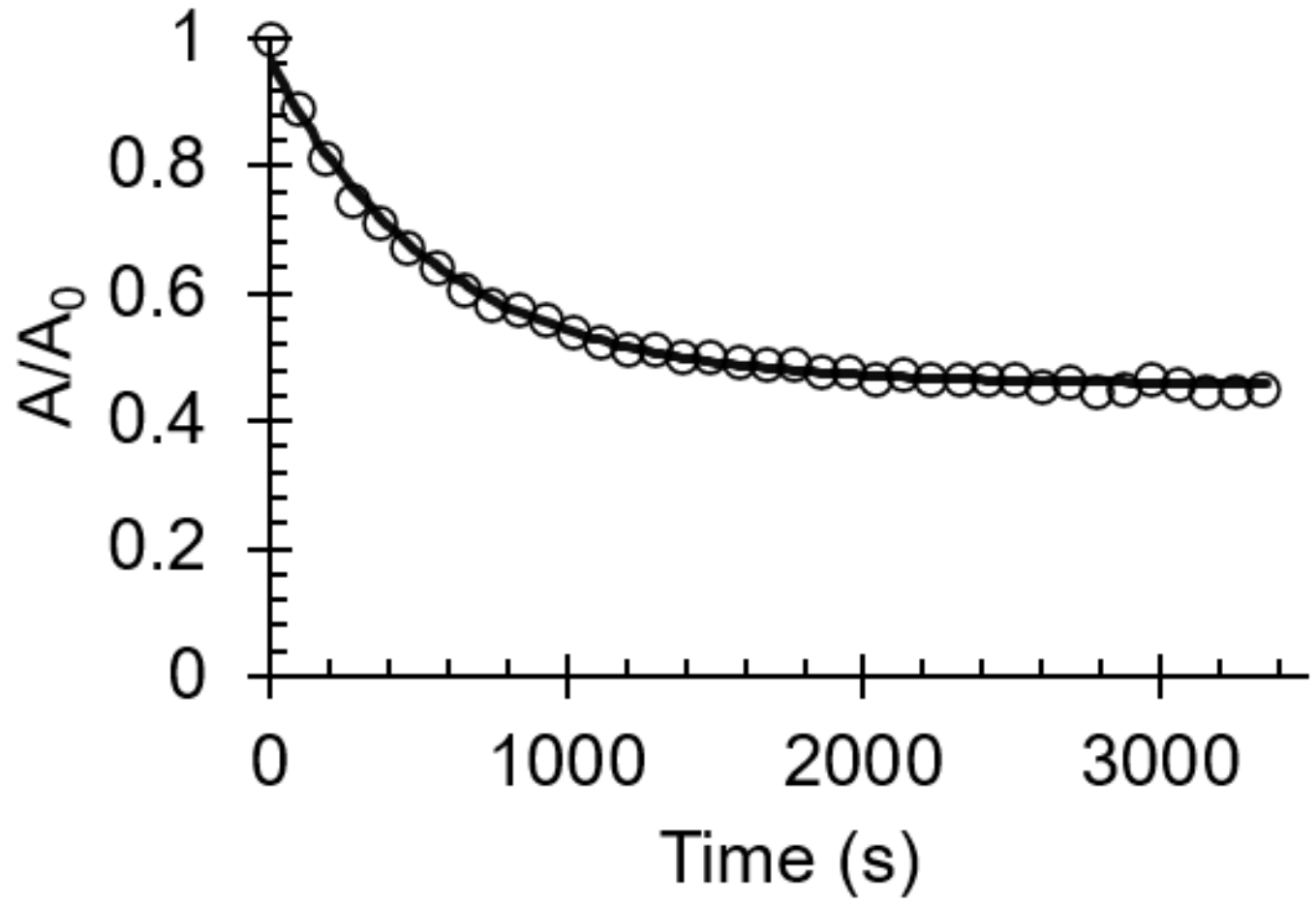

Figure S62. Curve fitting of base-catalyzed isomerization of glucose, run 3.

Fitting Results

Actual Et_3N loading: 12.5%

Fitting A/A_0 to t, using function f(t) = A*exp(-k*t)+C

Fitting results in 95% confidence bonds (n = 142; R^2 = 0.993)

A = 0.509 ± 0.008

C = 0.458 ± 0.003

k = (1.83 ± 0.056) × 10^{-3} (s^{-1})

8.3.6. Original Data

Table S18. Original data for run 1.

time (s)	alpha (δ5.5-5.8)	A/A_0
0	3.46E+06	1.00
23	3.02E+06	0.87
46	2.81E+06	0.81
69	2.65E+06	0.76
93	2.67E+06	0.77
116	2.61E+06	0.75
140	2.58E+06	0.74
163	2.50E+06	0.72
186	2.46E+06	0.71
209	2.37E+06	0.68
232	2.39E+06	0.69
255	2.38E+06	0.69
279	2.29E+06	0.66
302	2.23E+06	0.64
325	2.20E+06	0.64
348	2.16E+06	0.62
371	2.09E+06	0.60
394	2.09E+06	0.60
416	2.04E+06	0.59
439	2.02E+06	0.58
462	2.02E+06	0.58
485	1.95E+06	0.56
508	1.91E+06	0.55
531	1.78E+06	0.51
554	1.89E+06	0.55
577	1.83E+06	0.53
600	1.82E+06	0.52
623	1.71E+06	0.49
646	1.74E+06	0.50
669	1.75E+06	0.50
692	1.73E+06	0.50
715	1.71E+06	0.49
738	1.69E+06	0.49
761	1.64E+06	0.47
784	1.62E+06	0.47
807	1.54E+06	0.45
829	1.61E+06	0.46
852	1.50E+06	0.43
875	1.55E+06	0.45
898	1.53E+06	0.44
921	1.51E+06	0.44
945	1.50E+06	0.43
968	1.47E+06	0.42
991	1.46E+06	0.42
1014	1.45E+06	0.42
1038	1.39E+06	0.40
1061	1.42E+06	0.41
1084	1.38E+06	0.40
1107	1.38E+06	0.40
1130	1.37E+06	0.40
1153	1.37E+06	0.39
1176	1.34E+06	0.39
1199	1.34E+06	0.39
1223	1.34E+06	0.39
1245	1.37E+06	0.40
1269	1.33E+06	0.38
1291	1.32E+06	0.38
1314	1.36E+06	0.39
1337	1.33E+06	0.38
1360	1.35E+06	0.39
1383	1.30E+06	0.37
1405	1.28E+06	0.37
1428	1.33E+06	0.38
1451	1.26E+06	0.36
1474	1.32E+06	0.38
1497	1.29E+06	0.37
1521	1.30E+06	0.38
1543	1.29E+06	0.37
1567	1.28E+06	0.37
1591	1.29E+06	0.37
1613	1.28E+06	0.37
1636	1.30E+06	0.37
1659	1.26E+06	0.36
1682	1.30E+06	0.38
1705	1.25E+06	0.36
1727	1.30E+06	0.37
1750	1.25E+06	0.36
1773	1.29E+06	0.37
1797	1.26E+06	0.36
1820	1.24E+06	0.36

time (s)	alpha (δ5.5-5.8)	A/A_0
1843	1.24E+06	0.36
1866	1.26E+06	0.36
1889	1.27E+06	0.37
1912	1.24E+06	0.36
1935	1.26E+06	0.36
1958	1.23E+06	0.35
1981	1.26E+06	0.36
2004	1.22E+06	0.35
2027	1.22E+06	0.35
2049	1.24E+06	0.36
2072	1.23E+06	0.35
2095	1.24E+06	0.36
2119	1.22E+06	0.35
2142	1.22E+06	0.35
2165	1.22E+06	0.35
2188	1.25E+06	0.36
2211	1.23E+06	0.36
2234	1.20E+06	0.35
2257	1.23E+06	0.35
2280	1.22E+06	0.35
2303	1.22E+06	0.35
2326	1.22E+06	0.35
2349	1.22E+06	0.35
2372	1.22E+06	0.35
2395	1.20E+06	0.35
2418	1.24E+06	0.36
2440	1.20E+06	0.35
2463	1.21E+06	0.35
2486	1.26E+06	0.36
2510	1.24E+06	0.36
2533	1.22E+06	0.35
2556	1.21E+06	0.35
2579	1.26E+06	0.36
2602	1.21E+06	0.35
2625	1.23E+06	0.35
2648	1.21E+06	0.35
2671	1.20E+06	0.35
2694	1.21E+06	0.35
2717	1.21E+06	0.35
2739	1.22E+06	0.35
2763	1.23E+06	0.35
2786	1.21E+06	0.35
2808	1.23E+06	0.35

time (s)	alpha (δ5.5-5.8)	A/A_0
2831	1.22E+06	0.35
2855	1.20E+06	0.35
2878	1.20E+06	0.35
2901	1.20E+06	0.35
2924	1.22E+06	0.35
2947	1.21E+06	0.35
2970	1.22E+06	0.35
2992	1.23E+06	0.35
3015	1.25E+06	0.36
3038	1.21E+06	0.35
3061	1.22E+06	0.35
3084	1.21E+06	0.35
3107	1.24E+06	0.36
3129	1.23E+06	0.36
3152	1.21E+06	0.35
3175	1.22E+06	0.35
3197	1.25E+06	0.36
3220	1.19E+06	0.34
3243	1.19E+06	0.34
3266	1.24E+06	0.36
3289	1.18E+06	0.34
3313	1.20E+06	0.35

Table S19. Original data for run 2.

time (s)	alpha (δ5.6-5.7)	A/A_0
0	3.57E+06	1.00
23	3.06E+06	0.86
47	3.12E+06	0.87
70	3.12E+06	0.87
93	2.97E+06	0.83
117	2.82E+06	0.79
140	2.77E+06	0.78
163	2.73E+06	0.76
186	2.64E+06	0.74
209	2.66E+06	0.74
232	2.64E+06	0.74
255	2.45E+06	0.69
278	2.42E+06	0.68
301	2.39E+06	0.67
324	2.38E+06	0.67
347	2.30E+06	0.64
370	2.17E+06	0.61
393	2.15E+06	0.60
416	2.07E+06	0.58
439	2.11E+06	0.59
462	2.06E+06	0.58
485	2.00E+06	0.56
508	1.99E+06	0.56
531	1.97E+06	0.55
554	1.93E+06	0.54
577	1.90E+06	0.53
600	1.92E+06	0.54
624	1.85E+06	0.52
647	2.00E+06	0.56
670	1.91E+06	0.54
693	1.78E+06	0.50
717	1.77E+06	0.50
740	1.83E+06	0.51
763	1.74E+06	0.49
786	1.75E+06	0.49
809	1.72E+06	0.48
832	1.66E+06	0.46
854	1.82E+06	0.51
877	1.85E+06	0.52
901	1.99E+06	0.56

time (s)	alpha (δ5.6-5.7)	A/A_0
923	1.77E+06	0.50
946	1.65E+06	0.46
969	1.60E+06	0.45
993	1.72E+06	0.48
1016	1.61E+06	0.45
1039	1.58E+06	0.44
1062	1.56E+06	0.44
1085	1.55E+06	0.43
1108	1.65E+06	0.46
1131	1.58E+06	0.44
1154	1.95E+06	0.54
1177	1.53E+06	0.43
1200	1.52E+06	0.42
1224	1.52E+06	0.43
1246	1.47E+06	0.41
1270	1.50E+06	0.42
1293	1.53E+06	0.43
1316	1.49E+06	0.42
1339	1.48E+06	0.41
1362	1.47E+06	0.41
1385	1.42E+06	0.40
1408	1.80E+06	0.50
1431	1.40E+06	0.39
1454	1.43E+06	0.40
1477	1.44E+06	0.40
1500	1.40E+06	0.39
1524	1.53E+06	0.43
1548	1.43E+06	0.40
1570	1.38E+06	0.38
1593	1.40E+06	0.39
1616	1.41E+06	0.40
1639	1.38E+06	0.39
1662	1.40E+06	0.39
1685	1.40E+06	0.39
1708	1.41E+06	0.39
1731	1.40E+06	0.39
1754	1.39E+06	0.39
1777	1.40E+06	0.39
1801	1.42E+06	0.40
1826	1.40E+06	0.39

time (s)	alpha (δ5.6-5.7)	A/A_0
1850	1.38E+06	0.39
1873	1.37E+06	0.38
1897	1.36E+06	0.38
1920	1.38E+06	0.39
1943	1.38E+06	0.39
1966	1.36E+06	0.38
1990	1.38E+06	0.39
2012	1.38E+06	0.39
2036	1.38E+06	0.39
2059	1.35E+06	0.38
2082	1.35E+06	0.38
2105	1.37E+06	0.38
2128	1.36E+06	0.38
2150	1.34E+06	0.38
2174	1.35E+06	0.38
2197	1.35E+06	0.38
2220	1.36E+06	0.38
2243	1.33E+06	0.37
2266	1.36E+06	0.38
2289	1.32E+06	0.37
2312	1.35E+06	0.38
2335	1.36E+06	0.38
2358	1.34E+06	0.37
2381	1.41E+06	0.39
2404	1.37E+06	0.38
2427	1.33E+06	0.37
2450	1.33E+06	0.37
2473	1.35E+06	0.38
2497	1.42E+06	0.40
2521	1.33E+06	0.37
2544	1.33E+06	0.37
2567	1.34E+06	0.37
2590	1.34E+06	0.37
2613	1.37E+06	0.38
2636	1.30E+06	0.36
2660	1.33E+06	0.37
2683	1.29E+06	0.36
2706	1.33E+06	0.37
2729	1.31E+06	0.37
2752	1.25E+06	0.35

time (s)	alpha (δ5.6-5.7)	A/A_0
2774	1.29E+06	0.36
2797	1.28E+06	0.36
2821	1.28E+06	0.36
2844	1.37E+06	0.38
2868	1.33E+06	0.37
2891	1.26E+06	0.35
2914	1.33E+06	0.37
2937	1.33E+06	0.37
2960	1.32E+06	0.37
2983	1.33E+06	0.37
3005	1.33E+06	0.37
3029	1.33E+06	0.37
3051	1.30E+06	0.36
3075	1.29E+06	0.36
3099	1.33E+06	0.37
3122	1.31E+06	0.37
3145	1.26E+06	0.35
3168	1.31E+06	0.37
3191	1.32E+06	0.37
3213	1.26E+06	0.35
3236	1.29E+06	0.36
3259	1.24E+06	0.35
3283	1.35E+06	0.38
3307	1.30E+06	0.36
3330	1.34E+06	0.38
3353	1.33E+06	0.37
3375	1.30E+06	0.36

Table S20. Original data for run 3.

time (s)	alpha (δ5.6-5.7)	A/A_0
0	2.76E+06	1.00
24	2.77E+06	1.00
46	2.56E+06	0.93
70	2.51E+06	0.91
93	2.46E+06	0.89
116	2.42E+06	0.88
139	2.30E+06	0.83
162	2.23E+06	0.81
186	2.24E+06	0.81
209	2.18E+06	0.79
232	2.16E+06	0.78
256	2.10E+06	0.76
279	2.06E+06	0.75
302	2.03E+06	0.73
325	2.03E+06	0.74
348	1.96E+06	0.71
371	1.97E+06	0.71
394	1.92E+06	0.70
417	1.90E+06	0.69
440	1.86E+06	0.67
463	1.86E+06	0.67
487	1.84E+06	0.66
510	1.84E+06	0.67
533	1.77E+06	0.64
556	1.78E+06	0.64
579	1.74E+06	0.63
602	1.76E+06	0.64
625	1.69E+06	0.61
648	1.67E+06	0.61
671	1.66E+06	0.60
694	1.65E+06	0.60
717	1.64E+06	0.59
740	1.61E+06	0.58
764	1.62E+06	0.59
787	1.60E+06	0.58
810	1.58E+06	0.57
833	1.59E+06	0.58
856	1.55E+06	0.56
879	1.56E+06	0.57
902	1.61E+06	0.58

time (s)	alpha (δ5.6-5.7)	A/A_0
925	1.54E+06	0.56
948	1.51E+06	0.55
971	1.50E+06	0.54
994	1.50E+06	0.54
1017	1.49E+06	0.54
1040	1.49E+06	0.54
1063	1.51E+06	0.55
1086	1.47E+06	0.53
1109	1.45E+06	0.53
1133	1.46E+06	0.53
1156	1.46E+06	0.53
1180	1.45E+06	0.53
1203	1.42E+06	0.52
1226	1.42E+06	0.51
1250	1.42E+06	0.51
1274	1.46E+06	0.53
1297	1.42E+06	0.51
1320	1.39E+06	0.50
1343	1.38E+06	0.50
1366	1.41E+06	0.51
1390	1.39E+06	0.50
1413	1.38E+06	0.50
1436	1.40E+06	0.51
1459	1.39E+06	0.50
1482	1.38E+06	0.50
1506	1.39E+06	0.50
1529	1.34E+06	0.49
1552	1.37E+06	0.50
1575	1.37E+06	0.49
1598	1.34E+06	0.49
1620	1.36E+06	0.49
1643	1.36E+06	0.49
1667	1.36E+06	0.49
1690	1.35E+06	0.49
1713	1.32E+06	0.48
1737	1.35E+06	0.49
1761	1.35E+06	0.49
1784	1.32E+06	0.48
1807	1.34E+06	0.49
1829	1.33E+06	0.48

time (s)	alpha (δ5.6-5.7)	A/A_0
1852	1.32E+06	0.48
1876	1.33E+06	0.48
1898	1.31E+06	0.47
1921	1.35E+06	0.49
1945	1.32E+06	0.48
1968	1.32E+06	0.48
1992	1.31E+06	0.47
2015	1.31E+06	0.47
2038	1.29E+06	0.47
2061	1.32E+06	0.48
2084	1.30E+06	0.47
2107	1.32E+06	0.48
2130	1.31E+06	0.47
2154	1.31E+06	0.47
2177	1.28E+06	0.46
2200	1.28E+06	0.46
2224	1.29E+06	0.47
2247	1.29E+06	0.47
2271	1.31E+06	0.48
2295	1.29E+06	0.47
2319	1.29E+06	0.47
2342	1.28E+06	0.46
2365	1.30E+06	0.47
2388	1.29E+06	0.47
2412	1.29E+06	0.47
2434	1.29E+06	0.47
2457	1.28E+06	0.46
2481	1.26E+06	0.46
2504	1.29E+06	0.47
2527	1.30E+06	0.47
2550	1.26E+06	0.46
2573	1.27E+06	0.46
2596	1.26E+06	0.46
2619	1.28E+06	0.46
2642	1.26E+06	0.45
2666	1.26E+06	0.46
2689	1.28E+06	0.46
2712	1.28E+06	0.46
2735	1.25E+06	0.45
2758	1.28E+06	0.46

time (s)	alpha (δ5.6-5.7)	A/A_0
2781	0.45	0.45
2804	0.46	0.46
2827	0.45	0.45
2851	0.45	0.45
2874	0.45	0.45
2897	0.45	0.45
2921	0.46	0.46
2944	0.45	0.45
2968	0.47	0.47
2991	0.45	0.45
3014	0.46	0.46
3037	0.46	0.46
3060	0.46	0.46
3083	0.45	0.45
3106	0.45	0.45
3129	0.45	0.45
3152	0.45	0.45
3175	0.45	0.45
3199	0.45	0.45
3223	0.44	0.44
3246	0.45	0.45
3269	0.45	0.45
3292	0.45	0.45
3315	0.44	0.44
3338	0.45	0.45
3361	0.45	0.45

8.4. Kinetic Study of the Mumm Rearrangement

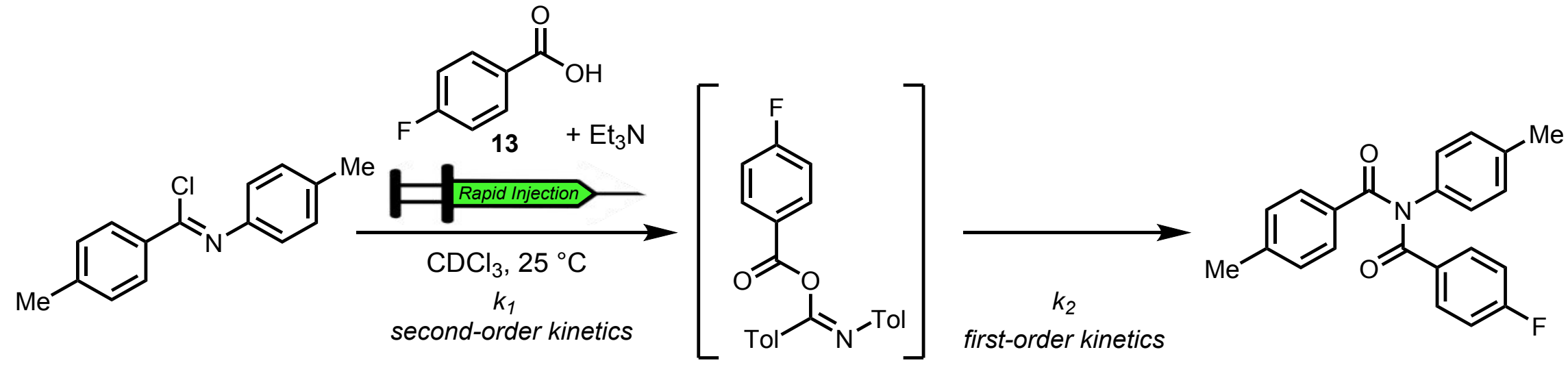

12 14 15

8.4.1. Experimental Procedure

The following stock solutions were freshly prepared for the reaction:

- A stock solution of **12** (0.10 M) with 1,2-difluorobenzene was prepared by diluting **12** (121.9 mg, 0.5 mmol) and 1,2-difluorobenzene (53.2 mg, 0.466 mmol) in chloroform-*d* in a N_2-purged 5.0 mL volumetric flask.
- A stock solution of **13** (1.0 M) was prepared by dissolving **13** (140.0 mg, 1.0 mmol) and triethylamine (303.0 mg, 3.0 mmol) with chloroform-*d* in a 1.0 mL volumetric flask and loaded to an injector syringe.

Stock solution of **12** (500 µL, 0.050 mmol, 1.0 equiv) was transferred to an oven-dried NMR tube. The sample tube was placed into a N_2-purged NMR spectrometer maintained at 25 °C. Stock solution of **13** was injected (55 µL, 0.055 mmol, 1.1 equiv) while the sample was being monitored by ^{19}F NMR. After the conclusion of kinetic experiments, an additional ^{19}F spectrum was acquired to determine the actual excess of **13**.

8.4.2. Peak Identification

The signals were compared to ^{19}F spectra of **13**-triethylamine (δ −111.7 ppm) and **10** (δ −106.07 ppm) (**Figure S63**). The ^{19}F signals of **13**, **14**, and **15** were monitored with internal standard (1,2-difluorobenzene, δ –138.3 ppm, 2 F) to track the reaction profile. A separate ^{1}H-observed injection without internal standard was conducted for structural elucidation (**Figure S64**).

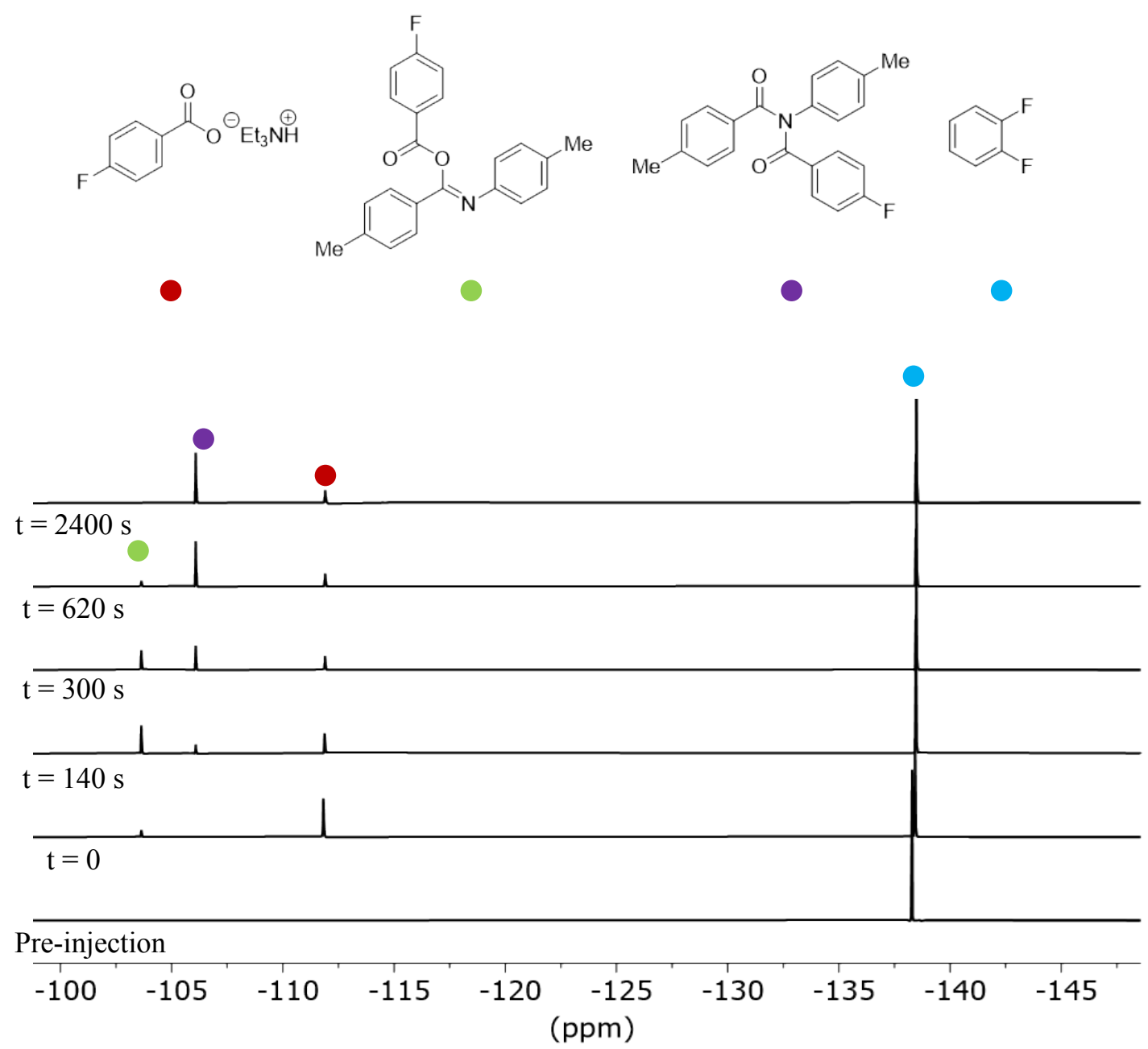

Figure S63. Peak Identification of the Mumm rearrangement, ^{19}F.

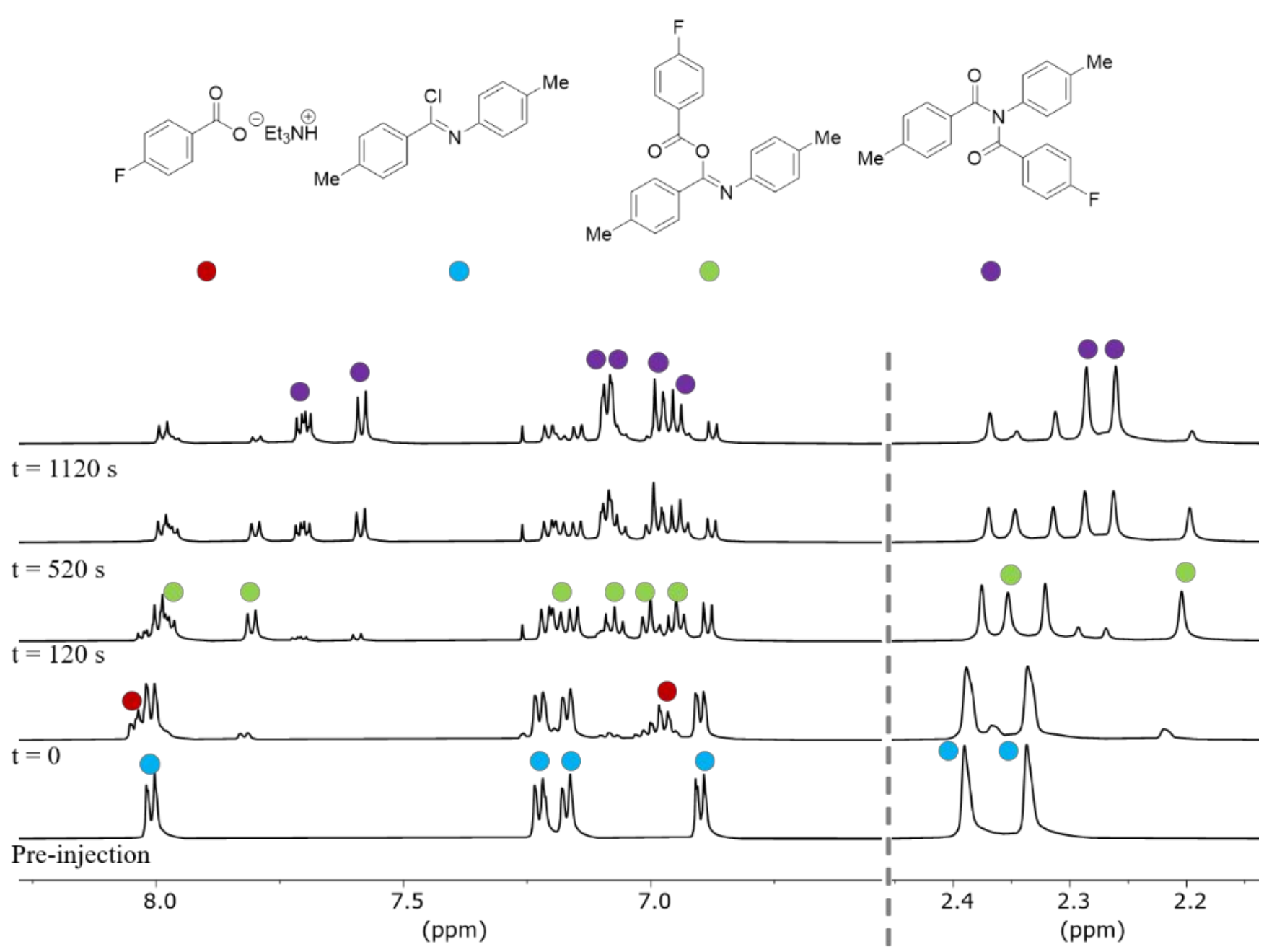

Figure S64. Peak Identification of the Mumm rearrangement, ^{1}H.

8.4.3. Data Acquisition

During the injection, the sample was monitored with a pseudo-2D program on ^{19}F with the following parameters.

Pulse angle: 30 degrees
Number of scans (NS) = 1
Spectral center (o1p) = −100 ppm
Spectral width (sw) = 241.5 ppm
Acquisition time = 0.57 sec
Variable delay (vd): 30 ms (for all scans)
Relaxation delay (d1) = 19.4 sec (20.0 s between each scan)

After the conclusion of the experiment, an additional spectrum was acquired with the following parameters.

Pulse angle: 30 degrees
Number of scans (NS) = 8
Spectral center (o1p) = −100 ppm
Spectral width (sw) = 241.5 ppm
Acquisition time = 0.57 sec
Relaxation delay (d1) = 20 sec

8.4.4. Data Analysis

Concentration was calculated by integration of the ^{19}F peaks relative to the internal standard and the total volume after injection (550 µL). The reaction profile of all ^{19}F species were fitted with the following rate equations:

$$\frac{d[\mathbf{13}]}{dt} = -\,[\mathbf{12}]\,[\mathbf{13}]k_1$$

$$\frac{d[\mathbf{14}]}{dt} = [\mathbf{12}]\,[\mathbf{13}]k_1 - [\mathbf{14}]k_2$$

$$\frac{d[\mathbf{15}]}{dt} = [\mathbf{14}]k_2$$

Since **12** is not observable by ^{19}F NMR, the excess of **13** relative to **12** was defined as δ ($[\mathbf{12}] = [\mathbf{13}] - \delta$). Therefore, the differential equations can be rewritten as:

$$\frac{d[\mathbf{13}]}{dt} = -\,[\mathbf{13}]([\mathbf{13}] - \delta)k_1$$

$$\frac{d[\mathbf{14}]}{dt} = [\mathbf{13}]([\mathbf{13}] - \delta)k_1 - [\mathbf{14}]k_2$$

$$\frac{d[\mathbf{15}]}{dt} = [\mathbf{14}]k_2$$

Where δ was measured in the post-injection acquisition and constrained between ±10% bound during fitting. With this setup, the differential equation was evaluated with ode45 solver, δ, k_1 and k_2 were optimized using nonlinear least-square curve fitter tool (lsqcurvefit) in Matlab®, and the confidence intervals were evaluated with nonlinear regression parameter confidence intervals tool (nlparci). The results of three runs were averaged with inverse-variance weighting method.

Full Matlab® Script for optimization

```
%Data import
S0 = S(1); I0 = I(1); P0 = P(1);
Conc_input = [S; I; P];
y0 = [S(1); I(1); P(1)];
Excess = 0.0351; %input from post injection measurement

%Initial guess
%p = [k1 k2 d];
startpt = [0.1, 0.001, Excess];

%Boundaries
% [k1 k2 d];
lb = [0, 0, Excess * 0.90];
ub = [1, 1, Excess * 1.10];

%Fitting method
options = optimoptions('lsqcurvefit', 'Display', 'iter', 'ScaleProblem', 'obj-and-constr',
'StepTolerance', 1e-20, 'FunctionTolerance', 1e-20);
[k_final, resnorm, residual, exitflag, output, lambda, jacobian] =
lsqcurvefit(@(p,t)fittingmodel(p,t,y0), startpt, t, Conc_input, lb, ub, options);

%Calculate CI
ci = nlparci(k_final, residual, 'jacobian', jacobian, 'alpha', 0.05);
errbar = (ci(:,2) - ci(:,1))/2;

%generate fitted curves
    k1 = k_final(1); k2 = k_final(2); d = k_final(3);
    [~, y_fitted] = ode45(@(t,y) [
    - k1*y(1)*(y(1)-d);
    k1*y(1)*(y(1)-d) - k2*y(2);
    k2*y(2);
    ], t, y0);

%Outputs
disp("k1 k2 d=")
disp(vpa(k_final, 10));
disp("95% Confidence Intervals (Lower, Upper):")
disp(vpa(ci, 10));
disp("error bars:")
disp(vpa(errbar, 10));

%-------------functions---------------
%set Fitting models
function GuessStack = fittingmodel(p, t, y0)
    %Assign variables
    k1 = p(1);
    k2 = p(2);
    d  = p(3);

%--------RATE LAW--------
    rate_eqn = @(t,y) [
    - k1*y(1)*(y(1)-d);
    k1*y(1)*(y(1)-d) - k2*y(2);
    k2*y(2)
    ];

    %Solve ODE
    [~, y_out] = ode45(rate_eqn, t, y0);

    %Stacked guess functions
    GuessStack = [
        y_out(:, 1);
        y_out(:, 2);
        y_out(:, 3);
        ];
end
```

8.4.5. Experiment Results

Run 1

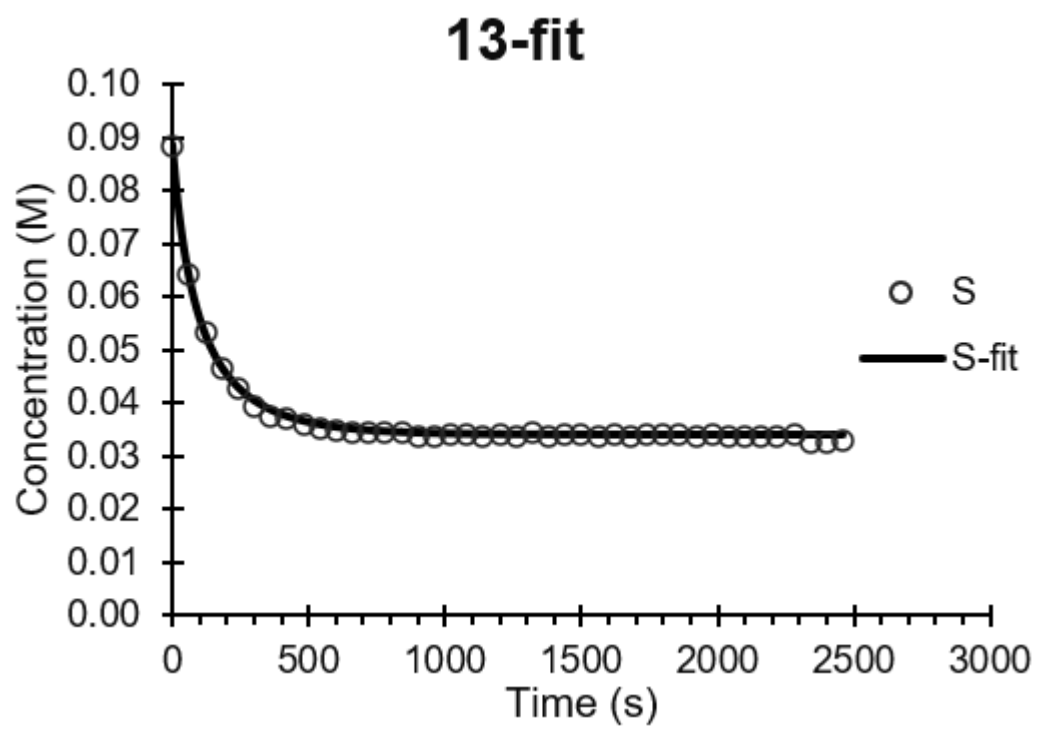

$R^2 = 0.996$

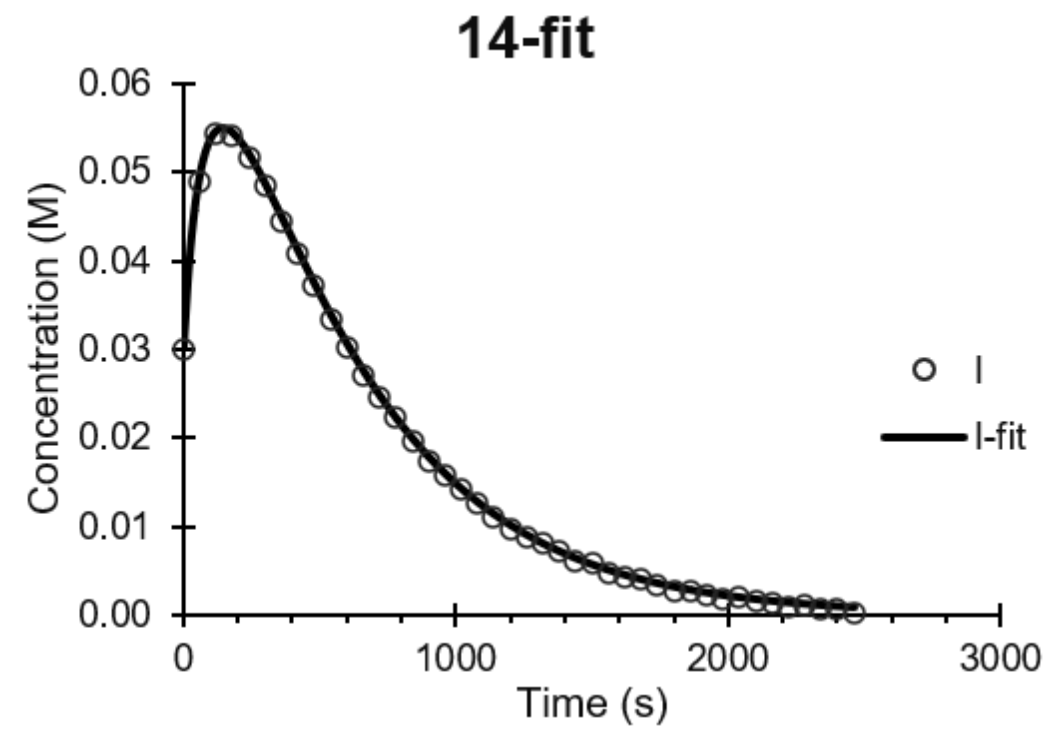

$R^2 = 0.999$

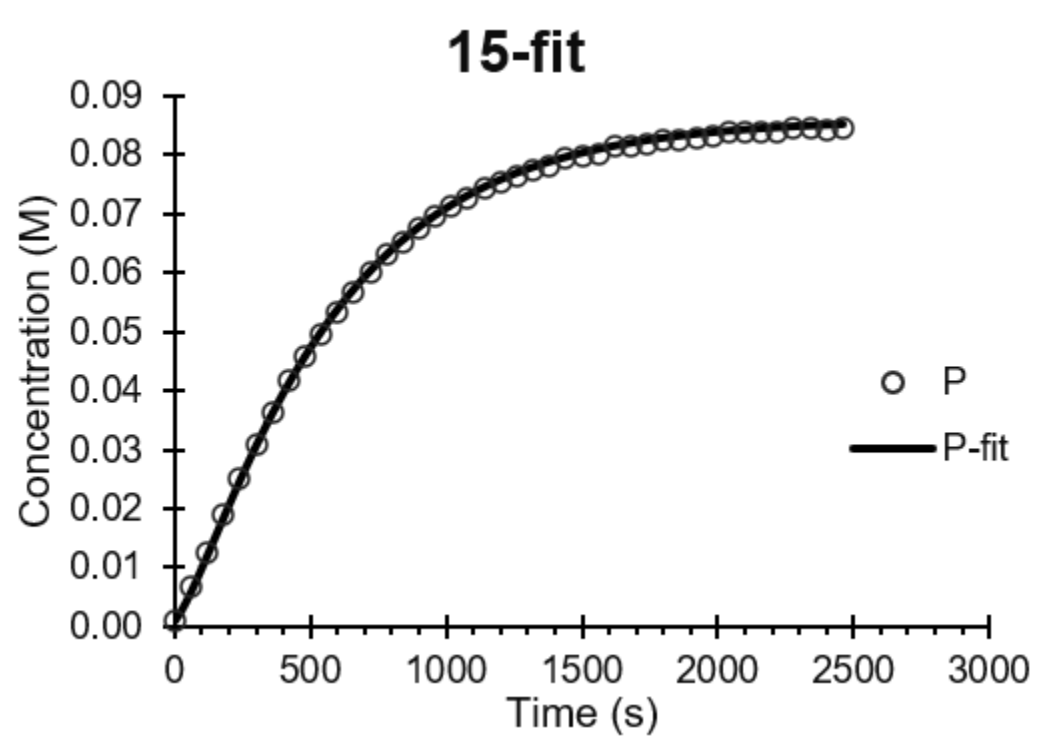

$R^2 = 0.999$

Fitting Results (n = 124)
Measured excess: 0.037 M
$\delta = (3.40 \pm 0.07) \times 10^{-2}$ M
$k_1 = (1.33 \pm 0.02) \times 10^{-1}$ M^{-1} s^{-1}
$k_2 = (1.95 \pm 0.0062) \times 10^{-3}$ s^{-1}

Run 2

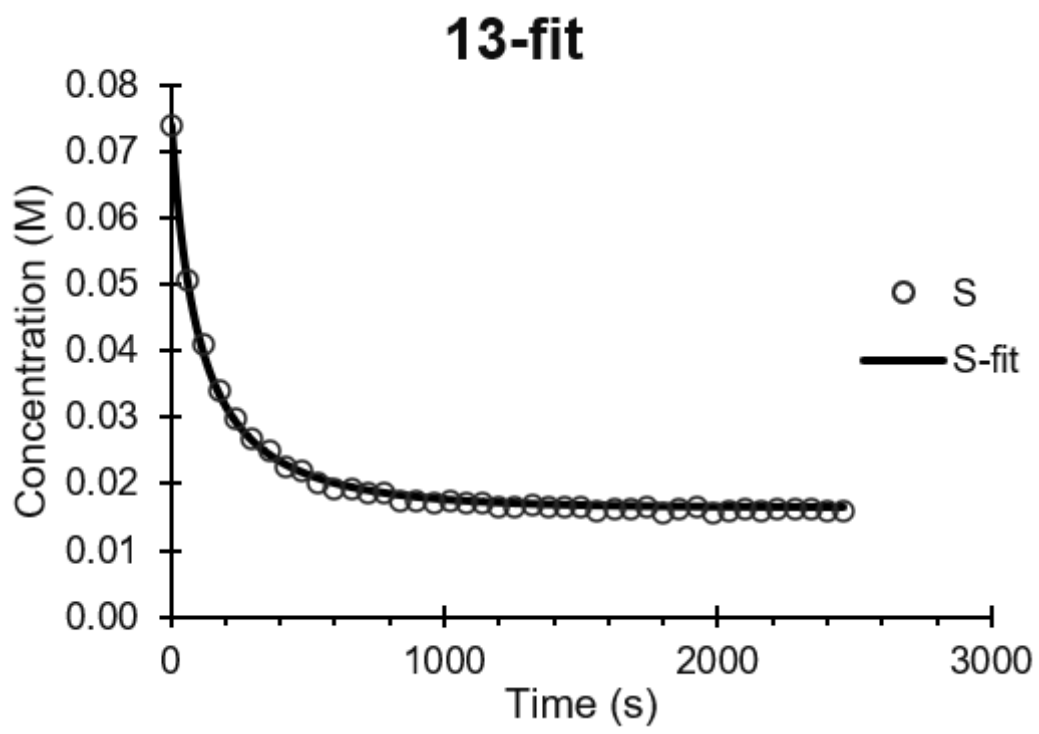

$R^2 = 0.995$

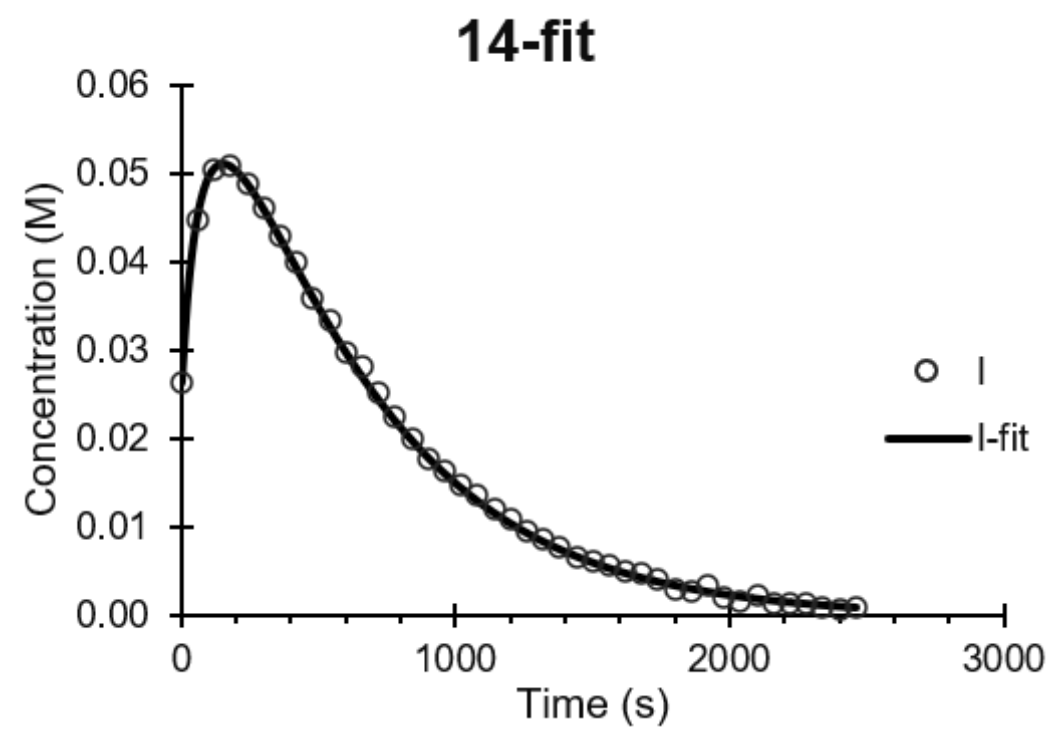

$R^2 = 0.999$

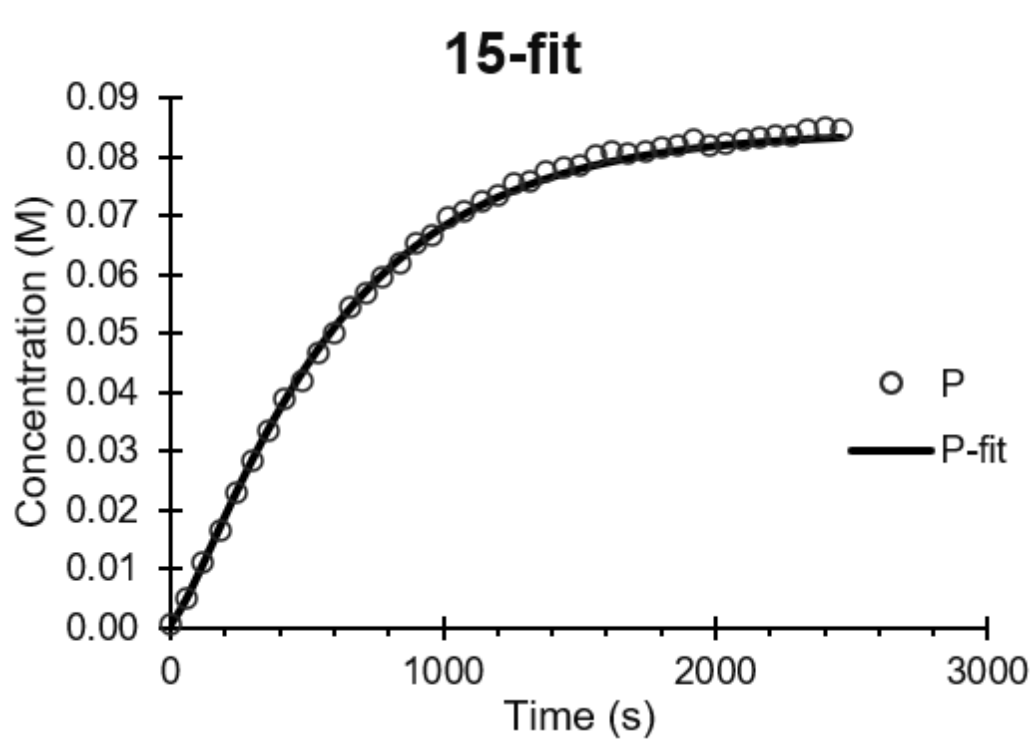

$R^2 = 0.999$

Fitting Results (n = 124)
Measured excess: 0.018 M
$\delta = (1.66 \pm 0.01) \times 10^{-2}$ M
$k_1 = (1.48 \pm 0.03) \times 10^{-1}$ M^{-1} s^{-1}
$k_2 = (1.98 \pm 0.01) \times 10^{-3}$ s^{-1}

Run 3

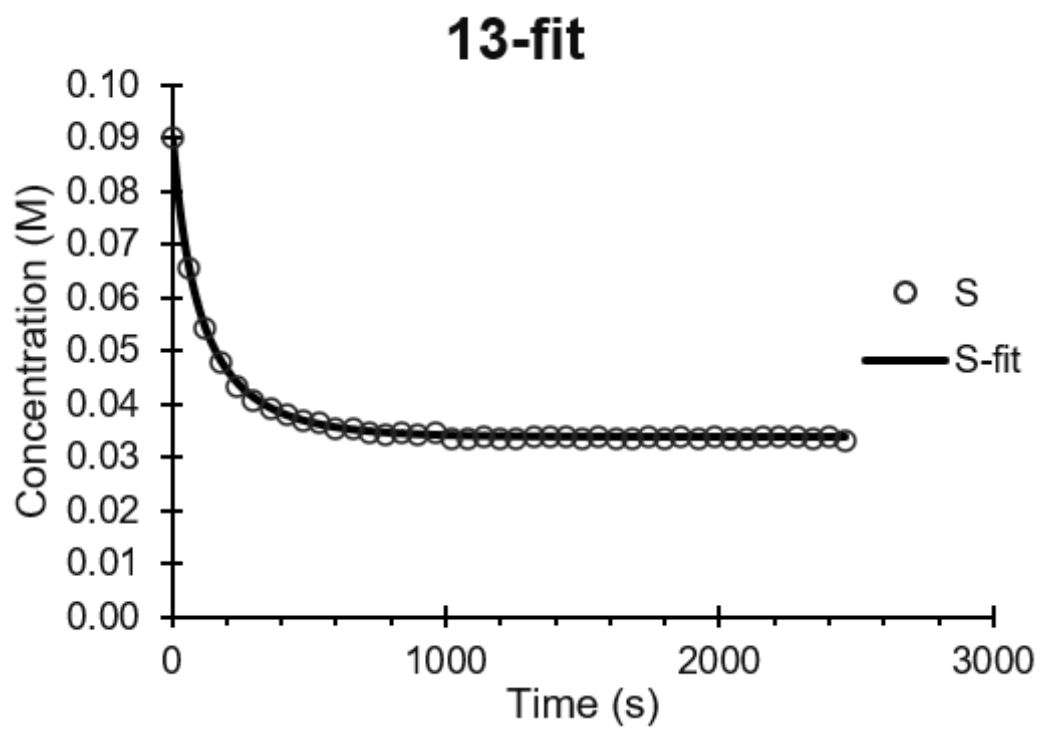

$R^2 = 0.999$

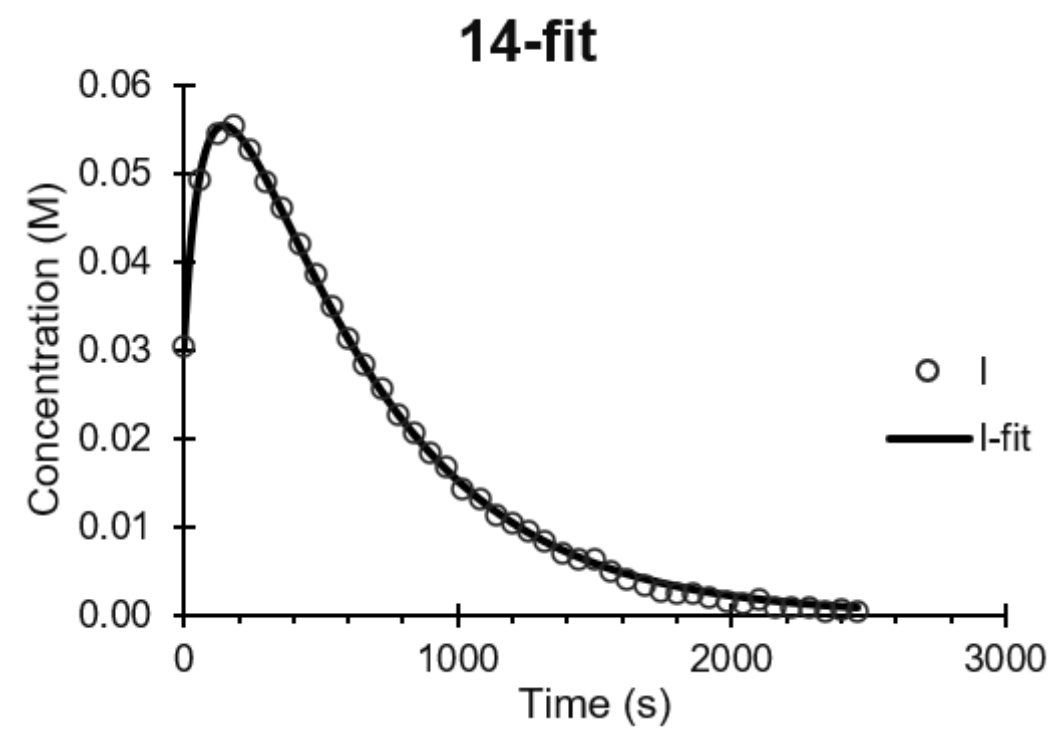

$R^2 = 0.999$

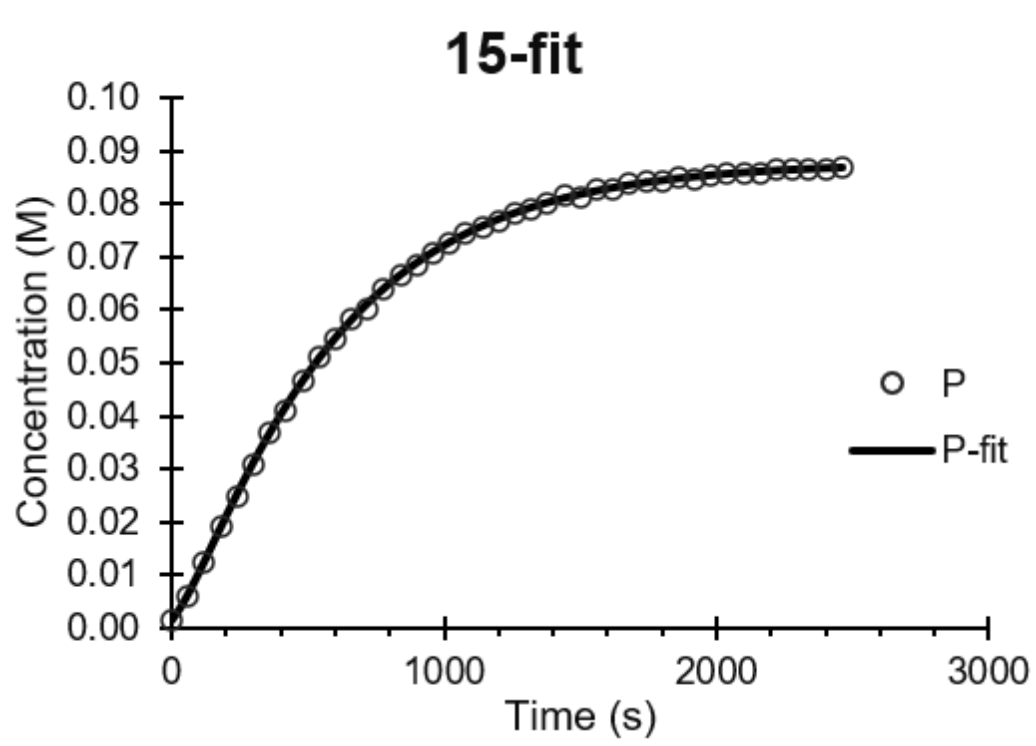

$R^2 = 0.999$

Fitting Results (n = 124)
Measured excess: 0.035 M
$\delta = (3.37 \pm 0.01) \times 10^{-2}$ M
$k_1 = (1.25 \pm 0.01) \times 10^{-1}$ M^{-1} s^{-1}
$k_2 = (1.95 \pm 0.005) \times 10^{-3}$ s^{-1}

8.4.6. Original Data

Table S21. Original data for Run 1.

Time (s)	**8** (M)	**9** (M)	**10** (M)
0	0.088	0.030	0.001
20	0.078	0.039	0.003
40	0.071	0.045	0.005
60	0.064	0.049	0.007
80	0.060	0.051	0.008
100	0.056	0.054	0.010
120	0.053	0.054	0.012
140	0.050	0.054	0.015
160	0.048	0.055	0.017
180	0.047	0.054	0.019
200	0.045	0.053	0.021
220	0.043	0.053	0.023
240	0.043	0.052	0.025
260	0.042	0.051	0.027
280	0.041	0.050	0.029
300	0.039	0.048	0.031
320	0.039	0.047	0.033
340	0.038	0.046	0.034
360	0.037	0.044	0.036
380	0.037	0.044	0.038
400	0.037	0.042	0.040
420	0.037	0.041	0.042
440	0.036	0.040	0.043
460	0.036	0.039	0.044
480	0.036	0.037	0.046
500	0.035	0.036	0.047
520	0.036	0.034	0.049
540	0.035	0.033	0.050
560	0.035	0.032	0.052
580	0.035	0.031	0.052
600	0.035	0.030	0.053
620	0.034	0.029	0.055
640	0.035	0.028	0.056
660	0.034	0.027	0.057
680	0.034	0.026	0.058
700	0.034	0.026	0.059
720	0.035	0.025	0.060
740	0.035	0.024	0.061
760	0.034	0.023	0.062
780	0.034	0.022	0.063
800	0.034	0.021	0.064
820	0.034	0.020	0.064

840	0.034	0.020	0.065
860	0.034	0.019	0.066
880	0.034	0.018	0.067
900	0.034	0.017	0.068
920	0.034	0.017	0.068
940	0.034	0.016	0.069
960	0.034	0.016	0.070
980	0.034	0.015	0.070
1000	0.034	0.015	0.071
1020	0.034	0.014	0.071
1040	0.034	0.014	0.072
1060	0.034	0.013	0.072
1080	0.034	0.013	0.073
1100	0.034	0.012	0.073
1120	0.034	0.012	0.073
1140	0.034	0.011	0.074
1160	0.034	0.011	0.075
1180	0.034	0.011	0.075
1200	0.034	0.010	0.075
1220	0.034	0.010	0.075
1240	0.034	0.009	0.076
1260	0.034	0.009	0.076
1280	0.034	0.008	0.077
1300	0.034	0.008	0.077
1320	0.034	0.008	0.078
1340	0.034	0.008	0.078
1360	0.034	0.008	0.078
1380	0.034	0.007	0.078
1400	0.034	0.007	0.079
1420	0.034	0.007	0.079
1440	0.034	0.006	0.079
1460	0.034	0.006	0.079
1480	0.034	0.006	0.080
1500	0.034	0.006	0.080
1520	0.034	0.005	0.080
1540	0.034	0.005	0.080
1560	0.034	0.005	0.080
1580	0.033	0.005	0.080
1600	0.034	0.005	0.081
1620	0.034	0.004	0.081
1640	0.034	0.004	0.081
1660	0.034	0.004	0.082
1680	0.034	0.004	0.082
1700	0.034	0.003	0.082
1720	0.034	0.004	0.082

1740	0.034	0.004	0.082
1760	0.034	0.003	0.081
1780	0.034	0.003	0.082
1800	0.034	0.003	0.082
1820	0.034	0.003	0.083
1840	0.034	0.003	0.082
1860	0.034	0.003	0.082
1880	0.034	0.003	0.083
1900	0.034	0.002	0.083
1920	0.034	0.002	0.083
1940	0.033	0.002	0.083
1960	0.033	0.002	0.083
1980	0.034	0.002	0.083
2000	0.034	0.002	0.083
2020	0.034	0.002	0.083
2040	0.034	0.002	0.084
2060	0.034	0.002	0.083
2080	0.034	0.002	0.084
2100	0.034	0.002	0.084
2120	0.034	0.002	0.084
2140	0.034	0.002	0.084
2160	0.034	0.001	0.084
2180	0.034	0.001	0.084
2200	0.033	0.002	0.084
2220	0.034	0.001	0.084
2240	0.034	0.001	0.084
2260	0.034	0.001	0.084
2280	0.034	0.001	0.085
2300	0.034	0.001	0.084
2320	0.034	0.001	0.084
2340	0.033	0.001	0.085
2360	0.033	0.001	0.085
2380	0.033	0.001	0.084
2400	0.033	0.001	0.084
2420	0.033	0.001	0.084
2440	0.033	0.001	0.085
2460	0.033	0.000	0.085

Table S22. Original data for Run 2.

Time (s)	**8** (M)	**9** (M)	**10** (M)
0	0.074	0.026	0.001
20	0.064	0.036	0.002
40	0.057	0.040	0.004
60	0.051	0.045	0.005
80	0.047	0.048	0.007
100	0.042	0.049	0.010
120	0.041	0.050	0.011
140	0.038	0.050	0.013
160	0.035	0.051	0.015
180	0.034	0.051	0.017
200	0.033	0.050	0.019
220	0.030	0.050	0.021
240	0.030	0.049	0.023
260	0.028	0.048	0.025
280	0.028	0.047	0.026
300	0.027	0.046	0.029
320	0.025	0.044	0.029
340	0.025	0.044	0.032
360	0.025	0.043	0.034
380	0.023	0.042	0.035
400	0.023	0.040	0.036
420	0.023	0.040	0.039
440	0.022	0.039	0.040
460	0.022	0.037	0.042
480	0.022	0.036	0.042
500	0.022	0.035	0.044
520	0.021	0.034	0.046
540	0.020	0.033	0.047
560	0.021	0.032	0.048
580	0.020	0.031	0.050
600	0.019	0.030	0.050
620	0.019	0.029	0.052
640	0.020	0.028	0.053
660	0.019	0.028	0.054
680	0.020	0.027	0.055
700	0.019	0.026	0.056
720	0.019	0.025	0.057
740	0.019	0.024	0.057
760	0.018	0.023	0.059
780	0.019	0.022	0.060
800	0.018	0.021	0.061
820	0.019	0.021	0.062
840	0.017	0.020	0.062

860	0.018	0.020	0.063
880	0.018	0.019	0.064
900	0.018	0.018	0.065
920	0.018	0.018	0.065
940	0.017	0.016	0.067
960	0.017	0.016	0.067
980	0.018	0.016	0.067
1000	0.017	0.015	0.068
1020	0.017	0.015	0.070
1040	0.017	0.014	0.069
1060	0.017	0.014	0.071
1080	0.017	0.014	0.071
1100	0.017	0.013	0.071
1120	0.016	0.012	0.072
1140	0.017	0.012	0.072
1160	0.017	0.011	0.073
1180	0.017	0.012	0.073
1200	0.016	0.011	0.074
1220	0.017	0.010	0.074
1240	0.017	0.010	0.075
1260	0.016	0.009	0.075
1280	0.017	0.009	0.075
1300	0.017	0.009	0.076
1320	0.017	0.009	0.076
1340	0.017	0.008	0.077
1360	0.017	0.009	0.076
1380	0.017	0.008	0.077
1400	0.017	0.007	0.078
1420	0.017	0.007	0.078
1440	0.017	0.007	0.078
1460	0.015	0.007	0.078
1480	0.017	0.006	0.078
1500	0.016	0.006	0.079
1520	0.016	0.006	0.079
1540	0.016	0.006	0.079
1560	0.016	0.006	0.080
1580	0.016	0.005	0.079
1600	0.016	0.006	0.081
1620	0.016	0.005	0.081
1640	0.016	0.004	0.080
1660	0.016	0.005	0.081
1680	0.016	0.005	0.081
1700	0.016	0.004	0.081
1720	0.016	0.004	0.081
1740	0.017	0.004	0.081

1760	0.017	0.004	0.081
1780	0.017	0.003	0.082
1800	0.015	0.003	0.082
1820	0.016	0.002	0.081
1840	0.016	0.002	0.083
1860	0.016	0.003	0.082
1880	0.016	0.003	0.083
1900	0.016	0.002	0.082
1920	0.016	0.003	0.083
1940	0.016	0.002	0.083
1960	0.017	0.002	0.083
1980	0.016	0.002	0.082
2000	0.016	0.002	0.083
2020	0.016	0.002	0.084
2040	0.016	0.002	0.082
2060	0.016	0.002	0.083
2080	0.016	0.002	0.084
2100	0.016	0.002	0.083
2120	0.016	0.001	0.084
2140	0.016	0.002	0.083
2160	0.016	0.001	0.083
2180	0.016	0.001	0.084
2200	0.016	0.001	0.084
2220	0.016	0.001	0.084
2240	0.016	0.001	0.083
2260	0.016	0.001	0.084
2280	0.016	0.001	0.084
2300	0.016	0.001	0.084
2320	0.016	0.001	0.084
2340	0.016	0.001	0.085
2360	0.017	0.001	0.084
2380	0.015	0.001	0.084
2400	0.016	0.001	0.085
2420	0.017	0.001	0.085
2440	0.016	0.001	0.084
2460	0.016	0.001	0.085

Table S23. Original data for Run 3.

Time (s)	**8** (M)	**9** (M)	**10** (M)
0	0.090	0.030	0.001
20	0.080	0.040	0.003
40	0.072	0.046	0.004
60	0.066	0.049	0.006
80	0.061	0.052	0.008
100	0.057	0.054	0.010
120	0.054	0.055	0.012
140	0.052	0.056	0.014
160	0.049	0.055	0.017
180	0.048	0.056	0.019
200	0.046	0.055	0.021
220	0.045	0.053	0.023
240	0.043	0.053	0.025
260	0.042	0.052	0.027
280	0.041	0.050	0.029
300	0.041	0.049	0.031
320	0.040	0.049	0.033
340	0.039	0.047	0.035
360	0.039	0.046	0.037
380	0.038	0.045	0.039
400	0.038	0.043	0.040
420	0.038	0.042	0.041
440	0.037	0.040	0.043
460	0.037	0.040	0.045
480	0.037	0.039	0.047
500	0.037	0.037	0.048
520	0.036	0.036	0.049
540	0.037	0.035	0.051
560	0.036	0.034	0.052
580	0.035	0.033	0.053
600	0.035	0.031	0.055
620	0.036	0.031	0.055
640	0.035	0.029	0.057
660	0.035	0.028	0.058
680	0.036	0.028	0.059
700	0.035	0.026	0.060
720	0.035	0.026	0.060
740	0.034	0.025	0.062
760	0.035	0.024	0.063
780	0.034	0.023	0.064
800	0.035	0.022	0.065
820	0.034	0.021	0.066
840	0.034	0.021	0.067

860	0.035	0.020	0.067
880	0.034	0.019	0.068
900	0.034	0.018	0.068
920	0.034	0.017	0.069
940	0.034	0.017	0.070
960	0.035	0.017	0.071
980	0.035	0.016	0.072
1000	0.034	0.015	0.072
1020	0.033	0.014	0.072
1040	0.034	0.015	0.074
1060	0.034	0.014	0.073
1080	0.034	0.013	0.075
1100	0.034	0.012	0.075
1120	0.034	0.012	0.076
1140	0.034	0.011	0.076
1160	0.034	0.011	0.076
1180	0.034	0.011	0.077
1200	0.034	0.010	0.077
1220	0.034	0.010	0.077
1240	0.034	0.010	0.078
1260	0.033	0.009	0.078
1280	0.034	0.009	0.079
1300	0.034	0.009	0.079
1320	0.034	0.008	0.079
1340	0.033	0.008	0.080
1360	0.034	0.008	0.080
1380	0.034	0.007	0.080
1400	0.034	0.007	0.081
1420	0.034	0.007	0.081
1440	0.034	0.006	0.081
1460	0.034	0.007	0.081
1480	0.034	0.006	0.082
1500	0.033	0.006	0.081
1520	0.034	0.004	0.082
1540	0.034	0.004	0.082
1560	0.034	0.005	0.083
1580	0.034	0.005	0.083
1600	0.034	0.004	0.083
1620	0.034	0.004	0.083
1640	0.034	0.004	0.083
1660	0.034	0.004	0.084
1680	0.033	0.003	0.084
1700	0.034	0.003	0.084
1720	0.034	0.004	0.084
1740	0.034	0.003	0.084

1760	0.034	0.003	0.084
1780	0.034	0.003	0.084
1800	0.033	0.003	0.084
1820	0.034	0.003	0.085
1840	0.033	0.003	0.085
1860	0.034	0.002	0.085
1880	0.034	0.002	0.085
1900	0.033	0.003	0.085
1920	0.034	0.002	0.085
1940	0.034	0.002	0.085
1960	0.034	0.002	0.085
1980	0.034	0.002	0.085
2000	0.033	0.002	0.085
2020	0.034	0.001	0.086
2040	0.033	0.001	0.086
2060	0.034	0.001	0.086
2080	0.034	0.001	0.086
2100	0.034	0.002	0.086
2120	0.034	0.001	0.086
2140	0.034	0.001	0.087
2160	0.034	0.001	0.086
2180	0.034	0.001	0.087
2200	0.033	0.001	0.086
2220	0.034	0.001	0.086
2240	0.033	0.001	0.086
2260	0.034	0.001	0.086
2280	0.034	0.001	0.086
2300	0.034	0.000	0.087
2320	0.034	0.001	0.086
2340	0.033	0.001	0.087
2360	0.033	0.001	0.087
2380	0.034	0.001	0.087
2400	0.034	0.001	0.087
2420	0.034	0.001	0.087
2440	0.033	0.001	0.088
2460	0.033	0.000	0.087

8.4.7. Synthesis of Starting Materials

4-Methyl-N-(p-tolyl)benzamide (S4)

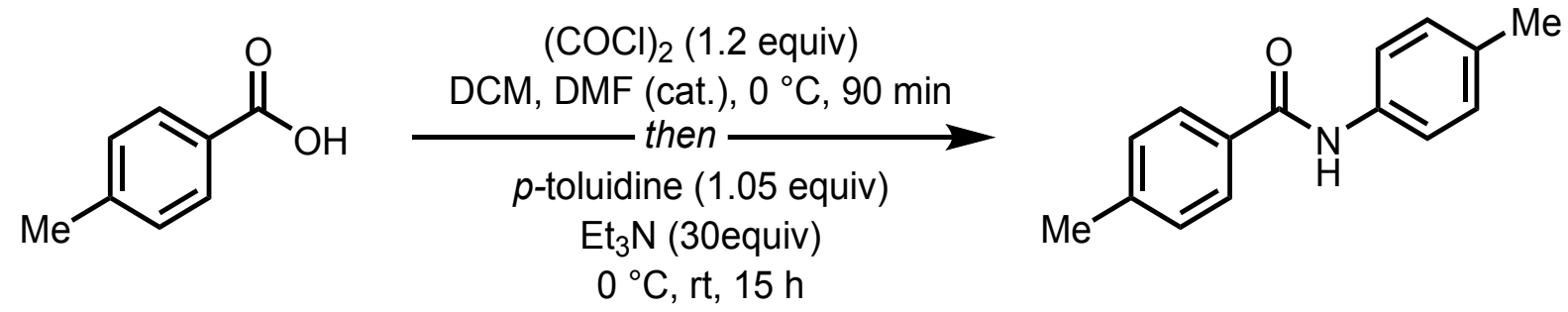

4-Methyl-*N*-(*p*-tolyl)benzamide (**S4**) was synthesized following a reported literature procedure.[6] *p*-Toluic acid (2.72 g, 20.0 mmol, 1.00 equiv) was dissolved in dichloromethane (50 mL, 0.40 M) under N_2 atmosphere and the reaction was cooled to 0 °C in an ice bath. Oxalyl chloride (2.06 mL, 1.20 equiv) was added dropwise at 0 °C, followed by the addition of catalytic *N*,*N*-dimethylformamide (200 μL). The reaction was then allowed to warm to room temperature and stirred for 1.5 h. After 1.5 h, the reaction was re-cooled to 0 °C, Triethylamine (8.32 mL, 3.00 equiv) and *p*-toluidine (2.25 g, 1.05 equiv) was added by briefly removing the septa cap. The reaction was allowed to warm up to room temperature and stirred for 15 h. After full conversion was confirmed with GC-MS, saturated $NaHCO_3$ (50 mL) was added, and the reaction mixture was extracted with dichloromethane (3 × 50 mL). The organic layers were combined and washed with saturated brine solution (50 mL) then dried with Na_2SO_4, and the solvent was removed *in vacuo* to afford crude **S4** as a brown solid (4.77 g, quant). ^{1}H NMR is in correspondence to the reported literature.[7] The crude material was used in the synthesis of **7** without further purification.

4-Methyl-N-(p-tolyl)benzimidoyl chloride (12)

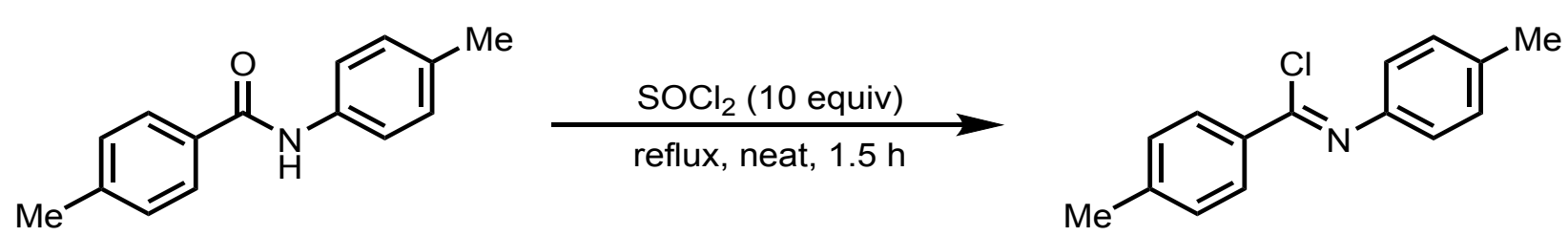

4-Methyl-*N*-(*p*-tolyl)benzimidoyl chloride **12** was synthesized by refluxing crude **S4** (4.77 g) in neat $SOCl_2$ (15 mL, 10.3 equiv) in an external oil bath set to 95 °C for 1.5 h. After the full consumption of **S4** was confirmed (^{1}H NMR), excess $SOCl_2$ was removed *in vacuo*. Hot hexanes (10 mL) was added to the resulting solid and the dark residue was removed with hot filtration. The filtrate was collected, and the solvent was removed *in vacuo* to afford **7** as a pale-orange crystal (4.37 g, 90% over two steps).

^{1}H NMR (500 MHz, $CDCl_3$) δ 7.96 (d, *J* = 7.9 Hz, 2H), 7.17 (d, *J* = 8.0 Hz, 2H), 7.12 (d, *J* = 7.8 Hz, 2H), 6.85 (d, *J* = 7.9 Hz, 2H), 2.33 (s, 3H), 2.28 (s, 3H).

^{13}C NMR (126 MHz, $CDCl_3$) δ 145.1, 142.9, 142.7, 134.8, 133.0, 129.5, 129.5, 129.3, 120.7, 21.6, 21.2.
ESI-MS (*m/z*): decomposed to amide during ionization. $[M+H]^+$ calculated for $C_{15}H_{16}NO^+$: 226.1226; found 226.1223.

4-Fluorobenzoic acid (13)

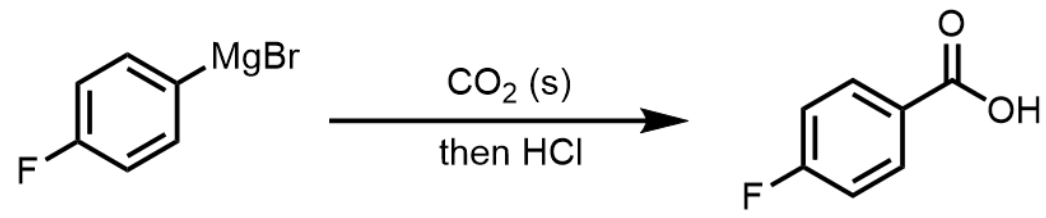

A flame-dried, argon-purged, uncapped 100 mL Schlenk flask was charged with excess crushed dry ice. Under a stream of argon, 4-fluorophenylmagnesium bromide (15 mmol, 1.35 M in THF, determined using the method of Hoye[8]) was added dropwise. After addition, the remaining dry ice was allowed to sublime. The residue was dissolved in a mixture of EtOAc/HCl (aq., 2M), the extracted with EtOAc (3x). The combined organic phase was washed with brine, dried over Na_2SO_4, filtered and concentrated to afford **13** as a white crystalline powder (1.54 g, 73%). The spectra are consistent with those previously reported.[9]

^{1}H NMR (400 MHz, $CDCl_3$) δ 8.19 – 8.09 (m, 2H), 7.21 – 7.10 (m, 2H).

^{13}C NMR (101 MHz, $CDCl_3$) δ 170.9, 166.5 (d, *J* = 256 Hz), 133.1 (d, *J* = 9.5 Hz), 125.6 (d, *J* = 3 Hz), 115.9 (d, *J* = 22.1 Hz).

^{19}F NMR (376 MHz, $CDCl_3$) δ −104.06 (tt, *J* = 8.3, 5.5 Hz).

4-Fluorobenzoic acid, triethylamine salt (S5)

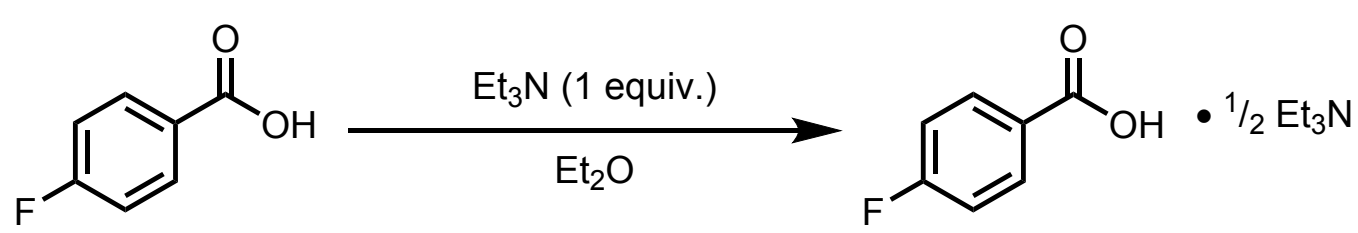

A flame-dried round-bottom flask was charged with **13** (700 mg, 5 mmol, 1 equiv.). Et_2O (30 mL) was added, then Et_3N (505 mg, 5 mmol, 1 equiv.) was added dropwise. After addition, the solution was concentrated to afford a yellow oil. The residue was recrystallized from cold pentane as a white powder with a stoichiometry of 2:1 benzoate: Et_3N (679.6 mg, 73%). The material was used in the synthesis of **15** with no further purification.
^{1}H NMR (500 MHz, $CDCl_3$) δ 11.93 (s,2H), 8.11 – 8.04 (m, 4H), 7.05 (t, *J* = 8.7 Hz, 4H), 3.18 (q, *J* = 7.3 Hz, 6H), 1.31 (t, *J* = 7.3 Hz, 9H).
^{13}C NMR (126 MHz, $CDCl_3$) δ 170.6, 165.2 (d, *J* = 251.0 Hz), 132.2, 132.1, 130.3, 130.2, 115.1, 114.9, 45.2, 8.6.
^{19}F NMR (471 MHz, $CDCl_3$) δ -108.80 – -108.93 (m).

4-Fluoro-*N*-(4-methylbenzoyl)-*N*-(*p*-tolyl)benzamide (15)

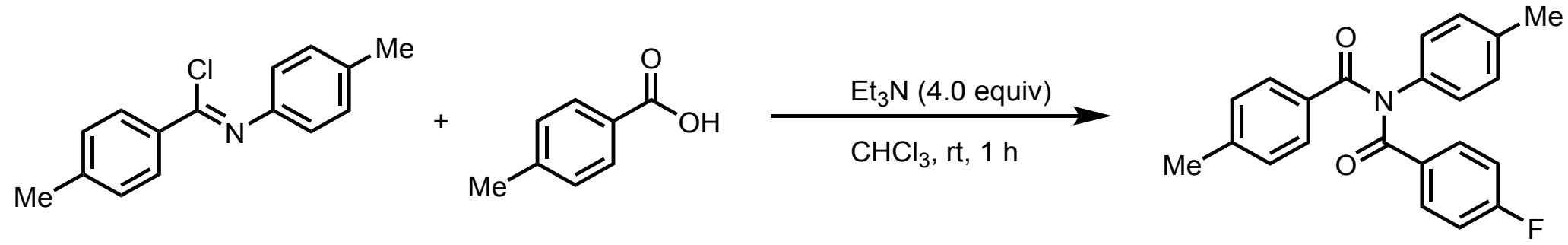

An oven-dried scintillation vial was charged with **12** (243 mg, 1 mmol) and **S5** (201 mg, 1.5 mmol, 1.5 equiv). Chloroform (5 mL) was added, followed by dropwise addition of triethylamine (303 mg, 3 mmol, 3 equiv). The solution was stirred at room temperature for 1 h, then washed with sat. $NaHCO_3$ (*aq*), extracted with Et_2O, washed with brine, dried over Na_2SO_4, and concentrated to afford a beige solid (346.7 mg, quant).

^{1}H NMR (500 MHz, $CDCl_3$) δ 7.73 – 7.64 (m, 2H), 7.60 – 7.54 (m, 2H), 7.07 (dd, J = 8.1, 4.4 Hz, 4H), 7.02 – 6.90 (m, 4H), 2.27 (s, 3H), 2.25 (s, 3H).

^{13}C NMR (126 MHz, $CDCl_3$) δ 173.5, 172.6, 165.1 (d, J = 254.5 Hz), 143.4, 137.8, 137.8, 132.0, 131.9 (d, J = 3.0 Hz), 131.4, 131.4, 130.4, 129.7, 129.4, 127.6, 115.8 (d, J = 22.3 Hz), 77.4, 77.2, 76.9, 21.8, 21.2.

^{19}F NMR (471 MHz, $CDCl_3$) δ −106.04 (tt, J = 8.4, 5.4 Hz).

ESI-MS (*m/z*): $[M+H]^+$ calculated for $C_{22}H_{19}FNO_2^+$: 348.1394; found: 348.1390.

8.4.8. T_1 Measurements

A standard ^{1}H NMR inversion-recovery sequence (180° - τ - 90°) was employed to measure the spin-lattice relaxation time constant (T_1). The spectral center was moved to the olefin signal for data collection, and the peak area was plotted against time and fitted to the exponential decay model:

$$M = M_0(1 - 2e^{\tau/T_1})$$

4-Fluorobenzoic acid, triethylamine salt **(13-**triethylamine**)**

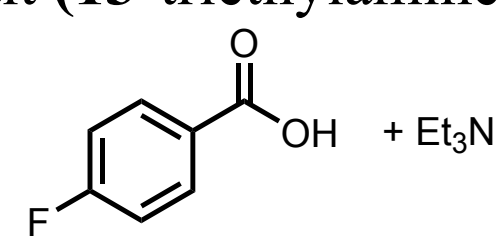

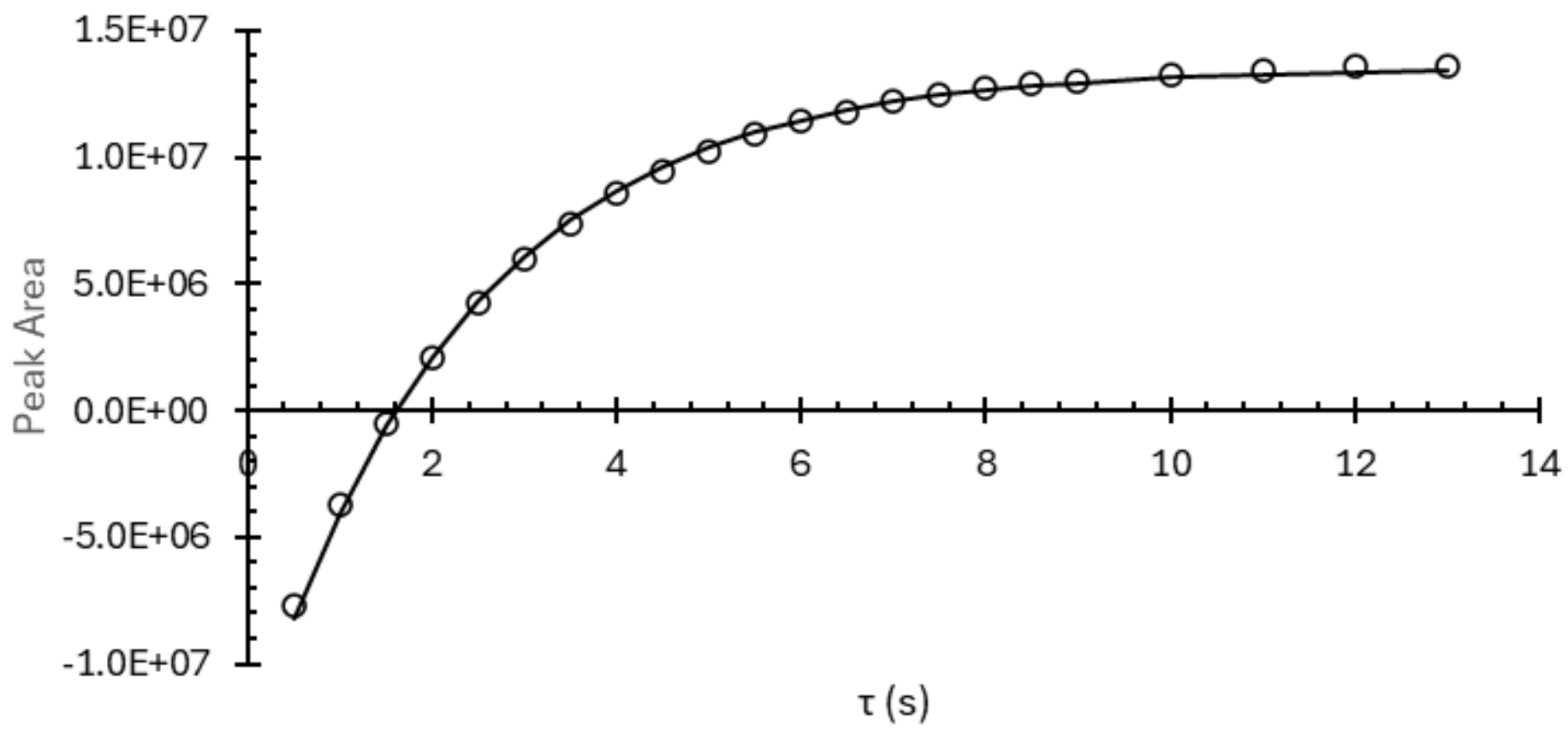

Figure S65. Inversion-recovery experiment for **13**-triethylamine.

Acquisition parameters
Number of scans (NS) = 1
Spectral center (o1p) = -108.8 ppm
Spectral width (sw) = 241.5 ppm
Acquisition time (AQ) = 0.577 sec
Relaxation delay (d1) = 60 sec
Fitting parameters
Function f(t) = M0*(1-2*exp(-t/T1))
Fitting results in 95% confidence bonds (R^2 = 0.999)
T_1 = 2.31 ± 0.04 (sec)
M_0 = (1.35 ± 0.01) × 10^7

4-Fluoro-*N*-(4-methylbenzoyl)-*N*-(*p*-tolyl)benzamide **(15)**

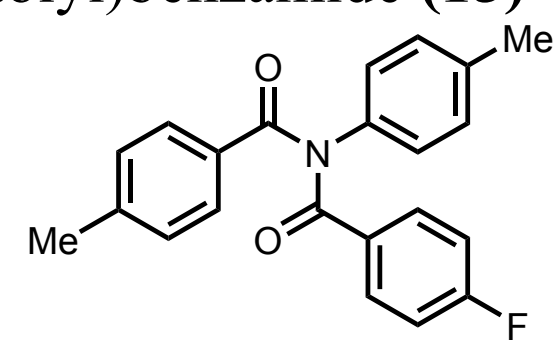

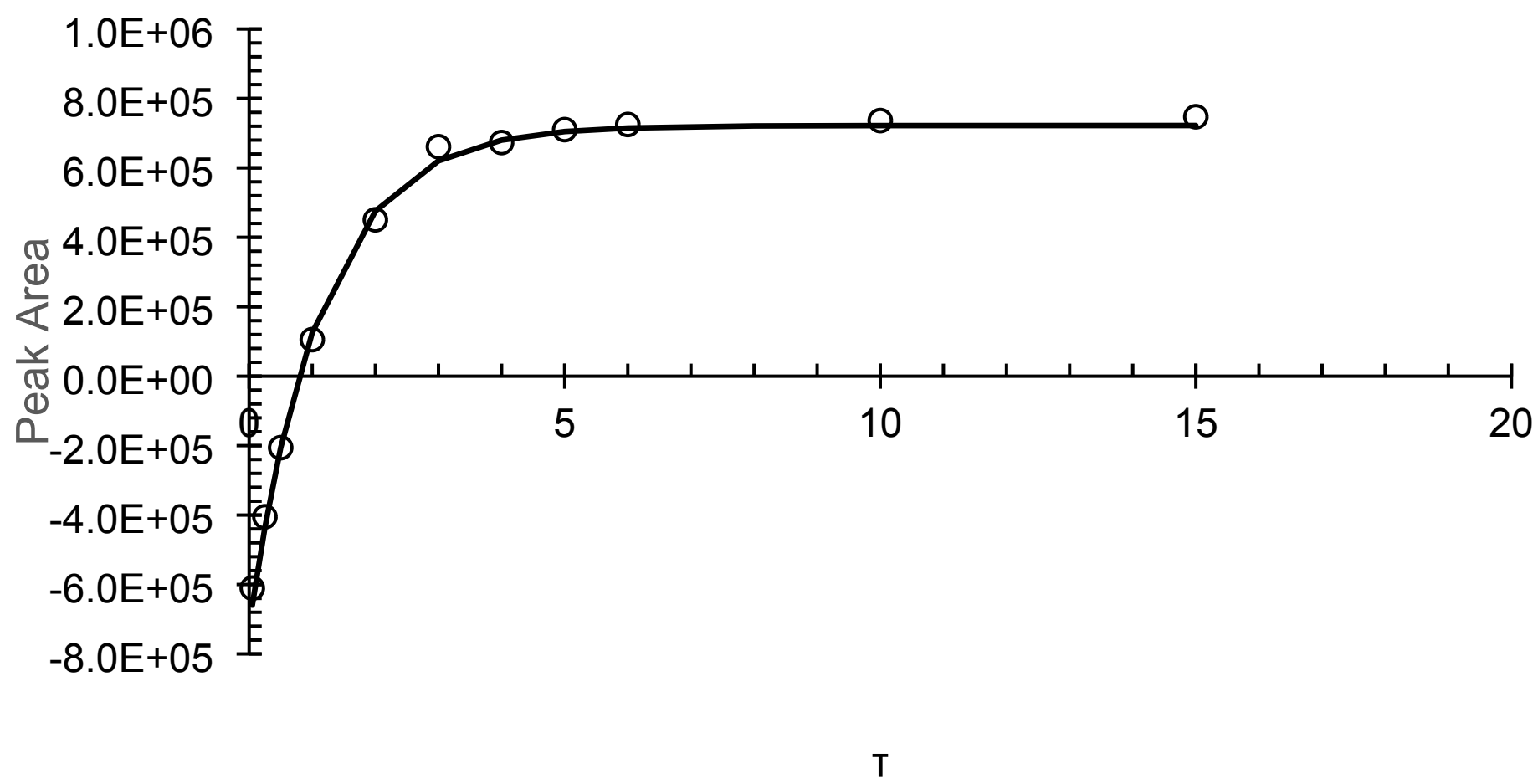

Figure S66. Inversion-recovery experiment for **15**.

Acquisition parameters
    Number of scans (NS) = 1
Spectral center (o1p) = -106.0 ppm
Spectral width (sw) = 241.5 ppm
Acquisition time (AQ) = 0.577 sec
    Relaxation delay (d1) = 60 sec
Fitting parameters
    Function f(t) = M0*(1-2*exp(-t/T1))
Fitting results in 95% confidence bonds (R^2 = 0.999)
    $T_1 = 1.13 \pm 0.07$ (sec)
    $M_0 = (7.2 \pm 0.3) \times 10^5$

1,2-Difluorobenzene

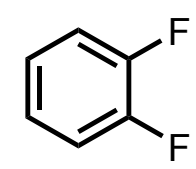

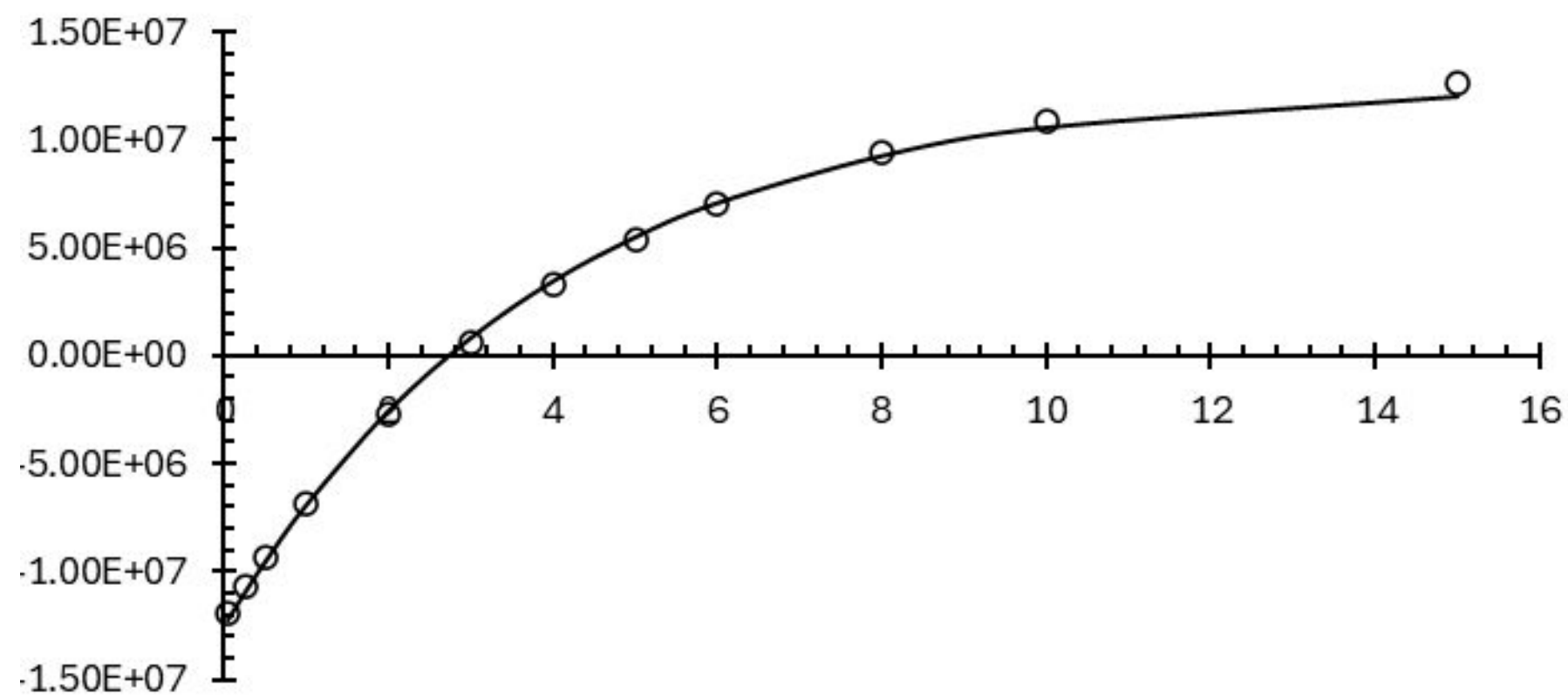

Figure S67. Inversion-recovery experiment for 1,2-difluorobenzene.

Acquisition parameters
Number of scans (NS) = 1
Spectral center (o1p) = -138.3 ppm
Spectral width (sw) = 241.5 ppm
Acquisition time (AQ) = 0.577 sec
Relaxation delay (d1) = 60 sec
Fitting parameters
Function f(t) = M0*(1-2*exp(-t/T1))
Fitting results in 95% confidence bonds (R^2 = 0.999)
$T_1 = 3.9 \pm 0.1$ (sec)
$M_0 = (1.26 \pm 0.03) \times 10^7$

9. Video Captions

	**Video S1.** Switching syringe housing for multiple injection sequences.<br>url: https://doi.org/10.6084/m9.figshare.32811101
Agitated, spinning	**Video S2.** Demonstration video for mixing efficiency, with agitation, spinning. Left to right: A: simple capillary; B: ringed capillary; C: bulbed capillary; D: arrowhead capillary; E: collared capillary.<br>url: https://doi.org/10.6084/m9.figshare.31955742
Agitated, not spinning	**Video S3.** Demonstration video for mixing efficiency, with agitation, not spinning. Left to right: A: simple capillary; B: ringed capillary; C: bulbed capillary; D: arrowhead capillary; E; collared capillary.<br>url: https://doi.org/10.6084/m9.figshare.31955796
Not agitated, spinning	**Video S4.** Demonstration video for mixing efficiency, without agitation, spinning. Left to right: A: simple capillary; B: ringed capillary; C: bulbed capillary; D: arrowhead capillary; E; collared capillary.<br>url: https://doi.org/10.6084/m9.figshare.31955778

Not agitated, not spinning	**Video S5.** Demonstration video for mixing efficiency, without agitation, not spinning. Left to right: A: simple capillary; B: ringed capillary; C: bulbed capillary; D: arrowhead capillary; E; collared capillary.<br>url: https://doi.org/10.6084/m9.figshare.31955763
	**Video S6.** Fusing a glass collar onto the capillary tip using a blowtorch.<br>url: https://doi.org/10.6084/m9.figshare.32811116
	**Video S7.** Opening the capillary tip using a 0.5 mm drill bit.<br>url: https://doi.org/10.6084/m9.figshare.32811131

10. NMR Spectra

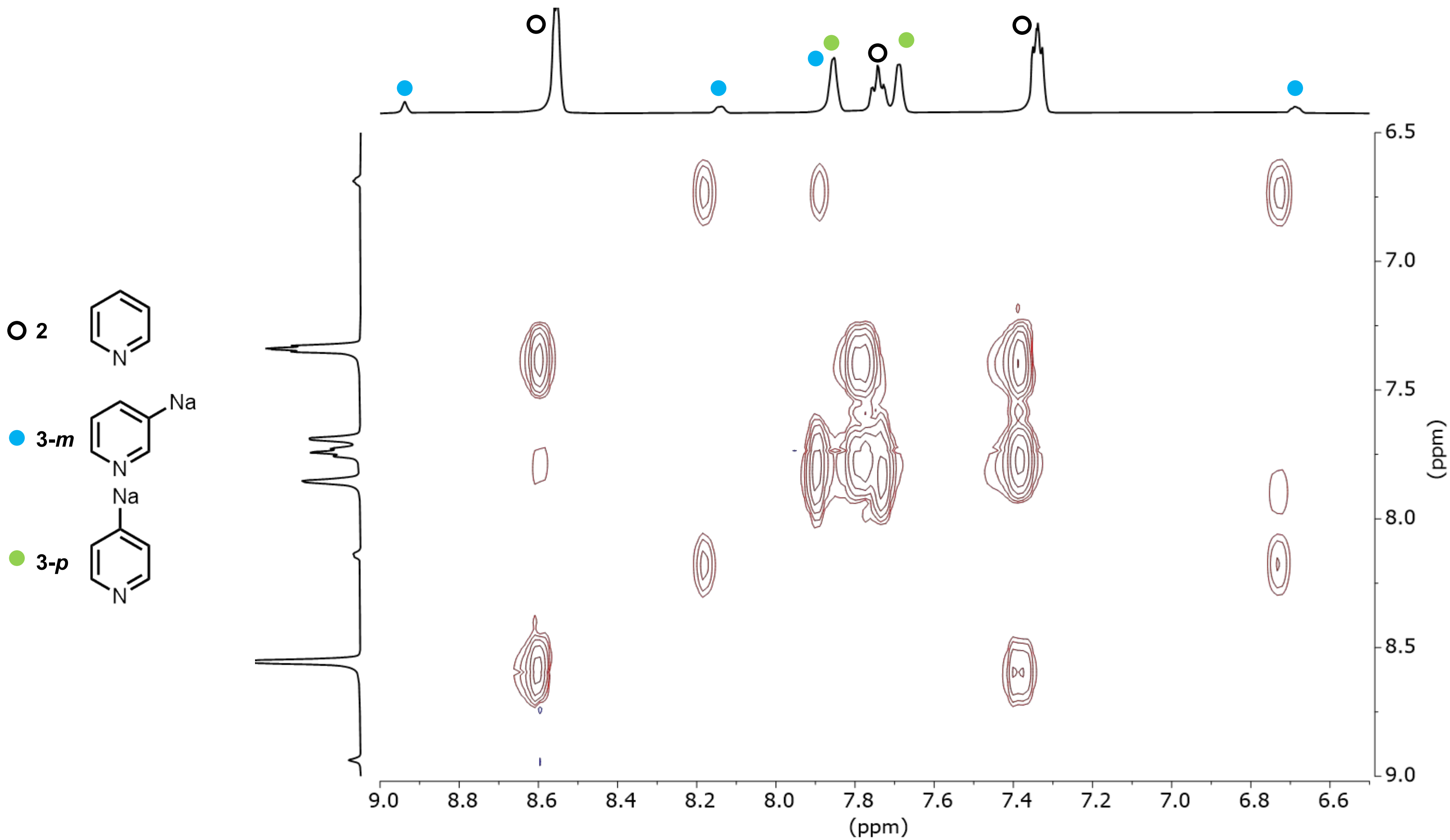

Figure S68. ^{1}H-^{1}H COSY of **2, 3-*m***, and **3-*p***.

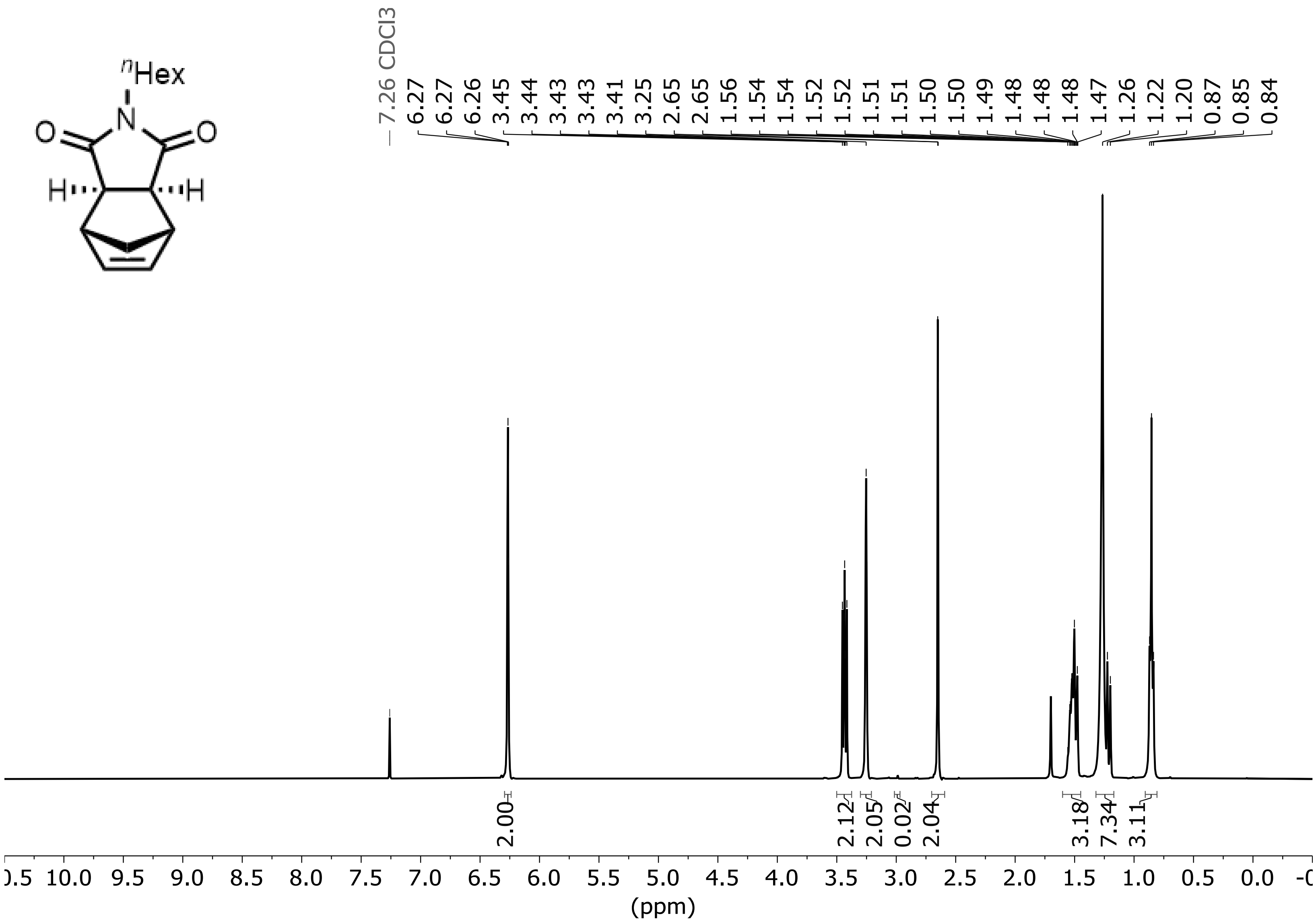

Figure S69. ^{1}H NMR spectrum of **6** (400 MHz, $CDCl_3$).

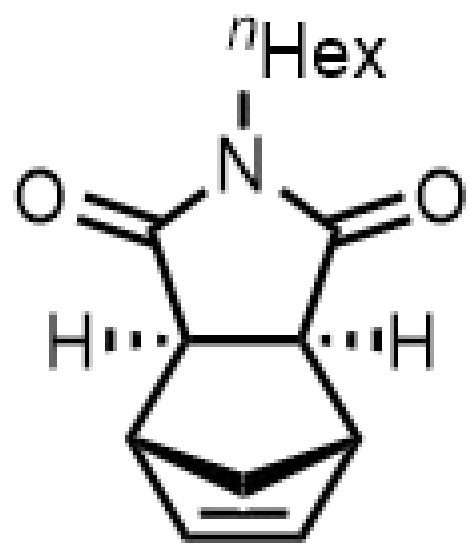

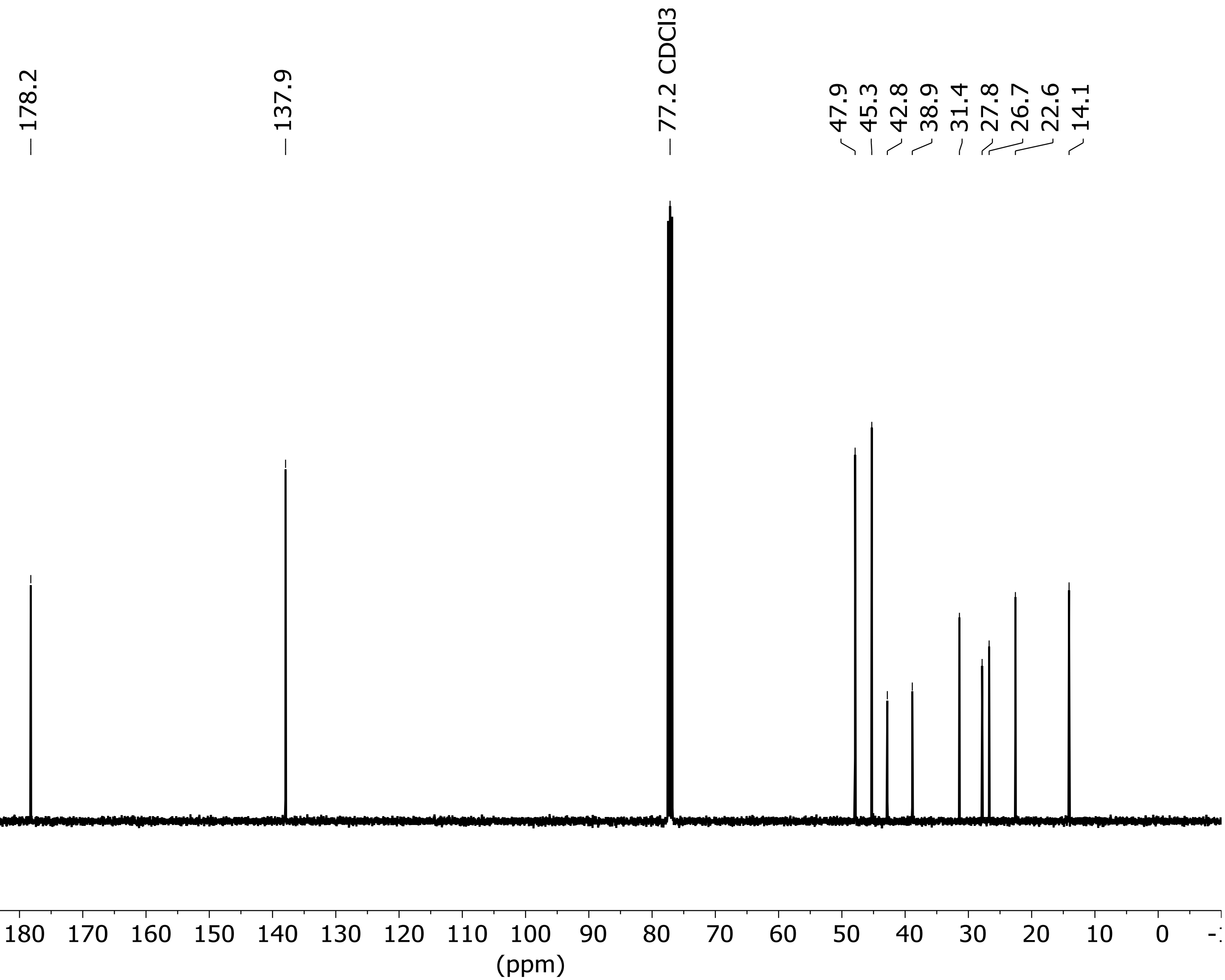

Figure S70. ^{13}C NMR spectrum of **6** (101 MHz, $CDCl_3$).

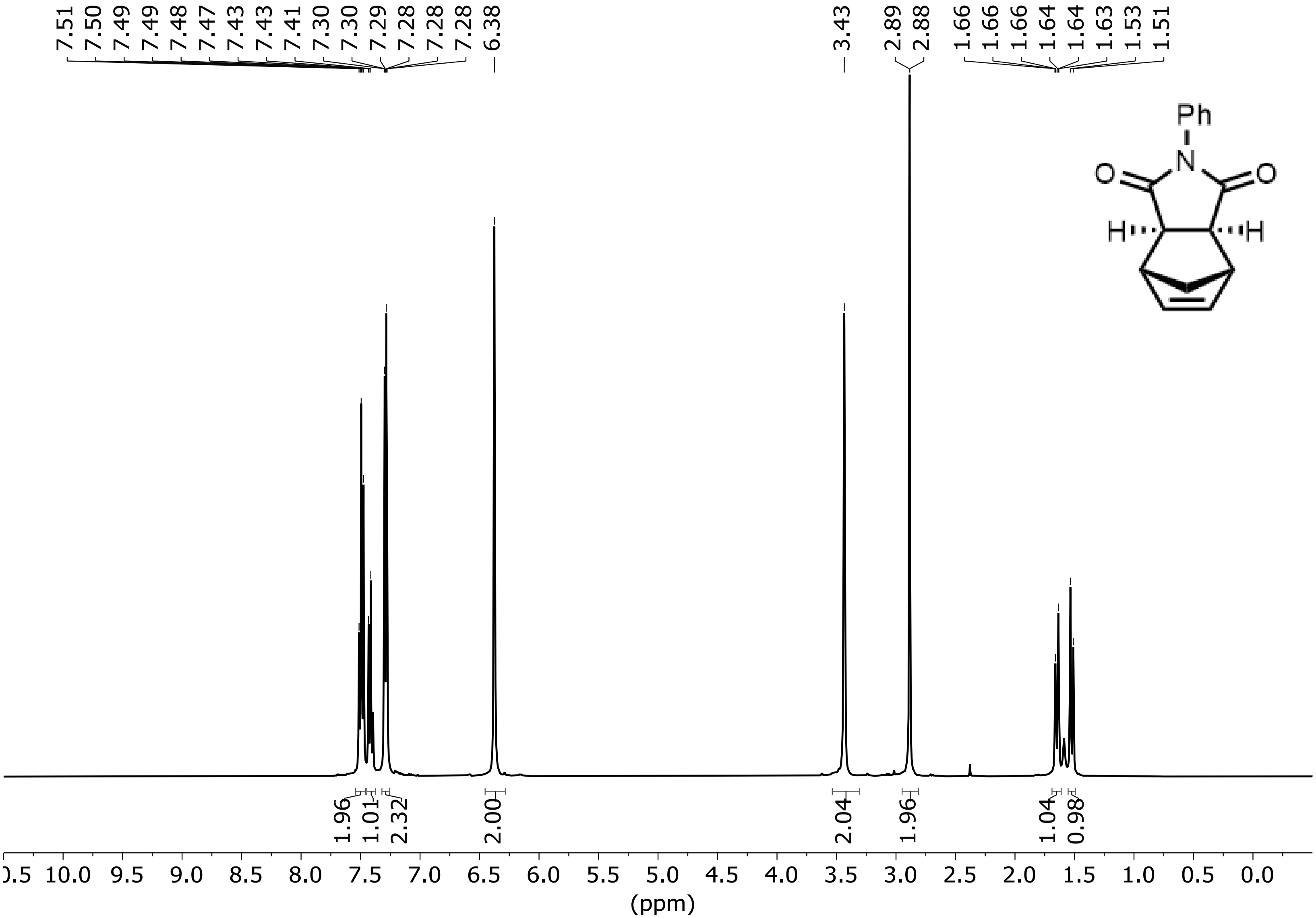

Figure S71. ^{1}H NMR spectrum of **8** (400 MHz, $CDCl_3$).

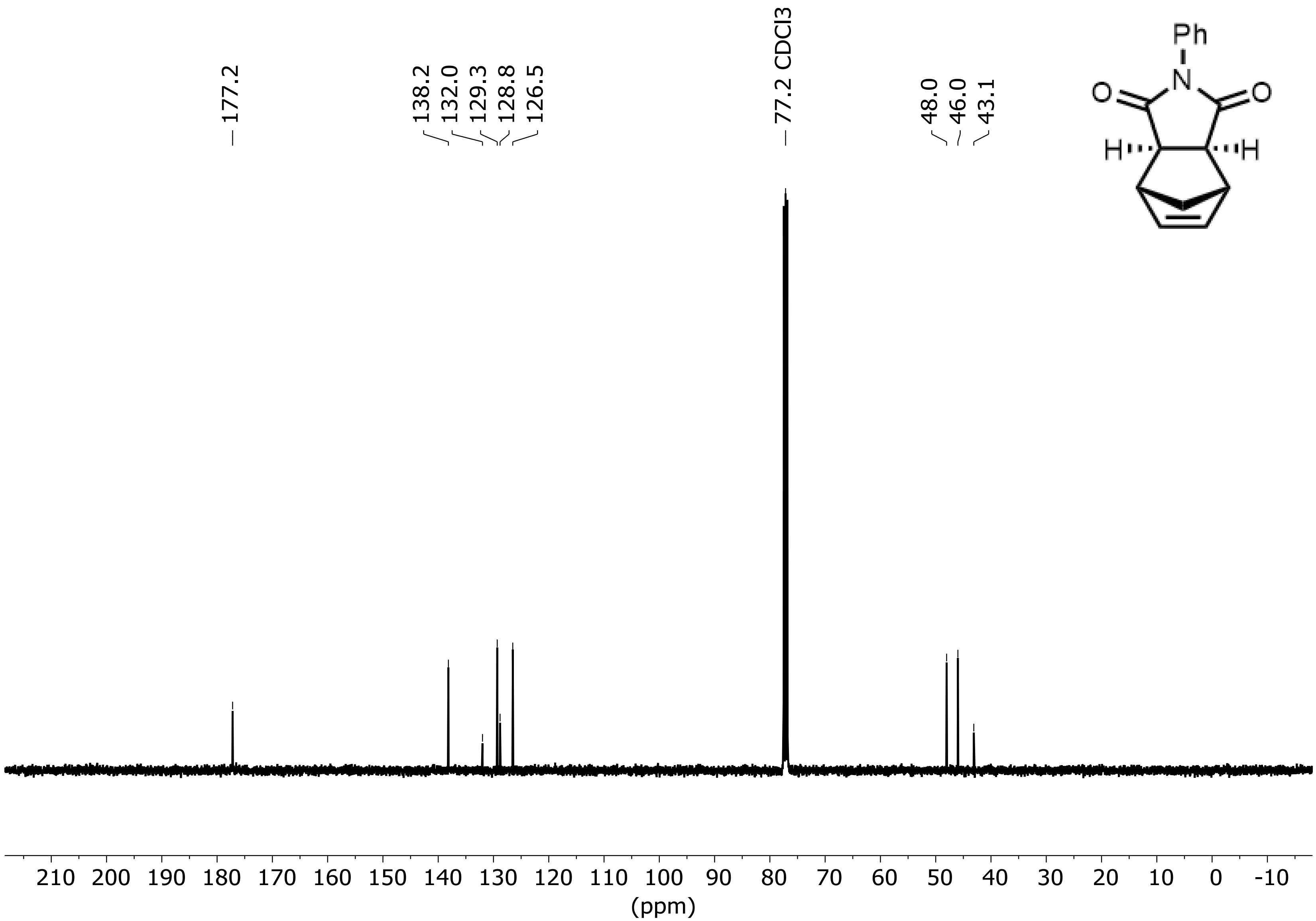

Figure S72. ^{13}C NMR spectrum of **8** (101 MHz, $CDCl_3$).

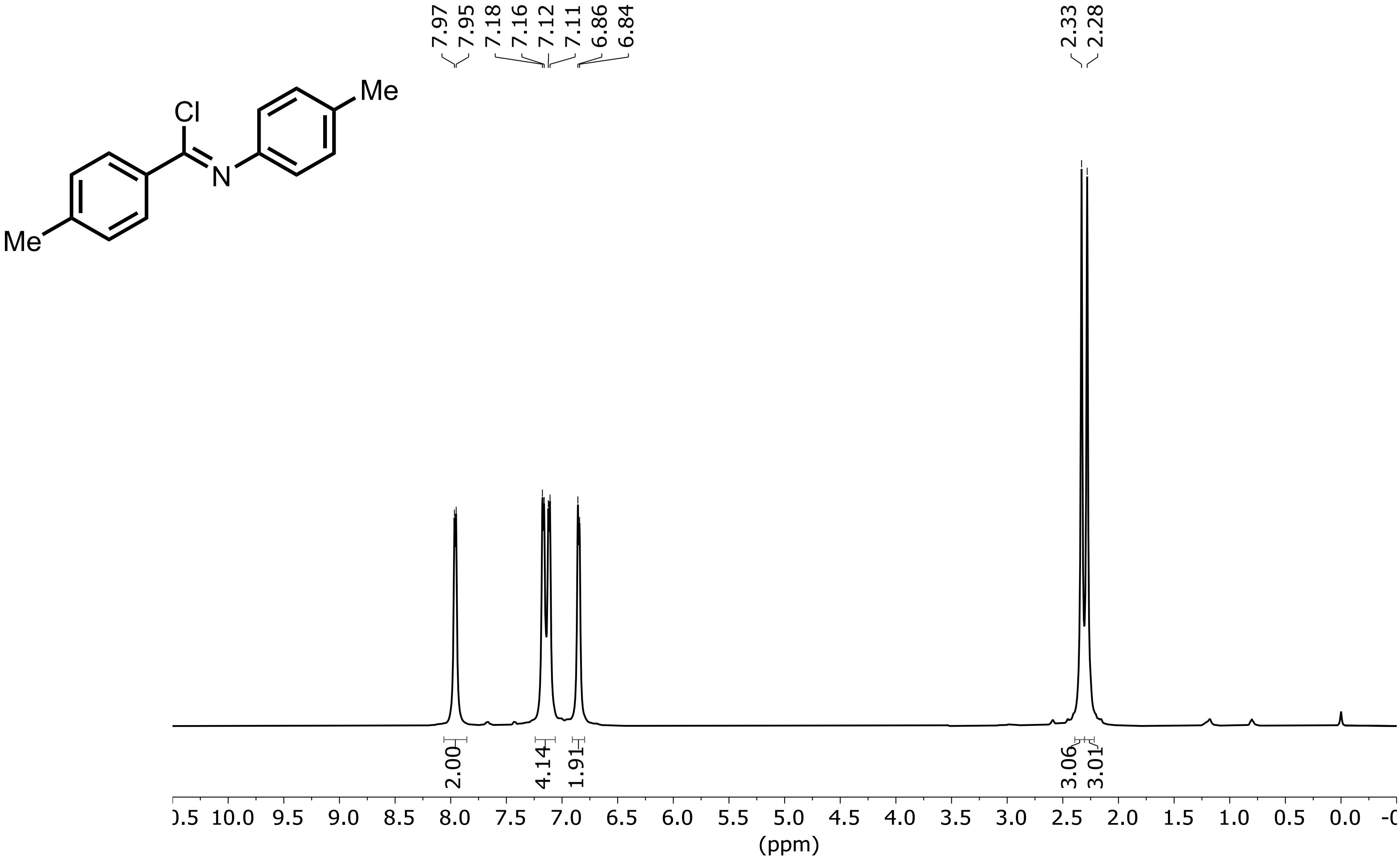

Figure S73. ^{1}H NMR spectrum of **12** (500 MHz, $CDCl_3$).

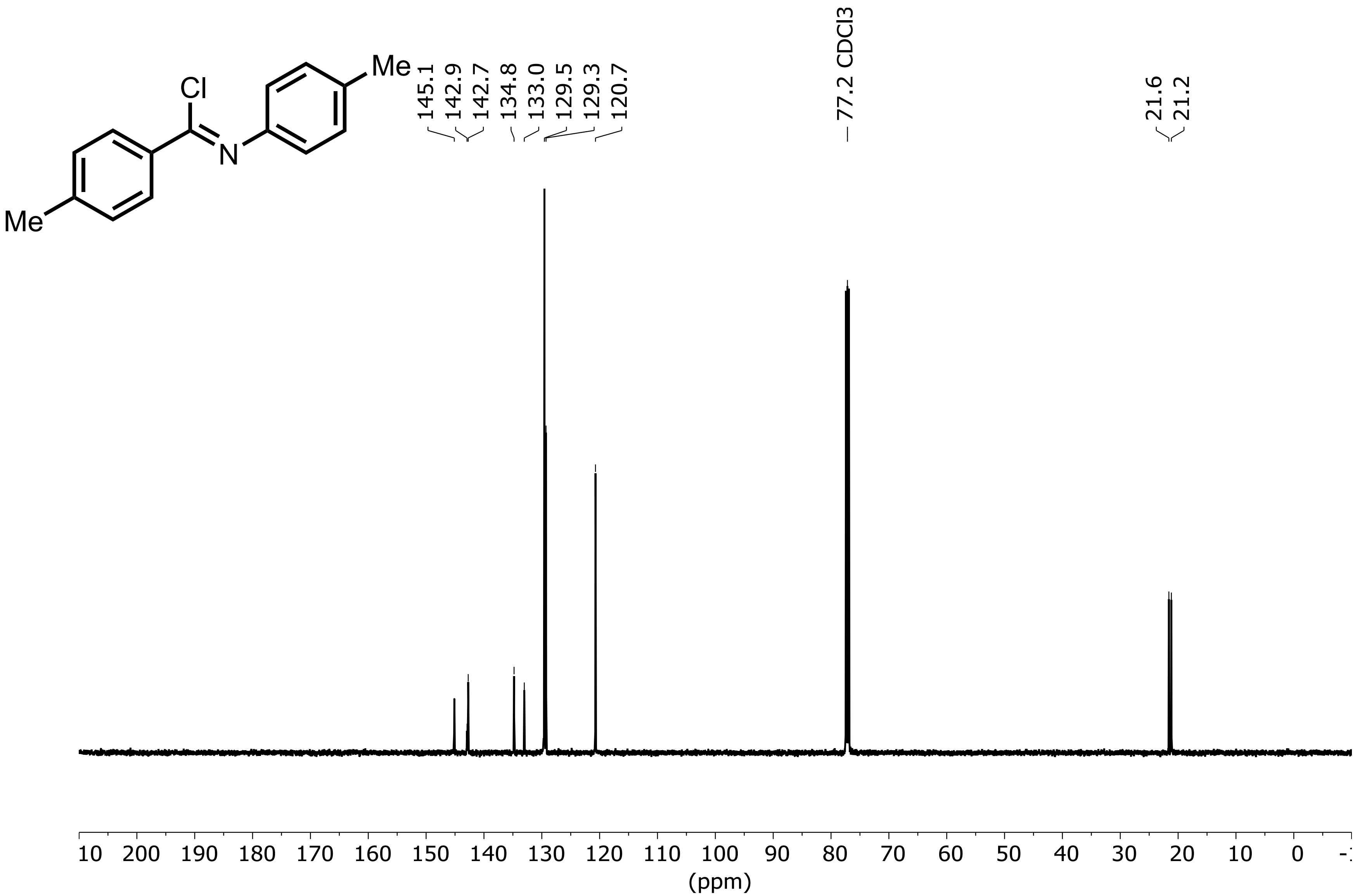

Figure S74. ^{13}C NMR spectrum of **12** (126 MHz, $CDCl_3$).

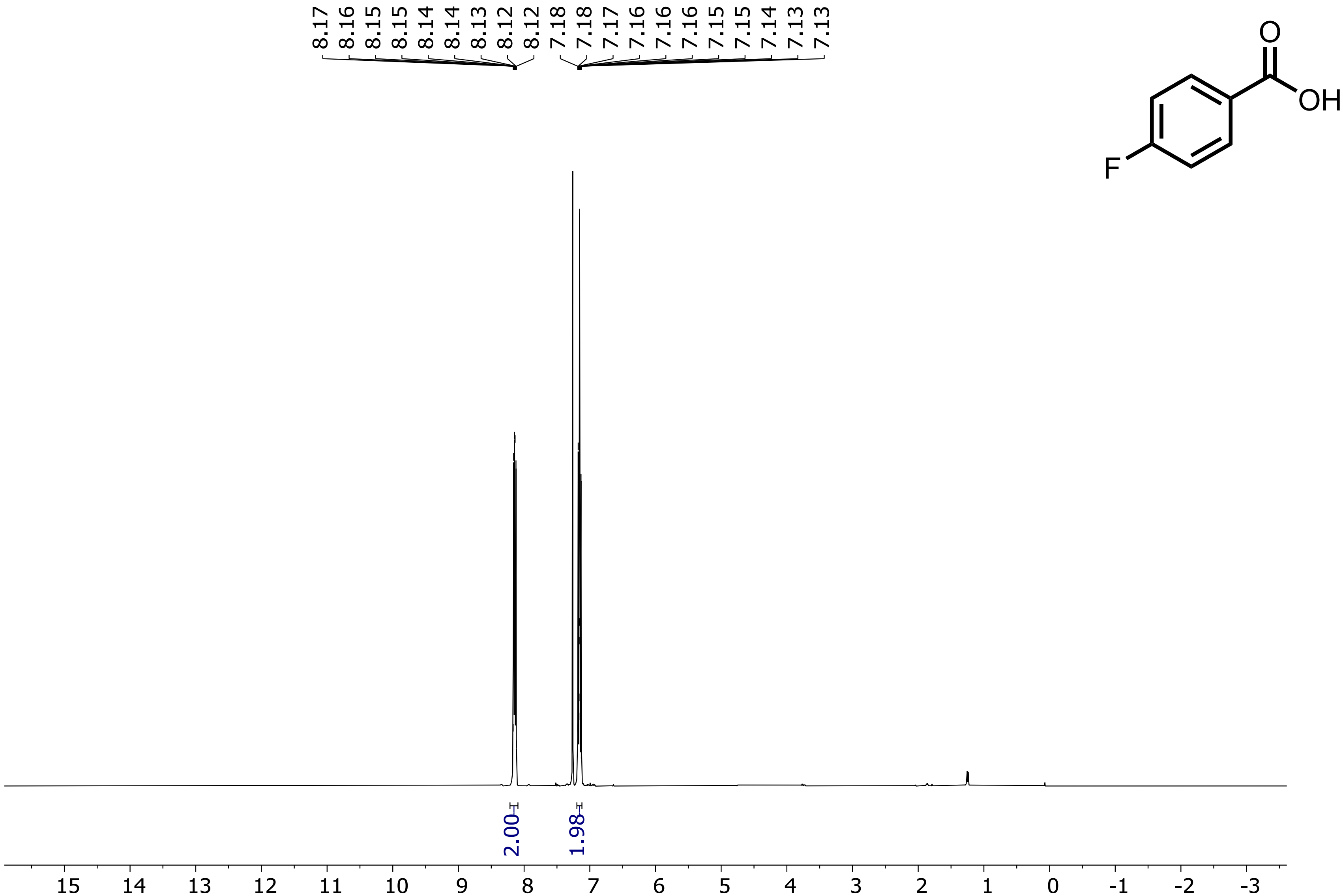

Figure S75. ^{1}H NMR spectrum of **13** (400 MHz, CDCl3).

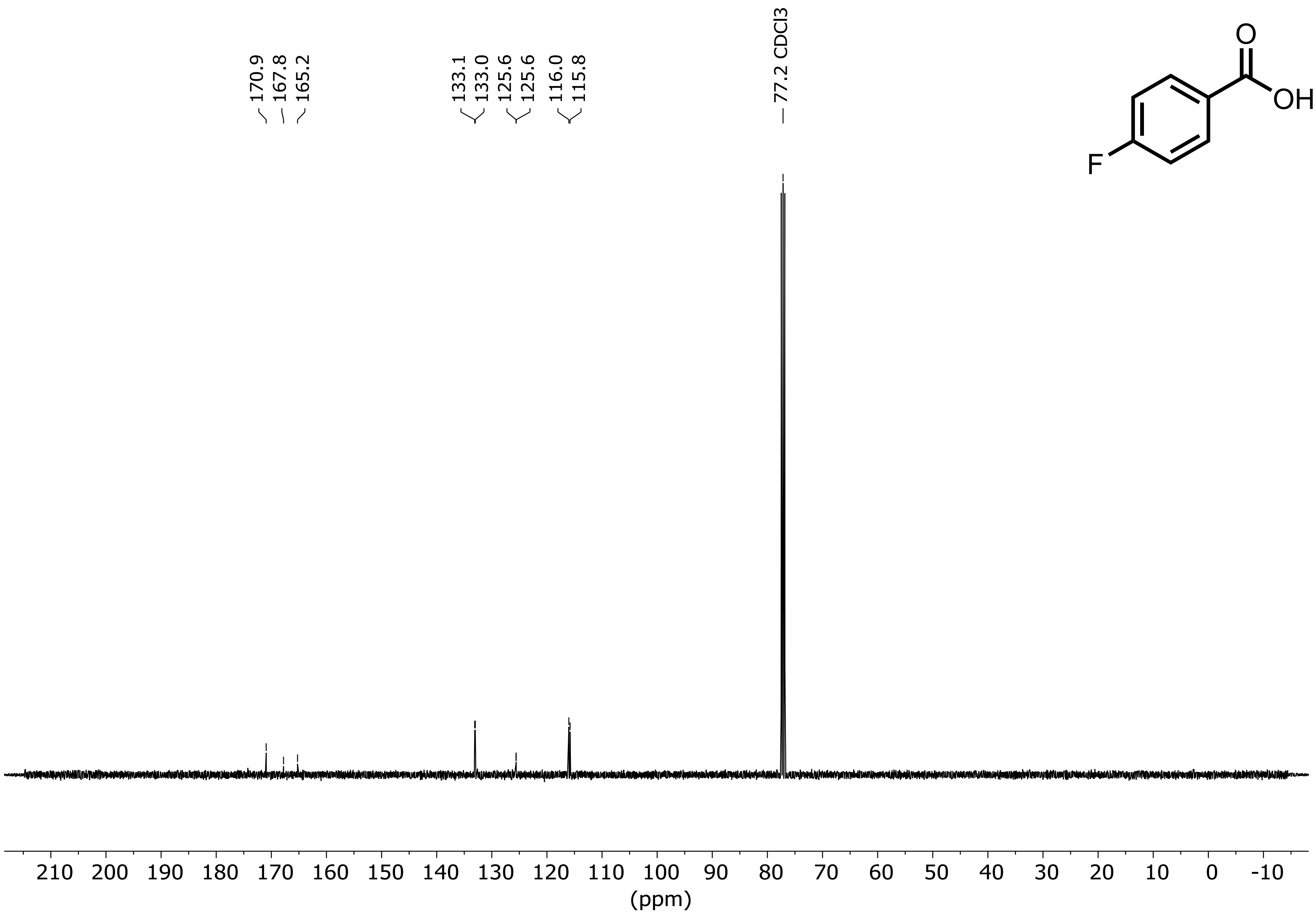

Figure S76. ^{13}C NMR spectrum of **13** (101 MHz, $CDCl_3$).

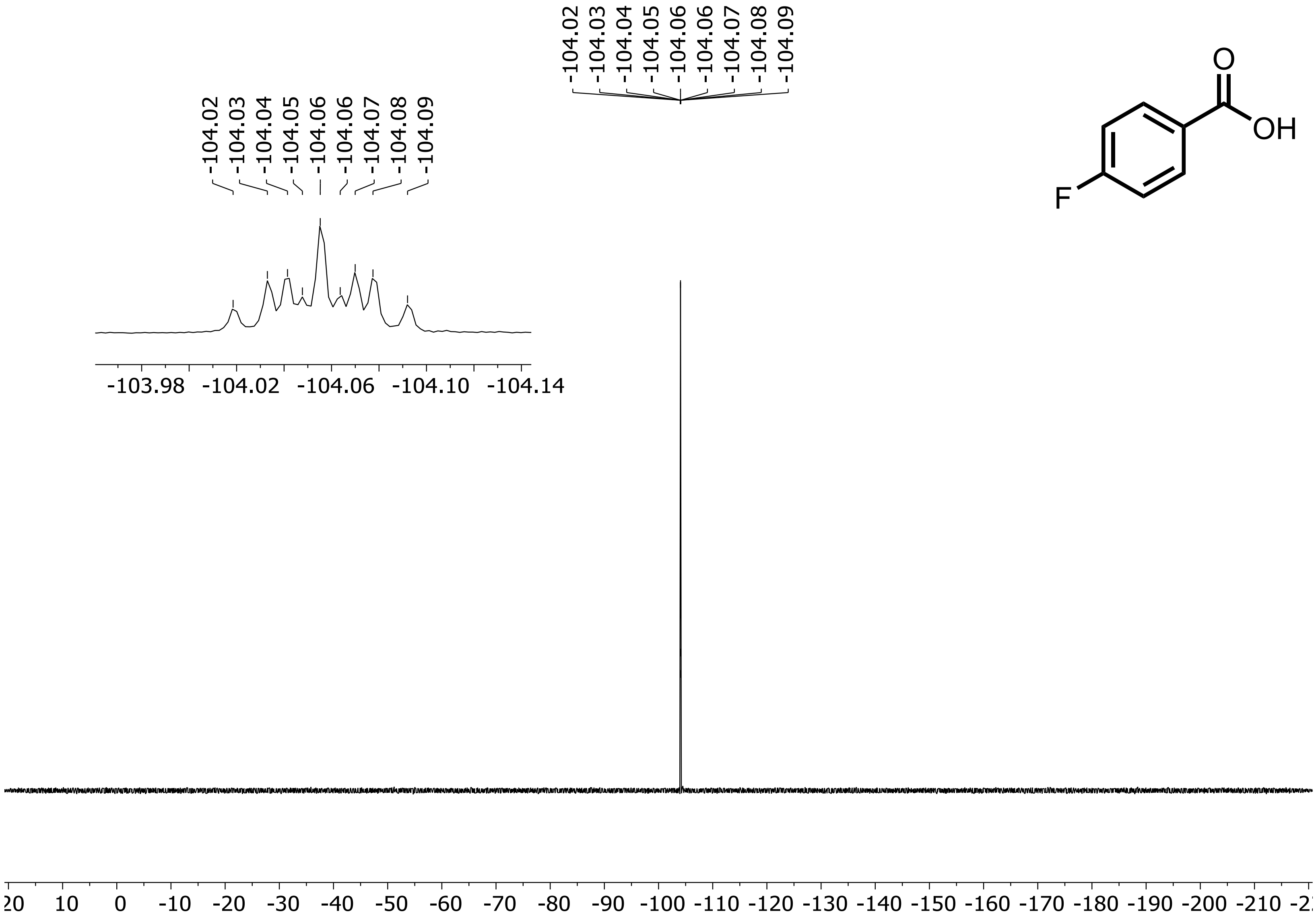

Figure S77. ^{19}F NMR spectrum of **13** (376 MHz, $CDCl_3$).

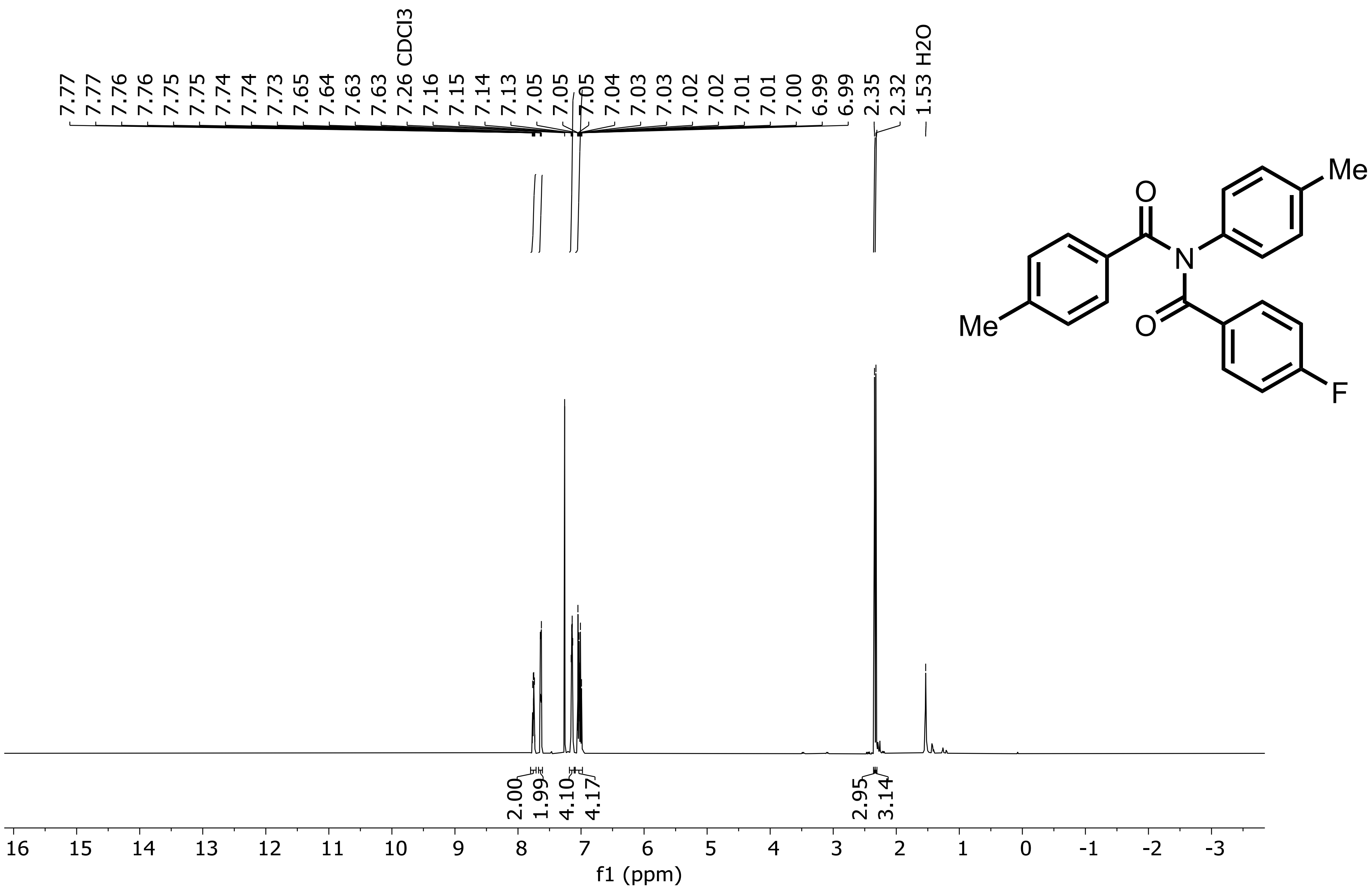

Figure S78. ^{1}H NMR spectrum of **15** (500 MHz, $CDCl_3$).

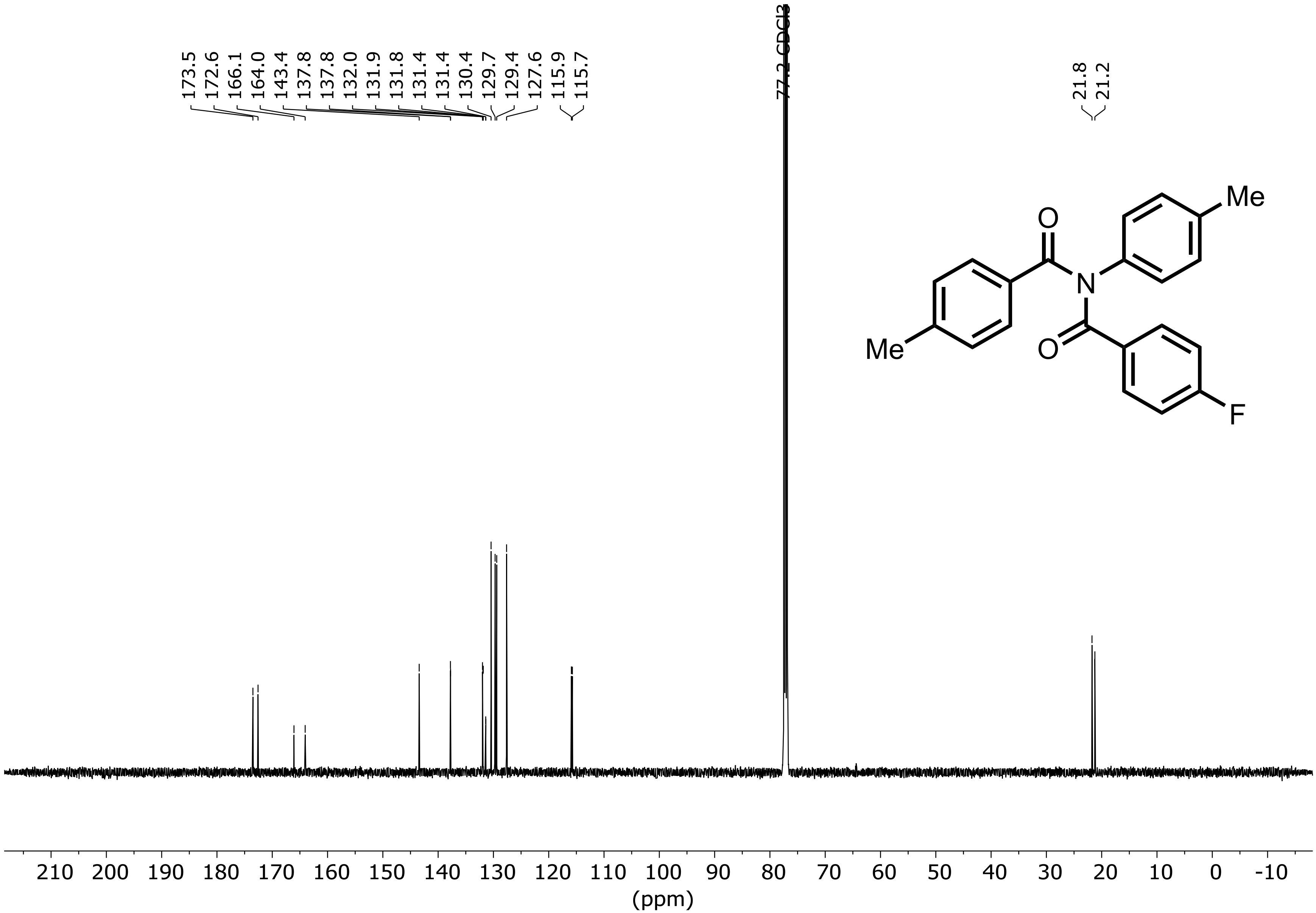

Figure S79. ^{13}C NMR spectrum of **15** (126 MHz, $CDCl_3$).

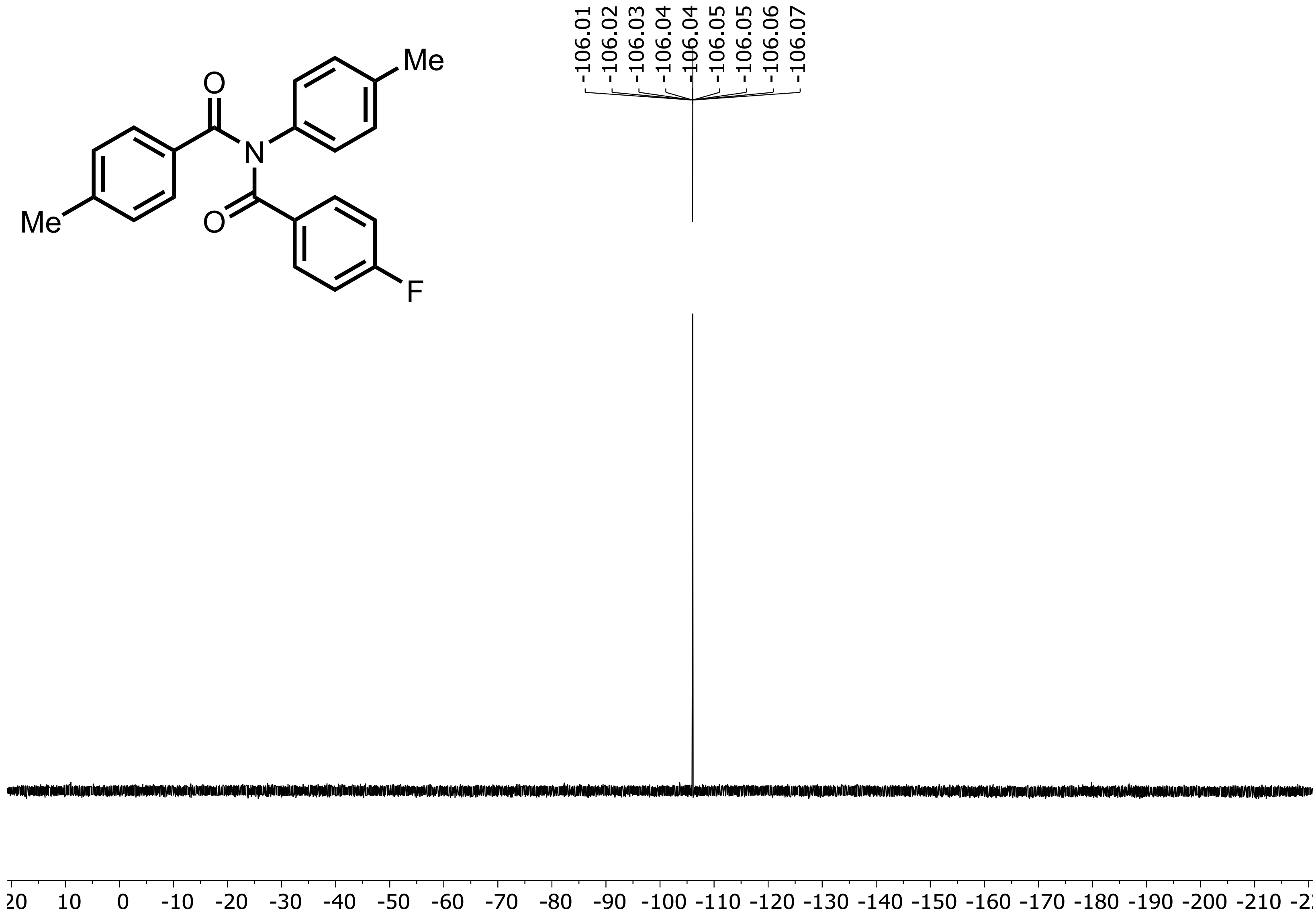

Figure S80. ^{19}F NMR spectrum of **15** (471 MHz, $CDCl_3$).

11. References

(1) Tortajada, A.; Anderson, D. E.; Hevia, E. Gram-Scale Synthesis, Isolation and Characterisation of Sodium Organometallics: *N* BuNa and NaTMP. *Helv. Chim. Acta* **2022**, *105* (8), e202200060. https://doi.org/10.1002/hlca.202200060.

(2) Hsu, H.; Kang, S.; Chen, C.; Sk, M. R.; Thomas, A. A. Functionalization of Pyridines at the C4 Position *via* Metalation and Capture. *Angew. Chemie Int. Ed.* **2025**, *64* (13), e202424172. https://doi.org/10.1002/anie.202424172.

(3) Kim, K. O.; Choi, T. L. Synthesis of Dendronized Polymers via Macromonomer Approach by Living ROMP and Their Characterization: From Rod-like Homopolymers to Block and Gradient Copolymers. *Macromolecules* **2013**, *46* (15), 5905–5914. https://doi.org/10.1021/ma401132u.

(4) Seong, H.; Chen, Z.; Emrick, T.; Russell, T. P. Reconfiguration and Reorganization of Bottlebrush Polymer Surfactants. *Angew. Chemie Int. Ed.* **2022**, *61* (19), e202200530. https://doi.org/10.1002/anie.202200530.

(5) Roslund, M. U.; Tähtinen, P.; Niemitz, M.; Sjöholm, R. Complete Assignments of the 1H and 13C Chemical Shifts and JH,H Coupling Constants in NMR Spectra of d-Glucopyranose and All d-Glucopyranosyl-d-Glucopyranosides. *Carbohydr. Res.* **2008**, *343* (1), 101–112. https://doi.org/10.1016/j.carres.2007.10.008.

(6) Das, R.; Kapur, M. Palladium-Catalyzed, Ortho-Selective C-H Halogenation of Benzyl Nitriles, Aryl Weinreb Amides, and Anilides. *J. Org. Chem.* **2017**, *82* (2), 1114–1126. https://doi.org/10.1021/acs.joc.6b02731.

(7) Zhu, L.; Le, L.; Yan, M.; Au, C. T.; Qiu, R.; Kambe, N. Carbon-Carbon Bond Formation of Trifluoroacetyl Amides with Grignard Reagents via C(O)-CF3 Bond Cleavage. *J. Org. Chem.* **2019**, *84* (9), 5635–5644. https://doi.org/10.1021/acs.joc.9b00583.

(8) Hoye, T. R.; Eklov, B. M.; Voloshin, M. No-D NMR Spectroscopy as a Convenient Method for Titering Organolithium (RLi), RMgX, and LDA Solutions. *Org. Lett.* **2004**, *6* (15), 2567–2570. https://doi.org/10.1021/ol049145r.

(9) Yoshida, M.; Katagiri, Y.; Zhu, W. Bin; Shishido, K. Oxidative Carboxylation of Arylaldehydes with Water by a Sulfoxylalkyl-Substituted N-Heterocyclic Carbene Catalyst. *Org. Biomol. Chem.* **2009**, *7* (19), 4062–4066. https://doi.org/10.1039/b910187j.
